# Supplementary material for: A modular synthesis of tetracyclic meroterpenoid antibiotics
Source: Nat Commun. 2017 Dec 12;8:2083. doi: 10.1038/s41467-017-02061-7 (PMC5727219; doi:10.1038/s41467-017-02061-7)
Supplement: Supplementary file 1 — Supplementary Information [file 41467_2017_2061_MOESM1_ESM.pdf]

Supplementary Methods

General Experimental Details

All reactions were performed in flame-dried glassware fitted with rubber septa under a positive pressure of argon, unless otherwise noted. Air- and moisture-sensitive liquids were transferred via syringe or stainless steel cannula through rubber septa. Solids were added under inert gas counter flow or were dissolved in appropriate solvents. Low temperature-reactions were carried out in a Dewar vessel filled with a cooling agent: acetone/dry ice (−78 °C), H_2O/ice (0 °C). Reaction temperatures above room temperature were conducted in a heated oil bath. The reactions were magnetically stirred and monitored by NMR spectroscopy or analytical thin-layer chromatography (TLC), using aluminum plates precoated with silica gel (0.25 mm, 60-Å pore size, Merck) impregnated with a fluorescent indicator (254 nm). TLC plates were visualized by exposure to ultraviolet light (UV), were stained by submersion in aqueous potassium permanganate solution ($KMnO_4$), ceric ammonium molybdate solution (CAM) or p-anisaldehyde solution (Anis), and were developed by heating with a heat gun. Flash-column chromatography on silica gel was performed as described by Still et al.,[1] employing silica gel (60 Å, 40–63 µm, Merck KGaA). The yields refer to chromatographically and spectroscopically (^1H and ^{13}C NMR) pure material.

Materials

Tetrahydrofuran (THF) and diethyl ether (Et_2O) were distilled under N_2 atmosphere from sodium and benzophenone prior to use. Dichloromethane (CH_2Cl_2), triethylamine (Et_3N), diisopropylamine (DIPA) and Hünig's base (DIPEA) were distilled under nitrogen atmosphere from CaH_2 prior to use. Dimethyl sulfoxide (DMSO), acetonitrile (MeCN), acetone, toluene, chlororform ($CHCl_3$) and methanol (MeOH) were purchased from Acros Organics as 'extra dry' reagents and used as received. All other reagents and solvents were purchased from chemical suppliers (Sigma-Aldrich, Acros Organics, Alfa Aesar, Strem Chemicals, ABCR) and were used as received. Solvents for extraction, crystallization and flash-column chromatography on silica gel were purchased in technical grade and distilled under reduced pressure prior to use. Lithium chloride was dried at 100 °C under vacuum (0.1 mmHg) for 12 h and stored in a drying oven at 150 °C (760 mmHg); the hot, dried solid was flame dried under vacuum (0.1 mmHg) for 4−5 min immediately prior to use. The molarity of n-butyllithium and t-butyllithium solutions was determined by titration against diphenylacetic acid as an indicator (average of three determinations).[2]

NMR spectroscopy

NMR spectra were measured on a Bruker Avance III HD 400 MHz spectrometer equipped with a CryoProbe™, Bruker AXR300, Varian VXR400 S, Bruker AMX600 or Bruker Avance HD 800. Proton chemical shifts are expressed in parts per million (ppm,: δ scale) and are referenced to residual proton in the NMR solvent ($CHCl_3$: δ 7.26, C_6D_5H: δ 7.16, CD_2Cl_2: δ 5.32). Carbon chemical shifts are expressed in parts per million (δ scale, assigned carbon atom) and are referenced to the carbon resonance of the NMR solvent ($CDCl_3$: δ 77.16, C_6D_6: δ 128.06, CD_2Cl_2: δ 54.00). ^1H NMR spectroscopic data are reported as follows: Chemical shift in ppm (multiplicity, coupling constants J (Hz), integration intensity, assigned proton). The multiplicities are abbreviated with s (singlet), br s (broad singlet), d (doublet), t (triplet), q (quartet) and m (multiplet). In case of combined multiplicities, the multiplicity with the larger coupling constant is stated first. Except for multiplets, the chemical shift of all signals, as well for centrosymmetric multiplets, is reported as the center of the resonance range. Additionally to ^1H and ^{13}C NMR measurements, 2D NMR techniques such as homonuclear correlation spectroscopy (COSY), total correlation spectroscopy (TOCSY), heteronuclear single quantum coherence (HSQC) and heteronuclear multiple bond coherence (HMBC) were used to assist signal assignment. For further elucidation of 3D structures of the products, nuclear Overhauser enhancement spectroscopy (NOESY) was conducted. Coupling constants J are reported in Hz. All raw fid files were processed and the spectra analyzed using the program MestReNOVA 9.1 from Mestrelab Research S. L.

Mass spectrometry

All mass spectra were measured by the analytic section of the Department of Chemistry, Ludwig-Maximilians-Universität München. Mass spectra were recorded on the following spectrometers (ionisation mode in brackets): MAT 95 (EI) and MAT 90 (ESI) from Thermo Finnigan GmbH. Mass spectra were recorded in high-resolution. The method used is reported at the relevant section of the experimental section.

IR spectroscopy

IR spectra were recorded on a PerkinElmer Spectrum BX II FT-IR system. If required, substances were dissolved in CH_2Cl_2 prior to direct application on the ATR unit. Data are represented as follows: frequency of absorption (cm^{-1}).

Optical rotation

Optical rotation values were recorded on a PerkinElmer 241 or Anton Paar MCP 200 polarimeter. The specific rotation is calculated according to Supplementary Equation 1.

$$[\alpha]_{\lambda}^{\varphi} = \frac{[\alpha] \cdot 100}{c \cdot d} \qquad (1)$$

Thereby, the wave length λ is reported in nm and the measuring temperature ϕ in °C. α represents the recorded optical rotation at the apparatus, c the concentration of the analyte in 10 mg/mL and d the length of the cuvette in dm. Thus, the specific rotation is given in $10^{-1}\cdot deg\cdot cm^{2}\cdot g^{-1}$. Usage of the sodium D line (λ = 589 nm) is indicated by D instead of the wavelength in nm. The respective concentration as well as the solvent is reported at the relevant section of the experimental section.

Melting Points

Melting points were determined on a B-450 melting point apparatus from BÜCHI Labortechnik AG. The values are uncorrected.

X-Ray Crystallographic Data

The data collections were performed either on an Oxford Diffraction Xcalibur diffractometer, on a Bruker D8Quest diffractometer or on a Bruker D8Venture at 100 K or at 173 K using MoKα-radiation (λ = 0.71073 Å, graphite monochromator). The CrysAlisPro software (version 1.171.33.41)[S8] was applied for the integration, scaling and multi-scan absorption correction of the data. The structures were solved by direct methods with SIR97[3] and refined by least-squares methods against F2 with SHELXL-97.[4] All non-hydrogen atoms were refined anisotropically. The hydrogen atoms were placed in ideal geometry riding on their parent atoms. Further details are summarized in the tables at the different sections.

Antibacterial assays

Overnight cultures of the bacteria were grown aerobically at 37 °C in Müller Hinton broth with added 1% glucose and pH 7.2 for Gram-negative strains, or with Trypticase soy yeast extract medium (TSY–30 g/l trypticase soy broth, 3 g/L yeast extract, pH 7.2) for Gram-positive strains. The cultures were adjusted to an OD600nm of 0.001, which resulted in a final start OD600nm of 0.0005 in the test. 25 µL of test culture was added to 25 µL of a serial dilution of the test compounds in the appropriate medium for the different strains in accordance with standardized procedures in 384 well plates (DIN 58940-7: Medical microbiology – susceptibility testing of microbial pathogens to antimicrobial agents – determination of the minimum bactericidal concentration (MBC) with the method of micro boullion dilution; text in German and English). Test compounds from stock solutions

in DMSO were used at final concentrations of 100, 50, 25, 12.5, 6,25, 3.125, 1.56, 0.78, 0.39, 0.2 µM. As positive control compounds, Linezolid (both MRSA strains), Ciprofloxacin (*E. faecium, E. coli, A. baumannii, K. pneumonia*), Amikacin (P. aeruginosa) and Amphotericin (*C. albicans*) were applied. The highest DMSO concentration in the assay was 1%, which had no apparent effect on the growth of the bacteria. After an incubation time of 18 h at 37 °C under moist conditions, the optical density at 600 nm was measured with a Fusion Universal Microplate Analyser (Perkin–Elmer, Waltham, USA). The lowest concentration that completely suppressed growth defined the MIC values. The following bacterial strains were used. Gram-negative: *Acinetobacter baumannii* (DSM 30007), *Escherichia coli* (DSM 1116), *Klebsiella pneumoniae* (DSM 11678) and *Pseudomonas aeruginosa* PA7 (DSM 24068). Gram-positive: *Enterococcus faecium* (DSM 20477), *Staphylococcus aureus* MRSA (clinical isolate, RKI 11-02670) and *Staphylococcus aureaus* MRSA (DSM 11822). The EC_{50} and MIC values were determined by curve fitting with Sigma Plot.

Antiproliferative assays

The effect of compounds on cell viability was probed with a WST-1 test using the procedure of Ishiyama et al.[5] as modified by Sasse et al.[6] The following cell lines were used: mouse fibroblast cell line L929 (DSM ACC 2), human cervix carcinoma cell line KB-3-1 (DSM ACC 158) and human breast cancer cell line MCF-7 (DSM ACC 115). In addition, the conditional immortalized human fibroblast cell line FS4-LTM (InScreenex, Braunschweig, Germany) was used without doxycyclin to induce primary cell-like behavior (Pub. No.: US2011/0189142 A2). The subconfluent cells were briefly washed with Earle's Balanced Salt Solution (Gibco) without Ca and Mg, trypsinized and re-suspended in Dulbecco's modified eagle's medium that contained 5% fetal bovine serum (FBS; L929, KB-31, FS4-LTM) or Roswell Park Memorial Institute medium that contained 5% FBS, 0.5% Minimum Essential Medium Non-Essential Amino Acids, Gibco (MEM NEAA), 0.5% GlutaMAX (Gibco) and insulin at 5 µg/mL (MCF-7). 25 µL of serial dilutions of the test compounds (100–0.2 µM), that were made with a pipetting robot (epMotion, Eppendorf, Hamburg, Germany), were added to 25 µL aliquots of a cell suspension (1500 cells for KB3-1 and L929, 3000 cells for MCF-7 and 7500 cells for FS4-LTM) in 384 well microtiter plates. Blank and solvent controls were incubated under identical conditions. After an incubation period of 5 days (for L929, KB-3-1, and MCF-7) or 24h (for FS4-LTM), 3 µL WST-1 (ready to use solution by Roche) was added. The incubation time of the plates at 37 °C varied between the cell lines from 20 min for KB-3-1, L929 for 30 min, FS4-LTM for 1 h and 2 h for MCF-7 before measuring absorbance at 450 nm (reference 600 nm) with an Infinite 200 PRO plate reader (Tecan, Männedorf, Switzerland). As positive control compounds, Auranofin and Staurosporin were applied. The absorbance of the solvent control was set to 100%. The EC_{50} values were determined with Sigma Plot.

Synthesis of (+)-Stachyflin (1), 38 and 39

Diene 10

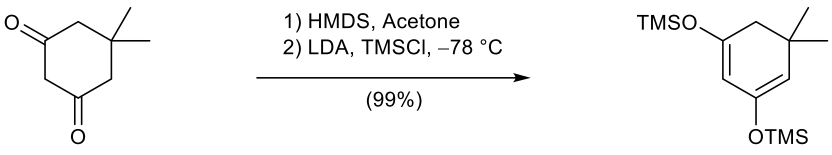

S01 **10**

To a suspension of dimedone (**S01**) (24.8 g, 177 mmol, 1 equiv) in dichloromethane (450 mL) was added hexamethyldisilazane (HMDS) (51.8 mL, 244 mmol, 1.38 equiv) and the resulting solution was stirred at 23 °C. After 23 h, the solution was concentrated and the residue was added dropwise to a solution of lithium diisopropylamine (195 mmol, 1.10 equiv), itself freshly prepared by the addition of *n*-butyllithium (2.4 M in hexanes, 81.1 mL, 195 mmol, 1.10 equiv) to a solution of diisopropylamine (27.5 mL, 195 mmol, 1.10 equiv) in tetrahydrofuran (350 mL) at –78 °C, over a period of 30 min. After 60 min, chlorotrimethylsilane (21.1 g, 195 mmol, 1.10 equiv) was added and the reaction mixture was allowed to warm to 23 °C. After 90 min, the reaction mixture was filtered and the filtrate was concentrated. The residue was dissolved in pentane, the so-obtained mixture was filtered through a plug of Celite® and the solvent was removed under reduced pressure. This process was repeated twice, yielding **10** (50.3 g, 99%) as a pale yellow oil. The obtained characterization data were in full agreement with the values previously reported.[7]

Dimethyl-3,5-dihydroxyphthalate 11

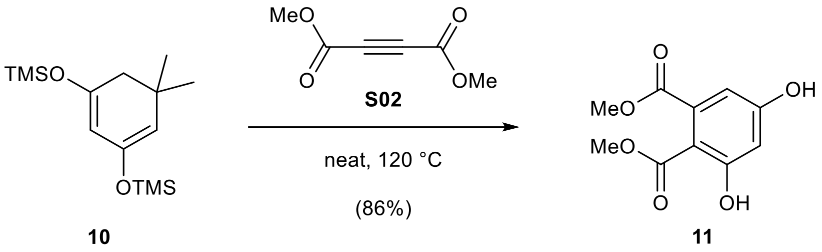

A mixture of dimethyl acetylenedicarboxylate (**S02**) (12.6 g, 88.4 mmol, 1 equiv) and diene **10** (50.3 g, 177 mmol, 2.00 equiv) was heated to 120 °C. After 17 h, the reaction mixture was diluted with a mixture of ethyl acetate-hexanes (2:3, 400 mL) and the resulting suspension was filtered through a plug of Celite®. The filtrate was concentrated and the residue was purified by flash-column chromatography on silica gel (30% ethyl acetate in hexanes initially, grading to 50% ethyl acetate in hexanes). The obtained yellowish solid was dissolved in a minimum amount of hot dichloromethane and precipitated by the addition of hexanes to give **11** (17.2 g, 86%) as a white powder.

TLC (40% ethyl acetate in hexanes): R_f = 0.32 ($KMnO_4$, UV).

^{1}H NMR ($CDCl_3$, 300 MHz): δ = 10.96 (s, 1H), 6.45 (d, *J* = 2.5 Hz, 1H), 6.40 (d, *J* = 2.5 Hz, 1H), 5.73 (s, 1H), 3.87 (s, 6H).

^{13}C NMR ($CDCl_3$, 75 MHz): δ = 169.9, 169.5, 164.1, 161.3, 137.9, 108.4, 105.2, 103.5, 53.2, 53.1.

IR (Diamond-ATR, neat): $\tilde{\upsilon}_{max}$ = 3343, 3202, 2952, 1671, 1615, 1588, 1437, 1238, 1194, 1154, 1024, 849, 729.

HRMS (ESI) calc. for $C_{10}H_9O_6^-$ [M–H]$^-$: 225.0405; found: 225.0406.

Melting point: 125–126 °C.

Dimethyl 3-hydroxy-5-methoxyphthalate 12

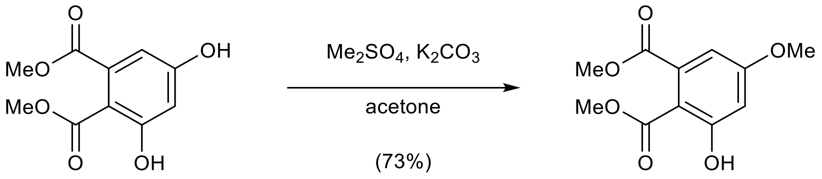

11 **12**

To a solution of phenol **11** (10.0 g, 44.2 mmol, 1 equiv) in acetone (200 mL) were added potassium carbonate (9.17 g, 66.3 mmol, 1.50 equiv) and dimethyl sulfate (5.58 g, 44.2 mmol, 1.00 equiv). After 3.5 h, the reaction mixture was filtered through a plug of Celite® and the filtrate was concentrated. The residue was purified by flash-column chromatography on silica gel (20% ethyl acetate in hexanes) to yield phthalate **12** (7.79 g, 73%) as a colorless solid

TLC (30% ethyl acetate in hexanes): R_f = 0.44 ($KMnO_4$, UV).

^{1}H NMR ($CDCl_3$, 300 MHz): δ = 11.00 (s, 1H), 6.48 (d, *J* = 2.5 Hz, 1H), 6.45 (d, *J* = 2.5 Hz, 1H), 3.85 (s, 3H), 3.85 (s, 3H), 3.80 (s, 3H).

^{13}C NMR ($CDCl_3$, 75 MHz): δ = 169.7, 169.6, 164.8, 164.2, 137.5, 108.1, 103.0, 102.6, 56.1, 53.0, 52.9.

IR (Diamond-ATR, neat): $\tilde{\upsilon}_{max}$ = 2954, 1733, 1615, 1501, 1433, 1264, 1197, 1149, 1022, 759.

HRMS (ESI) calc. for $C_{11}H_{11}O_6^-$ $[M–H]^-$: 239.0561; found: 239.0563.

Melting point: 68–69 °C.

Imide 13

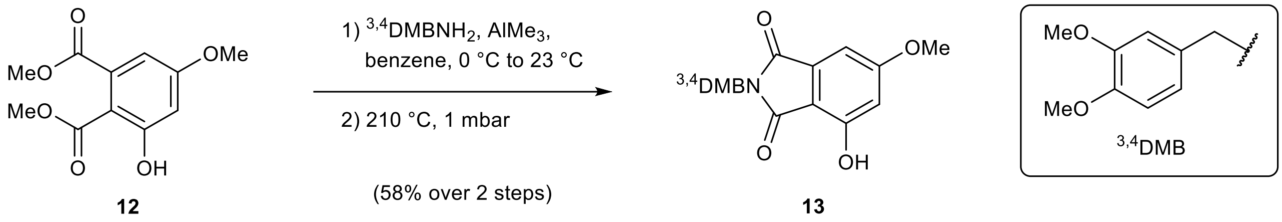

To a solution of 3,4-dimethoxybenzylamine (^{3,4}DMBNH$_2$) (15.8 mL, 104 mmol, 5.00 equiv) in benzene (13 mL) was added trimethylaluminium (2.0 M in toluene, 51.0 mL, 102 mmol, 4.90 equiv) at 0 °C and the solution was allowed to warm to 23 °C. After 40 min, a solution of diester **12** (5.00 g, 20.8 mmol, 1 equiv) in benzene (24 mL) was added to the yellow suspension. The transfer was quantified with benzene (2 × 3 mL). The reaction mixture was heated to 70 °C. After 3 h, the mixture was allowed to cool to 23 °C and ethyl acetate (400 mL) was added. The organic layer was washed with aqueous hydrochloric acid solution (2 M, 2 × 200 mL) and saturated aqueous sodium chloride solution (200 mL). The washed organic extract was dried over sodium sulfate, the dried solution was filtered and the filtrate was concentrated to give the phthalamide (10.6 g) as a yellow solid.

The crude phthalamide (10.6 g) was placed in a bulb-to-bulb distillation apparatus. (Set up: flask **1** was filled with starting material, flask **2** was in heating device, flask **3** as trap at 23 °C, flask **4** was cooled to –78 °C, Supplementary Figure 1). The oven was carefully heated to 210 °C under high vacuum (1 mbar) for 60 min. The dark orange residue remaining in flask **1** was recrystallized from ethanol (40 mL) to yield **13** (5.05 g, 58% over 2 steps) as pale yellow crystalline solid.

TLC (dichloromethane): R_f = 0.25 (UV, $KMnO_4$).

^{1}H NMR ($CDCl_3$, 400 MHz): δ = 7.51 (br s, 1H), 7.00–6.96 (m, 2H), 6.93 (d, *J* = 2.0 Hz, 1H), 6.80 (m, 1H), 6.56 (d, *J* = 2.0 Hz, 1H), 4.70 (s, 2H), 3.87 (s, 3H), 3.86 (s, 3H), 3.84 (s, 3H).

^{13}C NMR ($CDCl_3$, 101 MHz): δ = 169.8, 167.7, 167.0, 156.3, 149.1, 148.8, 134.0, 129.0, 121.3, 112.0, 111.1, 107.7, 105.9, 104.2, 56.4, 56.0, 56.0, 41.4.

IR (Diamond-ATR, neat): $\tilde{v}_{max}$ = 3440, 2939, 1693, 1624, 1516, 1400, 1260, 1156, 1026, 758.

HRMS (EI) calc. for $C_{18}H_{17}NO_6$ 343.1050 $[M]^+$; found: 343.1046.

Melting point: 155–157 °C.

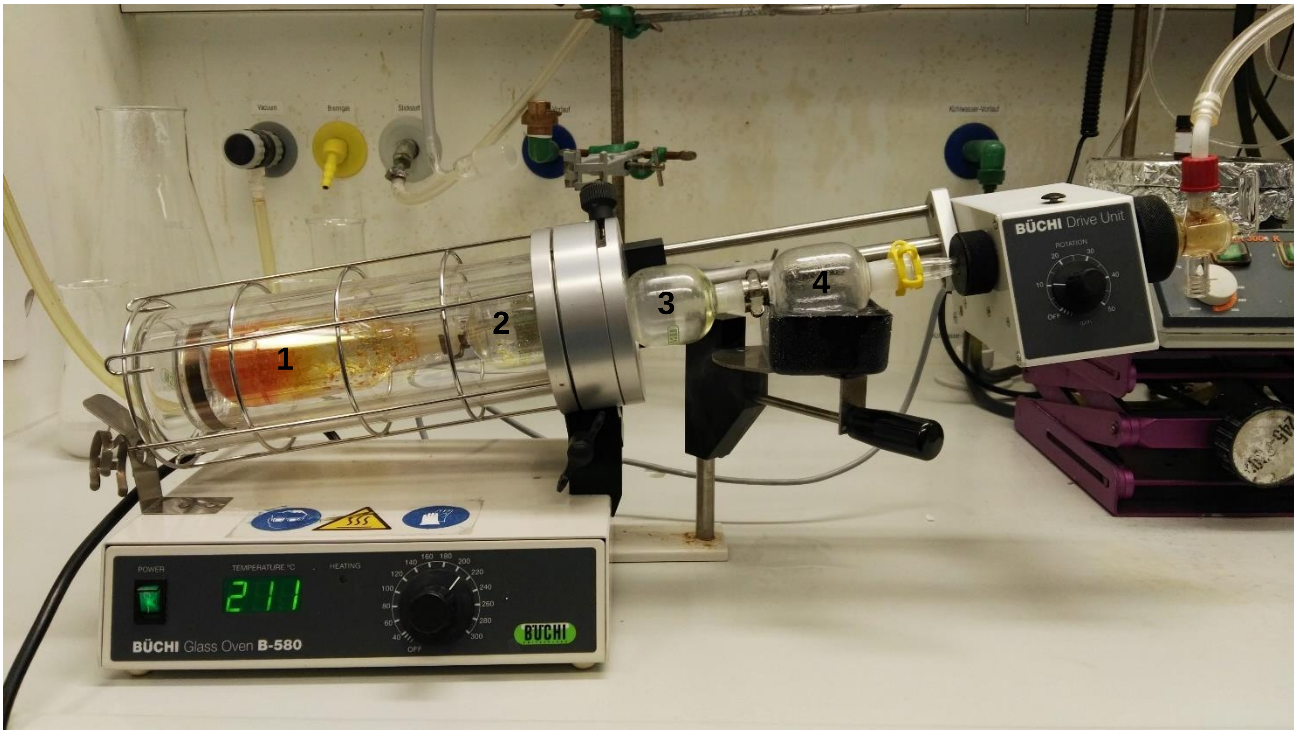

Supplementary Figure 1 Bulb-to-bulb setup: flask **1** was filled with starting material, flask **2** was in heating device, flask **3** as trap at 23 °C, flask **4** was cooled to –78 °C.

Iodide 14

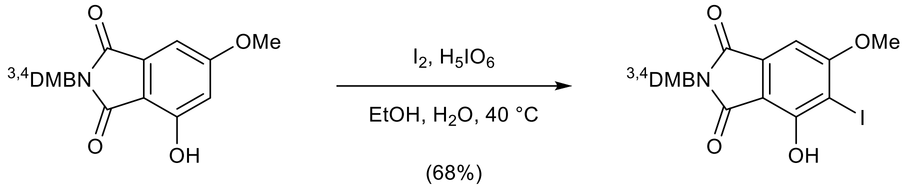

13 **14**

To a suspension of phenol **13** (5.05 g, 14.7 mmol, 1 equiv) and iodine (2.24 g, 8.83 mmol, 0.60 equiv) in ethanol (60 mL) was added a solution of periodic acid (671 mg, 2.94 mmol, 0.20 equiv) in water (3.5 mL) and the reaction mixture was heated to 40 °C. After 30 h, dichloromethane (200 mL) was added, the organic layer was washed with saturated aqueous sodium thiosulfate solution (2 × 150 mL) and saturated sodium chloride solution (150 mL). The washed solution was dried over magnesium sulfate, the dried solution was filtered and the filtrate was concentrated. The residue was purified by flash-column chromatography on silica gel (3% methanol in dichloromethane) to yield **14** (4.70 g, 68%) as an off-white solid.

TLC (4% methanol in dichloromethane): R_f = 0.70 (UV, $KMnO_4$).

^{1}H NMR ($CDCl_3$, 400 MHz): δ = 8.11 (br s, 1H), 7.00–6.94 (m, 2H), 6.89 (s, 1H), 6.80 (d, *J* = 8.0 Hz, 1H), 4.72 (s, 2H), 4.01 (s, 3H), 3.87 (s, 3H), 3.85 (s, 3H),

^{13}C NMR ($CDCl_3$, 101 MHz): δ = 169.5, 167.5, 165.3, 155.5, 149.2, 149.0, 133.9, 128.8, 121.4, 112.1, 111.3, 108.1, 99.3, 82.0, 57.6, 56.1, 56.1, 41.7.

IR (Diamond-ATR, neat): $\tilde{v}_{max}$ = 3413, 2940, 1693, 1620, 1393, 1259, 1158, 1061, 1025, 729.

HRMS (EI) calc. for $C_{18}H_{16}{}^{127}INO_4$ $[M]^+$: 469.0017; found: 469.0009.

Melting point: 214–216 °C.

Isoindolinone S03

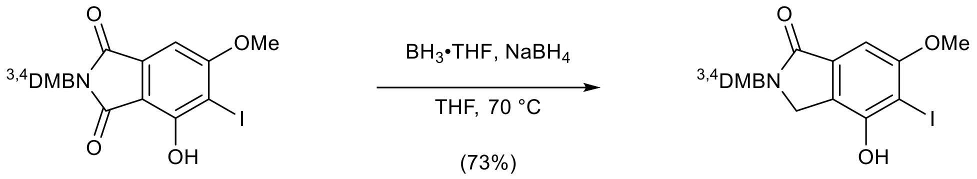

14 **S03**

To a solution of iodide **14** (810 mg, 1.73 mmol, 1 equiv) in tetrahydrofuran (9 mL) was added a solution of borane tetrahydrofuran complex (1.00 M in tetrahydrofuran, 2.16 mL, 2.16 mmol, 3.00 equiv) at 23 °C. After the gas evolution ceased, sodium borohydride (3.27 mg, 0.09 mmol, 0.05 equiv) was added at 23 °C and the mixture was heated to 70 °C in a pressure flask. After 17 h, the reaction mixture was cooled to 23 °C, diluted with aqueous hydrochloric acid solution (1 M, 75 mL) and extracted with ethyl acetate (4 × 75 mL). The combined organic extracts were dried over sodium sulfate, the dried solution was filtered and the filtrate was concentrated. The residue was purified by flash-column chromatography on silica gel (1% methanol in dichloromethane) to give **S03** (573 mg, 73%) as an off-white powder.

TLC (2% methanol in dichloromethane): R_f = 0.23 (UV, $KMnO_4$).

^{1}H NMR ($CDCl_3$, 599 MHz): δ = 6.92 (s, 1H), 6.85–6.77 (m, 3H), 6.22 (s, 1H), 4.69 (s, 2H), 4.21 (s, 2H), 3.94 (s, 3H), 3.84 (s, 3H), 3.82 (s, 3H).

^{13}C NMR ($CDCl_3$, 151 MHz): δ = 168.0, 159.4, 151.0, 149.5, 148.8, 135.3, 129.5, 120.7, 119.7, 111.5, 111.2, 97.9, 82.6, 57.2, 56.1, 56.1, 47.4, 46.6.

IR (Diamond-ATR, neat): $\tilde{v}_{max}$ = 2937, 1649, 1514, 1466, 1350, 1259, 1138, 1082, 1026, 729.

HRMS (EI) calc. for $C_{18}H_{18}{}^{127}INO_5$ $[M]^+$: 455.0224; found: 455.0222.

Melting point: 198–200 °C.

Methoxymethyl ether 15

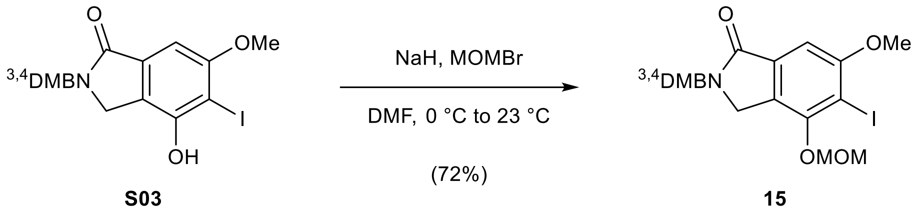

To a solution of amide **S03** (550 mg, 1.21 mmol, 1 equiv) in *N*,*N*-dimethylformamide (24 mL) was added sodium hydride (60% dispersion in mineral oil, 72.5 mg, 1.81 mmol, 1.50 equiv) at 0 °C. After 1 h, bromomethyl methyl ether (130 µL, 1.57 mmol, 1.30 equiv) was added and the reaction mixture was allowed to warm to 23 °C. After 2.5 h, the reaction mixture was diluted with saturated aqueous ammonium chloride solution (40 mL) and ethyl acetate (40 mL). The layers were separated and the aqueous phase was extracted with ethyl acetate (4 × 30 mL). The combined organic extracts were dried over sodium sulfate, the dried solution was filtered and the filtrate was concentrated. The residue was purified by flash-column chromatography on silica gel (50% ethyl acetate in hexanes) to give **15** (435 mg, 72%) as a white crystalline solid.

TLC (2% methanol in dichloromethane): R_f = 0.31 (UV, $KMnO_4$).

^{1}H NMR ($CDCl_3$, 599 MHz): δ = 7.11 (s, 1H), 6.86–6.79 (m, 3H), 5.12 (s, 2H), 4.71 (s, 2H), 4.32 (s, 2H), 3.96 (s, 3H), 3.86 (s, 3H), 3.84 (s, 3H), 3.52 (s, 3H).

^{13}C NMR ($CDCl_3$, 151 MHz): δ = 167.6, 160.2, 153.1, 149.5, 148.8, 135.8, 129.4, 124.5, 120.6, 111.4, 111.3, 101.2, 98.1, 88.4, 57.5, 57.2, 56.1, 56.1, 48.0, 46.5.

IR (Diamond-ATR, neat): $\tilde{\nu}_{max}$ = 2933, 1678, 1609, 1512, 1461, 1257, 1132, 1045, 1023, 762.

HRMS (EI) calc. for $C_{20}H_{22}{}^{127}INO_4$ $[M]^+$: 499.0486; found: 499.0483.

Melting point: 106–108 °C.

(*E*)-2-Methylbut-2-enoyl chloride (S05)

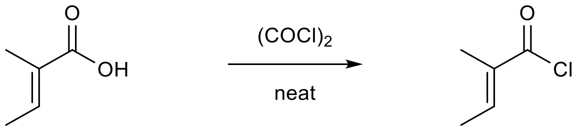

S04 **S05**

(*E*)-2,3-Dimethylacrylic acid (**S04**) (29.3 g, 293 mmol, 1 equiv) was added portionwise to oxalyl chloride (29.5 mL, 304 mmol, 1.30 equiv) followed by one drop of *N*,*N*-dimethylformamide. After 2.5 h, excess oxalyl chloride was removed under reduced pressure to give **S05** (34.7 g, 99%) as a colorless liquid. Acid chloride **S05** was used directly used in the next step without further purification.

Oxazolidinone 21

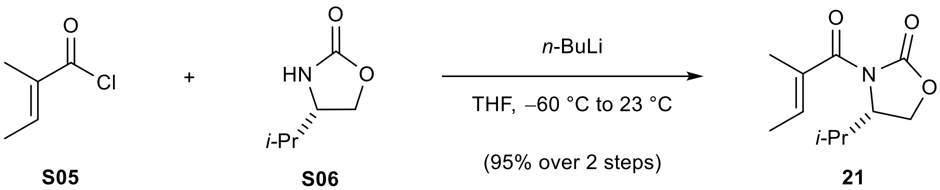

S05 **S06** **21**

A solution of *n*-butyllithium (2.5 M in hexanes, 99.0 mL, 248 mmol, 1.10 equiv) was added dropwise to a solution of **S06** (29.1 g, 225 mmol, 1 equiv) in tetrahydrofuran (600 mL) at −78 °C. After 15 min, **S05** (34.7 g, 293 mmol, 1.30 equiv) was slowly added to the reaction mixture via cannula at −78 °C. After 30 min, the reaction mixture was allowed to warm to 23 °C. After 14 h, aqueous hydrochloric acid (2 M, 1 L) was added to the reaction mixture, the layers were separated and the aqueous layer was extracted with ethyl acetate (3 × 300 mL). The combined organic extracts were dried over sodium sulfate, the dried solution was filtered and the filtrate was concentrated. The residue was purified by flash-column chromatography on silica gel (10% ethyl acetate in hexanes) to yield **21** (45.1 g, 95%) as a yellow solid. The obtained analytical data were in full agreement with those previously reported.[8]

2,2-Dimethyl-1,3-cyclohexadione (16)

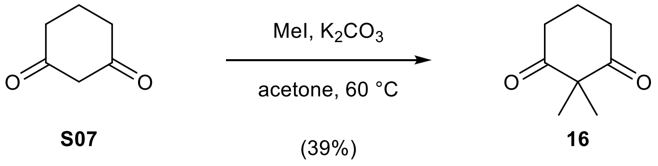

S07 **16**

A suspension of 1,3-cyclohexadione (**S07**) (38.0 g, 340 mmol, 1 equiv), potassium carbonate (93.7 g, 680 mmol, 2.00 equiv) and iodomethane (61.0 mL, 980 mmol, 2.90 equiv) in acetone (300 mL) was heated to 60 °C. After 3 h, excess iodomethane was removed by distillation into a cooling finger (cooled to –78 °C). The residue was diluted with chloroform (300 mL) and water (150 mL). The layers were separated, the aqueous layer was extracted with chloroform (2 × 150 mL) and the combined organic extracts were dried over sodium

sulfate. The dried solution was filtered and the filtrate was concentrated. The residue was purified by flash-column chromatography on silica gel (30% ethyl acetate in hexanes) to yield **16** (18.5 g, 39%) as a yellow oil. The obtained analytical data were in full agreement with those values reported in literature.[9]

Tributyl(vinyl)tin (S09)

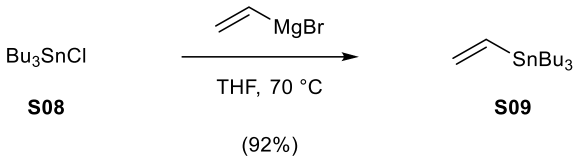

A solution of tributyltin chloride (**S08**) (42.0 mL, 155 mmol, 1 equiv) in tetrahydrofuran (50 mL) was slowly added to a solution of vinylmagnesium bromide (1.0 M in tetrahydrofuran, 310 mL, 310 mmol, 2.00 equiv) via a dropping funnel over a period of 2 h. Upon complete addition, the reaction mixture was heated to 70 °C. After 18 h, the reaction mixture was cooled to 23 °C and saturated aqueous ammonium chloride solution (100 mL) was carefully added. The layers were separated and the aqueous layer was extracted with diethyl ether (2 × 100 mL). The combined organic extracts were concentrated and the residue was purified by vacuum distillation (10 mbar, 128 °C) to yield **S09** (45.1 g, 92%) as a colorless oil. The obtained analytical data were in full agreement with those values reported in literature.[10]

(*R*)-(+)-2-Methyl-CBS-oxazaborilidine (S11)

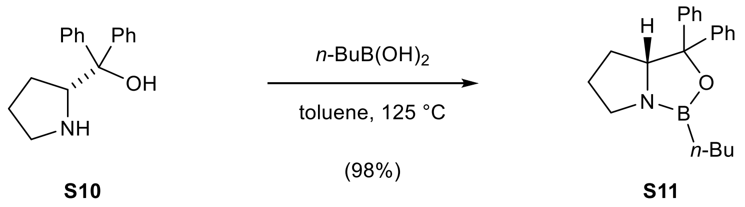

(R)-(+)-2-Methyl-CBS-oxazaborilidine (**S11**) was prepared according to the procedure described by E. J. Grabowski.[11]

A solution of (*R*)-(+)-diphenyl-2-pyrrolidinemethanol (**S10**) (5.25 g, 20.7 mmol, 1 equiv) and butylboronic acid (2.10 g, 20.7 mmol, 1 equiv) in toluene (250 mL) in a two-necked, round-bottomed flask equipped with an additional funnel (containing a cotton plug and 100 g of 4 Å molecular sieves) was heated to 125 °C. After 16 h, the reaction mixture was allowed to cool to 23 °C and the solvent was removed to give **S11** (6.80 g, 98%) as a colorless oil. The obtained analytical data were in full agreement with those values reported in literature.[11]

Alcohol 17

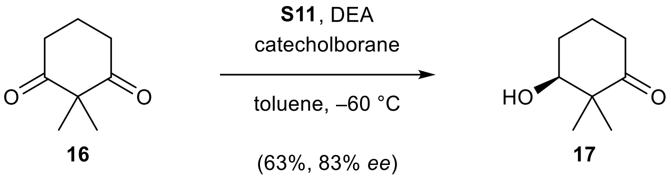

To a solution of (*R*)-(+)-2-methyl-CBS-oxazaborilidine (**S11**) (6.80 g, 22.3 mmol, 0.10 equiv), *N*,*N*-diethylaniline (DEA) (14.8 mL, 92.7 mmol, 0.500 equiv) and 2,2-dimethyl-1,3-cyclohexadione (**16**) (26.0 g, 185 mmol, 1 equiv) in toluene (200 mL) was added a solution of catecholborane (19.8 mL, 185 mmol, 1.00 equiv) in toluene (200 mL) via a dropping funnel over a period of 2.5 h at –60 °C. Upon complete addition, methanol (50 mL) was added and the mixture was diluted with diethyl ether (100 mL). The organic layer was washed with a 1:1 mixture of saturated sodium bicarbonate solution (50 mL) and aqueous sodium hydroxide solution (1 M, 50 mL). The aqueous layer was extracted with ethyl acetate (3 × 100 mL) and the combined organic extracts were washed with saturated aqueous sodium chloride solution (300 mL). The washed solution was dried over magnesium sulfate, the dried solution was filtered and the filtrate was concentrated. The residue was purified by flash-column chromatography on silica gel (20% ethyl acetate in hexanes) to yield **17** (16.7 g, 63%, 83% *ee*) as a yellow oil. The obtained analytical data were in full agreement with those values reported in literature.[12]

Mosher ester S13

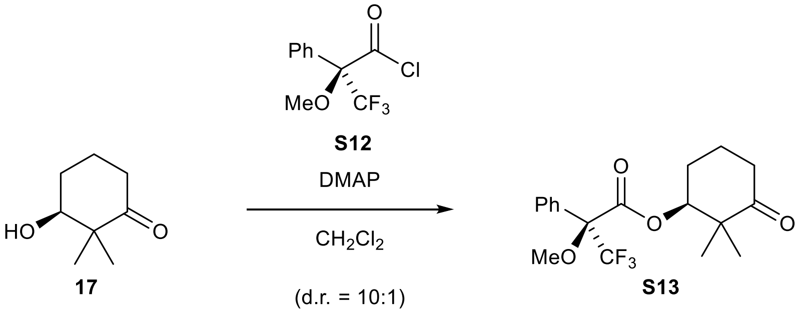

The enantiomeric excess of alcohol **17** was determined as 83% by ^{1}H-NMR analysis of its corresponding-MTPA ester **S13** according to the procedure described by E. J. Corey.[12]

To a solution of alcohol **17** (10.0 mg, 70.0 µmol, 1 equiv) in dichloromethane (1 mL) were added 4-dimethylaminopyridine (34.4 mg, 28.0 µmol, 4.00 equiv) and (*R*)-(−)-α-methoxy-α-(trifluoromethyl)phenylacetyl chloride (**S12**) (20.0 µL, 110 µmol, 1.50 equiv) at 23 °C. After 1 h, water (2 mL) and dichloromethane (3 mL) were added and the layers were separated. The aqueous layer was extracted with dichloromethane (2 × 3 mL) and the combined organic extracts were dried over magnesium sulfate. The dried solution was filtered and the filtrate was concentrated to give **S13** as a yellow oil. The obtained analytical data were in full agreement with those values reported in literature.

Benzyl ether 18

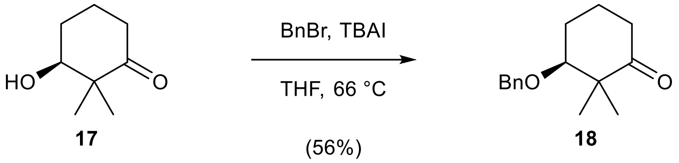

To a solution of alcohol **17** (14.0 g, 98.5 mmol, 1 equiv) in tetrahydrofuran (360 mL) was added sodium hydride (60% mineral oil dispersion, 4.33 g, 108 mmol, 1.10 equiv) at 0 °C. After 30 min, tetrabutylammonium iodide (TBAI) (72.7 g, 197 mmol, 2.00 equiv) and benzyl bromide (29.4 mL, 246 mmol, 2.5 equiv) were added subsequently and the resulting suspension was heated to 66 °C. After 14 h, the reaction mixture was allowed to cool to 23 °C, saturated aqueous ammonium chloride solution (300 mL) and diethyl ether (100 mL) were added. The layers were separated and the aqueous layer was extracted with diethyl ether (2 × 200 mL). The combined organic extracts were washed with saturated aqueous sodium chloride solution (200 mL) and the washed solution was dried over magnesium sulfate. The dried solution was filtered and the filtrate was concentrated. The residue was purified by flash-column chromatography on silica gel (hexanes initially, grading to 5% ethyl acetate in hexanes) to yield **18** (12.7 g, 56%) as a colorless oil.

TLC (5% ethyl acetate in hexanes): R_f = 0.18 (UV, $KMnO_4$).

^{1}H NMR (400 MHz, $CDCl_3$): δ = 7.29–7.25 (m, 4H), 7.23–7.18 (m, 1H), 4.56 (d, *J* = 11.8 Hz, 1H), 4.34 (d, *J* = 11.8 Hz, 1H), 3.37–3.31 (m, 1H), 2.33 (td, *J* = 6.4, 1.9 Hz, 2H), 2.01–1.90 (m, 2H), 1.88–1.78 (m, 1H), 1.61–1.51 (m, 1H), 1.13–1.11 (m, 6H).

^{13}C NMR (101 MHz, $CDCl_3$): δ = 213.9, 138.4, 128.1, 127.3, 127.3, 84.4, 71.1, 50.8, 37.1, 24.3, 23.2, 20.3, 20.3.

IR (Diamond-ATR, neat): $\tilde{\nu}_{max}$ = 2943, 2870, 1706, 1453, 1118, 1090, 1074, 1066, 1028, 737.

HRMS (EI) calc. for $C_{15}H_{20}O_2$ $[M]^+$: 232.1458; found: 232.1463.

$[\boldsymbol{\alpha}]_D^{20}$ = +9.1° (*c* = 6.80, CH_2Cl_2).

Triflate 19

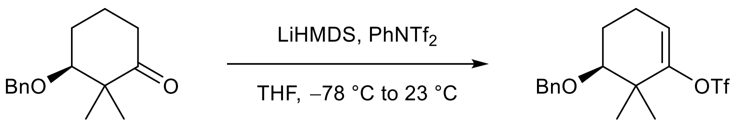

18 (80%) **19**

To a solution of benzyl ether **18** (12.0 g, 51.7 mmol, 1 equiv) in tetrahydrofuran (200 mL) was added lithium bis(trimethylsilyl)amide solution (1.00 M in tetrahydrofuran, 67.1 mL, 67.1 mmol, 1.30 equiv) at –78 °C. After 1 h, *N*-phenylbis(trifluoromethanesulfonimide) (24.0 g, 67.1 mmol, 1.30 equiv) was added portionwise. Upon complete addition, the reaction mixture was allowed to warm to 23 °C. After 2.5 h, saturated aqueous ammonium chloride solution (200 mL) was added and the layers were separated. The aqueous layer was extracted with diethyl ether (3 × 50 mL) and the combined organic extracts were dried over sodium sulfate. The dried solution was filtered and the filtrate was concentrated. The residue was purified by flash-column chromatography on silica gel (2% ethyl acetate in hexanes) to yield **19** (15.1 g, 80%) as a colorless oil.

TLC (5% ethyl acetate in hexanes): R_f = 0.50 (UV, CAM).

^{1}H NMR (400 MHz, $CDCl_3$): δ = 7.42–7.37 (m, 4H), 7.35–7.30 (m, 1H), 5.69 (t, *J* = 4.1 Hz, 1H), 4.71 (d, *J* = 11.8 Hz, 1H), 4.50 (d, *J* = 11.8 Hz, 1H), 3.40 (dd, *J* = 9.0, 2.7 Hz, 1H), 2.38–2.25 (m, 1H), 2.24–2.11 (m, 1H), 2.00–1.89 (m, 1H), 1.84–1.73 (m, 1H), 1.28–1.16 (m, 6H).

^{13}C NMR (101 MHz, $CDCl_3$): δ = 153.9, 138.6, 128.5, 127.7, 127.6, 118.5 (q, *J* = 319.3 Hz), 115.5, 82.0, 71.7, 40.6, 24.7, 21.8, 21.1, 20.7.

IR (Diamond-ATR, neat): $\tilde{\nu}_{max}$ = 2978, 2945, 2874, 1411, 1208, 1143, 1025, 983, 874, 698.

HRMS (EI) calc. for $C_{16}H_{19}F_3O_4{}^{32}S$ $[M]^+$: 364.0951; found: 364.0947.

$[\boldsymbol{a}]_{\boldsymbol{D}}^{\mathbf{20}}$ = +22.1° (*c* = 1.07, CH_2Cl_2).

Diene 20

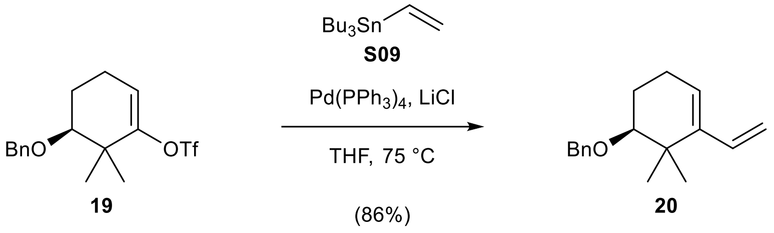

Note: benzene was degassed via freeze-pump-thaw (three cycles) prior to use.

To a mixture of lithium chloride (6.98 g, 165 mmol, 5.00 equiv) and tetrakis(triphenylphosphine)palladium(0) (1.90 g, 1.65 mmol, 5.00 mol%) in degassed tetrahydrofuran (235 mL) was added triflate **19** (13.6 g, 35.0 mmol, 1 equiv) and tributyl(vinyl)tin (**S09**) (20.5 mL, 70.0 mmol, 2.00 equiv) at 23 °C in an Ace® pressure tube. After complete addition, the tube was sealed and the mixture was heated to 75 °C. After 18 h, aqueous ammonia solution (10%, 100 mL) was added to the dark brown reaction mixture and the layers were separated. The aqueous layer was extracted with diethyl ether (3 × 50 mL) and the combined organic extracts were dried over sodium sulfate. The dried solution was filtered and the filtrate was concentrated. The residue was purified by flash-column chromatography on silica gel (hexanes initially, grading to 2% ethyl acetate in hexanes) to yield diene **20** (6.83 g, 86%) as a colorless oil.

TLC (10% ethyl acetate in hexanes): R_f = 0.69 (UV, CAM).

^{1}H NMR (400 MHz, $CDCl_3$): δ = 7.40–7.27 (m, 5H), 6.38–6.20 (m, 1H), 5.81–5.68 (m, 1H), 5.36–5.27 (m, 1H), 5.01–4.91 (m, 1H), 4.71 (d, *J* = 11.7 Hz, 1H), 4.47 (d, *J* = 11.7 Hz, 1H), 3.32–3.23 (m, 1H), 2.29–2.19 (m, 1H), 2.13–2.02 (m, 1H), 1.94–1.84 (m, 1H), 1.77–1.66 (m, 1H), 1.14–1.08 (m, 6H).

^{13}C NMR (101 MHz, $CDCl_3$): δ = 143.9, 139.2, 136.4, 128.2, 127.5, 127.3, 121.6, 113.6, 83.0, 71.3, 38.6, 26.2, 23.8, 22.3, 22.1.

IR (Diamond-ATR, neat): $\tilde{\nu}_{max}$ = 3028, 2965, 2940, 2869, 1454, 1359, 1096, 907, 735, 697.

HRMS (EI) calc. for $C_{17}H_{22}O$ $[M]^+$: 242,1665; found: 242.1677.

$[\alpha]_D^{20}$ = +29.0° (*c* = 1.45, CH_2Cl_2).

Diels-Alder product 22 and S14

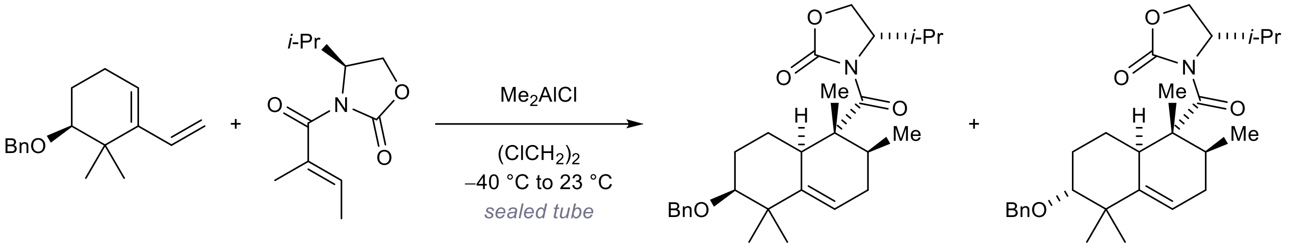

20 **21** **22** (51%) **S14** (4%)

A solution of dimethylaluminium chloride (1.00 M in hexanes, 82.9 mL, 82.9 mmol, 3.00 equiv) was added dropwise to a solution of dienophile **21** (7.59 g, 35.9 mmol, 1.30 equiv) in 1,2-dichloroethane (100 mL) at –40 °C in an oven dried Ace® round-bottom pressure flask (Sigma Aldrich, product number: Z567205) under nitrogen over a period of 30 min. After 30 min, a solution of diene **20** (6.70 g, 27.6 mmol, 1 equiv) in 1,2-dichlorethane (65 mL) was added dropwise over a period of 20 min to the reaction mixture. The transfer was quantitated with 1,2-dichloroethane (2 × 5 mL). After complete addition, the pressure tube was sealed and the reaction mixture was allowed to warm to 23 °C. After 40 h, the reaction mixture was cooled to 0 °C and aqueous hydrochloric acid (2 M, 200 mL) was carefully added. The layers were separated and the aqueous layer was extracted with dichloromethane (3 × 100 mL). The combined organic extracts were dried over magnesium sulfate, the dried solution was filtered and the filtrate was concentrated. The residue was purified by flash-column chromatography on silica gel (4% ethyl acetate in hexanes initially, grading to 10% ethyl acetate in hexanes) to yield the **22** (6.43 g, 51%) as colorless solid and **S14** (483 mg, 4%) as colorless solid. Recrystallization from ethyl acetate gave crystals of both epimers **22** and **S14** suitable for single-crystal X-ray diffraction.

***Major diastereomer* 22:**

TLC (20% ethyl acetate in hexanes): R_f = 0.69 (UV, CAM).

^{1}H NMR (599 MHz, $CDCl_3$): δ = 7.32 (dd, *J* = 3.9, 1.4 Hz, 4H), 7.26–7.23 (m, 1H), 5.56–5.48 (m, 1H), 4.61–4.53 (m, 2H), 4.35 (d, *J* = 12.3 Hz, 1H), 4.29 (t, *J* = 8.7 Hz, 1H), 4.26–4.18 (m, 1H), 3.45 (d, *J* = 12.7 Hz, 1H), 3.22–3.11 (m, 2H), 2.41–2.31 (m, 1H), 2.02–1.93 (m, 1H), 1.91–1.83 (m, 1H), 1.83–1.74 (m, 2H), 1.73–1.65 (m, 1H), 1.15 (s, 3H), 1.08 (s, 3H), 1.07–1.03 (m, 4H), 0.94–0.90 (m, 6H), 0.80 (d, *J* = 6.8 Hz, 3H).

^{13}C NMR (151 MHz, $CDCl_3$): δ = 178.0, 153.1, 142.4, 139.5, 128.3, 127.6, 127.3, 118.2, 83.1, 70.4, 62.9, 61.2, 53.6, 41.2, 36.8, 31.4, 29.8, 29.0, 28.3, 25.8, 23.7, 22.4, 18.6, 16.5, 14.6, 12.4.

IR (Diamond-ATR, neat): $\tilde{\nu}_{max}$ = 2963, 2874, 1777, 1682, 1454, 1383, 1240, 1196, 1117, 747.

HRMS (ESI) calc. for: $C_{28}H_{43}N_2O_4$ 471.3217 $[M+NH_4]^+$; found: 471.3225.

$[\alpha]_D^{20}$ = +46.6° (*c* = 0.56, CH_2Cl_2).

Melting point: 133–141 °C

***Minor diastereomer* S14:**

TLC (20% ethyl acetate in hexanes): R_f = 0.69 (UV, CAM).

^{1}H NMR (800 MHz, $CDCl_3$): δ = 7.36–7.31 (m, 4H), 7.27–7.25 (m, 1H), 5.59 (d, *J* = 6.0 Hz, 1H), 4.63 (d, *J* = 12.1 Hz, 1H), 4.56 (d, *J* = 8.2 Hz, 1H), 4.43 (d, *J* = 12.1 Hz, 1H), 4.32–4.27 (m, 1H), 4.21 (dd, *J* = 9.2, 2.3 Hz, 1H), 3.38–3.32 (m, 1H), 3.21–3.14 (m, 1H), 2.93 (dd, *J* = 11.5, 4.2 Hz, 1H), 2.38–2.31 (m, 1H), 2.02–1.97 (m, 1H), 1.97–1.92 (m, 1H), 1.76–1.70 (m, 1H), 1.54–1.47 (m, 1H), 1.40–1.35 (m, 1H), 1.24–1.20 (m, 1H), 1.18 (s, 3H), 1.06 (s, 3H), 1.01 (s, 3H), 0.93–0.90 (m, 6H), 0.80 (d, *J* = 6.8 Hz, 3H).

^{13}C NMR (201 MHz, $CDCl_3$): δ = 177.8, 153.0, 144.3, 139.5, 128.3, 127.5, 127.4, 117.9, 84.4, 71.6, 62.9, 61.1, 53.3, 42.5, 36.9, 31.2, 29.6, 28.3, 26.3, 26.2, 24.6, 22.5, 18.6, 16.4, 14.6, 12.6.

IR (Diamond-ATR, neat): $\tilde{\nu}_{max}$ = 2963, 2873, 1772, 1679, 1381, 1358, 1192, 1090, 1062, 914, 732.

HRMS (ESI) calc. for: $C_{28}H_{43}N_2O_4$ 471.3217 $[M+NH_4]^+$; found: 471.3226.

$[\alpha]_D^{20}$ = +18.7° (*c* = 1.19, CH_2Cl_2).

Melting point: 126–132 °C

Thioester 23

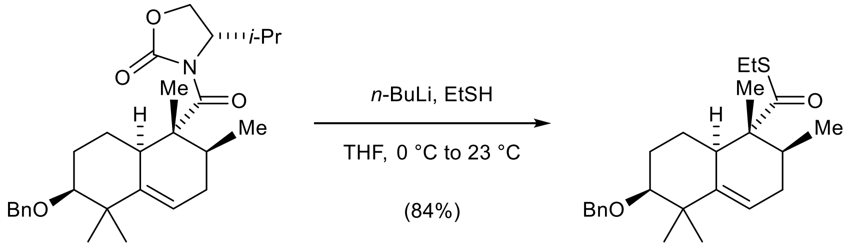

22 **23**

A solution of *n*-butyllithium (2.22 M in hexanes, 27.5 mL, 61.1 mmol, 4.70 equiv) was added dropwise to a solution of ethanethiol (4.82 mL, 65.0 mmol, 5.00 equiv) in tetrahydrofuran (200 mL) at 0 °C. After complete addition, the reaction mixture was allowed to warm to 23 °C. After 30 min, a solution of oxazolidinone **22** (5.90 g, 13.0 mmol, 1 equiv) in tetrahydrofuran (60 mL) was slowly added. The transfer was quantitated with tetrahdydrofuran (2 × 5 mL). After 21 h, saturated aqueous ammonium chloride solution (200 mL) was added, the layers were separated and the aqueous layer was extracted with diethyl ether (3 × 100 mL). The combined organic extracts were dried over sodium sulfate, the dried solution was filtered and the filtrate was concentrated. The residue was purified by flash-column chromatography on silica gel (5% ethyl acetate in hexanes initially, grading to 10% ethyl acetate in hexanes) to yield **23** (4.20 g, 84%) as a colorless oil.

TLC (9% ethyl acetate in hexanes): R_f = 0.32 (UV, CAM).

^{1}H NMR (599 MHz, $CDCl_3$): δ = 7.34–7.30 (m, 4H), 7.28–7.24 (m, 1H), 5.51–5.45 (m, 1H), 4.57 (d, *J* = 12.1 Hz, 1H), 4.34 (d, *J* = 12.1 Hz, 1H), 3.17–3.13 (m, 1H), 2.92–2.82 (m, 3H), 2.05–1.91 (m, 2H), 1.89–1.76 (m, 2H), 1.74–1.63 (m, 2H), 1.29–1.22 (m, 4H), 1.16 (s, 3H), 1.08–1.02 (m, 6H), 0.81 (d, *J* = 6.7 Hz, 3H).

^{13}C NMR (151 MHz, $CDCl_3$): δ = 208.2, 142.7, 139.4, 128.3, 127.6, 127.3, 117.9, 82.8, 70.3, 56.8, 42.9, 40.9, 36.9, 31.6, 29.4, 25.9, 23.2, 23.2, 21.0, 15.9, 14.9, 9.6.

IR (Diamond-ATR, neat): $\tilde{\nu}_{max}$ = 2963, 2929, 2872, 1669, 1453, 1383, 1113, 950, 909, 731.

HRMS (ESI) calc. for $C_{24}H_{38}NO_2S^+$ $[M+NH_4^+]^+$: 404.2618; found: 404.2607.

$[\alpha]_D^{20}$ = +3.22° (*c* = 3.29, CH_2Cl_2).

Alcohol 24

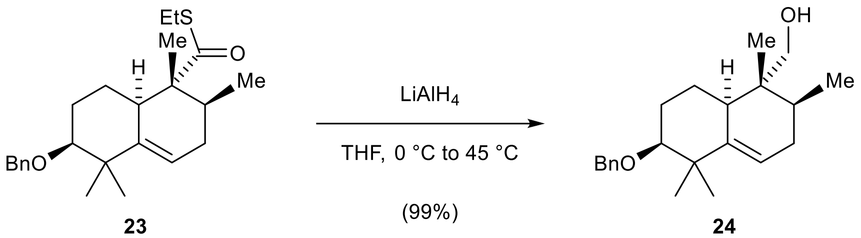

23 **24**

Lithium aluminium hydride (1.96 g, 51.7 mmol, 5.00 equiv) was added portionwise to a solution of thioester **23** (4.00 g, 10.3 mmol, 1 equiv) at 0 °C. After complete addition, the reaction mixture was heated to 45 °C. After 3 h, the reaction mixture was cooled to 0 °C, diluted with diethyl ether (100 mL) and saturated aqueous potassium sodium tartrate solution (400 mL) was carefully added. After stirring vigorously for 1 h, the layers were separated and the aqueous layer was extracted with diethyl ether (3 × 150 mL). The combined organic extracts were dried over magnesium sulfate, the dried solution was filtered and the filtrate was concentrated. The residue was purified by flash-column chromatography on silica gel (20% ethyl acetate in hexanes) to yield **24** (3.40 g, 99%) as a colorless oil.

TLC (15% ethyl acetate in hexanes): R_f = 0.15 (CAM).

^{1}H NMR (599 MHz, $CDCl_3$): δ = 7.34–7.30 (m, 4H), 7.26–7.23 (m, 1H), 5.52–5.49 (m, 1H), 4.59 (d, *J* = 12.5 Hz, 1H), 4.36 (d, *J* = 12.4 Hz, 1H), 3.55–3.50 (m, 1H), 3.49–3.41 (m, 1H), 3.18–3.13 (m, 1H), 2.51–2.42 (m, 1H), 1.93–1.81 (m, 3H), 1.76–1.67 (m, 2H), 1.56–1.49 (m, 2H), 1.23 (s, 1H), 1.15 (s, 3H), 1.03 (s, 3H), 0.89 (d, *J* = 6.8 Hz, 3H), 0.57 (s, 3H).

^{13}C NMR (151 MHz, $CDCl_3$): δ = 143.6, 139.6, 128.3, 127.7, 127.3, 118.3, 83.0, 70.3, 65.8, 41.1, 39.5, 37.4, 32.0, 31.6, 29.1, 26.0, 23.7, 20.5, 15.1, 11.5.

IR (Diamond-ATR, neat): $\tilde{\nu}_{max}$ = 3359, 2952, 2873, 1453, 1380, 1102, 1061, 1039, 733, 696.

HRMS (EI) calc. for $C_{22}H_{32}O_2$ $[M]^+$: 328.2397; found: 328.2403.

$[\alpha]_D^{20}$ = +50.9° (c = 0.89, CH_2Cl_2).

Iodide 25

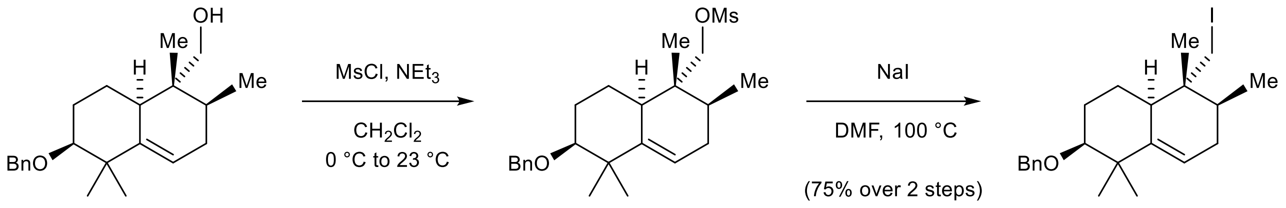

24 S15 25

Methanesulfonyl chloride (1.63 mL, 21.0 mmol, 3.00 equiv) was added to a solution of alcohol **24** (2.30 g, 7.00 mmol, 1 equiv) and triethylamine (5.84 mL, 42.0 mmol, 6.00 equiv) in dichloromethane (45 mL) at 0 °C. After 30 min, the orange, turbid reaction mixture was allowed to warm to 23 °C. After 3 h, dichloromethane (50 mL) and pH 7 phosphate buffer (100 mL) were added. The layers were separated and the aqueous layer was extracted with dichloromethane (2 × 60 mL). The combined organic extracts were dried over sodium sulfate. The dried solution was filtered and the filtrate was concentrated to give mesylate **S15** as an orange oil. The so-obtained mesylate was directly used in the following reaction without further purification.

To a solution of crude mesylate **S15** (2.85 g, 7.00 mmol, 1 equiv) in *N,N*-dimethylformamide (60 mL) was added sodium iodide (8.39 g, 56.0 mmol, 8.00 equiv) and the resulting orange suspension was heated to 100 °C. After 39 h, the reaction mixture was cooled to 23 °C and saturated, aqueous sodium thiosulfate solution (200 mL) and diethyl ether (200 mL) were added. The layers were separated, the aqueous layer was extracted with diethyl ether (3 × 100 mL) and the combined organic extracts were dried over sodium sulfate. The dried solution was filtered and filtrate was concentrated. The residue was purified by flash-column chromatography on silica gel (pentane initially, grading to 40% diethyl ether in pentane) to yield iodide **25** (2.30 g, 75% over 2 steps) as a brown oil and unreacted starting alcohol **24** (379 mg, 17%) as a colorless oil.

TLC (5% ethyl acetate in hexanes): R_f = 0.33 (CAM).

^{1}H NMR (800 MHz, $CDCl_3$): δ = 7.36–7.32 (m, 4H), 7.28–7.25 (m, 1H), 5.52–5.47 (m, 1H), 4.61 (d, *J* = 12.3 Hz, 1H), 4.38 (d, *J* = 12.3 Hz, 1H), 3.45 (d, *J* = 10.3 Hz, 1H), 3.23 (d, *J* = 10.3 Hz, 1H), 3.19–3.17 (m, 1H), 2.46–2.39 (m, 1H), 1.95–1.88 (m, 2H), 1.79–1.72 (m, 2H), 1.63–1.57 (m, 1H), 1.55–1.50 (m, 1H), 1.40–1.36 (m, 1H), 1.17 (s, 3H), 1.06 (s, 3H), 0.87 (s, 3H), 0.83 (d, *J* = 6.7 Hz, 3H).

^{13}C NMR (201 MHz, $CDCl_3$): δ = 143.2, 139.5, 128.3, 127.6, 127.3, 118.1, 82.9, 70.3, 41.1, 41.0, 37.4, 35.2, 31.6, 29.3, 25.9, 23.4, 22.2, 19.9, 14.6, 12.4.

IR (Diamond-ATR, neat): $\tilde{\nu}_{max}$ = 2960, 2871, 1453, 1380, 1231, 1182, 1110, 907, 732.

HRMS (EI) calc. for $C_{22}H_{31}{}^{127}IO$ $[M]^+$: 438.1414; found: 438.1419.

$[\alpha]_D^{20}$ = +6.25° (*c* = 0.80, CH_2Cl_2).

Cyclization precursor 26

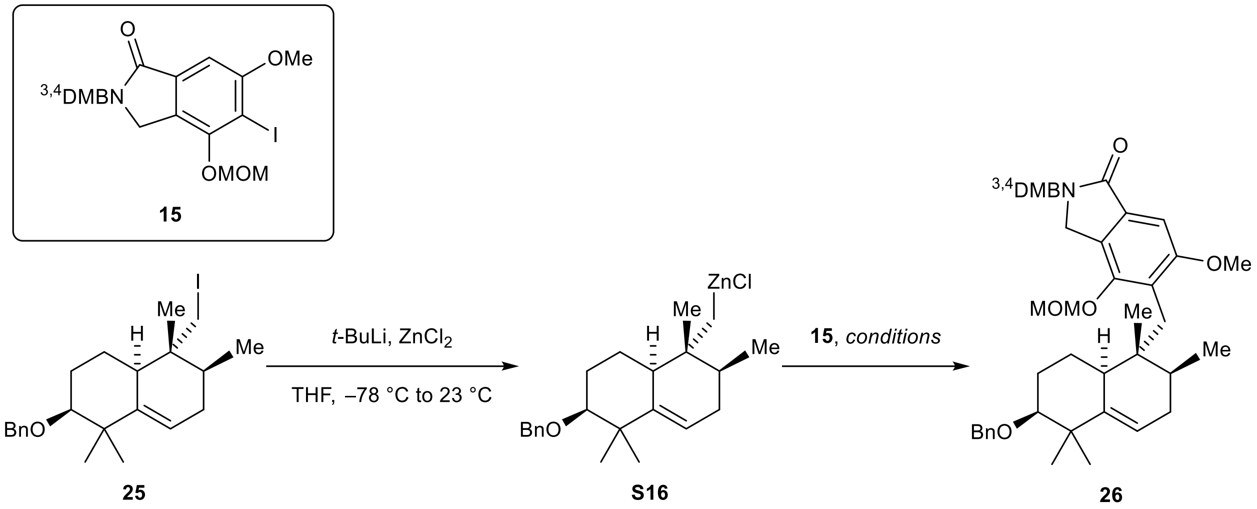

Supplementary Table 1 Conditions investigated for the sp^2–sp^3 Negishi cross coupling. n.r.: no reaction; [1] isolated yield of analytically pure product, [2] 60 µmol (**15**) scale, [3] 870 µmol (**15**) scale

Entry	Catalyst (20 mol%)	Additive	Solvent	Temperature	Yield
1	Pd(dba)$_2$, SPhos	-	THF	23 °C to 60 °C	n.r.
2	Pd(dba)$_2$, SPhos	DMA (25vol%)	THF	23 °C to 60 °C	n.r.
3	Pd(PPh$_3$)$_4$	-	THF	23 °C to 60 °C	n.r.
4	Pd(PPh$_3$)$_4$	DMA (25vol%)	THF	23 °C to 60 °C	n.r.
5	Pd-SPhos-G2, SPhos	-	THF	60 °C	n.r.
6	Pd-SPhos-G2, SPhos	DMA (25vol%)	THF	60 °C	37%[1]
7	Pd-SPhos-G2, SPhos	-	THF	23 °C	n.r.
8	Pd-SPhos-G2, SPhos	DMA (25vol%)	THF	40 °C	61%[1,2]
9	Pd-SPhos-G2, SPhos	DMA (25vol%)	THF	40 °C	56%[1,3]

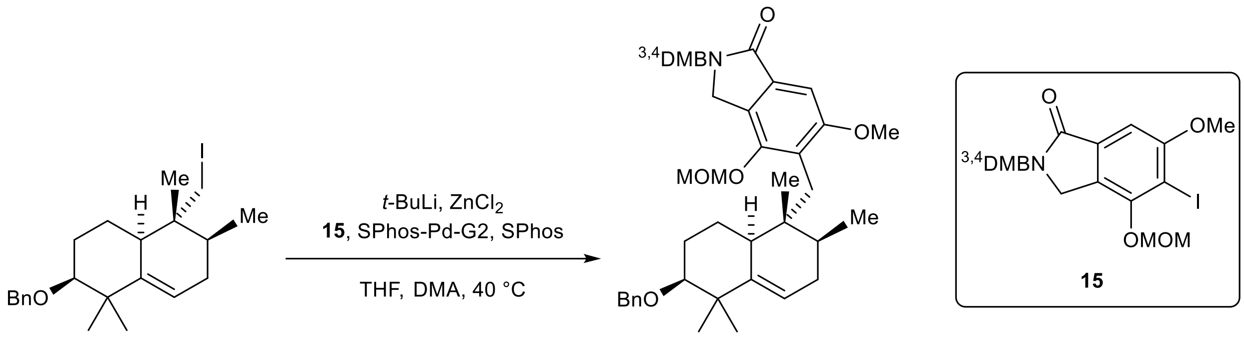

Note: Tetrahydrofuran was dried according to the procedure described by B. Williams prior to use.[13]

To a mixture of alkyl iodide **25** (554 mg, 1.26 mmol, 1.45 equiv) and a solution of zinc chloride (1.00 M in tetrahydrofuran, 1.39 mL, 1.39 mmol, 1.60 equiv) in tetrahydrofuran (1.2 mL) was added dropwise a solution of *tert*-butyllithium (1.50 M in pentane, 1.80 mL, 2.70 mmol, 3.10 equiv) at –78 °C. After 50 min, the mixture was allowed to warm to 23 °C. After 20 min, the mixture was added to a mixture of aryl iodide **15** (435 mg, 870 µmol, 1 equiv), SPhos (71.3 mg, 174 µmol, 0.20 equiv) and SPhos-Pd-G2 (125 mg, 174 µmol, 0.20 equiv) in tetrahydrofuran (2.2 mL) and freshly distilled (over CaH_2) *N*,*N*-dimethylacetamide (2.2 mL) and the reaction mixture was directly placed in a preheated oil bath at 40 °C. After 7 h, the reaction mixture was allowed to cool to 23 °C, and ethyl acetate (50 mL) and saturated aqueous ammonium chloride solution (60 mL) were added. The layers were separated, the aqueous layer was extracted with ethyl acetate (2 × 60 mL) and the combined organic extracts were washed with saturated aqueous sodium chloride solution (70 mL). The washed solution was dried over magnesium sulfate, the dried solution was filtered and the filtrate was concentrated. The residue was purified by flash-column chromatography on silica gel (20% ethyl acetate in benzene) to yield **26** (332 mg, 56%) as a yellow foam.

TLC (50% ethyl acetate in hexanes): R_f = 0.14 (UV, CAM).

^{1}H NMR (599 MHz, $CDCl_3$): δ = 7.30–7.26 (m, 4H), 7.24–7.21 (m, 1H), 7.15 (s, 1H), 6.88–6.86 (m, 2H), 6.84–6.82 (m, 1H), 5.46–5.41 (m, 1H), 4.99 (s, 2H), 4.76–4.69 (m, 2H), 4.48 (d, *J* = 12.5 Hz, 1H), 4.36–4.27 (m, 2H), 4.25 (d, *J* = 12.5 Hz, 1H), 3.88 (s, 3H), 3.87 (s, 3H), 3.86 (s, 3H), 3.49 (s, 3H), 3.03–3.01 (m, 1H), 2.92 (d, *J* = 12.9 Hz, 1H), 2.66 (d, *J* = 12.9 Hz, 1H), 2.59–2.55 (m 1H), 1.91–1.85 (m, 2H), 1.61–1.56 (m, 1H), 1.55–1.51 (m, 1H), 1.44–1.35 (m, 1H), 1.22–1.14 (m, 1H), 1.07 (s, 3H), 1.01 (d, *J* = 6.7 Hz, 3H), 0.96 (s, 3H), 0.85–0.78 (m, 1H), 0.76 (s, 3H).

^{13}C NMR (151 MHz, $CDCl_3$): δ = 168.4, 160.1, 152.4, 149.4, 148.7, 144.5, 139.4, 132.6, 129.9, 128.2, 127.8, 127.3, 126.6, 123.8, 120.7, 117.5, 111.6, 111.3, 100.5, 97.6, 82.7, 70.2, 57.1, 56.1, 56.1, 55.9, 48.6, 46.5, 42.0, 41.4, 41.3, 38.9, 35.4, 32.0, 28.5, 25.8, 24.5, 23.8, 16.5, 14.4.

IR (Diamond-ATR, neat): $\tilde{\nu}_{max}$ = 2933, 1686, 1514, 1464, 1259, 1259, 1237, 1152, 1055, 735.

HRMS (EI) calc. for $C_{42}H_{53}NO_7$ $[M]^+$: 683.3817; found: 683.3800.

$[\boldsymbol{a}]_D^{20}$ = +22.4° (*c* = 0.19, CH_2Cl_2).

Pentacycle S17 and S18

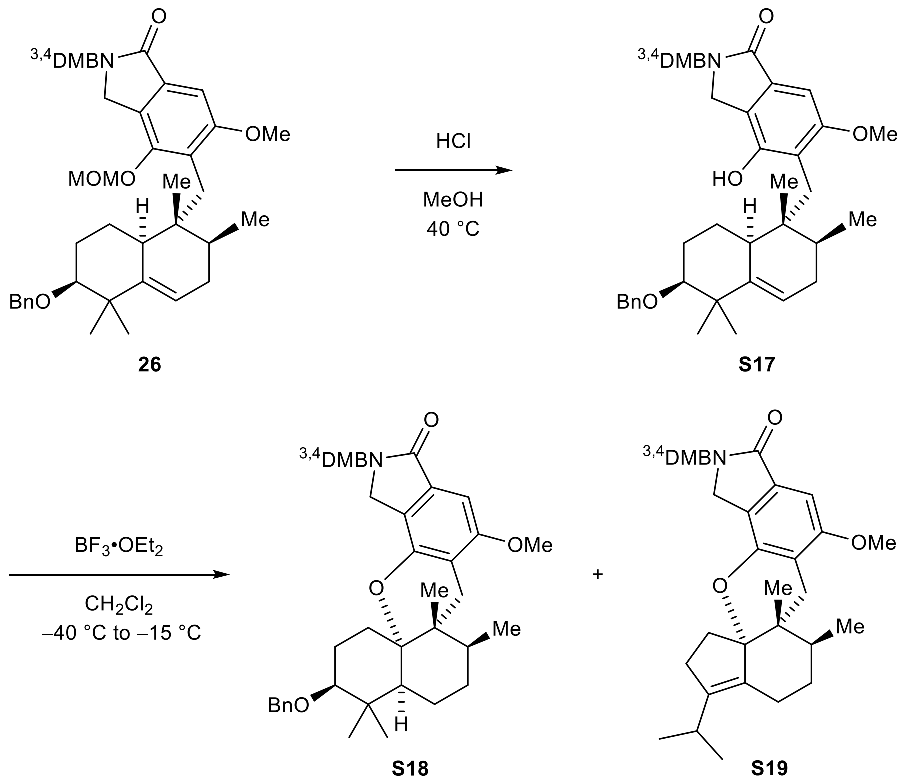

A solution of hydrochloric acid (~1.25 M in methanol, 10 mL) was added to a solution of **26** (290 mg, 424 µmol, 1 equiv) in dichloromethane (5 mL) and the reaction mixture was heated to 40 °C. After 1 h, the reaction mixture was diluted with dichloromethane (30 mL) and saturated aqueous sodium bicarbonate solution (70 mL) was added. The layers were separated, the aqueous layer was extracted with dichloromethane (3 × 60 mL) and the combined organic extracts were dried over magnesium sulfate. The dried solution was filtered and the filtrate was concentrated to yield **S17** as a yellow foam that was directly used in the following step without further purification.

A solution of boron trifluoride diethyl etherate (48% in diethyl ether, 1.11 mL, 4.24 mmol, 10.0 equiv) was added dropwise to a solution of the crude phenol **S17** (271 mg, 424 µmol, 1 equiv) in dichloromethane (40 mL) at –40 °C and the reaction mixture was allowed to slowly warm to –15 °C over a period of 1 h. After 5 h, saturated aqueous sodium bicarbonate solution (50 mL) was added. The layers were separated and the aqueous layer was extracted with dichloromethane (3 × 50 mL). The combined organic extracts were dried over magnesium sulfate, the dried solution was filtered and the filtrate was concentrated. The residue was filtered through a short plug of silica to yield a mixture of **S18** (83%) and rearranged **S19** (17%) as a colorless foam that was used in the following step without further purification.

Analytically pure samples of **S18** and **S19** were obtained by normal-phase semi-preparative HPLC purification using 5% *i*-propanol in *n*-heptane as eluent (flow rate: 15 mL/min; column: Microsorb 60-8 Si Dynamax 250 × 21.4mm (R00083121C); detection: 254 nm; retention times: 21.9 min for **S18** and 23.3 min for **S19**) to give **S18** as colorless foam and **S19** as colorless solid. Recrystallization of **S19** from diethyl ether gave crystals suitable for single-crystal X-ray diffraction.

For S18:

TLC (50% ethyl acetate in benzene): R_f = 0.30 (UV, CAM).

^{1}H NMR (400 MHz, $CDCl_3$): δ = 7.36–7.32 (m, 4H), 7.30–7.26 (m, 1H), 6.91 (s, 1H), 6.86–6.79 (m, 3H), 4.95 (d, J = 14.8 Hz, 1H), 4.62 (d, J = 12.1 Hz, 1H), 4.46 (d, J = 14.8 Hz, 1H), 4.36 (d, J = 12.1 Hz, 1H), 4.21–4.08 (m, 2H), 3.88 (s, 3H), 3.87 (s, 3H), 3.84 (s, 3H), 3.23–3.07 (m, 2H), 2.27–2.10 (m, 4H), 2.06–1.99 (m, 1H), 1.85–1.69 (m, 3H), 1.66–1.58 (m, 1H), 1.56–1.50 (m, 1H), 1.32 (d, J = 13.5 Hz, 1H), 1.10 (d, J = 7.4 Hz, 3H), 1.00–0.94 (m, 6H), 0.86 (s, 3H).

^{13}C NMR (101 MHz, $CDCl_3$): δ = 169.2, 158.6, 149.4, 148.6, 147.3, 139.7, 131.7, 130.2, 128.3, 127.2, 127.2, 121.0, 120.4, 114.0, 111.3, 111.1, 95.9, 84.1, 82.4, 71.7, 56.1, 56.1, 55.9, 47.6, 46.4, 45.0, 39.8, 38.6, 37.7, 32.3, 30.7, 28.0, 27.1, 24.4, 23.8, 21.0, 20.4, 17.3.

IR (Diamond-ATR, neat): $\tilde{\nu}_{max}$ = 2958, 1688, 1606, 1515, 1464, 1368, 1263, 1121, 1084, 737.

HRMS (EI) calc. for $C_{40}H_{49}NO_6$ $[M]^+$: 639.3554; found: 639.3557.

$[\boldsymbol{a}]_D^{20}$ = +90.1° (c = 0.69, CH_2Cl_2).

For S19:

TLC (50% ethyl acetate in benzene): R_f = 0.30 (UV, CAM).

^{1}H NMR (400 MHz, $CDCl_3$): δ = 6.91 (s, 1H), 6.87–6.84 (m, 2H), 6.81 (d, J = 8.4 Hz, 1H), 4.69 (s, 2H), 4.10 (s, 2H), 3.88 (s, 3H), 3.86 (s, 3H), 3.85 (s, 3H), 2.96 (d, J = 18.1 Hz, 1H), 2.83–2.72 (m, 1H), 2.46–2.38 (m, 1H), 2.37–2.31 (m, 1H), 2.28 (d, J = 18.1 Hz, 1H), 2.23–2.09 (m, 2H), 2.08–2.00 (m, 1H), 1.79–1.69 (m, 1H), 1.66–1.61 (m, 1H), 1.47 (d, J = 12.6 Hz, 1H), 1.30–1.25 (m, 1H), 1.03 (d, J = 6.8 Hz, 3H), 1.00 (d, J = 6.8 Hz, 3H), 0.78 (s, 3H), 0.75 (d, J = 6.8 Hz, 3H).

^{13}C NMR (101 MHz, $CDCl_3$): δ = 169.2, 158.6, 149.3, 148.9, 148.6, 143.4, 131.9, 131.6, 130.3, 121.8, 120.8, 113.6, 111.6, 111.0, 96.1, 95.2, 56.1, 56.0, 56.0, 47.4, 46.5, 37.7, 32.0, 31.6, 30.5, 29.3, 28.0, 27.1, 22.4, 22.2, 20.7, 16.0, 15.8.

IR (Diamond-ATR, neat): $\tilde{\nu}_{max}$ = 2930, 1680, 1602, 1466, 1365, 1258, 1144, 1106, 1024, 764.

HRMS (EI) calc. for $C_{33}H_{41}NO_5$ $[M]^+$: 531.2979; found: 531.2976.

$[\boldsymbol{a}]_D^{20}$ = –54.3° (c = 0.47, CH_2Cl_2).

Melting point: 175 °C

Neopentylic alcohol 30

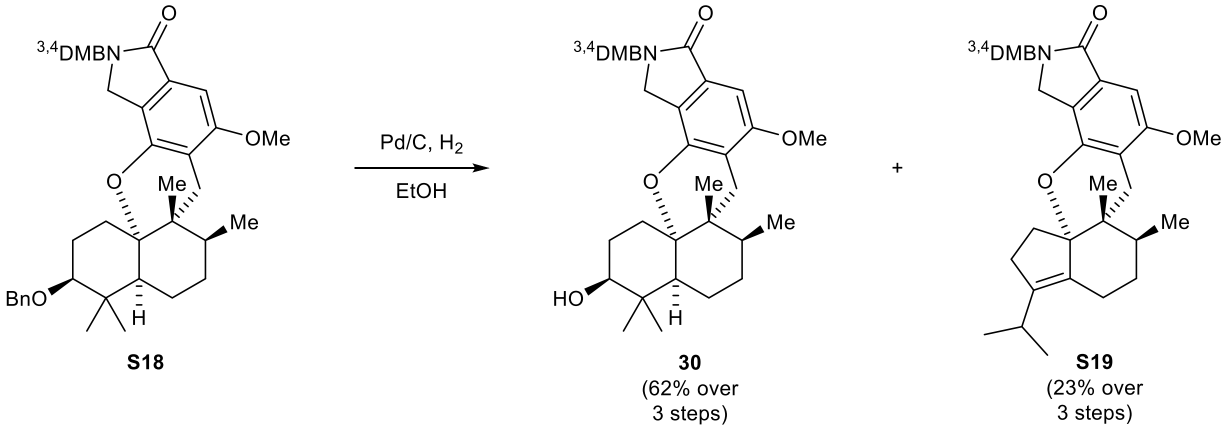

A crude mixture of **S18** and **S19** (424 µmol, 1 equiv) in ethanol (20 mL) was treated with palladium on carbon (10 wt.%, 451 mg, 424 µmol, 1.00 equiv) at 23 °C. An atmosphere of hydrogen was maintained by sparging with a stream of pure hydrogen gas through a stainless steel needle for 2 min and vigorous stirring of the suspension was then continued under hydrogen atmosphere at 23 °C. After 14 h, the reaction mixture was filtered through a pad of Celite® and the filter cake was thoroughly rinsed with dichloromethane (70 mL). The filtrate was concentrated and the residue was purified by flash-column chromatography on silica gel (20% ethyl acetate in benzene initially, grading to 50% ethyl acetate in benzene) to give alcohol **30** (145 mg, 62% over 3 steps) as a colorless foam and **S19** (59 mg, 26% over 2 steps) as a colorless foam. The obtained analytical data for **30** were in full agreement with those values previously reported.[14]

TLC (benzene with 50% EtOAc): R_f = 0.32 (UV, CAM).

^{1}H NMR (400 MHz, $CDCl_3$): δ = 6.91 (s, 1H), 6.87–6.79 (m, 3H), 4.94 (d, *J* = 14.8 Hz, 1H), 4.47 (d, *J* = 14.8 Hz, 1H), 4.21–4.07 (m, 2H), 3.88 (s, 3H), 3.86 (s, 3H), 3.83 (s, 3H), 3.52 (s, 1H), 3.14 (d, *J* = 18.2 Hz, 1H), 2.48–2.35 (m, 1H), 2.30–2.21 (m, 1H), 2.22–2.16 (m, 1H), 2.12–1.97 (m, 2H), 1.81–1.71 (m, 2H), 1.69–1.59 (m, 2H), 1.59–1.57 (m, 1H), 1.57–1.52 (m, 1H), 1.33 (d, *J* = 12.2 Hz, 1H), 1.12 (d, *J* = 7.5 Hz, 3H), 0.96 (s, 3H), 0.95 (s, 3H), 0.90–0.85 (m, 3H).

^{13}C NMR (101 MHz, $CDCl_3$): δ = 169.1, 158.4, 149.3, 148.5, 147.1, 131.7, 130.0, 120.8, 120.3, 113.8, 111.2, 111.1, 95.9, 83.8, 74.6, 56.0, 55.9, 55.8, 47.5, 46.3, 44.7, 39.5, 37.9, 37.6, 32.2, 30.3, 28.1, 26.3, 25.9, 24.1, 23.7, 20.2, 17.1.

IR (Diamond-ATR, neat): $\tilde{\nu}_{max}$ = 3447, 2935, 2871, 1673, 1606, 1515, 1463, 1260, 1120, 974.

HRMS (EI) calc. for $C_{33}H_{43}NO_6$ $[M]^+$: 549,3085; found: 549.3093.

$[\boldsymbol{\alpha}]_{\boldsymbol{D}}^{\mathbf{20}}$ = +106.0° (*c* = 0.20, $CHCl_3$); lit. $[\boldsymbol{\alpha}]_{\boldsymbol{D}}^{\mathbf{28}}$ = +71.7° (c = 2.82, $CHCl_3$)[14]

_O_-Methyl-stachyflin (S20)

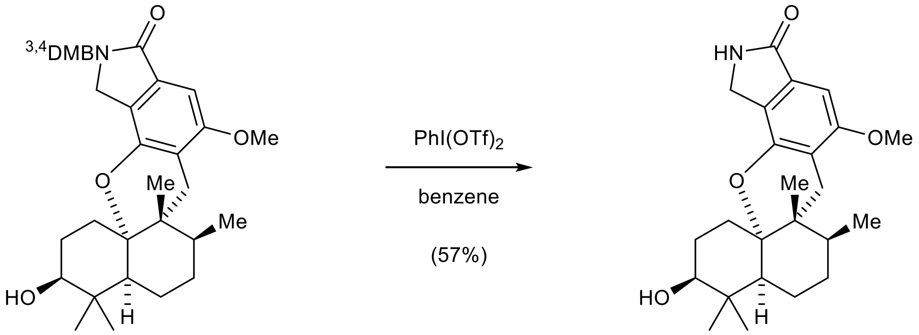

30 **S20**

Phenyliodine(III) bis(trifluoroacetate) (PIFA) (1.19 g, 2.77 mmol. 16.0 equiv) was added in small portions (1 equiv every 30 min) to a solution of **30** (95.0 mg, 173 µmol, 1 equiv) in benzene (24 mL). After 10 h, saturated aqueous sodium thiosulfate solution was added (60 mL) and the aqueous layer was extracted with ethyl acetate (3 × 50 mL). The combined organic extracts were washed with saturated aqueous sodium chloride solution. The washed solution was dried over magnesium sulfate, the dried solution was filtered and the filtrate was concentrated. The residue was purified by flash-column chromatography on silica gel (1% methanol in dichloromethane initially, grading to 5% methanol in dichloromethane) to give **S20** (39.0 mg, 57%) as a colorless solid. The obtained analytical data for **30** were in full agreement with those previously reported.[14]

(+)-Stachyflin (1)

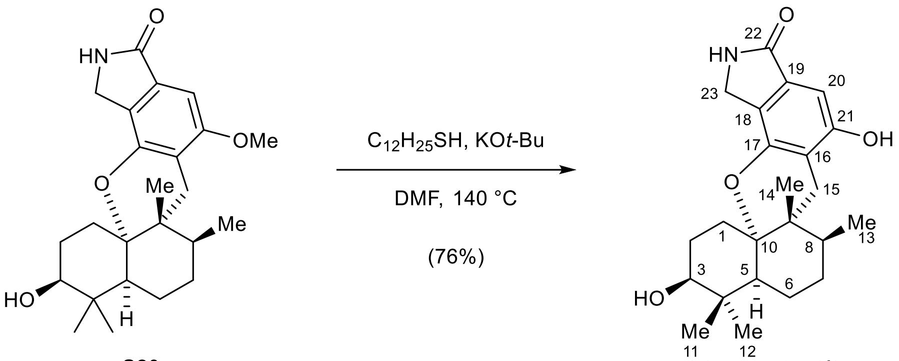

S20 (+)-stachyflin (**1**)

Potassium *tert*-butoxide (25.3 mg, 225 µmol, 3.00 equiv) was added to a solution of *O*-methyl-stachyflin (**S20**) (30.0 mg, 75.1 µmol, 1 equiv) and 1-dodecanethiol (71.9 µL, 30.0 µmol, 4.00 equiv) in *N*,*N*-dimethylformamide (3 mL) at 23 °C. After 5 min, the bright yellow solution was heated to 140 °C. After 75 min, the dark orange reaction mixture was allowed to cool to 23 °C and saturated aqueous ammonium chloride solution (50 mL) was added. The mixture was extracted with ethyl acetate (4 × 40 mL). The combined organic extracts were washed with saturated aqueous sodium chloride solution (2 × 40 mL). The washed solution was dried over magnesium sulfate, the dried solution was filtered and the filtrate was concentrated. The residue was purified by flash-column chromatography on silica gel (1% methanol in dichloromethane initially, grading to 5% methanol in dichloromethane) to yield (+)-stachyflin (**1**) (22.1 mg, 76%) as a colorless solid.

TLC (5% methanol in dichloromethane): R_f = 0.19 (UV, CAM).

^{1}H NMR (599 MHz,DMSO-*d*6): δ = 9.65 (s, 1H, Ar-OH), 8.26 (s, 1H, N-H), 6.60 (s, 1H, H-20), 4.43 (s, 1H), 4.16 (dd, $^2J_{23A/23B}$ = 17.0 Hz, $^3J_{23A/NH}$ = 1.1 Hz, 1H, H-23$_A$), 4.05 (dd, $^2J_{23B/23A}$ = 17.0 Hz, $^3J_{23B/NH}$ = 1.1 Hz, 1H, H-23$_B$), 3.07 (d, $^2J_{15A/15B}$ = 17.8 Hz, 1H, H-15$_A$), 3.33 (m, 1H, H-3; re-assigned based on HSQC correlations), 2.37–2.30 (m, 1H, H-2$_A$), 2.25–2.18 (m, 1H, H-1$_A$), 2.18–2.11 (m, 1H, H-6$_A$), 2.09 (d, $^2J_{15B/15A}$ = 17.8 Hz, 1H, H-15$_B$), 2.01–1.93 (m, 1H, H-7$_A$), 1.75–1.71 (m, 1H, H-8), 1.67–1.61 (m, 1H, H-6$_B$), 1.59–1.51 (m, 2H, H-1$_B$, H-2$_B$), 1.46 (dd, $^3J_{5/6A/6B}$ = 13.0 Hz, $^3J_{5/6A/6B}$ = 3.9 Hz, 1H, H-5), 1.27 –1.24 (m, 1H, H-7$_B$), 1.09 (d, $^3J_{13/8}$ = 7.5 Hz, 3H, H-13), 0.92 (s, 3H, H-14), 0.88 (s, 3H, H-12), 0.83 (s, 3H, H-11).

^{13}C NMR (101 MHz,DMSO-*d*6): δ = 170.4 (C-22), 155.9 (C-21), 147.2 (C-17), 131.6 (C-19), 120.8 (C-18), 112.1 (C-16), 99.1 (C-20), 83.3 (C-10), 72.2 (C-3), 44.4 (C-5), 42.6 (C-23), 39.1 (C-8; re-assigned based on HSQC correlations), 37.5 (C-4), 37.1 (C-9), 32.0 (C-15), 30.1 (C-11), 27.6 (C-7), 27.1 (C-12), 25.8 (C-2), 23.5 (C-1), 23.5 (C-6), 20.0 (C-14), 17.0 (C-13).

IR (Diamond-ATR, neat): $\tilde{\nu}_{max}$ = 3350, 2927, 2356, 2334, 1693, 1600, 1478, 1358, 1066, 974.

HRMS (EI) calc. for $C_{23}H_{31}NO_4$ $[M]^+$: 385.2248; found: 385.2250.

$[\boldsymbol{\alpha}]_D^{20}$ = +129.3° (*c* = 0.53, MeOH); lit. $[\boldsymbol{\alpha}]_D^{24.5}$ = +138.7° (c = 1.0, MeOH) (+)-stachyflin.[15]

Supplementary Table 2 Comparison of ^{1}H NMR data for synthetic and natural (+)-stachyflin (**1**).

**Proton**	**Synthetic (599 MHz, DMSO-*d*6)**	**Natural (600 MHz, DMSO-*d*6)**[15]	**Δ: δ (ppm)**
1A	2.25–2.18 (m, 1H)	2.21 (m, 1H)	+ 0.01
1B	1.59–1.51 (m, 2H)	1.57 (m, 1H)	– 0.01
2A	2.37–2.30 (m, 1H)	2.34 (m, 1H)	– 0.01
2B	1.59–1.51 (m, 2H)	1.54 (m, 1H)	± 0.00
3	3.33 (m, 1H)	3.34 (m, 1H)	– 0.01
5	1.46 (dd, *J* = 13.0, 3.9 Hz, 1H)	1.46 (dd, *J* = 13.0, 3.0 Hz, 1H)	± 0.00
6A	2.18–2.11 (m, 1H)	2.12 (m, 1H)	+ 0.03
6B	1.67–1.61 (m, 1H)	1.64 (m, 1H)	± 0.00
7A	2.01–1.93 (m, 1H)	1.97 (m, 1H)	± 0.00
7B	1.27–1.24 (m, 1H)	1.25 (m, 1H)	± 0.01
8	1.75–1.71 (m, 1H)	1.73 (m, 1H)	± 0.00
11	0.83 (s, 3H)	0.83 (s, 3H)	± 0.00
12	0.88 (s, 3H)	0.89 (s, 3H)	– 0.01
13	1.09 (d, *J* = 7.5 Hz, 3H)	1.09 (d, *J* = 7.5 Hz, 3H)	± 0.00
14	0.92 (s, 3H)	0.92 (s, 3H)	± 0.00
15A	3.07 (d, *J* = 17.8 Hz, 1H)	3.07 (d, *J* = 17.9 Hz, 1H)	± 0.00
15B	2.09 (d, *J* = 17.8 Hz, 1H)	2.09 (d, *J* = 17.9 Hz, 1H)	± 0.00
20	6.60 (s, 1H)	6.61 (s)	– 0.01
23A	4.16 (dd, *J* = 17.0, 1.1 Hz, 1H)	4.16 (d, *J* = 16.8 Hz, 1H)	± 0.00
23B	4.05 (dd, *J* = 17.0, 1.1 Hz, 1H)	4.06 (d, *J* = 16.8 Hz, 1H)	– 0.01
Alk-OH	4.43 (s, 1H)	4.46 (d, *J* = 3.0 Hz, 1H)	– 0.03
Ar-OH	9.65 (s, 1H)	9.70 (s)	– 0.05
NH	8.26 (s, 1H)	8.29 (s)	– 0.03

Supplementary Table 3 Comparison of ^{13}C NMR data for synthetic and natural (+)-stachyflin (**1**).

**Carbon**	**Synthetic (101 MHz, DMSO-*d*6)**	**Natural (150 MHz, DMSO-*d*6)**[15]	**Δ: δ (ppm)**
1	23.5	23.4	+ 0.1
2	25.8	25.6	+ 0.1
3	72.2	72.1	+ 0.1
4	37.5	37.3	+ 0.2
5	44.4	44.2	+ 0.2
6	23.5	23.3	+ 0.2
7	27.6	27.5	+ 0.1
8	39.1	39.0	+ 0.1
9	37.1	37.0	+ 0.1
10	83.3	83.2	+ 0.1
11	30.1	30.0	+ 0.1
12	27.1	26.9	+ 0.2
13	17.0	16.9	+ 0.1
14	20.0	19.8	+ 0.2
15	32.0	31.8	+ 0.2
16	112.1	111.9	+ 0.2
17	147.2	147.1	+ 0.1
18	120.8	120.7	+ 0.1
19	131.6	131.5	+ 0.1
20	99.1	99.0	+ 0.1
21	155.9	155.8	+ 0.1
22	170.4	170.4	± 0.0
23	42.6	42.4	+ 0.2

Isoindole-3-one 38

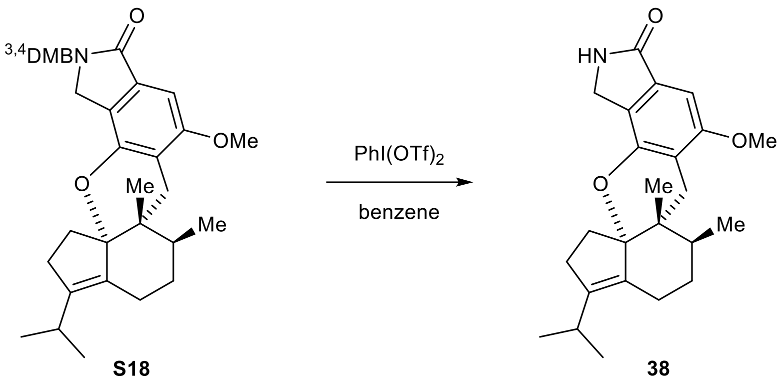

S18 **38**

Phenyliodine(III) bis(trifluoroacetate) (228 mg, 530 µmol, 10.0 equiv) was added to a solution of **S18** (28.2 mg, 53.0 µmol, 1 equiv) in benzene (5 mL). After 18 h, additional phenyliodine(III) bis(trifluoroacetate) (PIFA) (182 mg, 424 µmol. 8.00 equiv) was added in small portions over a period of 4 h (2 equiv/h). After 1 h, saturated aqueous sodium thiosulfate solution (50 mL) was added and the biphasic mixture was extracted with ethyl acetate (3 × 40 mL). The combined organic extracts were washed with saturated aqueous sodium chloride solution (2× 80 mL) and the washed solution was dried over magnesium sulfate. The dried solution was filtered and the filtrate was concentered. The residue was filtered through a plug of silica, the filtercake was rinsed with a mixture of 5% methanol in dichloromethane and used without further purification. An analytically pure sample of **38** was obtained by normal-phase semi-preparative HPLC purification using 10% *iso*-propanol in *n*-heptane as eluent (flow rate: 15 mL/min; column: Microsorb 60-8 Si Dynamax 250 × 21.4mm (R00083121C); detection: 254 nm; retention time: 13.7 min).

TLC (5% methanol in dichloromethane): R_f = 0.31 (UV, CAM).

^{1}H NMR (400 MHz, $CDCl_3$): δ = 6.89 (s, 1H), 6.23 (s, 1H), 4.29 (s, 2H), 3.87 (s, 3H), 2.99 (d, *J* = 18.1 Hz, 1H), 2.86–2.75 (m, 1H), 2.51–2.42 (m, 1H), 2.42–2.34 (m, 1H), 2.31 (d, *J* = 18.1 Hz, 1H), 2.27–2.13 (m, 2H), 2.12–2.03 (m, 1H), 1.82–1.75 (m, 1H), 1.72–1.64 (m, 1H), 1.56–1.46 (m, 1H), 1.36–1.24 (m, 1H), 1.06 (d, *J* = 6.9 Hz, 3H), 1.02 (d, *J* = 6.9 Hz, 3H), 0.81 (s, 3H), 0.77 (d, *J* = 6.9 Hz, 3H).

^{13}C NMR (101 MHz, $CDCl_3$): δ = 172.2, 158.7, 149.2, 143.4, 132.0, 130.7, 124.3, 114.1, 95.8, 95.2, 56.0, 43.4, 37.7, 32.1, 31.6, 30.5, 29.3, 28.0, 27.1, 22.5, 22.2, 20.8, 16.1, 15.8.

IR (Diamond-ATR, neat): $\tilde{\nu}_{max}$ = 2926, 2971, 1697, 1583, 1573, 1427, 1378, 1174, 1001, 934.

HRMS (EI) calc. for $C_{24}H_{31}NO_3$ $[M]^+$: 381.2298; found: 381.2296.

$[\boldsymbol{a}]_{\boldsymbol{D}}^{\boldsymbol{20}}$ = –14.6° (*c* = 0.27, CH_2Cl_2).

Melting point: 143–150 °C

Phenol 39

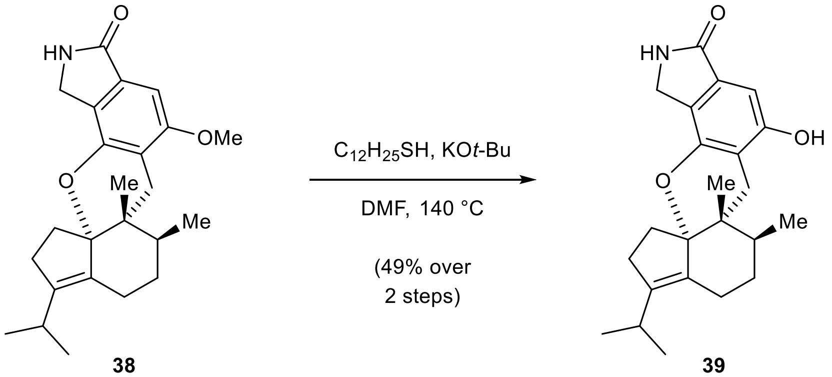

Potassium *tert*-butoxide (29.7 mg, 265 µmol, 5.00 equiv) was added to a solution of crude **38** (20.2 mg, 53.0 µmol, 1 equiv) and 1-dodecanethiol (76.2 µL, 318 µmol, 6.00 equiv) in *N*,*N*-dimethylformamide (0.9 mL) at 23 °C. After 5 min, the bright yellow suspension was heated to 140 °C. After 45 min, the dark orange reaction mixture was allowed to cool to 23 °C and saturated aqueous ammonium chloride solution (30 mL) was added and the aqueous layer was extracted with ethyl acetate (4 × 30 mL). The combined organic extracts were washed with saturated aqueous sodium chloride solution (2 × 40 mL), the washed solution was dried over magnesium sulfate, the dried solution was filtered and the filtrate was concentrated. The residue was filtered through a short plug of silica, the filtercake was rinsed with a mixture of 5% methanol in dichloromethane and was purified by normal-phase semi-preparative HPLC purification using 5% *iso*-propanol in *n*-heptane grading to 15% *iso*-propanol in *n*-heptane over 30 min as eluent (flow rate: 15 mL/min; column: Microsorb 60-8 Si Dynamax 250 × 21.4mm (R00083121C); detection: 254 nm; retention times: 14.3 min) to give **39** (9.0 mg, 49% over 2 steps) as a colorless solid

TLC (5% methanol in dichloromethane): R_f = 0.28 (UV, CAM).

^{1}H NMR (599 MHz, $CDCl_3$): δ = 7.07 (s, 1H), 6.41 (s, 1H), 4.28 (s, 2H), 3.01 (d, *J* = 17.8 Hz, 1H), 2.85–2.76 (m, 1H), 2.52–2.42 (m, 1H), 2.42–2.34 (m, 2H), 2.26–2.15 (m, 2H), 2.11–2.05 (m, 1H), 1.86–1.76 (m, 1H), 1.73–1.67 (m, 1H), 1.56–1.46 (m, 1H), 1.35–1.24 (m, 1H), 1.06 (d, *J* = 6.9 Hz, 3H), 1.02 (d, *J* = 6.9 Hz, 3H), 0.81 (s, 3H), 0.77 (d, *J* = 6.9 Hz, 3H).

^{13}C NMR (151 MHz, $CDCl_3$): δ = 172.5, 155.4, 149.5, 143.5, 132.0, 130.5, 123.4, 113.3, 100.9, 95.4, 43.6, 37.8, 32.1, 31.6, 30.5, 29.3, 27.9, 27.1, 22.5, 22.2, 20.8, 16.1, 15.8.

IR (Diamond-ATR, neat): $\tilde{\nu}_{max}$ = 3234, 2935, 2959, 1683, 1609, 1465, 1360, 1240, 1076, 934.

HRMS (EI) calc. for $C_{23}H_{28}NO_3$ $[M]^+$: 367.2142; found: 367.246.

$[\boldsymbol{\alpha}]_D^{20}$ = –85.3° (*c* = 0.15, CH_2Cl_2).

Melting point: 156–161 °C

Synthesis of Aldehyde 33

2,2-Dimethylcyclohexan-1-one (S22)

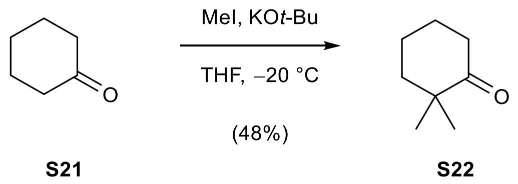

Note: The alkylation was carried out in three parallel 12.3 g batches.

Potassium *tert*-butoxide (29.6 g, 263 mmol, 2.10 equiv) was added to a solution of cyclohexanone (**S21**) (12.3 g, 125 mmol, 1 equiv) and methyl iodide (37.3 g, 263 mmol, 2.10 equiv) in tetrahydrofuran (600 mL) at −20 °C. After 15 h, saturated aqueous ammonium chloride solution (200 mL) was added to the reaction mixture. The layers were separated and the aqueous layer was extracted with diethyl ether (3 × 150 mL). The combined organic extracts were dried over sodium sulfate, the dried solution was filtered and the filtrate was concentrated. The residue was purified by flash-column chromatography on silica gel (20% diethyl ether in pentane) to yield **S22** (7.67 g, 48%) as a colorless oil. The obtained analytical data were in full agreement with those previously reported.[16]

2,2-Dimethyl-1-vinylcyclohexan-1-ol (S23)

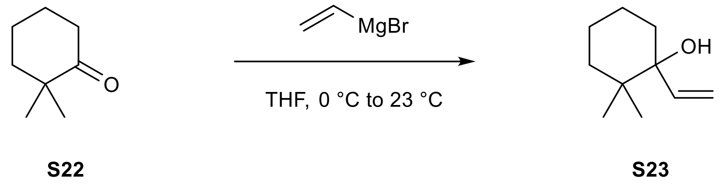

A solution of **S22** (23.0 g, 182 mmol, 1 equiv) in tetrahydrofuran (90 mL) was added dropwise to a solution of vinylmagnesium bromide (1 M in tetrahydroduran, 219 mL, 219 mmol, 1.20 equiv) over a period of 45 min at 0 °C. After 1.5 h, saturated aqueous ammonium chloride solution (500 mL) was added and the layers were separated. The aqueous layer was extracted with diethyl ether (2 × 300 mL) and the combined organic extracts were dried over sodium sulfate. The dried solution was filtered and the filtrate was concentrated to yield crude **S23** as a yellow oil. The residue was used without further purification in the next step.

Diene 31

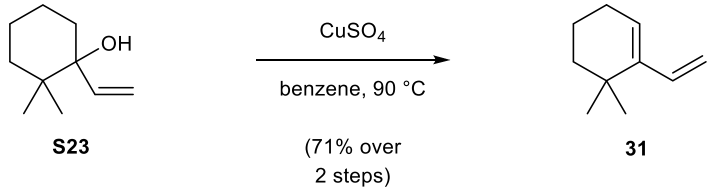

Note: $CuSO_4 \cdot 5H_2O$ was dried over night in a 140 °C oven.

To a solution of crude **S23** (28.1 g, 182 mmol, 1 equiv) in benzene (400 mL) was added anhydrous copper(II) sulfate (63.9 g, 400 mmol, 2.20 equiv) and the reaction mixture was heated to 90 °C under Dean-Stark conditions. After 16 h, the reaction mixture was allowed to cool to 23 °C, filtered through a pad of Celite® and was washed thoroughly with *n*-pentane. The filtrate was carefully concentrated (>220 mbar, 30 °C) and the residue was purified by flash-column chromatography on silica gel (*n*-pentane) to yield **31** as a yellow oil (17.7 g, 71% over 2 steps). The obtained analytical data were in full agreement with those previously reported.[17]

5,6-Dehydrodecalin S24

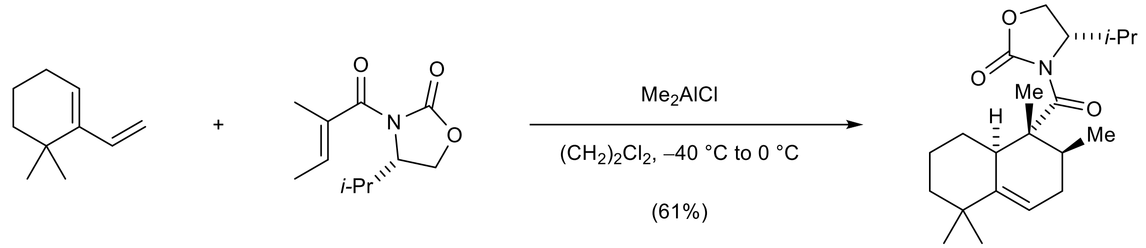

31 **21** **S24**

5,6-Dehydrogecalin **S24** was prepared according to the procedure described by A. J. Minnaard[18]:

A solution of dimethylaluminium chloride (1 M in hexanes, 93.0 mL, 93.0 mmol, 2.20 equiv) was added dropwise to a solution of **21** (9.83 g, 46.5 mmol, 1.10 equiv) in 1,2-dichloroethane (250 mL) over a period of 15 min at −40 °C. After 20 min, a solution of **30** (8.00 g, 42.3 mmol, 1 equiv) in 1,2-dichloroethane (100 mL) was added over a period of 15 min to the reaction mixture. After complete addition, the reaction mixture was allowed to warm to 23 °C. After 36 h, aqueous hydrogen chloride solution (1 M, 100 mL) was carefully added, the layers were separated and the aqueous layer was extracted with dichloromethane (3 × 100 mL). The combined organic extracts were dried over sodium sulfate, the dried solution was filtered and the filtrate was concentrated. The residue was purified by flash-column chromatography on silica gel (14% diethyl ether in *n*-pentane) to yield **S24** as a yellow highly viscous oil (9.02 g, 61%). The obtained analytical data were in full agreement with those previously reported.

Thioester 32

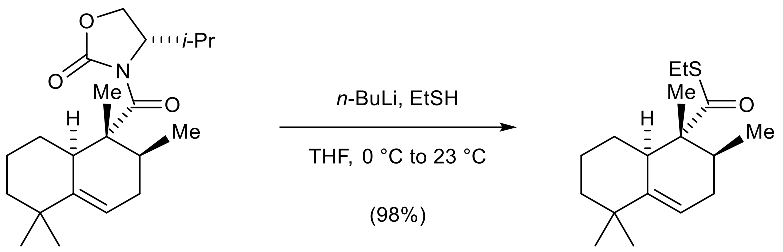

S24 **32**

A solution of *n*-butyllithium (2.40 M in hexanes 50.8 mL, 122 mmol, 4.70 equiv) was added dropwise to a solution of ethanethiol (11.3 mL, 153 mmol, 5.90 equiv) in tetrahydrofuran (250 mL) at 0 °C. After complete addition, the reaction mixture was slowly allowed to warm to 23 °C. After 30 min, a solution of **S24** (9.02 g,

26.0 mmol, 1 equiv) in tetrahydrofuran (70 mL) was added. After 7 h, diethyl ether (100 mL) and saturated aqueous ammonium chloride solution (100 mL) were added. The layers were separated, the aqueous layer was extracted with diethyl ether (3 × 100 mL) and the combined organic extracts were dried over sodium sulfate. The dried solution was filtered and the filtrate was concentrated. The residue was purified by flash-column chromatography on silica gel (2% diethyl ether in *n*-pentane) to yield **32** as a yellow oil (7.10 g, 98%). The obtained analytical data were in full agreement with those previously reported.[18]

Aldehyde 33

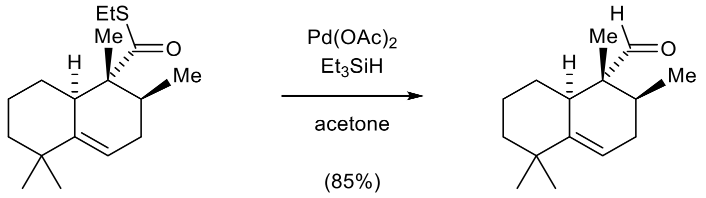

32 **33**

Triethylsilane (1.07 g, 9.20 mmol, 1.20 equiv) was added to a solution of thioester **32** (2.15 g, 7.67 mmol, 1 equiv) and palladium(II) acetate (103 mg, 460 µmol, 0.060 equiv) in acetone (60 mL). After 2.5 h, the dark brown solution was filtered through a plug of silica, the filtrate was concentrated and the residue was purified by flash-column chromatography on silica gel (2% ethyl acetate in hexanes) to provide **33** (1.43 g, 85%) as a colorless solid. The obtained analytical data were in full agreement with those previously reported.[18] Recrystallization from diethyl ether gave crystals suitable for single-crystal X-ray diffraction.

Synthesis of (+)-Aureol (2) and 5-*epi*-Aureol (9)

Benzylic alcohol (S25)

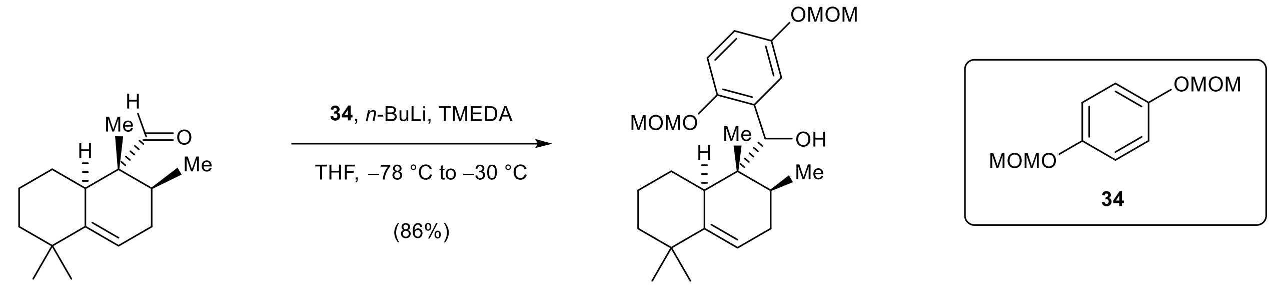

To a solution of **34**[19] (1.16 g, 5.85 mmol, 1.60 equiv) in tetrahydrofuran (8.5 mL) and freshly distilled *N*,*N*,*N′*,*N′*-tetramethylethane-1,2-diamine (TMEDA) (over CaH_2, 1.65 mL, 11.0 mmol, 3.00 equiv) was added a solution of *n*-butyllithium (2.40 M in hexanes, 2.28 mL, 5.48 mmol, 1.50 equiv) at –78 °C. After complete addition, the solution was allowed to warm to –30 °C. After 1.5 h, the reaction mixture was cooled to –78 °C and a solution of aldehyde **33** (805 mg, 3.65 mmol, 1 equiv) in tetrahydrofuran (3.5 mL) was added. The reaction mixture was allowed to warm to –30 °C over a period of 2 h, then diethyl ether (40 mL) and saturated aqueous ammonium chloride solution (40 mL) was added. The layers were separated and the aqueous layer was extracted with diethyl ether (3 × 40 mL). The combined organic extracts were washed with saturated aqueous sodium chloride solution (30 mL). The washed solution was dried over sodium sulfate and the dried solution was filtered. The filtrate was concentrated and the residue was purified by flash-column chromatography on silica gel (20% ethyl acetate in hexanes) to yield **S25** (1.32 g, 86%) as a colorless oil. The inconsequential mixture of diastereoisomers was partially characterized by HRMS and IR spectroscopy.

TLC (10% ethyl acetate in hexanes): R_f = 0.29 (CAM).

IR (Diamond-ATR, neat): $\tilde{\nu}_{max}$ = 3496, 2952, 1492, 1381, 1218, 1188, 1150, 1078, 1004, 922.

HRMS (EI) calcd for $C_{25}H_{38}O_5$ $[M]^+$: 418.2714; found: 418.2708.

Methoxymethyl-ether 35

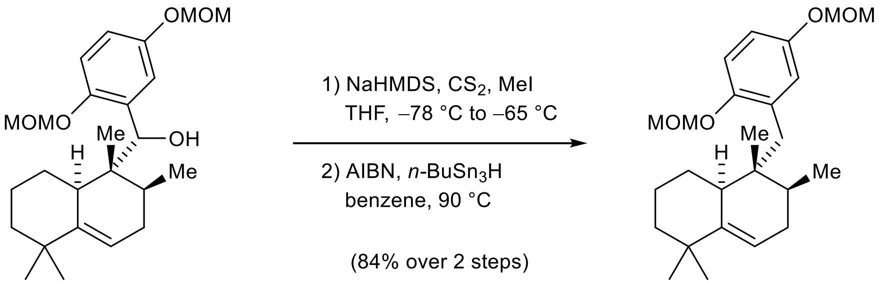

S25 **35**

A solution of sodium bis(trimethylsilyl)amide (1 M in tetrahydrofuran, 15.4 mL, 15.4 mmol, 5.00 equiv) was added dropwise to a solution of **S25** (1.29 g, 3.08 mmol, 1 equiv) in tetrahydrofuran (24 mL) at −78 °C. After 30 min, carbon disulfide (3.72 mL, 61.6 mmol, 20.0 equiv) was added dropwise to the orange solution and the resulting red solution was allowed to warm to −65 °C. After 1 h, methyl iodide (3.84 mL, 61.6 mmol, 20.0 equiv) was slowly added to the reaction mixture. After 1 h, saturated aqueous ammonium chloride solution (20 mL) was added, the layers were separated and the aqueous layer was extracted with ethyl acetate (3 × 50 mL). The combined organic extracts were dried over sodium sulfate, the dried solution was filtered and the filtrate was concentrated. The residue was filtered through a plug of silica and the obtained residue was used without further purification.

Note: benzene was degassed via freeze-pump-thaw (three cycles) prior to use.
A solution of the xanthogenate (1.57 g, 3.08 mmol, 1 equiv), 2,2'-azobis(2-methylpropionitrile) (AIBN) (253 mg, 1.54 mmol, 0.500 equiv) and tributyltin hydride (6.72 g, 23.1 mmol, 7.50 equiv) in benzene (50 mL) was heated to 90 °C. After 1.5 h, the reaction mixture was cooled to 23 °C and directly purified by flash-column chromatography on silica gel (hexanes initially, grading to 3% ethyl acetate in hexanes) to yield **35** as a colorless oil (1.05 g, 84% over 2 steps).

TLC (10% ethyl acetate in hexanes): R_f = 0.53 (CAM).

^{1}H NMR (599 MHz, $CDCl_3$): δ = 7.04 (d, *J* = 8.9 Hz, 1H), 6.91 (d, *J* = 3.0 Hz, 1H), 6.83 (d, *J* = 8.9 Hz, 1H), 5.39–5.36 (m, 1H), 5.15 (d, *J* = 6.6 Hz, 1H), 5.10 (s, 2H), 5.09 (d, *J* = 6.6 Hz, 1H), 3.49 (s, 3H), 3.47 (s, 3H), 2.82 (d, *J* = 13.6 Hz, 1H), 2.55 (d, *J* = 13.6 Hz, 1H), 2.13 (m, 1H), 1.96 (m, 1H), 1.80 (m, 2H), 1.55–1.47 (m, 2H), 1.47–1.42 (m, 1H), 1.39–1.34 (m, 1H), 1.17 m, 1H), 1.04 (d, *J* = 6.7 Hz, 3H), 1.02 (s, 3H), 0.95 (m, 1H), 0.90 (s, 3H), 0.78 (s, 3H).

^{13}C NMR (151 MHz, $CDCl_3$): δ = 152.0, 151.6, 146.8, 130.5, 120.5, 115.4, 115.0, 114.8, 95.7, 95.4, 56.1, 56.0, 41.5, 40.3, 39.9, 37.5, 36.5, 34.7, 31.8, 29.9, 29.9, 28.3, 22.7, 16.8, 16.4.

IR (Diamond-ATR, neat): $\tilde{\nu}_{max}$ = 2926, 1495, 1455, 1219, 1189, 1149, 1075, 1010, 922, 81.

HRMS (EI) calcd for $C_{25}H_{38}O_4$ $[M]^+$: 402.2765; found: 402.2757.

$[\alpha]_D^{22}$ = + 28.68° (c = 0.96, CH_2Cl_2).

(+)-Aureol (2)

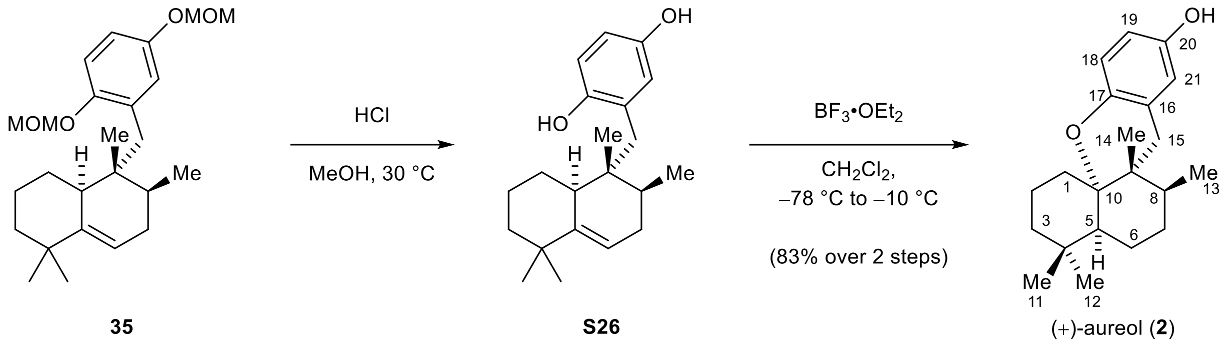

A solution of hydrochloric acid (~1.25 M in methanol, 9 mL) was added to a solution of **35** (300 mg, 744 µmol, 1 equiv) in dichloromethane (3 mL) and the resulting solution was heated to 30 °C. After 5 h, the reaction mixture was diluted with dichloromethane (25 mL) and saturated aqueous sodium bicarbonate solution (25 mL) was added. The layers were separated and the aqueous layer was extracted with dichloromethane (3 × 20 mL). The combined organic extracts were dried over sodium sulfate, the dried solution was filtered and the filtrate was concentrated to yield **S26** as a colorless foam that was directly used in the following step.

A solution of boron trifluoride diethyl etherate (48% in diethyl ether, 1.95 mL, 7.44 mmol, 10.0 equiv) was added dropwise to a solution of the crude *para*-hydroquinone **S26** (234 mg, 744 µmol, 1 equiv) in dichloromethane (30 mL) at –78 °C. After complete addition, the reaction mixture was allowed to warm to –10 °C over a period of 1.5 h. After 2 h at –10 °C, saturated aqueous ammonium chloride solution (20 mL) was added, the layers were separated and the aqueous layer was extracted with dichloromethane (3 × 20 mL). The combined organic extracts were dried over sodium sulfate, the dried solution was filtered and the filtrate was concentrated. The residue was purified by flash-column chromatography on silica gel (5% ethyl acetate in hexanes) to yield (+)-aureol (**2**) (193 mg, 83% over two steps) as a colorless foam.

TLC (20% ethyl acetate in hexanes): $R_f = 0.43$ (CAM).

^{1}H NMR (800 MHz, $CDCl_3$): δ = 6.61 (d, $^3J_{18/19}$ = 8.6 Hz, 1H, H-18), 6.56 (dd, $^3J_{18/19}$ = 8.6 Hz, $^4J_{18/21}$ = 3.0 Hz, 1H, H-19), 6.49 (d, $^4J_{21/19}$ = 3.0 Hz, 1H), 4.32 (s, 1H, O-H), 3.38 (d, $^2J_{15A/15B}$ = 17 Hz, 1H, H-15_A), 2.10–1.99 (m, 2H, 1_A, 2_A), 1.97 (d, $^2J_{15B/15A}$ = 17 Hz, 1H, H-15_B), 1.84–1.75 (m, 2H, H-7), 1.71–1.64 (m, 2H, H-6_A, H-8), 1.60–1.53 (m, 1H, H-6_B), 1.50–1.45 (m, 1H, H-2_B), 1.45–1.40 (m, 2H, H-3_A, H-5), 1.37–1.33 (m, 1H, H-1_B), 1.20–1.17 (m, 1H, H-3_B), 1.11 (d, $^3J_{13/8}$ = 7.5 Hz, 3H, H-13), 1.06 (s, 3H, H-11), 0.92 (s, 3H, H-14), 0.78 (s, 3H, H-14).

^{13}C NMR (201 MHz, $CDCl_3$): δ = 148.3 (C-20), 145.8 (C-17), 122.2 (C-16), 117.2 (C-18), 115.0 (C-19), 114.0 (C-21), 82.3 (C-10), 44.0 (C-5), 39.3 (C-8), 38.1 (C-9), 37.3 (C-15), 33.9 (C-4), 33.8 (C-3), 31.9 (C-12), 29.8 (C-11), 29.3 (C-7), 27.9 (C-1), 22.2 (C-6), 20.2 (C-14), 18.3 (C-2), 17.3 (C-13).

IR (Diamond-ATR, neat): $\tilde{v}_{max}$ = 3344, 2933, 1494, 1450, 1231, 1185, 1171, 1158, 952, 907, 733.

HRMS (EI) calcd for $C_{21}H_{30}O_2$ $[M]^+$: 314.2240; found: 314.2234.

$[\alpha]_D^{20}$ = +73.5° (c = 1.19, CCl_4); lit. $[\alpha]_D^{25}$ = +65.0 ° (c = 2.00, CCl_4) (+)-aureol.[20]

Supplementary Table 4 Comparison of ^{1}H NMR data for natural and synthetic (+)-aureol (**2**).

**Proton**	**Synthetic (800 MHz, $CDCl_3$)**	**Natural (n.a., $CDCl_3$)[20]**	**Δ: δ (ppm)**
1A	2.10–1.99 (m, 2H)		
1B	1.37–1.33 (m, 1H)		
2A	2.10–1.99 (m, 2H)		
2B	1.50–1.45 (m, 1H)		
3A	1.45–1.40 (m, 2H)		
3B	1.20–1.17 (m, 1H)		
5	1.45–1.40 (m, 2H)		
6A	1.71–1.64 (m, 2H)		
6B	1.60–1.53 (m, 1H)		
7	1.84–1.75 (m, 2H)		
8	1.71–1.64 (m, 2H)		
11	1.06 (s, 3H)	1.06 (s, 3H)	± 0.00
12	0.78 (s, 3H)	0.78 (s, 3H)	± 0.00
13	1.11 (d, *J* = 7.5 Hz, 3H)	1.11 (d, *J* = 7 Hz, 3H)	± 0.00
14	0.92 (s, 3H)	0.92 (s, 3H)	± 0.00
15A	3.38 (d, 1H)	3.38 (d, *J* = 16 Hz, 1H)	+ 0.00
15B	1.97 (d, *J* = 17.0 Hz, 1H),	1.96 (d, *J* = 16 Hz, 1H)	+ 0.01
18	6.61 (d, *J* = 8.6 Hz, 1H),		
19	6.56 (dd, *J* = 8.6, 3.0 Hz, 1H),		
21	6.49 (d, *J* = 3.0 Hz, 1H),	6.50 (br s, 1H)	– 0.01
OH	4.32 (s, 1H)	Not reported	

Supplementary Table 5 Comparison of ^{13}C NMR data for natural and synthetic (+)-aureol (**2**).

Carbon	Synthetic (201 MHz, $CDCl_3$)	Natural (n.a., $CDCl_3$)[20]	Δ: δ (ppm)
1	27.9	27.9	± 0.0
2	18.3	18.4	– 0.1
3	33.8	33.9	– 0.1
4	33.9	33.9	± 0.0
5	44.0	44.0	± 0.0
6	22.2	22.2	± 0.0
7	29.3	29.3	± 0.0
8	39.3	39.3	± 0.0
9	38.1	38.1	± 0.0
10	82.3	82.4	– 0.1
11	29.8	29.8	± 0.0
12	31.9	31.9	± 0.0
13	17.3	17.3	± 0.0
14	20.2	20.2	± 0.0
15	37.3	37.4	– 0.1
16	122.2	122.2	± 0.0
17	145.8	145.8	± 0.0
18	117.2	117.2	– 0.1
19	115.0	115.2	– 0.2
20	148.3	148.2	+ 0.1
21	114.0	114.2	– 0.2

Epi-aureol (9)

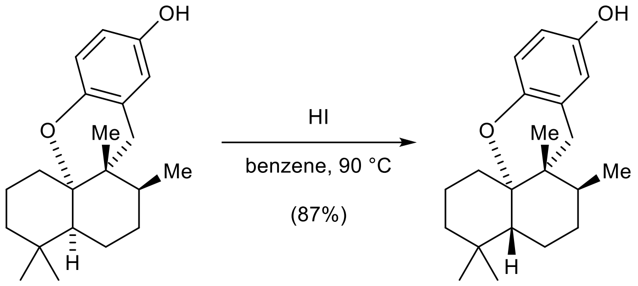

(+)-aureol (**2**) 5-*epi*-aureol (**9**)

A solution of hydroiodic acid (57 wt.% in H_2O, 63.0 µL, 477 µmmol, 10.0 equiv) was added to a solution of (+)-aureol (**2**) (15.0 mg, 47.7 µmol, 1 equiv) in benzene (4 mL) in a pressure tube. The tube was sealed and the reaction mixture was heated to 90 °C. After 24 h, the reaction mixture was cooled to 23 °C and saturated aqueous sodium bicarbonate solution (15 mL) and dichloromethane (10 mL) were added. The layers were separated and the aqueous layer was extracted with dichloromethane (3 × 15 mL). The combined organic extracts were dried over sodium sulfate, the dried solution was filtered and the filtrate was concentrated. The residue was purified by flash-column chromatography on silica gel (5% ethyl acetate in hexanes) to yield 5-*epi*-aureol (**9**) (13.0 mg, 87% over two steps) as a colorless foam. The obtained analytical data were in in full agreement with those values reported in literature.[21]

Synthesis of (+)-Strongylin A (4), 5-*epi*-Strongylin A (45), 46, 47, 3-Hydroxy-strongylin A (48) and 49

Arene Unit S28

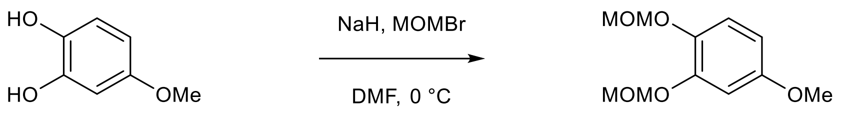

S27 **S28**

(80%)

To a solution of **S27**[22] (675 mg, 4.28 mmol, 1 equiv) in *N*,*N*-dimethylformamide (25 mL) was added in small portions sodium hydride (60% mineral oil dispersion, 482 mg, 12.0 mmol, 2.50 equiv) at 0 °C. After 30 min, bromomethyl methylether (1.51 g, 12.0 mmol, 2.50 equiv) was added dropwise to the dark green suspension and the resulting mixture was allowed to warm to 23 °C. After 13 h, saturated aqueous ammonium chloride solution (60 mL) and diethyl ether (80 mL) were added. The layers were separated and the aqueous layer was extracted with diethyl ether (3 × 60 mL). The combined organic extracts were washed with aqueous saturated sodium chloride solution (50 mL), the washed solution was dried over sodium sulfate, the dried solution was filtered and filtrate was concentrated. The residue was purified by flash-column chromatography on silica gel (20% ethyl acetate in hexanes) to provide **S28** (879 mg, 80%) as a colorless oil.

TLC (20% ethyl acetate in hexanes): R_f = 0.23 (UV, CAM).

^{1}H NMR (400 MHz, $CDCl_3$): δ = 7.06 (d, *J* = 8.8 Hz, 1H), 6.78 (d, *J* = 2.9 Hz, 1H), 6.47 (dd, *J* = 8.8, 2.9 Hz, 1H), 5.21 (s, 2H), 5.14 (s, 2H), 3.76 (s, 3H), 3.52 (s, 3H), 3.51 (s, 3H).

^{13}C NMR (101 MHz, $CDCl_3$): δ = 155.5, 148.5, 141.3, 118.4, 106.2, 104.2, 96.5, 95.5, 56.3, 56.2, 55.8.

IR (Diamond-ATR, neat): $\tilde{\nu}_{max}$ = 2902, 2829, 1609, 1506, 1259, 1219, 1150, 1072, 1039, 984, 921.

HRMS (EI) calcd for: $C_{11}H_{16}O_5$ $[M]^+$: 228.0992; found: 228.0989.

Benzylic alcohol S29

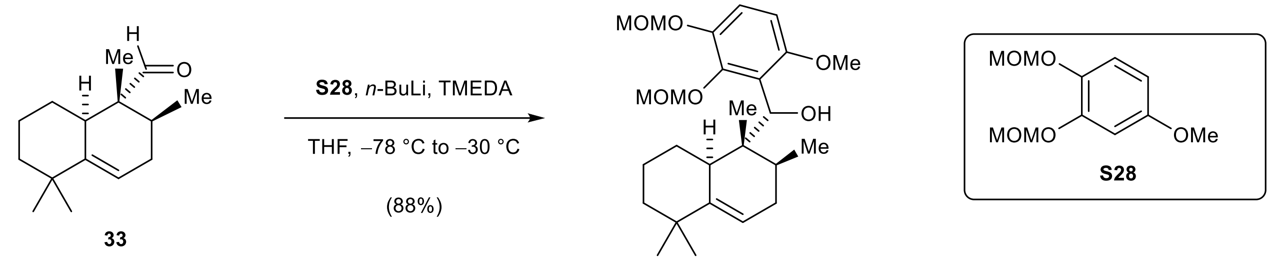

To a solution of **S28** (109 mg, 478 µmol, 1.40 equiv) in tetrahydrofuran (2 mL) and freshly distilled *N*,*N*,*N'*,*N'*-tetramethylethane-1,2-diamine (over CaH_2, 150 µL, 1.02 mmol, 3.00 equiv) was added a solution of *n*-butyllithium (2.44 M in hexanes, 128 µL, 443 µmol, 1.30 equiv) at –78 °C. After 10 min, the reaction mixture was allowed to warm to –30 °C. After 1.5 h, the reaction mixture was cooled to –78 °C and a solution of aldehyde **33** (75.2 mg, 341 µmol, 1 equiv) in tetrahydrofuran (1 mL) was added. The reaction mixture was allowed to warm to –30 °C over a period of 2 h. Diethyl ether (15 mL) and saturated aqueous ammonium chloride solution (15 mL) were added. The layers were separated and the aqueous layer was extracted with diethyl ether (3 × 15 mL). The combined organic extracts were washed with saturated aqueous sodium chloride solution (20 mL), the washed solution was dried over sodium sulfate and the dried solution was filtered. The filtrate was concentrated and the residue was purified by flash-column chromatography on silica gel (20% ethyl acetate in hexanes) to yield **S29** (153 mg, 88%) as a colorless oil. The inconsequential mixture of diastereoisomers was partially characterized by HRMS and IR spectroscopy.

TLC (10% ethyl acetate in hexanes): $R_f = 0.23$ (CAM).

IR (Diamond-ATR, neat): $\tilde{\nu}_{max}$ = 3552, 2925, 1591, 1483, 1357, 1251, 1152, 1069, 977, 924, 805.

HRMS (EI) calcd for $C_{26}H_{40}O_6$ $[M]^+$: 448.2819; found: 448.2816.

Olefin S30

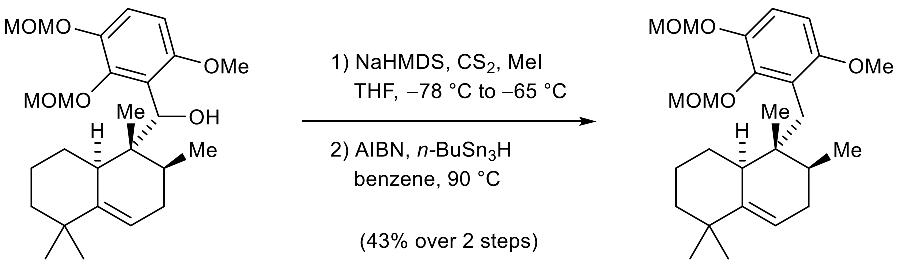

S29 **S30**

A solution of sodium bis(trimethylsilyl)amide (1 M in tetrahydrofuran, 1.53 mL, 1.53 mmol, 5.00 equiv) was added dropwise to a solution of **S29** (137 mg, 305 µmol, 1 equiv) in tetrahydrofuran (2 mL) at −78 °C. After 30 min, carbon disulfide (370 µL, 610 µmol, 20.0 equiv) was slowly added and the reaction mixture was allowed to warm to −65 °C. After 1 h, methyl iodide (380 µL, 6.10 mmol, 20.0 equiv) was slowly added to the reaction mixture. After 1 h, saturated aqueous ammonium chloride solution (10 mL) and ethyl acetate (10 mL) were added to the reaction mixture, the layers were separated and the aqueous layer was extracted with ethyl acetate (3 × 10 mL). The combined organic extracts were dried over sodium sulfate, the dried solution was filtered and the filtrate was concentrated. The residue was filtered through a short plug of silica and the obtained residue was used without further purification.

Note: benzene was degassed via freeze-pump-thaw (three cycles) prior to use.
A degassed solution of the xanthogenate (164 mg, 305 µmol, 1 equiv), 2,2'-azobis(2-methylpropionitrile) (AIBN) (25.0 mg, 152 µmol, 0.500 equiv) and tributyltin hydride (666 mg, 2.29 mmol, 7.50 equiv) in benzene (6 mL) was heated to 90 °C. After 4 h, the reaction mixture was cooled to 23 °C and directly purified by flash-column chromatography on silica gel (hexanes initially, grading to 3% ethyl acetate in hexanes) to yield **S30** as a colorless oil (57.0 mg, 43% over 2 steps).

TLC (20% ethyl acetate in hexanes): $R_f = 0.35$ (CAM).

^{1}H NMR (800 MHz, C_6D_6): δ = 6.99 (d, J = 8.9 Hz, 1H), 6.28 (d, J = 8.9 Hz, 1H), 5.61–5.54 (m, 1H), 5.15–5.09 (m, 2H), 4.92–4.88 (m, 2H), 3.41 (s, 3H), 3.35 (s, 3H), 3.24 (d, J = 12.9 Hz, 1H), 3.22 (s, 3H), 2.97 (d, J = 12.9 Hz, 1H), 2.90–2.86 (m, 1H), 2.01–1.97 (m, 2H), 1.79–1.74 (m, 1H), 1.61–1.54 (m, 1H), 1.51–1.47 (m, 1H), 1.46–1.40 (m, 2H), 1.27–1.22 (m, 1H), 1.21 (s, 3H), 1.17 (d, J = 6.7 Hz, 3H), 1.16 (s, 3H), 1.06 (s, 3H), 0.94–0.88 (m, 1H).

^{13}C NMR (201 MHz, C_6D_6): δ = 154.7, 148.6, 147.7, 144.5, 125.0, 115.8, 115.6, 105.5, 99.6, 96.4, 57.5, 55.8, 55.1, 42.6, 41.9, 41.2, 39.4, 36.9, 35.9, 32.3, 31.5, 30.2, 28.4, 23.4, 16.8, 14.8.

IR (Diamond-ATR, neat): $\tilde{\nu}_{max}$ = 2928, 2837, 1483, 1464, 1250, 1154, 1069, 1038, 983, 951, 801.

HRMS (EI) calcd for $C_{26}H_{40}O_5$ $[M]^+$: 432.2870; found: 432.2865.

$[\alpha]_D^{20}$ = +13.1° (c = 5.38, CH_2Cl_2).

(+)-Strongylin A (4)

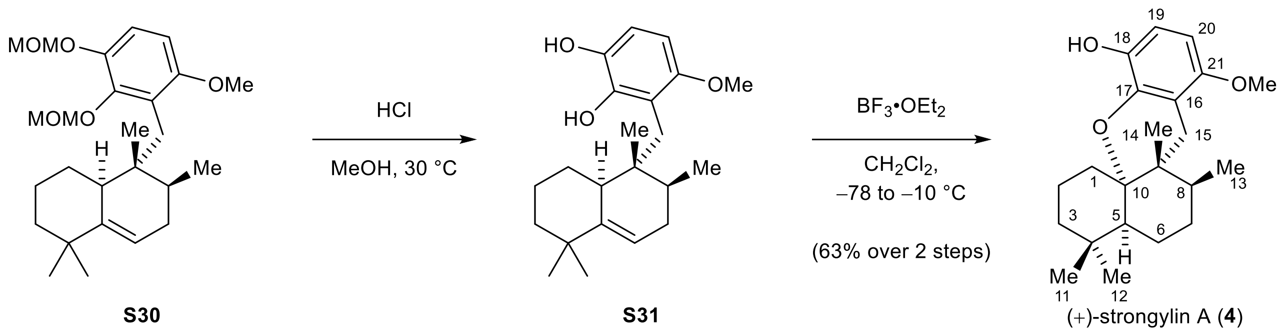

A solution of hydrochloric acid (~1.25 M in methanol, 4 mL) was added to a solution of **S30** (33.0 mg, 76.0 µmol, 1 equiv) in dichloromethane (2 mL) and the resulting solution was heated to 30 °C. After 6.5 h, the reaction mixture was diluted with dichloromethane (5 mL) and saturated aqueous sodium bicarbonate solution (10 mL) was added. The layers were separated and the aqueous layer was extracted with dichloromethane (3 × 10 mL). The combined organic extracts were dried over sodium sulfate, the dried solution was filtered and the filtrate was concentrated to yield **S31** as a colorless solid that was directly used in the following step.

A solution of boron trifluoride diethyl etherate (48% in diethyl ether, 140 µL, 534 µmol, 7.00 equiv) was added dropwise to a solution of crude hydroquinone **S31** (26.0 mg, 76.0 µmol, 1 equiv) in dichloromethane (3 mL) at –78 °C and the reaction mixture was allowed to warm to –30 °C over a period of 30 min. After 1.5 h, saturated aqueous ammonium chloride solution (10 mL) and dichloromethane (5 mL) were added. The layers were separated and the aqueous layer was extracted with dichloromethane (3 × 10 mL). The combined organic extracts were dried over sodium sulfate, the dried solution was filtered and the filtrate was concentrated. The residue was purified by flash-column chromatography on silica gel (5% ethyl acetate in hexanes) to yield (+)-strongylin A (**4**) (16.6 mg, 63% over two steps) as a colorless foam.

TLC (20% ethyl acetate in hexanes): R_f = 0.50 (CAM).

^{1}H NMR (400 MHz, C_6D_6): δ = 6.96 (d, $^3J_{19/20}$ = 8.6 Hz, 1H, H-19), 6.13 d, $^3J_{20/19}$ = 8.6 Hz, 1H, H-20), 5.12 (s, 1H, O-H), 3.42 (s, 3H. H-22), 3.23 (d, $^2J_{15A/15B}$ = 17.7 Hz, 1H, H-15$_A$), 2.33 (d, $^2J_{15B/15A}$ = 17.7 Hz, 1H, H-15$_B$), 1.93–1.78 (m, 2H, H-2$_A$, H7$_A$), 1.69–1.51 (m, 3H, H-1$_A$, H-1$_B$, H-8), 1.45–1.36 (m, 2H, H-5, H-6$_A$), 1.33–1.18 (m, 3H, H-2$_B$, H-3$_A$, H-6$_B$), 1.12–1.04 (m, 5H, H-3$_B$, H-7$_B$, H-12), 0.88 (d, $^3J_{13/8}$ = 7.6 Hz, 3H, H-13), 0.82 (s, 3H, H-14), 0.64 (s, 3H, H-11).

^{13}C NMR (101 MHz, C_6D_6): δ = 151.4 (C-21), 139.8 (C-17), 139.6 (C-18), 111.5 (C-19), 110.7 (C-16), 100.9 (C-20), 83.9 (C-10), 55.0 (C-22), 44.0 (C-5), 39.8 (C-8), 38.3 (C-9), 34.0 (C-3), 33.5 (C-4), 32.9 (C-15), 32.2 (C-11), 29.5 (C-1), 29.3 (C-12), 28.0 (C-7), 22.7 (C-6), 20.3 (C-14), 18.9 (C-2), 17.4 (C-13).

IR (Diamond-ATR, neat): $\tilde{\nu}_{max}$ = 3567, 2953, 1490, 1384, 1264, 1182, 1107, 1089, 946.

HRMS (EI) calcd for $C_{22}H_{32}O_3$ $[M]^+$: 344.2346; found: 344.2346.

$[\alpha]_D^{20}$ = +66.3° (c = 1.46, CH_2Cl_2); lit. $[\alpha]_D^{20}$ = +72.0° (c = 0.012, CH_2Cl_2) (+)-strongylin A.[23]

Supplementary Table 6 Comparison of ^{1}H NMR data for natural and synthetic (+)-strongylin A (**4**).

Proton	Synthetic (400 MHz, C_6D_6)	Natural (360 MHz, C_6D_6)[23]	Δ: δ (ppm)
1	1.69–1.51 (m, 3H)	1.66 (2H, m, 2H)	
2A	1.93–1.78 (m, 2H)	1.86 (m, 2H)	± 0.00
2B	1.33–1.18 (m, 3H)	1.32 (m, 1H)	
3A	1.33–1.18 (m, 3H)	1.25 (m, 2H)	
3B	1.12–1.04 (m, 5H)	1.12 (m, 2H)	
5	1.45–1.36 (m, 2H)	1.43 (m, 1H)	
6A	1.45–1.36 (m, 2H)	1.41 (m, 1)H	
6B	1.33–1.18 (m, 3H)	1.25 (m, 2H)	± 0.00
7A	1.93–1.78 (m, 2H)	1.86 (m, 2H)	± 0.00
7B	1.12–1.04 (m, 5H)	1.12 (m ,2H)	
8	1.69–1.51 (m, 3H)	1.60 (m, 1H)	
11	0.64 (s, 3H)	0.65 (s, 3H)	– 0.01
12	1.12–1.04 (m, 5H)	1.09 (s, 3H)	
13	0.88 (d, *J* = 7.6 Hz, 3H)	0.90 (d, *J* = 7.6 Hz, 3H)	– 0.02
14	0.82 (s, 3H)	0.82(s, 3H)	± 0.00
15A	3.23 (d, *J* = 17.7 Hz, 1H)	3.20 (d, *J* = 7.7 Hz, 1H)	+ 0.03
15B	2.33 (d, *J* = 17.7 Hz, 1H)c	2.30 (d, *J*= 7.7 Hz, 1H)	+ 0.03
19	6.96 (d, *J* = 8.6 Hz, 1H)	6.91 (d, *J* = 8.6 Hz, 1H)	+ 0.05
20	6.13 (d, *J* = 8.6 Hz, 1H)	6.13 (d, *J* = 8.6, 1H)	± 0.00
22	3.42 (s, 3H)	3.44 (s, 3H)	– 0.02
OH	5.12 (s, 1H)	5.12 (s, 1H)	± 0.00

Supplementary Table 7 Comparison of ^{13}C NMR data for natural and synthetic (+)-strongylin A (**4**).

Carbon	Synthetic (101 MHz, C_6D_6)	Natural (90 MHz, C_6D_6)[23]	Δ: δ (ppm)
1	29.5	29.5	± 0.0
2	18.9	18.9	± 0.0
3	34.0	34.0	± 0.0
4	33.5	33.5	± 0.0
5	44.0	44.0	± 0.0
6	22.7	22.7	± 0.0
7	28.0	27.9	+ 0.1
8	39.8	39.8	± 0.0
9	38.3	38.3	± 0.0
10	83.9	83.8	+ 0.1
11	32.2	32.1	+ 0.1
12	29.3	29.2	+ 0.1
13	17.4	17.4	± 0.0
14	20.3	20.5	– 0.2
15	32.9	32.8	+ 0.1
16	110.7	110.7	± 0.0
17	139.8	139.7	+ 0.1
18	139.6	139.4	+ 0.2
19	111.5	111.5	± 0.0
20	100.9	100.8	+ 0.1
21	151.4	151.3	+ 0.1
22	55.0	55.0	± 0.0

5-*epi*-strongylin A (45)

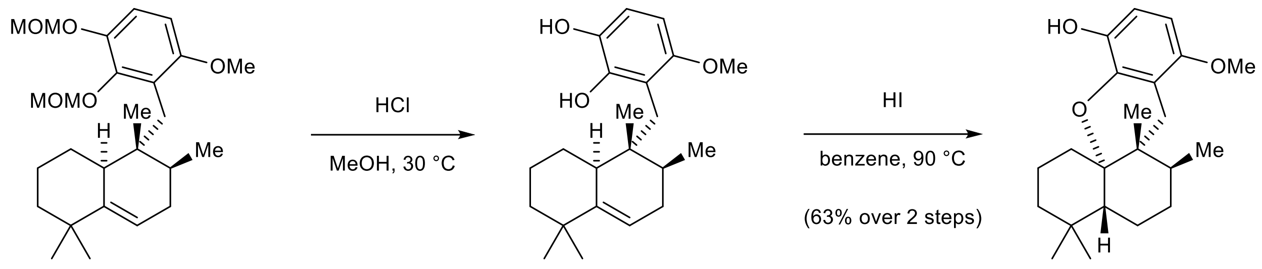

S30 **S31** 5-*epi*-strongylin A (**45**)

A solution of hydrochloric acid (~1.25 M in methanol, 9 mL) was added to a solution of **S30** (117 mg, 270 µmol, 1 equiv) in dichloromethane (7 mL) at 23 °C and the mixture was heated to 30 °C. After 6 h, the reaction mixture was diluted with dichloromethane (30 mL) and saturated aqueous sodium bicarbonate solution (20 mL) was added. The layers were separated and the aqueous layer was extracted with dichloromethane (3 × 20 mL). The combined organic extracts were dried over sodium sulfate, the dried solution was filtered and the filtrate was concentrated to yield **S31** as a colorless solid that was directly used in the following reaction.

A solution of hydroiodic acid (57 wt.% in water, 357 µL, 2.70 mmol, 10.0 equiv) was added to a solution of the crude hydroquinone **S31** (93.2 mg, 270 µmol, 1 equiv) in benzene (10 mL) in an Ace® pressure tube. The tube was sealed and the reaction mixture was heated to 90 °C. After 16 h, the reaction mixture was cooled to 23 °C and saturated aqueous sodium bicarbonate solution (15 mL) and dichloromethane (10 mL) were added. The layers were separated and the aqueous layer was extracted with dichloromethane (3 × 15 mL). The combined organic extracts were dried over sodium sulfate, the dried solution was filtered and the filtrate was concentrated. The residue was purified by flash-column chromatography on silica gel (5% ethyl acetate in hexanes) to yield *epi*-strongylin A (**45**) (59.0 mg, 63% over two steps) as a colorless foam.

TLC (20% ethyl acetate in hexanes): R_f = 0.47 (CAM).

^{1}H NMR (800 MHz, C_6D_6): δ = 6.99 (d, *J* = 8.6 Hz, 1H), 6.13 (d, *J* = 8.6 Hz, 1H), 5.38 (s, 1H), 3.40 (s, 3H), 2.98 (d, *J* = 17.8 Hz, 1H), 2.30 (dd, *J* = 17.8, 1.0 Hz, 1H), 1.69–1.61 (m, 2H), 1.60–1.51 (m, 2H), 1.40–1.35 (m, 1H), 1.32–1.25 (m, 3H), 1.14–1.06 (m, 2H), 1.04 (s, 3H), 1.03–0.96 (m, 2H), 0.81 (s, 3H), 0.74 (s, 3H), 0.68 (d, *J* = 6.8 Hz, 3H).

^{13}C NMR (201 MHz, C_6D_6): δ = 149.8, 138.88, 138.86, 109.9, 109.2, 100.0, 81.5, 53.7, 44.5, 40.1, 35.9, 31.8, 31.1, 30.6, 29.1, 27.0, 27.0, 21.3, 20.6, 16.7, 15.7, 15.0.

IR (Diamond-ATR, neat): $\tilde{\nu}_{max}$ = 3557, 2938, 1618, 1489, 1390, 1264, 1170, 1083, 918, 788.

HRMS (EI) calcd for $C_{22}H_{32}O_3$ $[M]^+$: 344.2346; found: 344.2336.

$[\boldsymbol{\alpha}]_{\boldsymbol{D}}^{\mathbf{20}}$ = –8.7° (c = 0.21, CH_2Cl_2).

Quinone 46

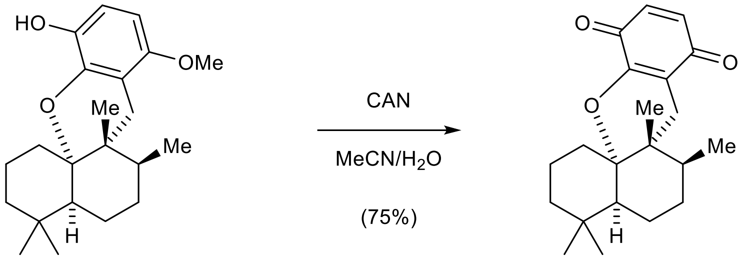

(+)-strongylin A (**4**) **46**

A solution of diammonium cerium(IV) nitrate (28.2 mg, 51.5 µmol, 2.50 equiv) in water (2.5 mL) was added dropwise to a solution of strongylin A (**4**) (7.10 mg, 21.0 µmol, 1 equiv) in acetonitrile (2.5 mL) over a period of 40 min at 0 °C. After 2 h, the bright yellow solution was diluted with water (10 mL) and the mixture was extracted with diethyl ether (3 × 10 mL). The combined organic extracts were dried over sodium sulfate, the dried solution was filtered and the filtrate was concentrated. The residue was purified by flash-column chromatography on silica gel (7% diethyl ether in pentane) to yield **46** (5.10 mg, 75%) as a yellow oil.

TLC (10% ethyl acetate in hexanes): R_f = 0.25 (UV, CAM).

^{1}H NMR (599 MHz, $CDCl_3$): δ = 6.64 (d, *J* = 10.2, 1H), 6.57 (d, *J* = 10.2 Hz, 1H), 2.86 (d, *J* = 18.7 Hz, 1H), 2.17–2.06 (m, 1H), 2.05–1.97 (m, 1H), 1.94 (d, *J* = 18.7 Hz, 1H), 1.89–1.80 (m, 2H), 1.80–1.72 (m, 2H), 1.60–1.51 (m, 2H), 1.44–1.35 (m, 3H), 1.25–1.21 (m, 1H), 1.09 (d, *J* = 7.5, 1.2 Hz, 3H), 0.99 (s, 3H), 0.87 (s, 3H), 0.83 (s, 3H).

^{13}C NMR (151 MHz, $CDCl_3$): δ = 187.4, 181.6, 150.6, 137.0, 134.0, 117.9, 87.5, 45.1, 39.2, 38.1, 33.9, 33.6, 32.1, 30.9, 29.8, 29.1, 27.9, 22.6, 20.3, 18.4, 17.3.

IR (Diamond-ATR, neat): $\tilde{v}_{max}$ = 2931, 2360, 1675, 1646, 1598, 1393, 1220, 1048, 839, 733.

HRMS (EI) calcd for $C_{21}H_{28}O_3$ $[M]^+$: 328.2033; found: 328.2043.

$[\alpha]_D^{20}$ = +22.3° (c = 1.20, CH_2Cl_2).

Quinone 47

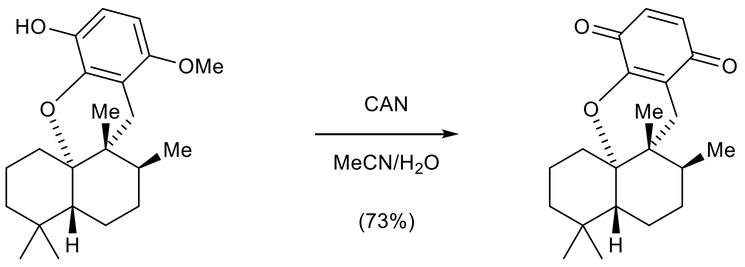

5-*epi*-strongylin A (**45**) **47**

A solution of diammonium cerium(IV) nitrate (35.8 mg, 65.3 µmol, 2.50 equiv) in water (3 mL) was added dropwise to a solution of *epi*-strongylin A (**45**) (9.00 mg, 26.0 µmol, 1 equiv) in acetonitrile (3 mL) over a period of 30 min at 0 °C. After 2 h, the bright yellow solution was diluted with water (10 mL) and the mixture was extracted with diethyl ether (3 × 10 mL). The combined organic extracts were dried over magnesium sulfate, the dried solution was filtered and the filtrate was concentrated. The residue was purified by flash-column chromatography on silica gel (5% ethyl acetate in hexanes) to yield **47** (6.30 mg, 73%) as a yellow solid.

TLC (20% ethyl acetate in hexanes): R_f = 0.50 (UV, CAM).

^{1}H NMR (599 MHz, $CDCl_3$): δ = 6.63 (d, *J* = 10.1 Hz, 1H), 6.58 (d, *J* = 10.1 Hz, 1H), 2.55 (d, *J* = 19.2 Hz, 1H), 1.99 (d, *J* = 19.2 Hz, 1H), 1.70–1.64 (m, 1H), 1.64–1.58 (m, 3H), 1.53–1.41 (m, 4H), 1.39–1.33 (m, 2H), 1.31–1.27 (m, 1H), 1.23–1.18 (m, 1H), 1.16 (s, 3H), 0.96 (s, 3H), 0.93 (s, 3H), 0.78 (d, *J* = 6.7 Hz, 3H).

^{13}C NMR (151 MHz, $CDCl_3$): δ = 187.2, 181.6, 152.1, 137.0, 134.2, 117.8, 86.2, 45.8, 41.8, 37.3, 33.6, 32.61, 32.60, 30.4, 29.4, 26.9, 22.3, 22.0, 18.0, 17.0, 16.5.

IR (Diamond-ATR, neat): $\tilde{\nu}_{max}$ = 2942, 1677, 1645, 1595, 1387, 1344, 1202, 1162, 1041, 898, 839, 731.

HRMS (EI) calcd for $C_{21}H_{28}O_3$: 328.2033 $[M]^+$; found: 328.2035.

$[\alpha]_D^{20}$ = –1.9° (c = 0.17, CH_2Cl_2).

Melting point: 138–143 °C

2-Hydroxy-3-iodo-4-methoxybenzaldehyde (S33)

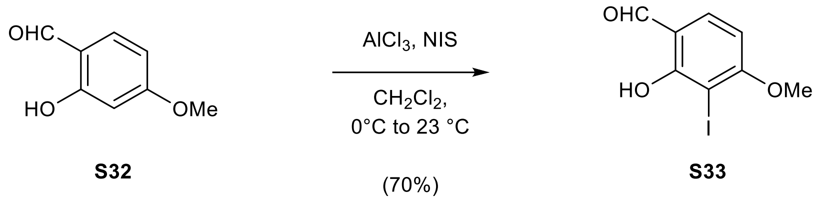

2-Hydroxy-3-iodo-4-methoxybenzaldehyde (**S33**) was synthesized according to a procedure described by U. Schilde:[24]

Aluminum trichloride (1.17 g, 8.81 mmol, 1 equiv) was added to a solution of aldehyde **S32** (1.34 g, 8.81 mmol, 1 equiv) in dichloromethane (40 mL) at –20 °C. After 15 min, *N*-iodosuccinimide (NIS) (2.18 g, 9.96 mmol, 1.10 equiv) was added to the bright orange solution and the reaction mixture was allowed to warm to 23 °C. After 13 h, aqueous hydrochloric acid solution (2 M, 100 mL) and dichloromethane (60 mL) were added. The layers were separated and the aqueous layer was extracted with dichloromethane (2 × 100 mL). The combined organic extracts were dried over magnesium sulfate, the dried solution was filtered and the filtrate was concentrated. The residue was purified by flash-column chromatography on silica gel (20% ethyl acetate in hexanes) to yield aldehyde **S33** (1.72 g, 70%) as a colorless solid. The obtained analytical data were in full agreement with those previously reported.

3-Iodo-4-methoxy-2-(methoxymethoxy)benzaldehyde (S34)

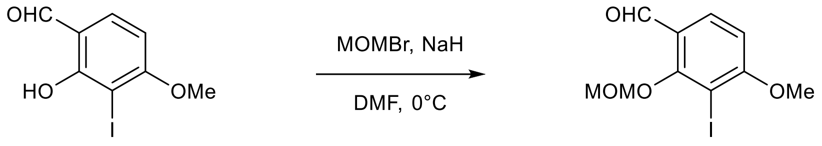

S33 **S34**

To a solution of **S33** (1.05 g, 3.78 mmol, 1 equiv) in *N*,*N*-dimethylformamide (40 mL) was added sodium hydride (60% mineral oil dispersion, 196 mg, 4.91 mmol, 1.30 equiv) at 0 °C. After 60 min, bromomethyl methylether (339 µL, 4.15 mmol, 1.10 equiv) was added dropwise to the dark brown suspension that became clear upon addition. After 1 h, saturated aqueous ammonium chloride solution (60 mL) was added and the mixture was extracted with diethyl ether (3 × 60 mL). The combined organic extracts were washed with saturated aqueous sodium chloride solution (100 mL). The washed solution was dried over sodium sulfate, the dried solution was filtered and filtrate was concentrated. The residue was purified by flash-column chromatography on silica gel (25% ethyl acetate in hexanes) to provide **S34** (1.08 g, 89%) as a colorless solid.

TLC (30% ethyl acetate in hexanes): R_f = 0.38 (UV, $KMnO_4$).

^{1}H NMR (599 MHz, $CDCl_3$): δ = 10.18 (d, *J* = 0.8 Hz, 1H), 7.88 (d, *J* = 8.7 Hz, 1H), 6.76 (dd, *J* = 8.7, 0.8 Hz, 1H), 5.18 (s, 2H), 3.98 (s, 3H), 3.63 (s, 3H).

^{13}C NMR (151 MHz, $CDCl_3$): δ = 189.1, 164.5, 162.2, 130.6, 125.1, 107.6, 101.7, 85.2, 58.6, 57.1.

IR (Diamond-ATR, neat): $\tilde{\nu}_{max}$ = 2940,1683, 1583, 1376, 1283, 1248, 1120, 1059, 962, 900.

HRMS (EI) calc. for $C_{10}H_{11}O_4{}^{127}I$ $[M]^+$: 321.9697; found: 321.9695.

Melting point: 67 °C.

2-Iodo-1-methoxy-3,4-bis(methoxymethoxy)benzene (S36)

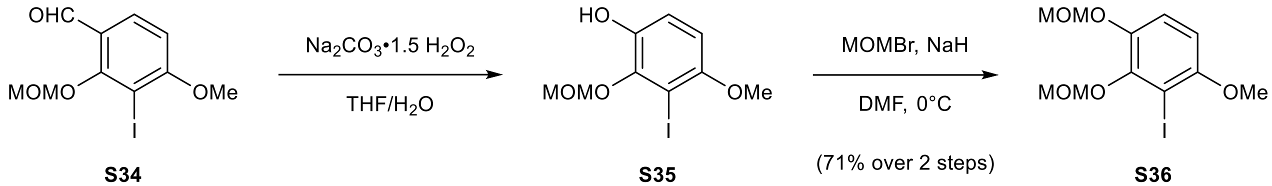

Sodium percarbonate (143mg, 914 µmol, 1 equiv) was added to a solution of **S34** (254 mg, 914 µmol, 1 equiv) in tetrahydrofuran (20 mL) and water (8 mL). After 1.5 h, saturated aqueous sodium thiosulfate solution (20 mL) and diethyl ether (20 mL) were added. The layers were separated and the aqueous layer was extracted with diethyl ether (2 × 20 mL). The combined organic extracts were washed with saturated aqueous sodium chloride solution (50 mL) and the washed solution was dried over magnesium sulfate. The dried solution was filtered and the filtrate was concentrated. The residue was directly used in the following reaction without further purification.

Sodium hydride (60% mineral oil dispersion, 84.0 mg, 2.10 mmol, 2.30 equiv) was added to a solution of crude **S35** (283 mg, 914 µmol, 1 equiv) in *N*,*N*-dimethyl formamide (5 mL) at 0 °C. After 1 h, bromomethyl methylether (153 µL, 1.87 µmol, 2.05 equiv) was added and the reaction mixture was allowed to warm to 23 °C. After 1 h, water (40 mL) and diethyl ether (40 mL) were added. The layers were separated and the aqueous layer was extracted with diethyl ether (2 × 30 mL). The combined organic extracts were washed with saturated aqueous sodium chloride solution (70 mL) and the washed solution was dried over magnesium sulfate. The dried solution was filtered and the filtrate was concentrated. The residue was purified by flash-column chromatography on silica gel (25% ethyl acetate in hexanes) to provide **S36** (147 mg, 71% over 2 steps) as a colorless oil.

TLC (20% ethyl acetate in hexanes): R_f = 0.23 (UV, CAM).

^{1}H NMR (400 MHz, $CDCl_3$): δ = 7.11 (d, *J* = 9.0 Hz, 1H), 6.55 (d, *J* = 9.0 Hz, 1H), 5.21 (s, 2H), 5.11 (s, 2H), 3.84 (s, 3H), 3.68 (s, 3H), 3.50 (s, 3H).

^{13}C NMR (101 MHz, $CDCl_3$): δ = 154.8, 148.4, 144.3, 118.2, 106.4, 99.2, 96.4, 85.7, 58.6, 57.0, 56.4.

IR (Diamond-ATR, neat): $\tilde{\nu}_{max}$ = 2954, 2900, 2833, 1477, 1436, 1251, 1154, 1069, 971, 923.

HRMS (EI) calc. for $C_{11}H_{15}{}^{127}IO_5$ $[M]^+$: 353.9959; found: 353.9960.

Olefin S37

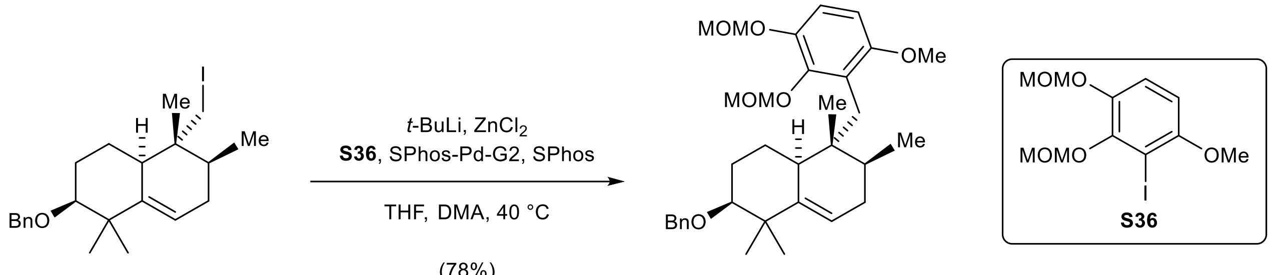

25 S37 S36

Note: Tetrahydrofuran was dried according to the procedure described by B. Williams prior to use.[13]

To a solution of alkyl iodide **25** (278 mg, 634 µmol, 1.50 equiv) and a solution of zinc chloride (1.00 M in tetrahydrofuran, 676 µL, 676 µmol, 1.6 equiv) in tetrahydrofuran (6 mL) was added dropwise a solution of *tert*-butyllithium (1.50 M in pentane, 902 µL, 1.35 mmol, 3.2 equiv) at –78 °C. After 50 min, the mixture was allowed to warm to 23 °C. After 20 min, the mixture was transferred into a mixture of aryl iodide **S36** (150 mg, 423 µmol, 1 equiv), SPhos (17.4 mg, 42.3 µmol, 0.100 equiv) and SPhos-Pd-G2 (30.5 mg, 42.3 µmol, 0.100 equiv) in tetrahydrofuran (3 mL) and *N*,*N*-dimethylacetamide (3 mL) and the reaction mixture was directly placed in a preheated oil bath (35 °C). diethyl ether (50 mL) and saturated aqueous ammonium chloride solution (50 mL) were added, the layers were separated and the aqueous layer was extracted with diethyl ether (3 × 50 mL). The combined organic extracts were washed with saturated aqueous sodium chloride solution (80 mL), the washed solution was dried over sodium sulfate, the dried solution was filtered and the filtrate was concentrated. The residue was purified by flash-column chromatography on silica gel (9% ethyl acetate in hexanes) to yield **S37** (178 mg, 78%) as a yellow oil.

TLC (20% ethyl acetate in hexanes): R_f = 0.60 (UV, CAM).

^{1}H NMR (599 MHz, $CDCl_3$): δ = 7.31–7.26 (m, 4H), 7.24–7.21 (m, 1H), 6.95 (d, *J* = 8.9 Hz, 1H), 6.53 (d, *J* = 8.9 Hz, 1H), 5.48–5.39 (m, 1H), 5.13–5.09 (m, 2H), 5.07 (s, 2H), 4.50 (d, *J* = 12.4 Hz, 1H), 4.26 (d, *J* = 12.4 Hz, 1H), 3.76 (s, 3H), 3.59 (s, 3H), 3.51 (s, 3H), 3.03 (d, *J* = 2.7 Hz, 1H), 2.94 (d, *J* = 12.9 Hz, 1H), 2.68–2.62 (m, 2H), 1.94–1.82 (m, 2H), 1.65–1.59 (m, 1H), 1.56–1.51 (m, 1H), 1.50–1.44 (m, 1H), 1.23–1.14 (m, 1H), 1.08 (s, 3H), 1.03 (d, *J* = 6.8 Hz, 3H), 0.99 (s, 3H), 0.91–0.87 (m, 1H), 0.79 (s, 3H).

^{13}C NMR (151 MHz, $CDCl_3$): δ = 154.5, 148.0, 144.8, 143.8, 139.6, 128.2, 127.9, 127.2, 124.9, 117.5, 115.5, 105.4, 99.3, 96.4, 82.9, 70.2, 57.9, 56.4, 55.5, 41.7, 41.3, 40.9, 39.1, 35.4, 32.1, 28.6, 25.8, 24.5, 23.8, 16.4, 14.2.

IR (Diamond-ATR, neat): $\tilde{\nu}_{max}$ = 2950, 1483, 1252, 1154, 1068, 1038, 984, 951, 736, 698.

HRMS (EI) calc. for $C_{33}H_{46}O_{cc}$ $[M]^+$: 538.3289; found: 538.3303.

$[\alpha]_D^{20}$ = +18.1° (*c* = 0.85, CH_2Cl_2).

Phenol S39

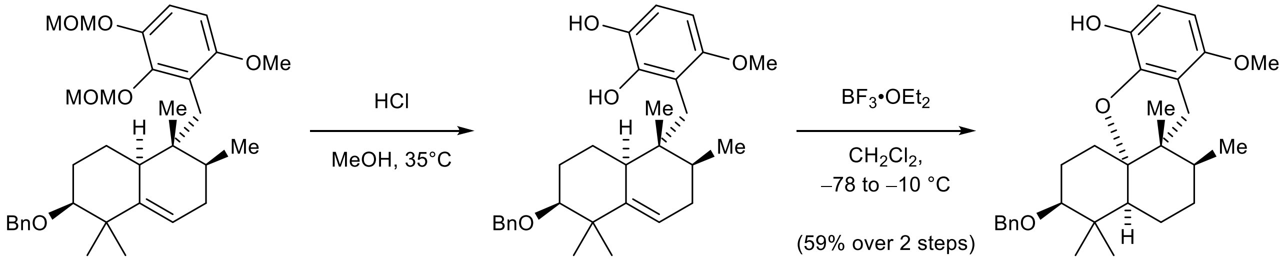

A solution of hydrochloric acid (~1.25 M in methanol, 12 mL) was added to a solution of **S37** (145 mg, 269 µmol, 1 equiv) in dichloromethane (6 mL) and the resulting solution was heated to 35 °C. After 1 h, the reaction mixture was diluted with dichloromethane (25 mL) and saturated aqueous sodium bicarbonate solution (25 mL) was added. The layers were separated and the aqueous layer was extracted with dichloromethane (3 × 30 mL). The combined organic extracts were dried over magnesium sulfate, the dried solution was filtered and the filtrate was concentrated to yield **S38** as a yellow oil that was directly used in the following reaction without further purification.

A solution of boron trifluoride diethyl etherate (48% in diethyl ether, 707 µL, 2.69 mmol, 10.0 equiv) was added dropwise to a solution of the crude **S38** (83.4 mg, 269 µmol, 1 equiv) in dichloromethane (35 mL) at –50 °C. After 15 min, the reaction mixture was allowed to warm to –15 °C. After 30 min, saturated aqueous sodium bicarbonate solution (50 mL) was added, the layers were separated and the aqueous layer was extracted with dichloromethane (3 × 50 mL). The combined organic extracts were dried over magnesium sulfate, the dried solution was filtered and the filtrate was concentrated. The residue was purified by flash-column chromatography on silica gel (hexanes with 5% ethyl acetate) to yield **S39** (71.4 mg, 59% over 2 steps) as a colorless, highly viscous oil.

TLC (20% ethyl acetate): R_f = 0.61 (UV, CAM).

^{1}H NMR (400 MHz, $CDCl_3$): δ = 7.40–7.33 (m, 4H), 7.32–7.26 (m, 1H), 6.69 (d, *J* = 8.6 Hz, 1H), 6.27 (d, *J* = 8.6 Hz, 1H), 4.94 (s, 1H), 4.66 (d, *J* = 12.0 Hz, 1H), 4.40 (d, *J* = 12.0 Hz, 1H), 3.78 (s, 3H), 3.23–3.11 (m, 2H), 2.34–2.21 (m, 2H), 2.20–2.10 (m, 2H), 2.09–1.99 (m, 1H), 1.96–1.88 (m, 1H), 1.82–1.71 (m, 3H), 1.63–1.56 (m, 1H), 1.35–1.29 (m, 1H), 1.13 (d, *J* = 7.5 Hz, 3H), 1.03 (s, 3H), 1.00 (s, 3H), 0.92 (s, 3H).

^{13}C NMR (101 MHz, $CDCl_3$): δ = 151.0, 139.8, 139.2, 138.6, 128.3, 127.2, 127.1, 110.7, 110.7, 100.3, 84.4, 82.4, 71.8, 55.5, 44.4, 39.7, 38.4, 38.1, 32.0, 30.2, 28.0, 27.2, 24.4, 23.8, 21.4, 20.3, 17.3.

IR (Diamond-ATR, neat): $\tilde{\nu}_{max}$ = 3564, 2938, 2874, 1490, 1384, 1264, 1185, 1085, 908, 732.

HRMS (EI) calc. for $C_{29}H_{38}O_4$ $[M]^+$: 4 50.2765; found: 450.2762.

$[\alpha]_D^{20}$ = +73.6° (*c* = 1.48, CH_2Cl_2).

3-Hydroxy-strongylin A (48)

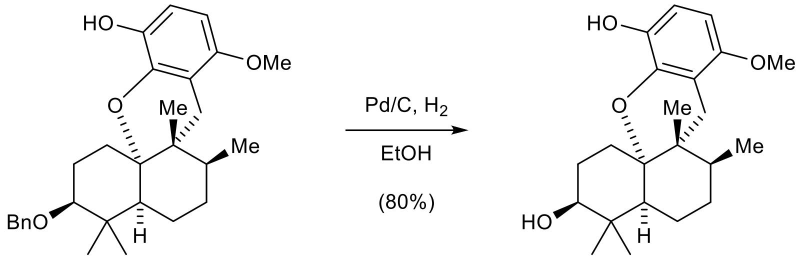

S39 **48**

A solution of **S39** (62.0 mg, 138 µmol, 1 equiv) in ethanol (5 mL) was treated with palladium on carbon (10 wt.%, 146 mg, 138 µmol, 1 equiv) at 23 °C. An atmosphere of hydrogen was maintained by sparging with a stream of pure hydrogen gas through a stainless steel needle for 2 min and vigorous stirring of the suspension was then continued under hydrogen atmosphere at 23 °C. After 14 h, the reaction mixture was filtered through a pad of Celite®. The filtercake was thoroughly rinsed with dichloromethane (40 mL). The filtrate was concentrated and the residue was purified by flash-column chromatography on silica gel (20% ethyl acetate in hexanes) to give 3-hydroxy-strongylin A (**47**) (40.0 mg, 80%) as a colorless solid. Recrystallization from ethyl acetate gave crystals suitable for single-crystal X-ray diffraction.

TLC (30% ethyl acetate in hexanes): R_f = 0.27 (UV, CAM).

^{1}H NMR (400 MHz, C_6D_6): δ = 6.95 (d, *J* = 8.6 Hz, 1H), 6.12 (d, *J* = 8.6 Hz, 1H), 4.99 (s, 1H), 3.41 (s, 3H), 3.28 (d, *J* = 17.5 Hz, 1H), 3.18–3.12 (m, 1H), 2.35 (d, *J* = 17.5 Hz, 1H), 2.24–2.04 (m, 3H), 1.90–1.78 (m, 1H), 1.62–1.50 (m, 3H), 1.50–1.42 (m, 1H), 1.37–1.27 (m, 1H), 1.19–1.10 (m, 1H), 1.00–0.95 (m, 6H), 0.87 (s, 3H), 0.76 (s, 1H), 0.74 (s, 3H).

^{13}C NMR (101 MHz, C_6D_6): δ = 151.4, 139.6, 139.5, 111.5, 110.9, 100.8, 84.2, 74.3, 55.0, 44.2, 40.0, 38.2, 37.8, 32.7, 30.0, 28.3, 26.7, 26.5, 24.3, 24.1, 20.4, 17.2.

IR (Diamond-ATR, neat): $\tilde{\nu}_{max}$ = 3472, 3157, 2986, 2963, 2874, 1605, 1464, 1265, 1096, 979.

HRMS (EI) calc. for $C_{22}H_{32}O_4$ $[M]^+$: 360.2295; found: 360.2302.

$[\boldsymbol{\alpha}]_{\boldsymbol{D}}^{\mathbf{20}}$ = +66.8° (*c* = 0.79, CH_2Cl_2).

Melting point: 187 °C.

Quinone 49

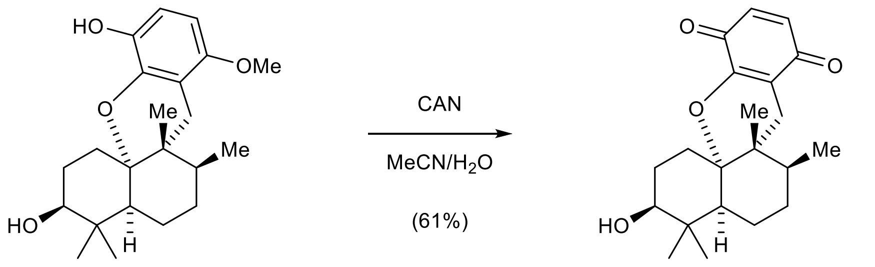

47 49

A solution of diammonium cerium(IV) nitrate (35.7 mg, 65.2 µmol, 2.50 equiv) in water (2.5 mL) was added dropwise to a solution of 3-hydroxy-strongylin A (**47**) (9.40 mg, 26.1 µmol, 1 equiv) in acetonitrile (2.5 mL) over a period of 1 h at 0 °C. After 2 h, the bright yellow solution was diluted with water (10 mL) and the mixture was extracted with diethyl ether (3 × 10 mL). The combined organic extracts were dried over magnesium sulfate, the dried solution was filtered and the filtrate was concentrated. The residue was purified by flash-column chromatography on silica gel (10% ethyl acetate in hexanes) to yield **48** (5.50 mg, 61%) as a yellow oil.

TLC (30% ethyl acetate in hexanes): R_f = 0.37 (UV, CAM).

^{1}H NMR (400 MHz, $CDCl_3$): δ = 6.65 (d, *J* = 10.1 Hz, 1H), 6.57 (d, *J* = 10.1 Hz, 1H), 3.56 (s, 1H), 2.87 (d, *J* = 19.0 Hz, 1H), 2.62–2.50 (m, 1H), 2.25 (td, *J* = 13.6, 3.9 Hz, 1H), 2.08 (td, *J* = 13.6, 4.9 Hz, 1H), 2.01–1.86 (m, 2H), 1.82–1.72 (m, 3H), 1.71–1.63 (m, 1H), 1.61–1.50 (m, 1H), 1.49–1.42 (m, 1H), 1.37–1.30 (m, 1H), 1.11 (d, *J* = 7.4 Hz, 3H), 0.99 (s, 3H), 0.96 (s, 3H), 0.90 (s, 3H).

^{13}C NMR (101 MHz, $CDCl_3$): δ = 187.3, 181.6, 150.3, 137.1, 133.9, 118.1, 87.6, 74.3, 45.3, 39.3, 38.1, 38.1, 30.7, 30.4, 28.2, 26.6, 26.0, 24.3, 23.6, 20.4, 17.1.

IR (Diamond-ATR, neat): $\tilde{\nu}_{max}$ = 3540, 2928, 2876, 1675, 1645, 1596, 1394, 1193, 1050, 970.

HRMS (EI) calcd for $C_{21}H_{28}O_4$ $[M]^+$: 344.1982; found: 344.1981.

$[\alpha]_D^{20}$ = +112.0° (c = 0.10, $CHCl_3$).

Synthesis of 5-*epi*-Cyclosmenospongine (49), (+)-Smenoqualone (3) & 5-*epi*-Smenoqualone (50)

Bromide S42

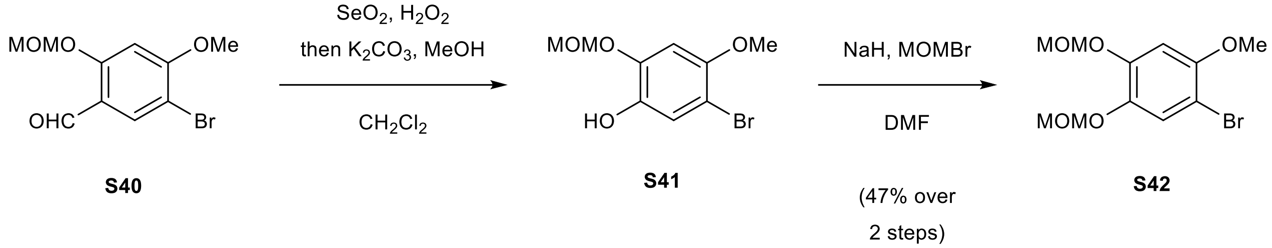

Benzaldehyde **S40**[24,25] (1.48 g, 5.38 mmol, 1 equiv) was added to a solution of selenium dioxide (47.8 mg, 430 µmol, 8.0 mol%) and hydrogen peroxide (30% in water, 1.21 mL, 11.8 mmol, 2.20 equiv) in dichloromethane (30 mL). After 14 h, saturated aqueous ammonium chloride solution (40 mL) was added, the layers were separated and the aqueous layer was extracted with dichloromethane (3 × 30 mL). The combined organic extracts were dried over magnesium sulfate, the dried solution was filtered and the filtrate was concentrated. The residue was dissolved in methanol (40 mL) and an aqueous solution of potassium carbonate (14%, 10 mL) was added. After 1 h, the reaction mixture was extracted with dichloromethane (3 × 30 mL), the combined organic extracts were dried over magnesium sulfate, the dried solution was filtered and the filtrate was concentrated to give crude **S41** (924 mg) as a yellow oil that was used without further purification.

To a solution of crude **S41** (924 mg, 3.51 mmol, 1 equiv) in *N,N*-dimethylformamide (12 mL) was added sodium hydride (60% mineral oil dispersion, 211 g, 5.27 mmol, 1.50 equiv) at 0 °C. After 1 h, bromomethyl methyl ether (344 µL, 4.21 mmol, 1.20 equiv) was added and the reaction mixture was allowed to warm to 23 °C. After 1.5 h, water (20 mL) was added and the mixture was extracted with diethyl ether (3 × 20 mL). The combined organic extracts were washed with saturated aqueous sodium chloride solution (40 mL). The washed solution was dried over sodium sulfate, the dried solution was filtered and the filtrate was concentrated. The residue was purified by flash-column chromatography on silica gel (20 ethyl acetate in hexanes) to provide **S42** (510 mg, 48% over 2 steps) as a yellow oil. The obtained analytical data were in full agreement with those previously reported.[26]

Arene S43

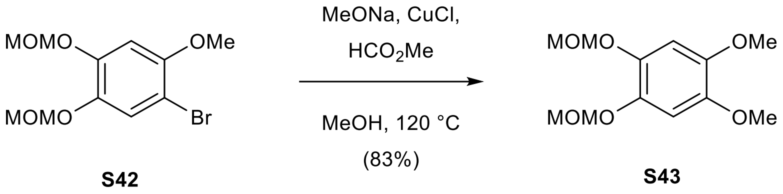

A mixture of aryl bromide **S42** (503 mg, 1.64 mmol, 1 equiv), sodium methoxide (177 mg, 3.28 mmol, 2.00 equiv), copper(I) chloride (6.49 mg, 65.5 µmol, 0.04 equiv) and formic acid methyl ester (40.6 µL, 65.5 µmol, 0.40 equiv) in methanol (1 mL) was heated to 120 °C in a pressure tube. After 13 h, the reaction mixture was cooled to 23 °C, dichloromethane (10 mL) and saturated aqueous ammonium chloride solution (10 mL) were added. The layers were separated and the aqueous layer was extracted with dichloromethane (2 × 10 mL). The combined organic extracts were dried over sodium sulfate, the dried solution was filtered and the filtrate was concentrated. The residue was purified by flash-column chromatography on silica gel (20% ethyl acetate in hexanes initially, grading to 50% ethyl acetate in hexanes) to provide **S43** (350 mg, 83%) as a colorless oil.

TLC (20% ethyl acetate in hexanes): R_f = 0.22 (UV, CAM).

^{1}H NMR (400 MHz, $CDCl_3$): δ = 6.79 (s, 2H), 5.14 (s, 4H), 3.83 (s, 6H), 3.53 (s, 6H).

^{13}C NMR (101 MHz, $CDCl_3$): δ = 144.6, 141.2, 104.2, 96.9, 56.7, 56.4.

IR (Diamond-ATR, neat): $\tilde{\nu}_{max}$ = 2937, 2830, 1512, 1465, 1384, 1214, 1191, 1151, 1009, 908, 729.

HRMS (EI) calcd for $C_{12}H_{18}O_6$ $[M]^+$: 258.1098; found: 258.1098.

Benzylic alcohol (S44)

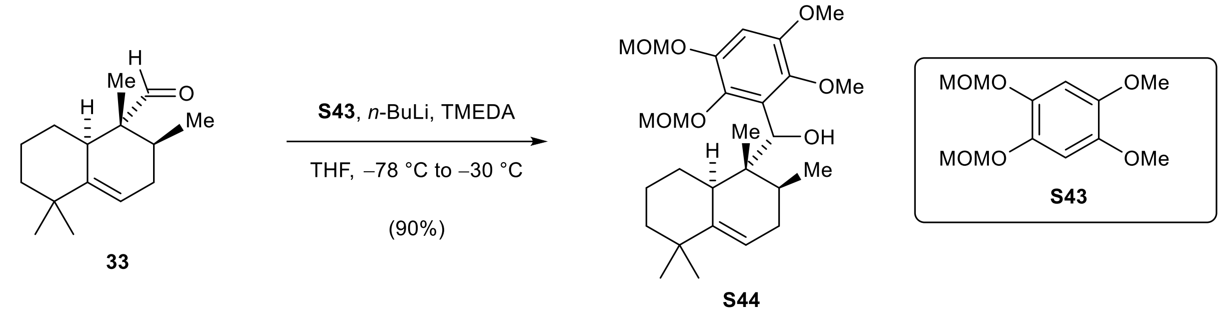

To a solution of **S43** (327 mg, 1.27 mmol, 1.40 equiv) in tetrahydrofuran (6 mL) and freshly distilled *N*,*N*,*N′*,*N′*-tetramethylethane-1,2-diamine (over CaH_2, 409 µL, 2.71 mmol, 3.00 equiv) was added a solution of *n*-butyllithium (2.44 M in hexanes 0.45 mL, 1.13 mmol, 1.25 equiv) at –78 °C. The reaction mixture was allowed to warm to –60 °C. After 1.5 h, the yellow suspension was cooled to –78 °C and a solution of aldehyde **33** (199 mg, 904 µmol, 1 equiv) in tetrahydrofuran (4 mL) was added to give a clear yellow solution. The reaction mixture was warmed to –30 °C over a period of 2 h. Diethyl ether (30 mL) and saturated aqueous ammonium chloride solution (40 mL) were added. The layers were separated, the aqueous layer was extracted with diethyl ether (3 × 30 mL) and the combined organic extracts were washed with saturated aqueous sodium chloride solution (50 mL). The washed solution was dried over sodium sulfate and the dried solution was filtered. The filtrate was concentrated and the residue was purified by flash-column chromatography on silica gel (20% ethyl acetate in hexanes) to yield **S44** (391 mg, 90%) as a colorless oil. The mixture of inseparable diastereoisomers was partially characterized by HRMS and IR spectroscopy.

TLC (30% ethyl acetate in hexanes): R_f = 0.35 (CAM).

IR (Diamond-ATR, neat): $\tilde{\nu}_{max}$ = 3559, 2928, 1592, 1482, 1435, 1227, 1153, 1035, 948.

HRMS (EI) calcd for $C_{27}H_{42}O_7$ $[M]^+$: 478.2925; found: 478.2925.

Olefin S45

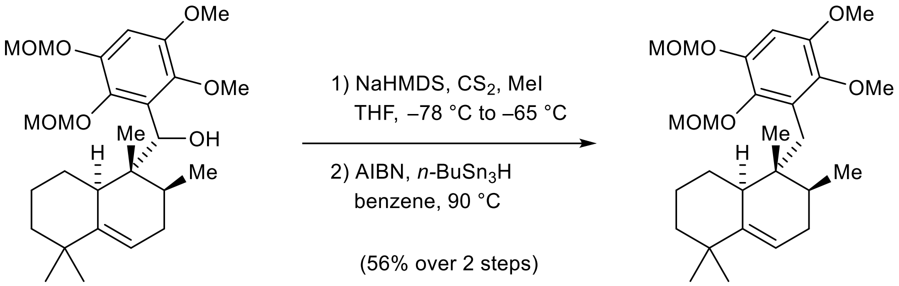

S44 **S45**

A solution of sodium bis(trimethylsilyl)amide (1 M in tetrahydrofuran, 3.90 mL, 3.90 mmol, 5.00 equiv) was added dropwise to a solution of **S44** (373 mg, 779 µmol, 1 equiv) in tetrahydrofuran (10 mL) at −78 °C. After 30 min, carbon disulfide (940 µL, 15.6 mmol, 20.0 equiv) was slowly added and the reaction mixture was allowed to warm to −65 °C. After 1 h, methyl iodide (970 µL, 15.6 mmol, 20.0 equiv) was slowly added to the reaction mixture. After 1 h, saturated aqueous ammonium chloride solution (25 mL) and ethyl acetate (30 mL) were added, the layers were separated and the aqueous layer was extracted with ethyl acetate (3 × 30 mL). The combined organic extracts were dried over sodium sulfate, the dried solution was filtered and the filtrate was concentrated. The residue was filtered through a short plug of silica and the obtained crude xanthogenate was used without further purification.

Note: benzene was degassed via freeze-pump-thaw (three cycles) prior to use.
A degassed solution of the xanthogenate (420 mg, 779 µmol, 1 equiv), 2,2'-azobis(2-methylpropionitrile) (AIBN) (64.0 mg, 390 µmol, 0.50 equiv) and tributyltin hydride (1.04 mL, 3.90 mmol, 5.00 equiv) in benzene (25 mL) was heated to 90 °C. After 7.5 h, the reaction mixture was cooled to 23 °C and directly purified by flash-column chromatography on silica gel (3% ethyl acetate in hexanes) to yield **S45** as a colorless oil (202 mg, 56% over 2 steps).

TLC (20% ethyl acetate in hexanes): R_f = 0.34 (CAM).

^{1}H NMR (800 MHz, $CDCl_3$): δ = 6.66 (s, 1H), 5.41–5.36 (m, 1H), 5.17–5.12 (m, 2H), 5.02–4.98 (m, 2H), 3.83 (s, 3H), 3.72 (s, 3H), 3.59 (s, 3H), 3.52 (s, 3H), 2.85 (d, *J* = 12.8 Hz, 1H), 2.60 (d, *J* = 12.8 Hz, 1H), 2.59–2.55 (m, 1H), 1.97–1.90 (m, 1H), 1.84–1.78 (m, 1H), 1.56–1.52 (m, 1H), 1.44–1.37 (m, 1H), 1.34–1.28 (m, 2H), 1.19–1.14 (m, 1H), 1.11–1.06 (m, 1H), 1.03–0.99 (m, 9H), 0.75 (s, 3H), 0.72–0.66 (m, 1H).

^{13}C NMR (201 MHz, $CDCl_3$): δ = 149.2, 147.7, 145.8, 143.8, 140.9, 129.8, 114.7, 101.6, 99.5, 96.5, 60.4, 57.8, 56.5, 56.3, 42.1, 41.7, 40.9, 38.9, 36.7, 36.2, 31.8, 31.1, 30.0, 28.1, 23.0, 16.6, 14.8.

IR (Diamond-ATR, neat): $\tilde{\nu}_{max}$ = 2973, 2840, 1593, 1483, 1338, 1239, 1154, 1049, 956, 731.

HRMS (EI) calcd for $C_{27}H_{42}O_6$ $[M]^+$: 462.2976; found: 462.2972.

$[\alpha]_D^{20}$ = +24.3° (c = 0.41, CH_2Cl_2).

Phenol S47

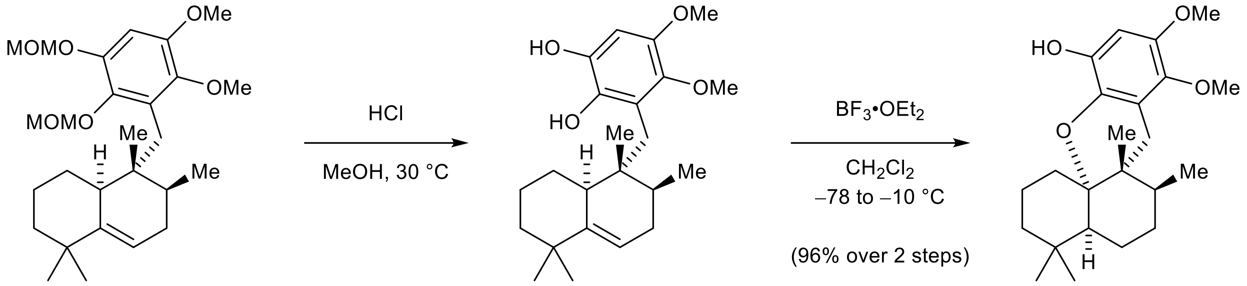

S45 **S46** **S47**

A solution of hydrochloric acid (~1.25 M in methanol, 3 mL) was added to a solution of **S45** (24.4 mg, 52.7 µmol, 1 equiv) in dichloromethane (1 mL) and the resulting solution was heated to 30 °C. After 4 h, the reaction mixture was diluted with dichloromethane (5 mL) and saturated aqueous sodium bicarbonate solution (3 mL) was added. The layers were separated and the aqueous layer was extracted with dichloromethane (3 × 5 mL). The combined organic extracts were dried over sodium sulfate, the dried solution was filtered and the filtrate was concentrated to yield **S46** as a yellow foam that was directly used in the following step.

A solution of boron trifluoride diethyl etherate (48% in diethyl ether, 138 µL, 527 µmol, 10.0 equiv) was added dropwise to a solution of the crude hydroquinone **S46** (19.7 mg, 52.7 µmol, 1 equiv) in dichloromethane (3 mL) at –78 °C. After complete addition, the reaction mixture was allowed to warm to –10 °C over a period of 30 min. After 1.5 h, saturated aqueous ammonium chloride solution (5 mL) and dichloromethane (5 mL) were added. The layers were separated and the aqueous layer was extracted with dichloromethane (3 × 5 mL). The combined organic extracts were dried over sodium sulfate, the dried solution was filtered and the filtrate was concentrated. The residue was purified by flash-column chromatography on silica gel (20% ethyl acetate in hexanes) to yield **S47** (19.0 mg, 96% over two steps) as an amorphous solid.

TLC (20% ethyl acetate in hexanes): R_f = 0.58 (UV, $KMnO_4$).

^{1}H NMR (400 MHz, C_6D_6): δ = 6.64 (s, 1H), 5.26 (s, 1H), 3.78 (s, 3H), 3.37 (s, 3H), 3.28 (d, J = 17.6 Hz, 1H), 2.26 (d, J = 17.6 Hz, 1H), 1.91–1.75 (m, 2H), 1.67–1.58 (m, 2H), 1.54–1.48 (m, 1H), 1.46–1.39 (m, 2H), 1.31–1.21 (m, 3H), 1.12 (s, 4H), 1.09–1.05 (m, 1H), 0.89 (d, J = 7.5 Hz, 3H), 0.82 (s, 3H), 0.67 (s, 3H).

^{13}C NMR (101 MHz, C_6D_6): δ = 146.3, 140.9, 140.5, 132.8, 116.1, 99.9, 83.4, 60.1, 56.2, 44.1, 39.7, 38.4, 34.1, 33.6, 33.1, 32.2, 29.5, 29.3, 27.9, 22.7, 20.2, 18.9, 17.4.

IR (Diamond-ATR, neat): $\tilde{\nu}_{max}$ = 2934, 2279, 1618, 1492, 1329, 1259, 1188, 1049, 811.

HRMS (EI) calcd for $C_{23}H_{34}O_4$ $[M]^+$: 374.2452; found: 374.2449.

$[\alpha]_D^{20}$ = +45.8° (c = 1.46, CH_2Cl_2).

(+)-Smenoqualone (3)

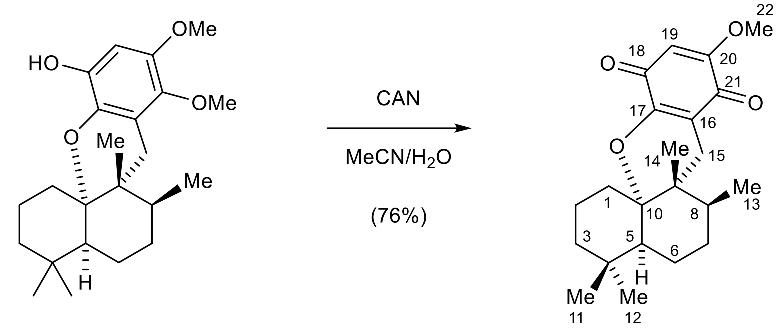

S47 (+)-smenoqualone (**3**)

A solution of ammonium cerium(IV) nitrate (55.1 mg, 100 µmol, 2.20 equiv) in water (3 mL) was added dropwise over a period of 40 min to a solution of phenol **S47** (17.1 mg, 45.7 µmol, 1 equiv) in acetonitrile (3 mL) at 0 °C. After 3 h, the bright yellow solution was diluted with water (5 mL) and diethyl ether (5 mL). The layers were separated and the aqueous layer was extracted with diethyl ether (3 × 5 mL). The combined organic extracts were washed with saturated aqueous sodium chloride solution (20 mL). The washed solution was dried over sodium sulfate, the dried solution was filtered and the filtrate was concentrated. The residue was purified by flash-column chromatography on silica gel (10% diethyl ether in pentane initially, grading to 30% diethyl ether in pentane) to provide (+)-smenoqualone (**3**) (12.4 mg, 76%) as a bright yellow foam.

TLC (30% ethyl acetate in hexanes): R_f = 0.35 (UV, CAM).

^{1}H NMR (800 MHz, $CDCl_3$): δ = 5.73 (s, 1H, H-19), 3.81 (s, 3H, H-3), 2.85 (d, $^2J_{15A/15B}$ = 18.3 Hz, 1H, H-15$_A$), 2.15–2.07 (m, 1H, H-2$_A$), 2.04–1.98 (m, 1H, H-7$_A$), 1.95 (d, $^2J_{15B/15A}$ = 18.3 Hz, 1H, H-15$_B$), 1.89–1.85 (m, 1H, H-1$_A$), 1.84–1.79 (m, 1H, H-1$_B$), 1.79–1.76 (m, 1H, H-8), 1.76–1.72 (m, 1H, H-6$_A$), 1.59–1.55 (m, 1H, H-6$_B$), 1.54–1.50 (m, 1H, H-2$_B$), 1.42–1.35 (m, 3H, H-3$_A$), 1.25–1.22 (m, 1H, H-3$_B$), 1.09 (d, $^3J_{13/8}$ = 7.6 Hz, 3H, H-13), 1.00 (s, 3H, H-12), 0.85 (s, 3H, H-14), 0.82 (s, 3H, H-11).

^{13}C NMR (201 MHz, $CDCl_3$): δ = 181.53 (C-21), 181.47 (C-18), 159.5 (C-20), 151.1 (C-17), 115.2 (C-16), 104.6 (C-19), 87.8 (C-10), 56.3 (C-22), 45.1 (C-5), 39.0 (C-8), 38.0 (C-9), 33.7 (C-4), 33.4 (C-3), 31.9 (C-11), 30.7 (C-15), 29.7 (C-12), 28.9 (C-1), 27.7 (C-7), 22.4 (C-6), 20.1 (C-14), 18.3 (C-2), 17.1 (C-13).

IR (Diamond-ATR, neat): $\tilde{\nu}_{max}$ = 2938, 1665, 1646, 1602, 1457, 1342, 1276, 1220, 1037, 842.

HRMS (EI) calcd for $C_{22}H_{30}O_4$ $[M]^+$: 358.2139; found: 358.2139.

$[\alpha]_D^{20}$ = +84.3° (c 0.37, $CHCl_3$); $[\alpha]_D^{n.a.}$ = +70° (c = 0.001, $CHCl_3$) for (+)-smenoqualone.[27] n.a.: Temperature not available

Supplementary Table 8 Comparison of ^{1}H NMR data for natural and synthetic (+)-smenoqualone (**3**).

**Proton**	**Synthetic (800 MHz, $CDCl_3$)**	**Natural**	**Δ: δ (ppm)**
1A	1.89–1.85 (m, 1H)		
1B	1.84–1.79 (m, 1H)		
2A	2.15–2.07 (m, 1H)		
2B	1.54–1.50 (m, 1H)		
3A	1.42–1.35 (m, 3H)		
3B	1.25–1.22 (m, 1H)		
5	1.42–1.35 (m, 3H)		
6A	1.76–1.72 (m, 1H)		
6B	1.59–1.55 (m, 1H)		
7A	2.04–1.98 (m, 1H)		
7B	1.42–1.35 (m, 3H)		
8	1.79–1.76 (m, 1H)		
11	0.82 (s, 3H).		
12	1.00 (s, 3H)		
13	1.09 (d, *J* = 7.6 Hz, 3H)		
14	0.85 (s, 3H)		
15A	2.85 (d, *J* = 18.3 Hz, 1H)		
15B	1.95 (d, *J* = 18.3 Hz, 1H)		
19	5.73 (s, 1H)		
22	3.81 (s, 3H)		

Supplementary Table 9 Comparison of ^{13}C NMR data for natural and synthetic (+)-smenoqualone (**3**).

Carbon	Synthetic (201 MHz, $CDCl_3$)	Natural (75 MHz, $CDCl_3$)[27]	Δ: δ (ppm)
1	28.9	28.9	± 0.0
2	18.3	18.3	± 0.0
3	33.4	33.4	± 0.0
4	33.7	33.7	± 0.0
5	45.1	45.1	± 0.0
6	22.4	22.5	– 0.1
7	27.7	27.7	± 0.0
8	39.0	39.0	± 0.0
9	38.0	37.9	+ 0.1
10	87.8	87.8	± 0.0
11	31.9	31.9	± 0.0
12	29.7	29.7	± 0.0
13	17.1	17.1	± 0.0
14	20.1	20.1	± 0.0
15	30.7	30.7	± 0.0
16	115.2	115.3	– 0.1
17	151.1	151.1	± 0.0
18	181.5	181.5	± 0.0
19	104.6	104.7	– 0.1
20	159.5	159.5	± 0.0
21	181.5	181.5	+0.0
22	56.3	56.3	± 0.0

5-*Epi*-cyclosmenospongine (49)

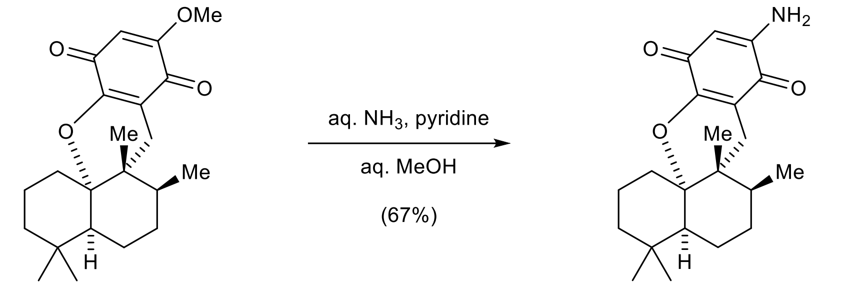

(+)-smenoqualone (**3**)

5-*epi*-cyclosmenospongine (**50**)

To a solution of (+)-smenoqualone (**3**) (6.70 mg, 18.7 µmol, 1 equiv) and pyridine (600 µL) in aqueous methanol (50%, 6 mL) was added aqueous ammonia (25%, 600 µL) at 23 °C. After 14 h, the reaction mixture was concentrated, water (5 mL) was added and the aqueous layer was extracted with diethyl ether (4 × 10 mL). The combined organic extracts were dried over sodium sulfate, the dried solution was filtered and the filtrate was concentrated. The residue was purified by flash-column chromatography on Sephadex® LH-20 (9% ethanol in chloroform) to yield 5-*epi*-cyclosmenospongine (**50**) (4.30 mg, 67%) as a dark red oil.

TLC (30% ethyl acetate in hexanes): R_f = 0.19 (UV, $KMnO_4$).

^{1}H NMR (800 MHz, $CDCl_3$): δ = 5.53 (s, 1H), 5.10 (s, 2H), 2.83 (d, *J* = 18.1 Hz, 1H), 2.18–2.09 (m, 1H), 2.05–1.98 (m, 1H), 1.92–1.86 (m, 2H), 1.85–1.79 (m, 1H), 1.78–1.71 (m, 2H), 1.57–1.55 (m, 1H), 1.53–1.49 (m, 1H), 1.44–1.39 (m, 2H), 1.39–1.35 (m, 1H), 1.26–1.22 (m, 1H), 1.09 (d, *J* = 7.6 Hz, 3H), 1.03 (s, 3H), 0.87 (s, 3H), 0.82 (s, 3H).

^{13}C NMR (201 MHz, $CDCl_3$): δ = 182.8, 180.3, 153.2, 147.7, 113.0, 99.2, 88.0, 45.3, 39.2, 38.1, 33.9, 33.6, 32.1, 30.7, 29.9, 29.1, 27.9, 22.6, 20.2, 18.5, 17.3.

IR (Diamond-ATR, neat): $\tilde{v}_{max}$ = 3448, 3338, 1936, 2873, 2360, 2340, 1593, 1373, 1226, 943.

HRMS (EI) calcd for $C_{21}H_{29}NO_3$ $[M]^+$: 343.2142; found: 343.2135.

$[\alpha]_D^{20}$ = +492.3° (c = 0.16, CH_2Cl_2).

5-E*pi*-smenoqualone (50)

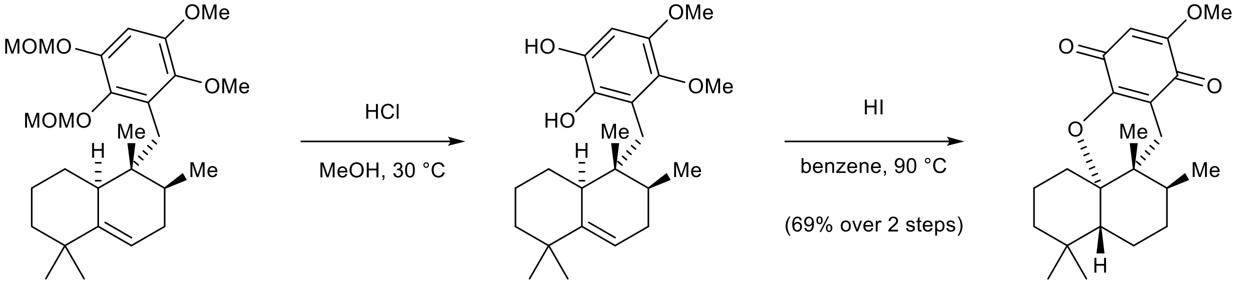

S45 **S46** 5-*epi*-smenoqualone (**51**)

A solution of hydrochloric acid (~1.25 M in methanol, 4 mL) was added to a solution of **S45** (64.0 mg, 138 µmol, 1 equiv) in dichloromethane (2 mL) and the mixture was heated to 40 °C. After 2 h, the reaction mixture was diluted with dichloromethane (20 mL) and saturated aqueous sodium bicarbonate solution (30 mL) was added. The layers were separated and the aqueous layer was extracted with dichloromethane (2 × 20 mL). The combined organic extracts were dried over sodium sulfate. The dried solution was filtered and the filtrate was concentrated to yield **S46** as a yellow oil that was directly used in the following reaction.

A solution of hydroiodic acid (57 wt.% in H_2O, 183 µL, 1.38 mmol, 10.0 equiv) was added to a solution of crude **S46** (51.7 mg, 138 µmol, 1 equiv) in benzene (5 mL) in an Ace® pressure tube. The tube was sealed and the reaction mixture was heated to 90 °C. After 20 h, the reaction mixture was cooled to 23 °C, saturated aqueous sodium bicarbonate chloride solution (15 mL) and dichloromethane (10 mL) were added. The layers were separated and the aqueous layer was extracted with dichloromethane (3 × 15 mL). The combined organic extracts were dried over sodium sulfate, the dried solution was filtered and the filtrate was concentrated. The residue was purified by flash-column chromatography on silica gel (20% ethyl acetate in hexanes) to yield 5-*epi*-smenoqualone (**51**) (34.2 mg, 69% over two steps) as a bright yellow solid. The obtained analytical data were in full agreement with those values previously reported by our group.[21]

Synthetic Analogues 40, 41, 42, 43 and 44

Olefin S48

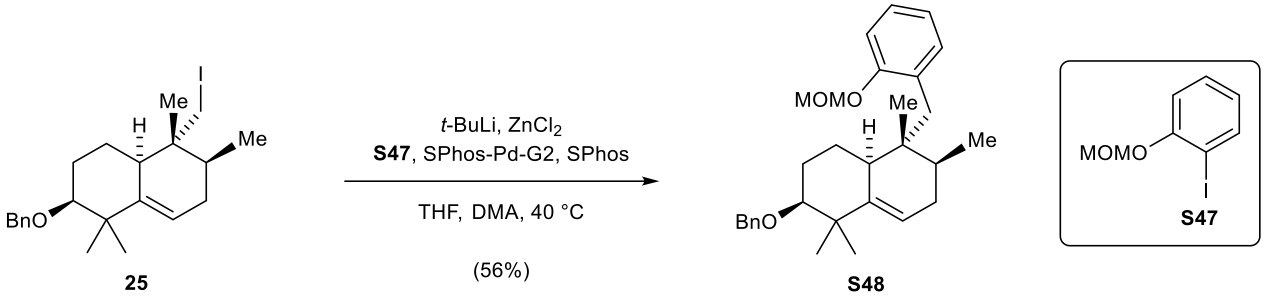

Note: Tetrahydrofuran was dried according to the procedure described by B. Williams prior to use.[13]

To a mixture of alkyl iodide **25** (120 mg, 274 µmol, 1.50 equiv) and a solution of zinc chloride (1.00 M in tetrahydrofuran, 292 µL, 292 µmol, 1.6 equiv) in tetrahydrofuran (3 mL) was added dropwise a solution of *tert*-butyllithium (1.50 M in pentane, 389 µL, 584 µmol, 3.20 equiv) at –78 °C. After 50 min, the mixture was allowed to warm to 23 °C. After 20 min, the mixture was added to a mixture of aryl iodide **S47**[28] (435 mg, 870 µmol, 1 equiv), SPhos (71.3 mg, 174 µmol, 0.200 equiv) and SPhos-Pd-G2 (125 mg, 174 µmol, 0.20 equiv) in tetrahydrofuran (2.2 mL) and freshly distilled (over CaH_2) *N*,*N*-dimethylacetamide (2.2 mL) and the reaction mixture was directly placed in a preheated oil bath at 40 °C. After 15 min, the reaction mixture was allowed to cool to 23 °C and ethyl acetate (20 mL) and saturated aqueous ammonium chloride solution (20 mL) were added. The layers were separated, the aqueous layer was extracted with ethyl acetate (2 × 20 mL) and the combined organic extracts were washed with saturated aqueous sodium chloride solution (40 mL). The washed solution was dried over magnesium sulfate, the dried solution was filtered and the filtrate was concentrated. The residue was purified by flash-column chromatography on silica gel (1% ethyl acetate in hexanes) to yield methoxymethyl-ether **S48** (46.1 mg, 56%) as a colorless oil.

TLC (10% ethyl acetate in hexanes): R_f = 0.47 (UV, CAM).

^{1}H NMR (800 MHz, $CDCl_3$): δ = 7.34–7.29 (m, 4H), 7.26–7.23 (m, 1H), 7.20 (dd, *J* = 7.6, 1.7 Hz, 1H), 7.16–7.13 (m, 1H), 7.10 (dd, *J* = 8.2, 1.3 Hz, 1H), 6.92 (td, *J* = 7.6, 1.3 Hz, 1H), 5.42 (d, *J* = 5.7 Hz, 1H), 5.21 (d, *J* = 6.6 Hz, 1H), 5.15 (d, *J* = 6.6 Hz, 1H), 4.57 (d, *J* = 12.4 Hz, 1H), 4.33 (d, *J* = 12.4 Hz, 1H), 3.50 (s, 3H), 3.08 (s, 1H), 2.87 (d, *J* = 13.7 Hz, 1H), 2.61 (d, *J* = 13.7 Hz, 1H), 2.22–2.17 (m, 1H), 1.98–1.92 (m, 1H), 1.90–1.84 (m, 1H), 1.84–1.78 (m, 1H), 1.58–1.51 (m, 3H), 1.49–1.44 (m, 1H), 1.09 (s, 3H), 1.07 (d, *J* = 6.7 Hz, 3H), 0.88 (s, 3H), 0.83 (s, 3H).

^{13}C NMR (201 MHz, $CDCl_3$): δ = 156.7, 139.6, 132.1, 129.2, 128.2, 128.2, 127.8, 127.8, 127.3, 127.2, 121.3, 117.5, 114.3, 95.1, 83.0, 70.3, 70.3, 56.2, 56.2, 41.2, 39.9, 39.7, 37.2, 34.8, 32.0, 28.6, 25.8, 24.4, 22.7, 16.7.

IR (Diamond-ATR, neat): $\tilde{\nu}_{max}$ = 2952, 1493, 1453, 1232, 1154, 1077, 1051, 1004, 754, 697.

HRMS (EI) calc. for $C_{30}H_{40}O_3$ $[M]^+$: 448.2972; found: 448.2933.

$[\alpha]_D^{20}$ = +55.2° (*c* = 0.91, $CHCl_3$).

Alcohol 40

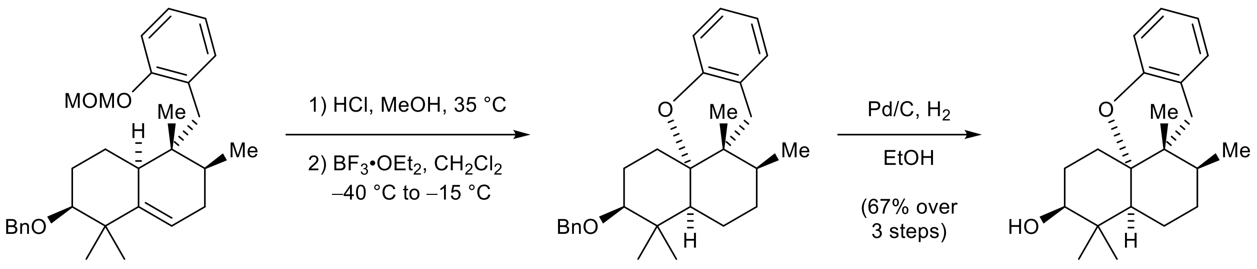

S48 **S49** **40**

A solution of hydrochloric acid (~1.25 M in methanol, 1.5 mL) was added to a solution of **S48** (36.0 mg, 80.2 µmol, 1 equiv) in dichloromethane (1 mL) and the resulting solution was heated to 35 °C. After 1 h, the reaction mixture was diluted with dichloromethane (8 mL) and saturated aqueous sodium bicarbonate solution (10 mL) was added. The layers were separated and the aqueous layer was extracted with dichloromethane (2 × 10 mL). The combined organic extracts were dried over magnesium sulfate. The dried solution was filtered and the filtrate was concentrated yielding a yellow foam that was directly used in the following reaction.

A solution of boron trifluoride diethyl etherate (48% in diethyl ether, 211 µL, 802 µmol, 10.0 equiv) was added dropwise to a solution of the crude phenol (32.2 mg, 80.2 µmol, 1 equiv) in dichloromethane (5 mL) at –40 °C and the reaction mixture was allowed to warm to –10 °C. After 0.5 h, saturated aqueous sodium bicarbonate solution (10 mL) was added, the layers were separated and the aqueous layer was extracted with dichloromethane (3 × 10 mL). The combined organic extracts were dried over magnesium sulfate, the dried solution was filtered and the filtrate was concentrated. The residue was directly used in the following step.

A solution of crude **S49** (32.2 mg, 80.2 µmol, 1 equiv) in ethanol (3 mL) was treated with oalladium on carbon (10 wt.%, 85.4 mg, 80.2 µmol, 1 equiv) at 23 °C. An atmosphere of hydrogen was maintained by sparging with a stream of pure hydrogen gas through a stainless steel needle for 2 min and vigorous stirring of the suspension was then continued under hydrogen atmosphere at 23 °C. After 14 h, the reaction mixture was filtered through a pad of Celite®. The filtercake was thoroughly rinsed with dichloromethane (50 mL). The filtrate was concentrated and the residue was purified by flash-column chromatography on silica gel (6% ethyl acetate in hexanes) to give **40** (17.0 mg, 67% over 3 steps) as a colorless oil.

TLC (10% ethyl acetate in hexanes): R_f = 0.28 (UV, CAM).

^{1}H NMR (400 MHz, $CDCl_3$): δ = 7.10–7.03 (m, 1H), 7.00 (d, *J* = 7.3 Hz, 1H), 6.81 (t, *J* = 7.3 Hz, 1H), 6.69 (d, *J* = 8.1 Hz, 1H), 3.60–3.53 (m, 1H), 3.44 (d, *J* = 17.0 Hz, 1H), 2.62–2.50 (m, 1H), 2.30–2.19 (m, 1H), 2.16–2.01 (m, 2H), 2.00–1.90 (m, 1H), 1.79–1.61 (m, 4H), 1.58–1.45 (m, 2H), 1.39–1.29 (m, 1H), 1.14 (d, *J* = 7.5 Hz, 3H), 1.05 (s, 3H), 0.99–0.93 (m, 6H).

^{13}C NMR (101 MHz, $CDCl_3$): δ = 151.6, 129.2, 126.8, 121.6, 119.5, 116.6, 82.9, 75.0, 44.7, 39.6, 38.3, 38.2, 37.1, 30.8, 28.3, 26.5, 26.0, 24.1, 23.9, 20.3, 17.3.

IR (Diamond-ATR, neat): $\tilde{\nu}_{max}$ = 3434, 2960, 2872, 1589, 1490, 1455, 1257, 974, 908, 732.

HRMS (EI) calc. for $C_{21}H_{30}O_2$ $[M]^+$: 314.2240; found: 314.2245.

$[\boldsymbol{a}]_D^{20}$ = +18.9° (c = 0.55, $CHCl_3$).

Benzylic alcohol S50

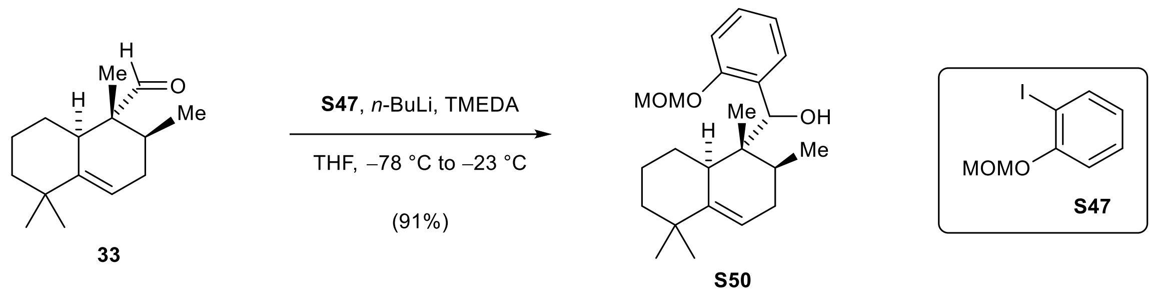

To a solution of iodide **S47** (168 mg, 635 µmol, 1.40 equiv) in tetrahydrofuran (1.5 mL) and freshly distilled *N*,*N*,*N′*,*N′*-tetramethylethane-1,2-diamine (over CaH_2, 192 µL, 1.27 mmol, 2.80 equiv) was added a solution of *n*-butyllithium (2.22 M in hexanes, 266 µL, 590 µmol, 1.30 equiv) at –78 °C and the reaction mixture was allowed to warm to –30 °C. After 45 min, the reaction mixture was cooled to –78 °C and a solution of aldehyde **33** (100 mg, 454 µmol, 1 equiv) in tetrahydrofuran (0.5 mL) was added. The reaction mixture was allowed to warm to 23 °C over a period of 30 min. Diethyl ether (10 mL) and saturated aqueous ammonium chloride solution (10 mL) was added. The layers were separated and the aqueous layer was extracted with diethyl ether (3 × 10 mL).The combined organic extracts were washed with saturated aqueous sodium chloride solution (30 mL). The washed solution was dried over sodium sulfate and the dried solution was filtered. The filtrate was concentrated and the crude product was purified by flash-column chromatography on silica gel (9% ethyl acetate in hexanes) to yield **S50** (148 mg, 91%) as a colorless oil. The inconsequential mixture of diastereoisomers was partially characterized by HRMS and IR spectroscopy.

TLC (10% ethyl acetate in hexanes): R_f = 0.44 (CAM).

IR (Diamond-ATR, neat): $\tilde{\nu}_{max}$ = 3480, 2954, 2924, 1488, 1452, 1229, 1153, 997, 923, 755.

HRMS (ESI) calcd for $C_{23}H_{35}O_3^+$ $[M+H]^+$: 359.2581; found: 359.2586.

Methoxymethyl-Ether S51

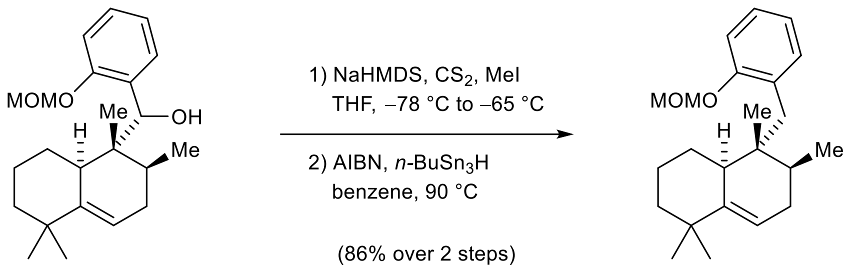

S50 **S51**

A solution of sodium bis(trimethylsilyl)amide (1 M in tetrahydrofuran, 2.02 mL, 2.02 mmol, 5.00 equiv) was added dropwise to a solution of **S50** (145 mg, 404 µmol, 1 equiv) in tetrahydrofuran (5 mL) at −78 °C. After 30 min, carbon disulfide (488 µL, 8.09 mmol, 20.0 equiv) was added dropwise to the orange solution and the resulting red solution was allowed to warm to −65 °C. After 1 h, methyl iodide (504 µL, 8.09 mmol, 20.0 equiv) was added to the reaction mixture. After 1 h, saturated aqueous ammonium chloride solution (20 mL) was added, the layers were separated and the aqueous layer was extracted with ethyl acetate (3 × 20 mL). The combined organic extracts were dried over sodium sulfate, the dried solution was filtered and the filtrate was concentrated. The residue was filtered through a plug of silica and the obtained crude product was used without further purification.

Note: benzene was degassed via freeze-pump-thaw (three cycles) prior to use.
A degassed solution of the xanthogenate (145 mg, 404 µmol, 1 equiv), 2,2'-azobis(2-methylpropionitrile) (AIBN) (33.2 mg, 202 µmol, 0.500 equiv) and tributyltin hydride (817 µL, 3.03 mmol, 7.50 equiv) in benzene (6.5 mL) was heated to 90 °C. After 1.5 h, the reaction mixture was cooled to 23 °C and directly purified by flash-column chromatography on silica gel (hexanes initially, grading to 2% ethyl acetate in hexanes) to yield **S51** as a colorless oil (119 mg, 86% over 2 steps).

TLC (5% ethyl acetate in hexanes): R_f = 0.37 (CAM).

^{1}H NMR (599 MHz, $CDCl_3$): δ = 7.19 (dd, *J* = 7.6, 1.7 Hz, 1H), 7.16–7.10 (m, 2H), 6.94–6.90 (m, 1H), 5.39–5.36 (m, 1H), 5.22 (d, *J* = 6.7 Hz, 1H), 5.16 (d, *J* = 6.7 Hz, 1H), 3.51 (s, 3H), 2.85 (d, *J* = 13.7 Hz, 1H), 2.60 (d, *J* = 13.7 Hz, 1H), 2.14 –2.10 (m, 1H), 2.01–1.95 (m, 1H), 1.85–1.77 (m, 2H), 1.56–1.52 (m, 1H), 1.52–1.48 (m, 1H), 1.46–1.41 (m, 1H), 1.39–1.35 (m, 1H), 1.17 (td, *J* = 13.2, 4.5 Hz, 1H), 1.05 (d, *J* = 6.7 Hz, 3H), 1.02 (s, 3H), 0.97–0.93 (m, 1H), 0.89 (s, 3H), 0.79 (s, 3H).

^{13}C NMR (151 MHz, $CDCl_3$): δ = 156.7, 146.8, 132.1, 129.1, 127.2, 121.2, 115.0, 114.2, 95.1, 56.2, 41.6, 40.3, 39.8, 37.3, 36.6, 34.8, 31.8, 30.0, 29.9, 28.3, 22.8, 16.8, 16.5.

IR (Diamond-ATR, neat): $\tilde{\nu}_{max}$ = 2956, 2928, 1493, 1453, 1232, 1155, 1078, 1008, 923, 754.

HRMS (EI) calcd for $C_{23}H_{34}O_2$ $[M]^+$: 342.2553; found: 342.2553.

$[\alpha]_D^{20}$ = +35.6° (c = 1.53, CH_2Cl_2).

Tetracycle 41

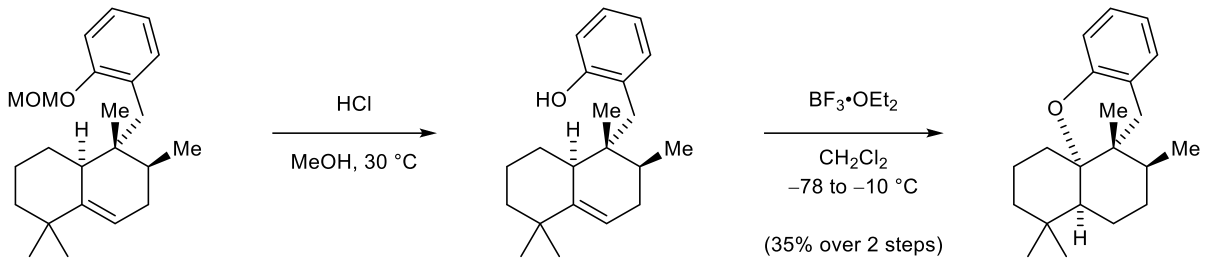

A solution of hydrochloric acid (~1.25 M in methanol, 1.5 mL) was added to a solution of **S51** (27.0 mg, 78.8 µmol, 1 equiv) in dichloromethane (0.5 mL) and the resulting solution was heated to 35 °C. After 1 h, the reaction mixture was diluted with dichloromethane (8 mL) and saturated aqueous sodium bicarbonate solution (10 mL) was added. The layers were separated and the aqueous layer was extracted with dichloromethane (2 × 10 mL). The combined organic extracts were dried over magnesium sulfate. The dried solution was filtered and the filtrate was concentrated to provide **S52** as a colorless foam that was used in the following reaction without further purification.

A solution of boron trifluoride diethyl etherate (48% in diethyl ether, 207 µL, 788 µmol, 10.0 equiv) was added dropwise to a solution of crude phenol **S52** (23.5 mg, 78.8 µmol, 1 equiv) in dichloromethane (2 mL) at –40 °C and the reaction mixture was allowed to warm to –10 °C over a period of 2.5 h. After 0.5 h, saturated aqueous sodium bicarbonate solution (10 mL) was added, the layers were separated and the aqueous layer was extracted with dichloromethane (3 × 10 mL). The combined organic extracts were dried over magnesium sulfate, the dried solution was filtered and the filtrate was concentrated. The residue was purified by flash-column chromatography on silica gel (hexanes initially, grading to 2% ethyl acetate in hexanes) to yield **41** (8.3 mg, 35% over two steps) as a colorless oil.

TLC (5% ethyl acetate in hexanes): $R_f = 0.68$ (CAM).

^{1}H NMR (400 MHz, $CDCl_3$): δ = 7.06 (t, *J* = 7.4 Hz, 1H), 6.99 (d, *J* = 7.4 Hz, 1H), 6.80 (t, *J* = 7.4 Hz, 1H), 6.73 (d, *J* = 8.1 Hz, 1H), 3.43 (d, *J* = 16.9 Hz, 1H), 2.19–1.99 (m, 3H), 1.88–1.76 (m, 2H), 1.75–1.65 (m, 2H), 1.64–1.55 (m, 1H), 1.54–1.42 (m, 3H), 1.40–1.33 (m, 1H), 1.25–1.17 (m, 1H), 1.12 (d, *J* = 7.5 Hz, 3H), 1.08 (s, 3H), 0.92 (s, 3H), 0.79 (s, 3H).

^{13}C NMR (101 MHz, $CDCl_3$): δ = 151.9, 129.2, 126.8, 121.5, 119.4, 116.7, 82.8, 44.5, 39.5, 38.2, 37.2, 34.1, 33.9, 32.1, 30.0, 29.4, 28.0, 22.4, 20.3, 18.5, 17.5.

IR (Diamond-ATR,neat): $\tilde{v}_{max}$ = 2932, 2872, 2362, 2335, 1590, 1492, 1456, 1260, 960, 750.

HRMS (EI) calcd for $C_{21}H_{30}O$ $[M]^+$: 298.2291; found: 298.2299.

$[\alpha]_D^{20}$ = +101.1° (c = 0.12, CH_2Cl_2)

Tetracycle 42

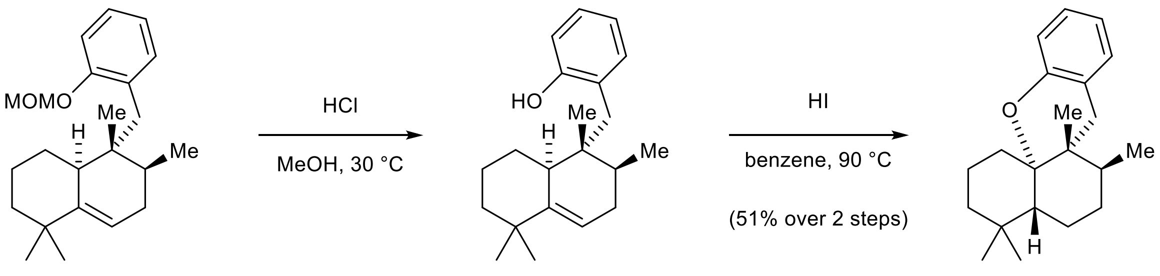

A solution of hydrochloric acid (~1.25 M in methanol, 1.5 mL mmol, equiv) was added to a solution of **S51** (26.5 mg, 77.4 µmol, 1 equiv) in dichloromethane (0.5 mL) and the resulting solution was heated to 35 °C. After 1 h, the reaction mixture was diluted with dichloromethane (8 mL) and saturated aqueous sodium bicarbonate solution (10 mL) was added. The layers were separated and the aqueous layer was extracted with dichloromethane (2 × 10 mL). The combined organic extracts were dried over magnesium sulfate, the dried solution was filtered and the filtrate was concentrated to yield **S52** as a colorless foam that was directly used in the following reaction without further purification.

A solution of hydroiodic acid (57 wt.% in water 102 µL, 774 µmol, 10.0 equiv) was added to a solution of the crude phenol **S52** (23.1 mg, 77.4 µmol, 1 equiv) in benzene (2 mL) in an Ace® pressure tube. The tube was sealed and the reaction mixture was heated to 90 °C. After 16 h, the reaction mixture was cooled to 23 °C and saturated aqueous sodium bicarbonate solution (10 mL) was added. The mixture was extracted with dichloromethane (3 × 10 mL), the combined organic extracts were dried over magnesium sulfate, the dried solution was filtered and the filtrate was concentrated. The residue was purified by flash-column chromatography on silica gel (*n*-heptane) to yield **42** (11.8 mg, 51% over two steps) as a colorless oil.

TLC (5% ethyl acetate in hexanes): R_f = 0.76 (CAM).

^{1}H NMR (400 MHz, $CDCl_3$): δ = 7.12–7.07 (m, 1H), 7.00–6.96 (m, 1H), 6.84–6.78 (m, 2H), 2.59 (d, *J* = 4.0 Hz, 2H), 1.79–1.63 (m, 4H), 1.61–1.54 (m, 1H), 1.52–1.26 (m, 6H), 1.25–1.16 (m, 1H), 1.14 (s, 3H), 0.93 (s, 3H), 0.93 (s, 3H), 0.76 (d, *J* = 6.8 Hz, 3H).

^{13}C NMR (101 MHz, $CDCl_3$): δ = 152.9, 129.5, 127.2, 121.3, 119.6, 117.0, 81.6, 45.8, 42.1, 37.4, 33.6, 33.6, 32.8, 31.9, 30.7, 29.0, 22.6, 22.0, 18.0, 17.1, 16.4.

IR (Diamond-ATR, neat): $\tilde{\nu}_{max}$ = 2949, 1586, 1488, 1456, 1387, 1254, 1171, 1036, 935, 750.

HRMS (EI) calcd for $C_{21}H_{30}O$ $[M]^+$: 298.2291; found: 298.2291.

$[\alpha]_D^{20}$ = +25.5° (c = 0.13, CH_2Cl_2).

Benzylic alcohol S54

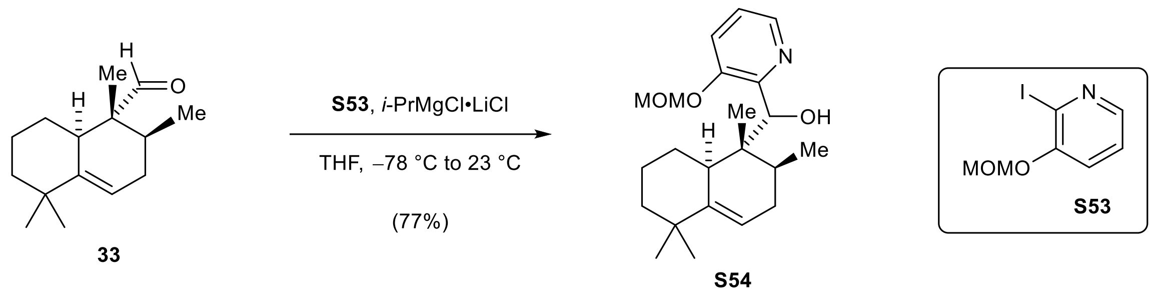

To a solution of **S53**[29] (178 mg, 672 µmol, 1.40 equiv) in tetrahydrofuran (1.5 mL) was added a solution of *iso*-propylmagnesium chloride lithium chloride complex (1.30 M in tetrahydrofuran, 517 µL, 672 µmol, 1.40 equiv) at –40 °C. After 15 min, a solution of aldehyde **33** (106 mg, 480 µmol, 1 equiv) in tetrahydrofuran (0.5 mL) was added and the reaction mixture was allowed to warm to 23 °C. After 24 h, diethyl ether (10 mL) and saturated aqueous ammonium chloride solution (10 mL) were added. The layers were separated and the aqueous layer was extracted with diethyl ether (3 × 10 mL).The combined organic extracts were washed with saturated aqueous sodium chloride solution (30 mL). The washed solution was dried over magnesium sulfate and the dried solution was filtered. The filtrate was concentrated and the residue was purified by flash-column chromatography on silica gel (10% ethyl acetate in hexanes) to yield **S54** (133 mg, 91%) as a colorless oil. The inconsequential mixture of diastereoisomers was partially characterized by HRMS and IR spectroscopy.

TLC (10% ethyl acetate in hexanes): $R_f = 0.11$ (CAM).

IR (Diamond-ATR, neat): $\tilde{v}_{max}$ = 3468, 2955, 2925, 1448, 1408, 1265, 1154, 1082, 1040, 986.

HRMS (ESI) calcd for $C_{22}H_{34}NO_3^+$ $[M+H]^+$: 360.2533; found: 360.2533.

Methoxymethyl-ether S55

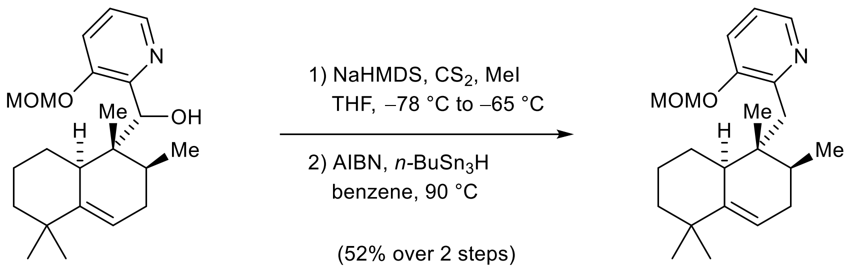

A solution of sodium bis(trimethylsilyl)amide (1 M in tetrahydrofuran, 1.78 mL, 1.78 mmol, 5.00 equiv) was added dropwise to a solution of **54** (128 mg, 356 µmol, 1 equiv) in tetrahydrofuran (5 mL) at −78 °C. After 35 min, carbon disulfide (430 µL, 7.12 mmol, 20.0 equiv) was added dropwise to the orange solution and the resulting red solution was allowed to warm to −65 °C. After 1 h, methyl iodide (443 µL, 7.12 mmol, 20.0 equiv) was added to the reaction mixture. After 1 h, saturated aqueous ammonium chloride solution (20 mL) was added, the layers were separated and the aqueous layer was extracted with diethyl ether (3 × 20 mL). The combined organic extracts were dried over magnesium sulfate, the dried solution was filtered and the filtrate was concentrated. The residue was filtered through a plug of silica and the obtained residue was used without further purification.

Note: benzene was degassed via freeze-pump-thaw (three cycles) prior to use.
A degassed solution of the xanthogenate (160 mg, 356 µmol, 1 equiv), 2,2'-azobis(2-methylpropionitrile) (AIBN) (29.2 mg, 178 µmol, 0.500 equiv) and tributyltin hydride (720 µL, 2.67 mmol, 7.50 equiv) in benzene (6.5 mL) was heated to 90 °C. After 2 h, the reaction mixture was cooled to 23 °C and directly purified by flash-column chromatography on silica gel (hexanes initially, grading to 5% ethyl acetate in hexanes) to yield **S55** as a colorless oil (64.0 mg, 52% over 2 steps).

TLC (10% ethyl acetate in hexanes): R_f = 0.38 (UV, $KMnO_4$).

^{1}H NMR (599 MHz, $CDCl_3$): δ = 8.19 (dd, *J* = 4.7, 1.5 Hz, 1H), 7.38 (dd, *J* = 8.2, 1.5 Hz, 1H), 7.04 (dd, *J* = 8.2, 4.7 Hz, 1H), 5.40 (dt, *J* = 5.7, 2.1 Hz, 1H), 5.21 (d, *J* = 6.9 Hz, 1H), 5.17 (d, *J* = 6.9 Hz, 1H), 3.50 (s, 3H), 3.00 (d, *J* = 13.4 Hz, 1H), 2.79 (d, *J* = 13.4 Hz, 1H), 2.38 (d, *J* = 12.6 Hz, 1H), 1.95–1.87 (m, 1H), 1.82–1.76 (m, 1H), 1.66 (d, *J* = 12.2 Hz, 1H), 1.62–1.56 (m, 1H), 1.51–1.46 (m, 1H), 1.46–1.40 (m, 1H), 1.38–1.34 (m, 1H), 1.20–1.14 (m, 1H), 1.06 (d, *J* = 6.7 Hz, 3H), 1.02 (s, 3H), 0.98–0.92 (m, 1H), 0.91 (s, 3H), 0.81 (s, 3H).

^{13}C NMR (151 MHz, $CDCl_3$): δ = 152.7, 151.4, 146.7, 141.8, 121.7, 120.7, 115.5, 95.0, 56.4, 41.5, 41.0, 40.6, 39.6, 36.4, 35.4, 31.9, 29.9, 29.6, 28.5, 22.8, 16.4, 16.0.

IR (Diamond-ATR, neat): $\tilde{\nu}_{max}$ = 2929, 1584, 1446, 1380, 1260, 1156, 1081, 1065, 995, 799.

HRMS (ESI) calcd for $C_{22}H_{34}NO_2^+$ $[M+H^+]^+$: 344.2584; found: 344.2584.

$[\alpha]_D^{22}$ = +45.4° (c = 0.55, CH_2Cl_2).

Pyrindine 43

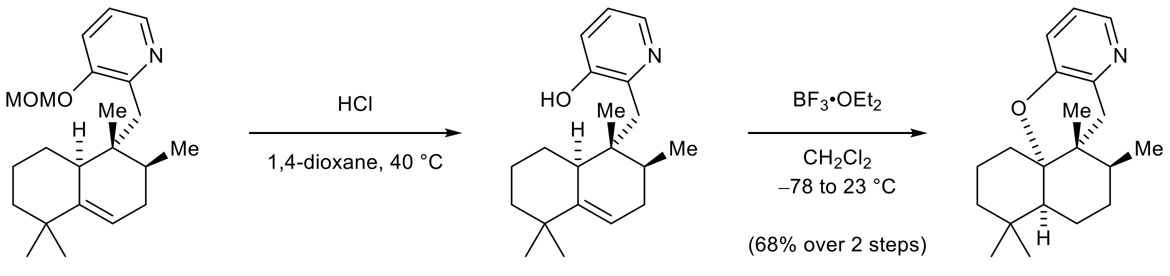

A solution of hydrochloric acid (4 M in 1,4-dioxane, 0.5 mL, 2.00 mmol, 43.0 equiv) was added to a solution of **S55** (16.0 mg, 46.6 µmol, 1 equiv) in dichloromethane (1 mL) and the resulting solution was heated to 40 °C. After 6 h, the reaction mixture was diluted with dichloromethane (8 mL) and saturated aqueous sodium bicarbonate solution (10 mL) was added. The layers were separated and the aqueous layer was extracted with dichloromethane (2 × 10 mL). The combined organic layers were dried over magnesium sulfate. The dried solution was filtered and the filtrate was concentrated to yield **S56** as a colorless foam that was directly used in the following reaction without further purification.

A solution of boron trifluoride diethyl etherate (48% in diethyl ether, 122 µL, 466 µmol, 10.0 equiv) was added dropwise to a solution of the crude phenol **S56** (14.0 mg, 46.6 µmol, 1 equiv) in dichloromethane (2.5 mL) at –40 °C and the reaction mixture was allowed to warm to 23 °C over a period of 2.5 h. After 7 h, saturated aqueous sodium bicarbonate solution (10 mL) was added, the layers were separated and the aqueous layer was extracted with dichloromethane (3 × 10 mL). The combined organic extracts were dried over magnesium sulfate, the dried solution was filtered and the filtrate was concentrated. The residue was purified by flash-column chromatography on silica gel (15% ethyl acetate in *n*-heptane) to yield **43** (9.5 mg, 68% over two steps) as an amorphous solid.

TLC (20% ethyl acetate in hexanes): R_f = 0.21 (UV, CAM).

^{1}H NMR (800 MHz, DMSO-d6): δ = 9.56 (s, 1H), 7.92 (dd, *J* = 4.5, 1.5 Hz, 1H), 7.06 (dd, *J* = 8.1, 1.6 Hz, 1H), 6.97 (dd, *J* = 8.1, 4.6 Hz, 1H), 2.96 (d, *J* = 13.3 Hz, 1H), 2.68 (d, *J* = 13.2 Hz, 1H), 2.27–2.20 (m, 1H), 1.98–1.90 (m, 1H), 1.92–1.82 (m, 3H), 1.82–1.76 (m, 1H), 1.56–1.49 (m, 2H), 1.43–1.37 (m, 1H), 1.37–1.32 (m, 1H), 1.29–1.25 (m, 1H), 0.93 (s, 3H), 0.92 (s, 3H), 0.84 (s, 3H), 0.76 (d, *J* = 6.8 Hz, 3H).

^{13}C NMR (201 MHz, DMSO-d6): δ = 151.9, 148.0, 138.9, 133.8, 132.4, 121.6, 120.9, 41.9, 39.2, 38.5, 33.9, 33.2, 28.4, 28.1, 26.1, 25.9, 21.8, 21.2, 19.6, 15.8.

IR (Diamond-ATR,neat): $\tilde{\nu}_{max}$ = 2926, 2612, 1576, 1456, 1378, 1359, 1287, 1173, 1115, 798.

HRMS (EI) calcd for $C_{20}H_{29}NO$ $[M]^+$: 299.2244; found: 299.2243.

$[\boldsymbol{\alpha}]_{\boldsymbol{D}}^{\mathbf{20}}$ =+51.6° (c = 0.17, CH_2Cl_2)

Pyridine 44

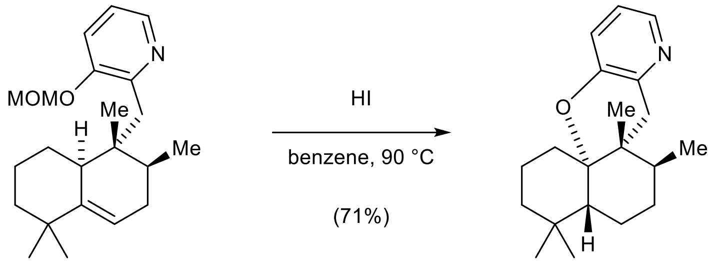

S55 **44**

A solution of hydroiodic acid (57 wt.% in water, 154 µL, 1.16 mmol, 40.0 equiv) was added to a solution of **S55** (10.0 mg, 29.1 µmol, 1 equiv) in benzene (1.5 mL) in an Ace® pressure tube. The tube was sealed and heated to 90 °C. After 17 h, the reaction mixture was cooled to 23 °C and saturated aqueous sodium bicarbonate solution (8 mL) and saturated aqueous sodium thiosulfate (2 mL) were added. The aqueous layer was extracted with dichloromethane (3 × 10 mL). The combined organic extracts were dried over magnesium sulfate, the dried solution was filtered and the filtrate was concentrated. The residue was purified by flash-column chromatography on silica gel (20% ethyl acetate in *n*-heptane) to yield **44** (6.20 mg, 71%) as colorless amorphous solid. Recrystallization from ethyl acetate gave crystals suitable for single-crystal X-ray diffraction.

TLC (20% ethyl acetate in hexanes): $R_f = 0.27$ (UV, CAM).

^{1}H NMR (599 MHz, $CDCl_3$)): δ = 8.09 (dd, *J* = 4.6, 1.5 Hz, 1H), 7.09 (dd, *J* = 8.2, 1.5 Hz, 1H), 7.04 (dd, *J* = 8.2, 4.6 Hz, 1H), 2.86 (d, *J* = 18.0 Hz, 1H), 2.70 (d, *J* = 18.0 Hz, 1H), 1.74–1.57 (m, 5H), 1.53–1.48 (m, 1H), 1.47–1.38 (m, 3H), 1.34–1.28 (m, 2H), 1.21 (td, *J* = 13.5, 3.5 Hz, 1H), 1.10 (s, 3H), 0.97 (s, 3H), 0.93 (s, 3H), 0.80 (d, *J* = 6.8 Hz, 3H).

^{13}C NMR (151 MHz, $CDCl_3$): δ = 149.2, 143.5, 141.2, 124.0, 122.5, 82.6, 45.7, 41.9, 38.3, 36.7, 33.6, 32.7, 32.4, 30.6, 29.0, 22.5, 21.9, 17.9, 17.1, 16.4.

IR (Diamond-ATR, neat): $\tilde{v}_{max}$ = 2948, 1438, 1258, 1170, 1107, 1016, 931, 908, 805, 720.

HRMS (ESI) calcd for $C_{20}H_{30}NO^+$ $[M+H]^+$: 300.2322; found: 300.2321.

$[\alpha]_D^{20}$ = +54.9° (c = 0.12, CH_2Cl_2).

Synthesis of (–)-Mamanuthaquinone (6)

Arene 36

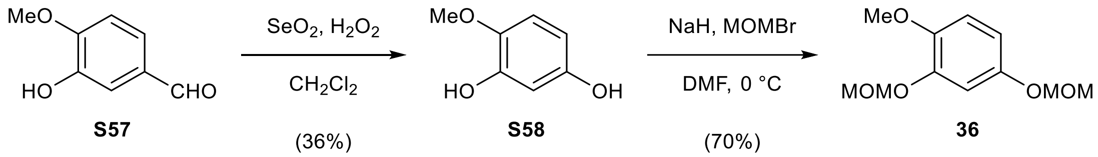

S57 **S58** **36**

Selenium dioxide (233 mg, 2.10 mmol, 0.0800 equiv) was added to a solution of *iso*-vanillin (**S57**) (4.00 g, 26.3 mmol, 1 equiv) and hydrogenperoxide (30% in water, 5.91 mL, 57.8 mmol, 2.20 equiv) in dichloromethane (70 mL). After 16 h, water (30 mL) was added. The layers were separated and the organic layer was washed with saturated aqueous sodium bicarbonate solution (30 mL). The washed solution was dried over sodium sulfate, the dried solution was filtered and the filtrate was concentrated to yield **S58** (1.32 g, 36%) as a clear, colorless solid. The obtained analytical data were in full agreement with those values reported in the literature.[30]

Sodium hydride (60% mineral oil dispersion, 771 mg, 19.3 mmol, 2.50 equiv) was slowly added to a solution of bisphenol **S58** (1.08 g, 7.71 mmol, 1 equiv) in *N*,*N*-dimethylformamide (30 mL) at 0 °C. After 1 h, bromomethyl methyl ether (1.29 mL, 15.8 mmol, 2.05 equiv) was added to the dark brown suspension and the reaction mixture was allowed to warm to 23 °C. After 5 h, water (40 mL) was carefully added and the aqueous layer was extracted with diethyl ether (3 × 30 mL). The combined organic extracts were washed with saturated aqueous sodium chloride solution (100 mL), the washed solution was dried over sodium sulfate, the dried solution was filtered and the filtrate was concentrated. The residue was purified by flash-column chromatography on silica gel (30% ethyl acetate in hexanes) to yield **36** (1.23 g, 70%) as a colorless oil.

TLC (20% ethyl acetate in hexanes): $R_f = 0.20$ (CAM).

^{1}H NMR (599 MHz, $CDCl_3$): δ = 6.90 (d, *J* = 2.8 Hz, 1H), 6.80 (d, *J* = 8.9 Hz, 1H), 6.67 (dd, 8.9, 2.8 Hz, 1H), 5.21 (s, 2H), 5.10 (s, 2H), 3.83 (s, 3H), 3.51 (s, 3H), 3.48 (s, 3H).

^{13}C NMR (151 MHz, $CDCl_3$): δ = 151.5, 147.1, 145.1, 112.4, 109.0, 106.8, 95.5, 95.2, 56.4, 56.2, 55.9.

IR (Diamond-ATR, neat): $\tilde{\nu}_{max}$ = 2902, 2363, 1596, 1505, 1226, 1150, 1130, 1074, 998, 922.

HRMS (EI) calcd for $C_{11}H_{16}O_5$ $[M]^+$: 228.0992; found: 228.0984.

Benzylic alcohol S59

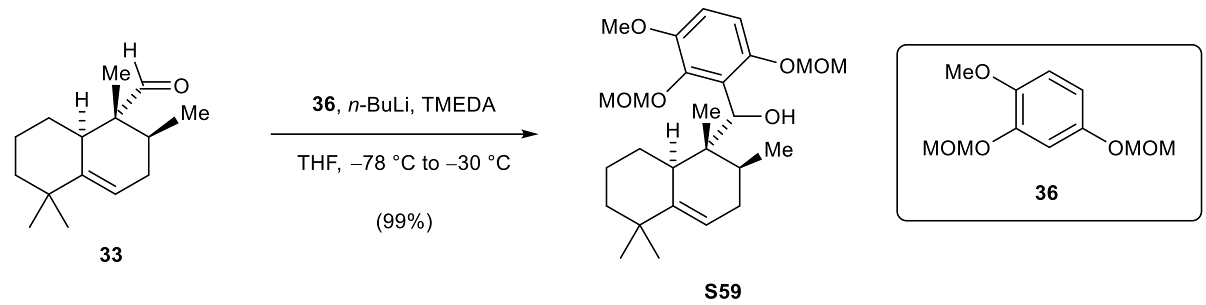

To a solution of **36** (224 mg, 981 µmol, 1.40 equiv) in tetrahydrofuran (4 mL) and freshly distilled (over CaH_2) *N*,*N*,*N′*,*N′*-tetramethylethane-1,2-diamine (317 µL, 2.10 mmol, 3.00 equiv) was added a solution of *n*-butyllithium (2.44 M in hexanes 362 µL, 911 µmol, 1.30 equiv) at –78 °C. The reaction mixture was allowed to warm to –30 °C. After 1.5 h, the reaction mixture was cooled to –78 °C and aldehyde **33** (154 mg, 700 µmol, 1 equiv) in tetrahydrofuran (2 mL) was added. The reaction mixture was warmed to –30 °C over a period of 2 h, then diethyl ether (30 mL) and saturated aqueous ammonium chloride solution (30 mL) were added. The layers were separated, the aqueous layer was extracted with diethyl ether (3 × 20 mL) and the combined organic extracts were washed with saturated aqueous sodium chloride solution (40 mL). The washed solution was dried over sodium sulfate and the dried solution was filtered. The filtrate was concentrated and the residue was purified by flash-column chromatography on silica gel (10% ethyl acetate in hexanes) to yield **S59** (313 mg, 99%) as a colorless oil. The mixture of inseparable diastereoisomers was characterized by HRMS and IR spectroscopy.

TLC (20% ethyl acetate in hexanes): $R_f = 0.18$ (CAM).

IR (Diamond-ATR, neat): $\tilde{v}_{max}$ = 3565, 2953, 1508, 1484, 1256, 1229, 1154, 1077, 1027, 924.

HRMS (EI) calcd for $C_{26}H_{40}O_6$ $[M]^+$: 448.2819; found: 448.2841.

Olefin 37

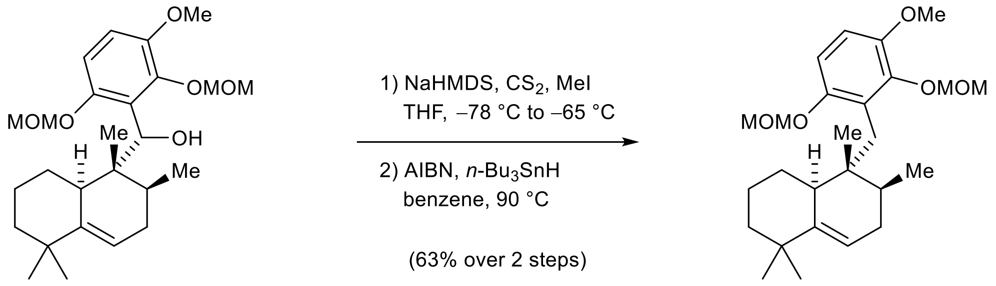

A solution of sodium bis(trimethylsilyl)amide (1 M in tetrahydrofuran, 3.49 mL, 3.49 mmol, 5.00 equiv) was added dropwise to a solution of **S59** (313 mg, 698 µmol, 1 equiv) in tetrahydrofuran (7 mL) at −78 °C. After 30 min, carbon disulfide (842 µL, 14.0 mmol, 20.0 equiv) was slowly added and the reaction mixture was allowed to warm to −65 °C. After 1 h, methyl iodide (869 µL, 14.0 mmol, 20.0 equiv) was slowly added to the reaction mixture. After 1 h, saturated aqueous ammonium chloride solution (25 mL) and ethyl acetate (30 mL) were added, the layers were separated and the aqueous layer was extracted with ethyl acetate (3 × 30 mL). The combined organic extracts were dried over sodium sulfate, the dried solution was filtered and the filtrate was concentrated. The residue was filtered through a short plug of silica and the obtained residue was used without further purification.

Note: benzene was degassed via freeze-pump-thaw (three cycles) prior to use.
A degassed solution of the xanthogenate (376 mg, 698 µmol, 1 equiv), 2,2'-azobis(2-methylpropionitrile) (AIBN) (56.6 mg, 344 µmol, 0.500 equiv) and tributyltin hydride (1.50 g, 5.17 mmol, 7.50 equiv) in benzene (25 mL) was heated to 90 °C. After 6 h, the reaction mixture was directly purified by flash-column chromatography on silica gel (3% ethyl acetate in hexanes) to yield **37** as a colorless oil (190 mg, 63% over 2 steps).

TLC (20% ethyl acetate in hexanes): R_f = 0.34 (CAM).

^{1}H NMR (800 MHz, , C_6D_6): δ = 6.94 (d, *J* = 8.9 Hz, 1H), 6.48 (d, *J* = 8.9 Hz, 1H), 5.62–5.56 (m, 1H), 5.13 (q, *J* = 5.8 Hz, 2H), 4.98–4.88 (m, 2H), 3.42 (s, 3H), 3.30 (s, 3H), 3.26 (d, *J* = 12.9 Hz, 1H), 3.23 (s, 3H), 3.00 (d, *J* = 12.9 Hz, 1H), 2.96–2.91 (m, 1H), 2.04–1.99 (m, 2H), 1.84–1.77 (m, 1H), 1.67–1.58 (m, 1H), 1.53–1.48 (m, 1H), 1.46–1.40 (m, 2H), 1.28–1.23 (m, 1H), 1.22 (s, 3H), 1.18 (d, *J* = 6.8 Hz, 3H), 1.16 (s, 3H), 1.09 (s, 3H), 0.97–0.89 (m, 1H).

^{13}C NMR (201 MHz, C_6D_6): δ = 151.9, 147.8, 147.7, 147.5, 126.0, 115.5, 111.3, 109.7, 99.5, 95.8, 57.4, 56.0, 55.8, 42.6, 41.9, 41.2, 39.6, 36.9, 36.3, 32.3, 31.5, 30.3, 28.4, 23.4, 16.8, 15.0.

IR (Diamond-ATR, neat): $\tilde{\nu}_{max}$ = 2930, 1484, 1251, 1152, 1075, 1038, 982, 925, 801, 720.

HRMS (EI) calcd for $C_{26}H_{40}O_5$ $[M]^+$: 432.2870; found: 432.2868.

$[\alpha]_D^{20}$ = +1.61° (c = 0.66, CH_2Cl_2)

(–)-Mamanuthaquinone (6)

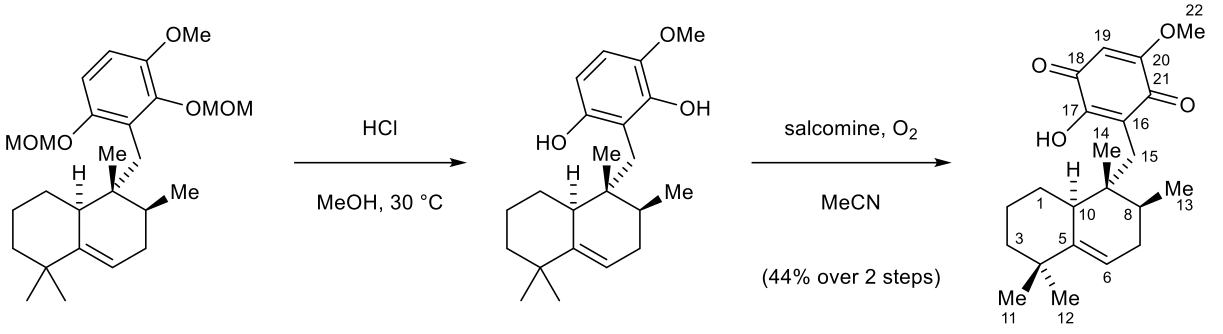

A solution of hydrochloric acid (~1.25 M in methanol, 3 mL) was added to a solution of **37** (33.0 mg, 76.3 µmol, 1 equiv) in dichloromethane (1 mL) and the resulting solution was heated to 30 °C. After 5 h, the reaction mixture was diluted with dichloromethane (5 mL) and saturated aqueous sodium bicarbonate solution (5 mL) was added. The layers were separated and the aqueous layer was extracted with dichloromethane (3 × 5 mL). The combined organic extracts were dried over sodium sulfate. The dried solution was filtered and the filtrate was concentrated to yield **S60** as a brown foam that was directly used in the following reaction.

N,*N'*-Bis(salicylidene)ethylenediaminocobalt(II) (19.8 mg, 61.0 mmol, 0.800 equiv) was added to a solution of crude phenol **S60** (26.3 mg, 76.3 µmol, 1 equiv) in *N*,*N*-dimethylformamide (6 mL) at 23 °C and oxygen was passed through the reaction mixture for 20 min. After 60 min, water (20 mL) was added and the mixture was extracted with diethyl ether (4 × 20 mL). The combined organic extracts were dried over sodium sulfate, the dried solution was filtered and the filtrate was concentrated. The residue was purified by flash-column chromatography on silica gel (40% ethyl acetate in hexanes with 0.5% acetic acid) to give (–)-mamanuthaquinone (**6**) (12.0 mg, 44% over 2 steps) as a bright yellow oil.

TLC (40% ethyl acetate in hexanes with 0.5% acetic acid), R_f = 0.21 (UV, CAM).

^{1}H NMR (599 MHz, $CDCl_3$): δ = 7.43 (s, 1H, O-H), 5.87 (s, 1H, H-19), 5.44–5.34 (m, 1H, H-6), 3.87 (s, 3H, H-22), 2.61 (d, $^2J_{15A/15B}$ = 13.2 Hz, 1H, H-15$_A$), 2.48 (d, $^2J_{15B/15A}$ = 13.2 Hz, 1H, H-15$_B$), 2.11 (br d, $^3J_{10/1A}$ = 12.4 Hz, 1H, H-10), 2.02–1.96 (m, 1H, H-7$_A$), 1.83 (d, $^3J_{1A/10}$ = 12.4 Hz, 1H, H-1$_A$), 1.80–1.74 (m, 1H, H-7$_B$), 1.51–1.48 (m, 1H, H-2$_A$), 1.46–1.35 (m, 3H, H-2$_B$, H-3$_A$, H-8), 1.17–1.13 (m, 1H, H-3$_B$), 1.03 (s, 3H, H-11), 1.00 (d, $^3J_{13/8}$ = 7.0 Hz, 3H, H-13), 0.97–0.94 (m, 4H, H-1$_B$, H-12), 0.75 (s, 3H, H-14).

^{13}C NMR (201 MHz, $CDCl_3$): δ = 182.6 (C-18), 182.4 (C-21), 161.8 (C-20), 153.0 (C-17), 146.5 (C-5), 118.5 (C-16), 115.1 (C-6), 102.2 (C-19), 57.0 (C-22), 41.9 (C-10), 41.5 (C-3), 41.1 (C-9), 36.7 (C-8), 36.6 (C-4), 32.9 (C-15), 31.7 (C-7), 30.8 (C-1), 29.9 (C-11), 28.2 (C-12), 22.9 (C-2), 16.8 (C-13), 16.2 (C-14).

IR (Diamond-ATR, neat): $\tilde{v}_{max}$ = 3344, 2924, 2891, 2363, 1645, 1608, 1446, 1350, 1234, 1035.

HRMS (EI) calcd for $C_{22}H_{30}O_4$ $[M]^+$: 358.2139; found: 358.2145.

$[\alpha]_D^{20}$ = –258.4° (*c* = 0.16, CH_2Cl_2); lit. $[\alpha]_{546}^{n.a.}$ = –31° (c = 0.058, $CHCl_3$). n.a. not available[31]*

* This lamp (λ = 546 nm) was not accessible in our laboratory.

Supplementary Table 10 Comparison of ^{1}H NMR data for natural and synthetic (–)-mamanuthaquinone (**6**).

**Proton**	**Synthetic (599 MHz, $CDCl_3$)**	**Natural (500 MHz, $CDCl_3$)**[31]	**Δ: δ (ppm)**
1A	1.83 (d, *J* = 12.4 Hz, 1H)	1.79 (br d, 1H)	+0.04
1B	0.97–0.94 (m, 4H)	0.90 (s, 1H)	
2A	1.51–1.48 (m, 1H)		
2B	1.46–1.35 (m, 3H)		
3A	1.46–1.35 (m, 3H)		
3B	1.17–1.13 (m, 1H)	1.12 (ddd, *J* = 13.5, 13.5, 4.3 Hz, 1H)	+ 0.03
6	5.44–5.34 (m, 1H)	5.35 (br s, 1H)	+ 0.04
7A	1.99 (d, *J* = 17.8 Hz, 1H)	1.95 (ddd, *J* = 18, 17.5, 4.5 Hz, 1H)	+0.04
7B	1.80–1.74 (m, 1H)	1.73 (m, 1H)	+ 0.04
8	1.46–1.35 (m, 3H)	1.33–1.45 (m, 4H)	
10	2.11 (d, *J* = 12.4 Hz, 1H)	2.08 (br d, *J* = 13 Hz, 1H)	+0.03
11	1.03 (s, 3H),	0.99 (s, 3H)	+0.04
12	0.97–0.94 (m, 4H)	0.92 (s, 3H)	
13	1.00 (d, *J* = 7.0 Hz, 3H)	0.96 (d, *J* = 7 Hz, 3H)	+0.04
14	0.75 (s, 3H).	0.73 (s, 3H)	+0.02
15A	2.61 (d, *J* = 13.2 Hz, 1H)	2.58 (d = 13.0 Hz, 1H)	+0.03
15B	2.48 (d, *J* = 13.1 Hz, 1H)	2.45 (d, *J* = 13.0 Hz, 1H)	+0.03
19	5.87 (s, 1H)	5.84 (s, 1H)	+0.03
22	3.87 (s, 3H)	3.84 (s, 3H)	+0.03
OH	7.43 (s, 1H)	7.45 (s, 1H)	–0.02

Supplementary Table 11 Comparison of ^{13}C NMR data for natural and synthetic (–)-mamanuthaquinone (**6**). n.a. not available

Carbon	Synthetic (201 MHz, $CDCl_3$)	Natural (n.a., $CDCl_3$)[31]	Δ: δ (ppm)
1	30.8	30.6	+ 0.2
2	22.9	22.7	+ 0.2
3	41.5	41.2	+ 0.3
4	36.6	36.3	+ 0.3
5	146.5	146.3	+ 0.2
6	115.1	114.8	+ 0.3
7	31.7	31.5	+ 0.2
8	36.7	36.4	+ 0.3
9	41.1	40.9	+ 0.2
10	41.9	41.7	+ 0.2
11	29.9	29.7	+ 0.2
12[#]	28.2	27.9	+ 0.3
13[#]	16.8	16.5	+ 0.3
14	16.2	16.0	+ 0.2
15	32.9	32.7	+ 0.2
16	118.5	118.3	+ 0.2
17	153.0	152.8	+ 0.2
18	182.6	182.4	+ 0.2
19	102.2	102.0	+ 0.2
20	161.8	161.5	+ 0.3
21	182.4	182.0	+ 0.4
22	57.0	56.8	+ 0.2

[#] Carbon was reassigned by us on the basis of 2D-NMR studies

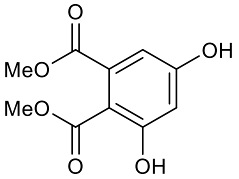

11

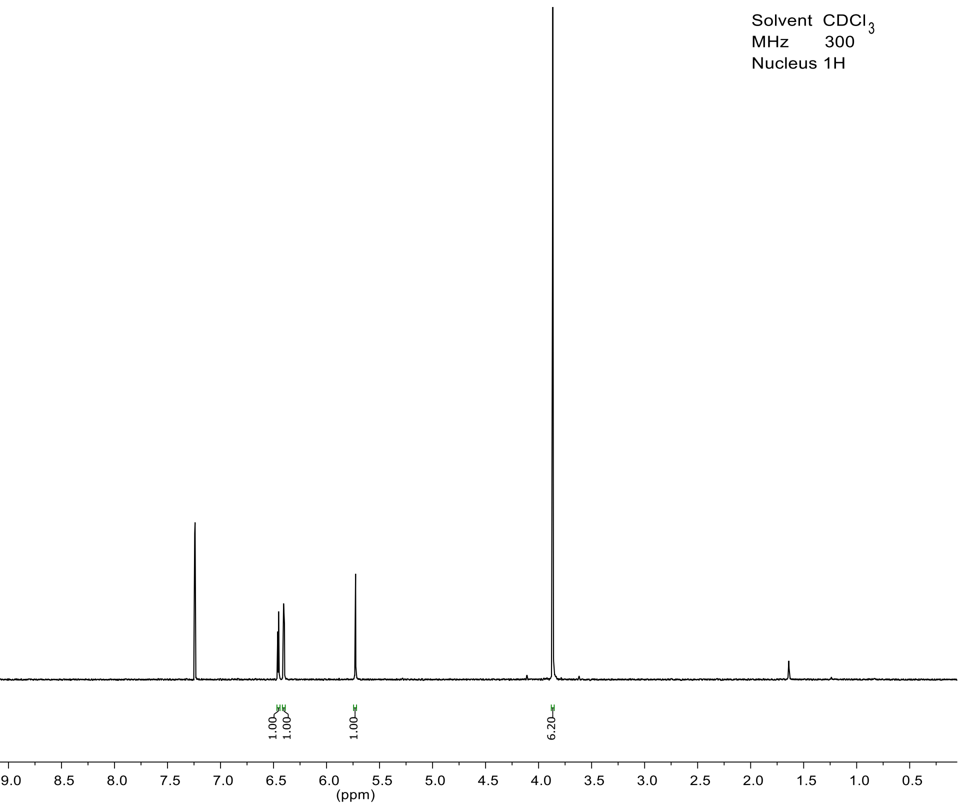

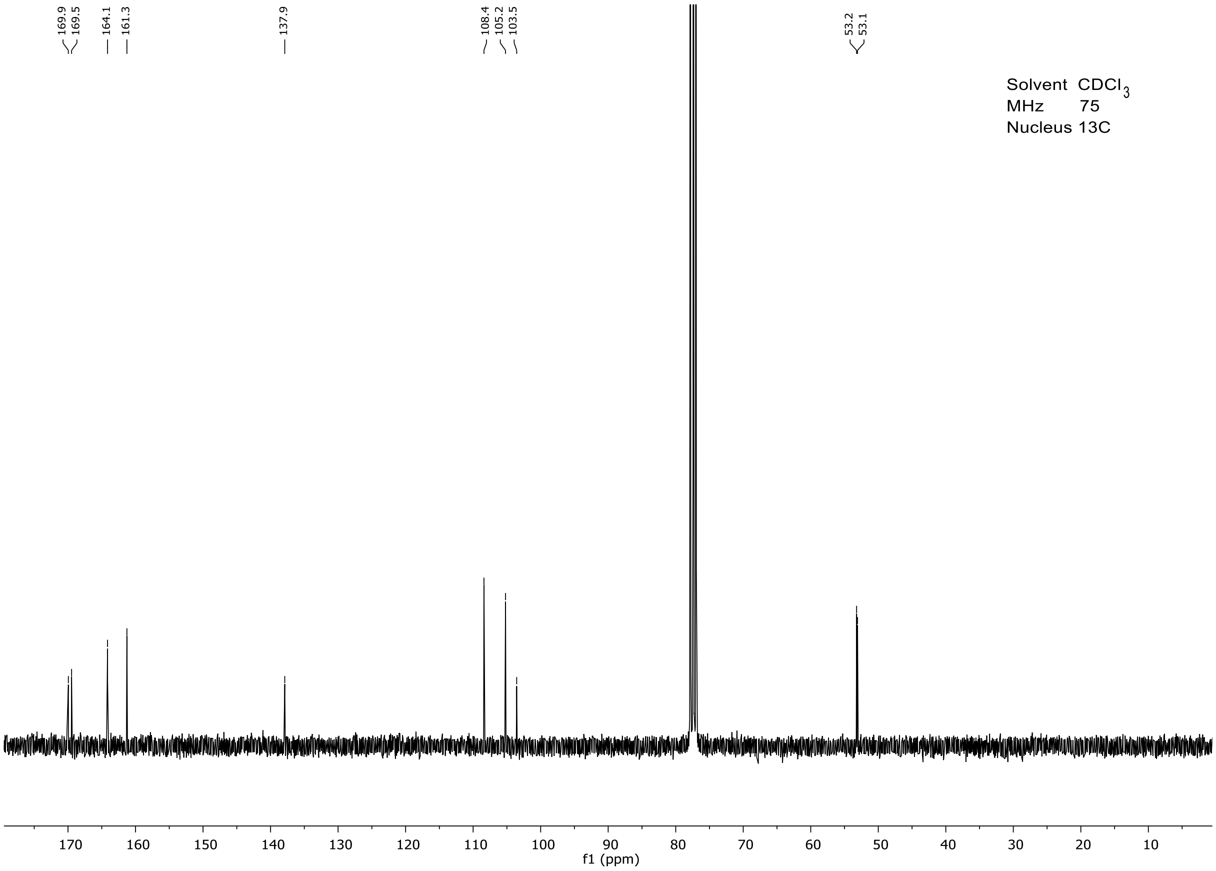

Supplementary Figure 2 ^{1}H and ^{13}C NMR Spectra for **11** in $CDCl_3$.

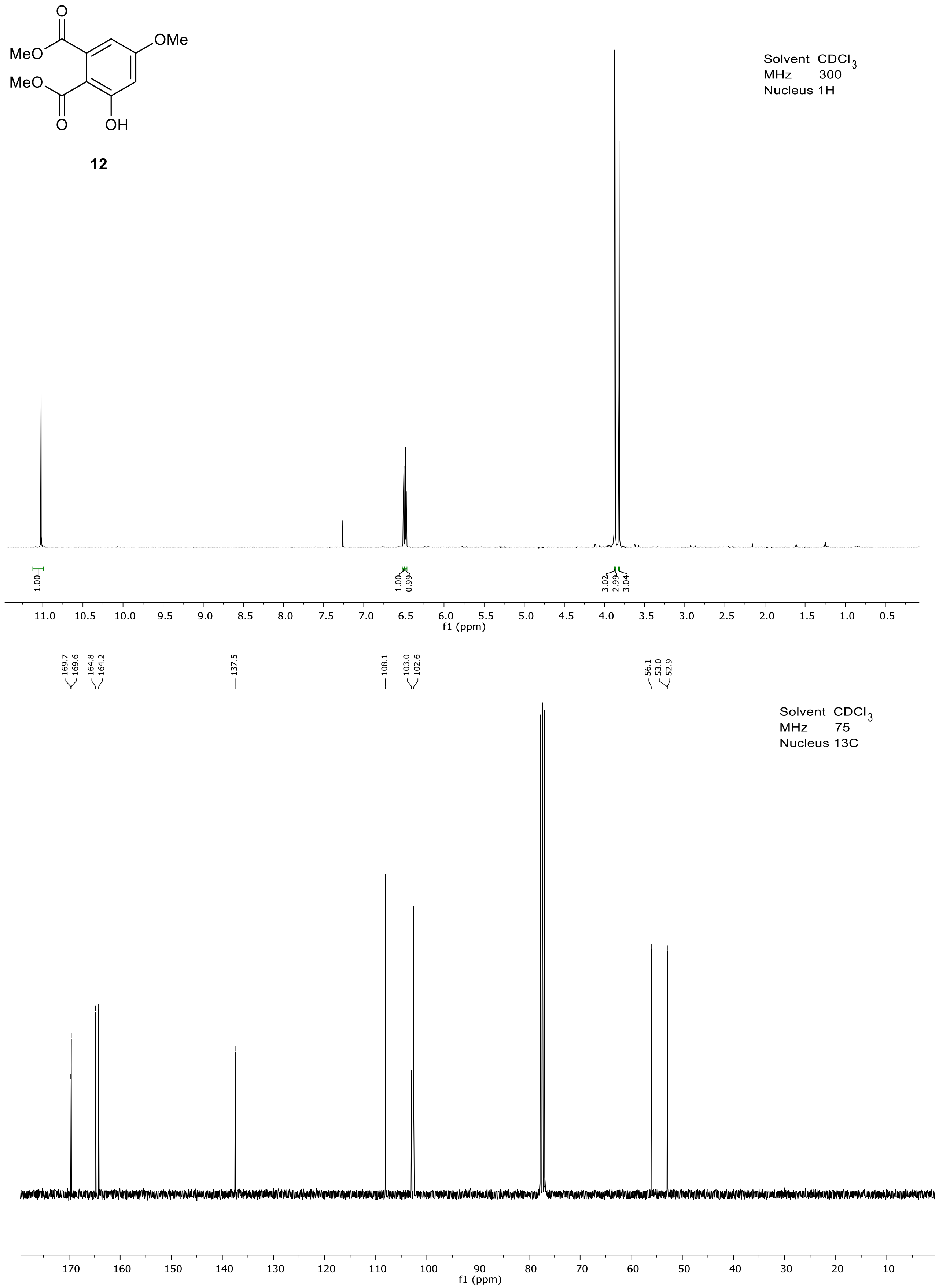

Supplementary Figure 3 ^{1}H and ^{13}C NMR Spectra for **12** in $CDCl_3$.

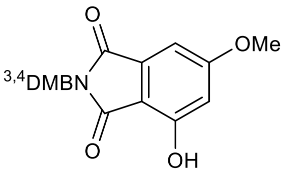

13

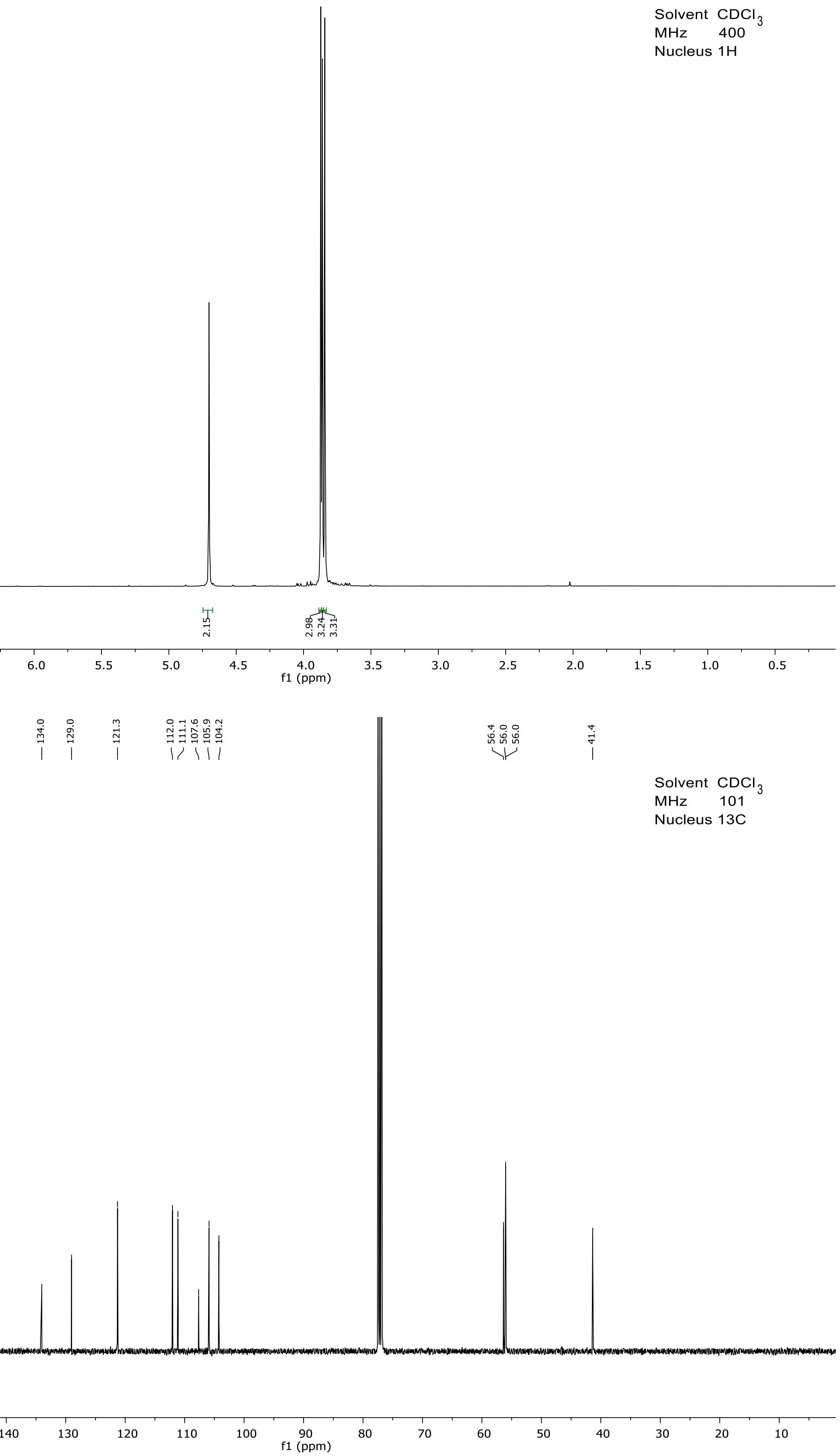

Supplementary Figure 4 ^{1}H and ^{13}C NMR Spectra for **13** in $CDCl_3$.

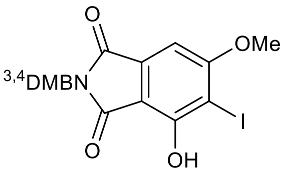

14

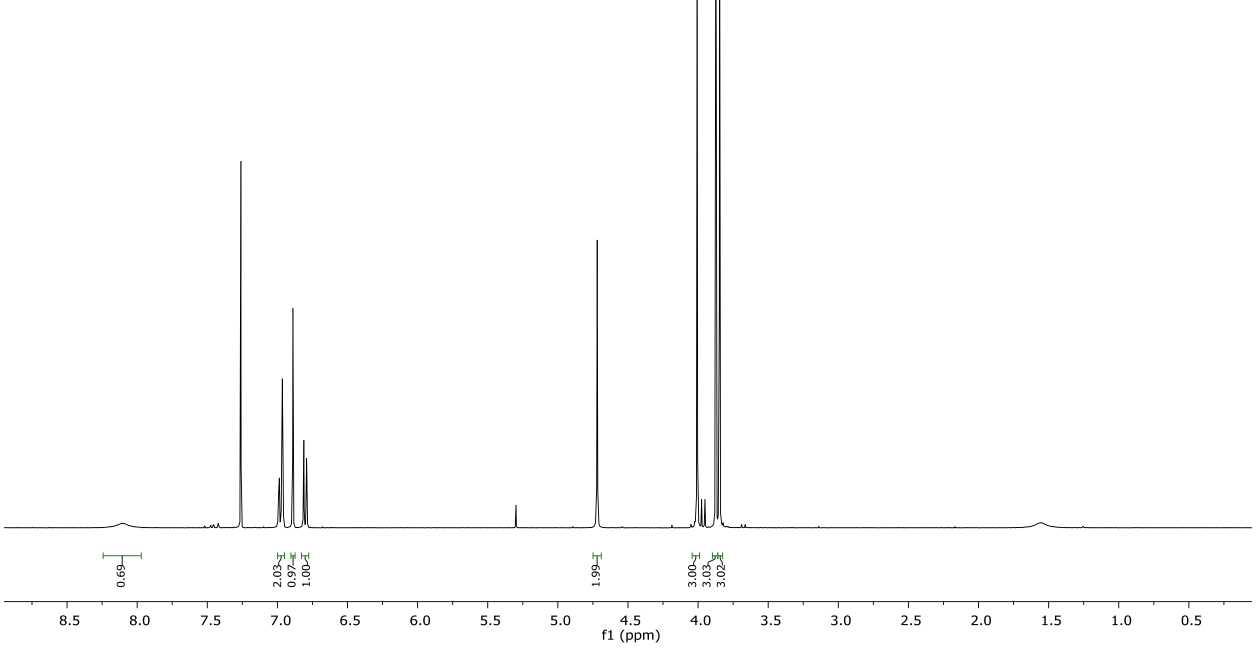

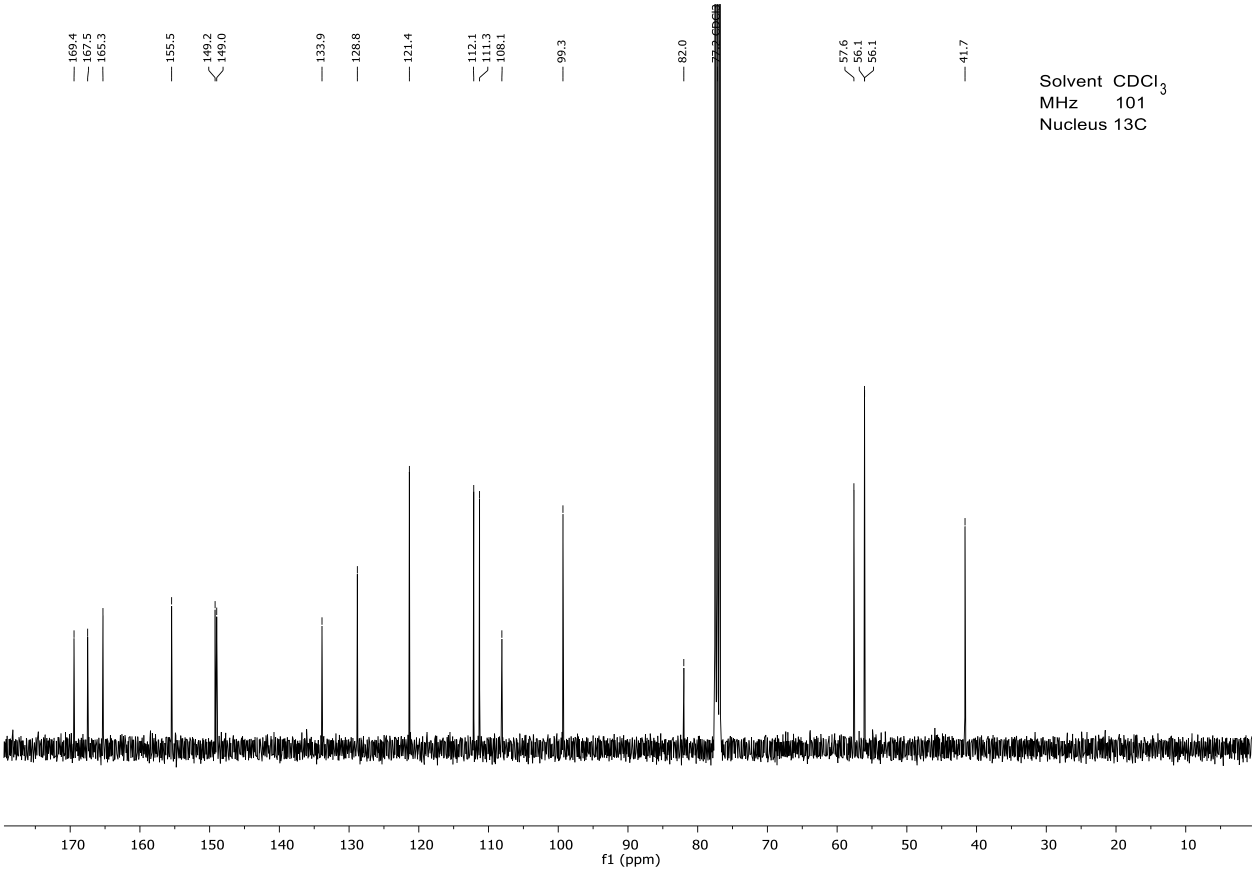

Supplementary Figure 5 ^{1}H and ^{13}C NMR Spectra for **14** in $CDCl_3$.

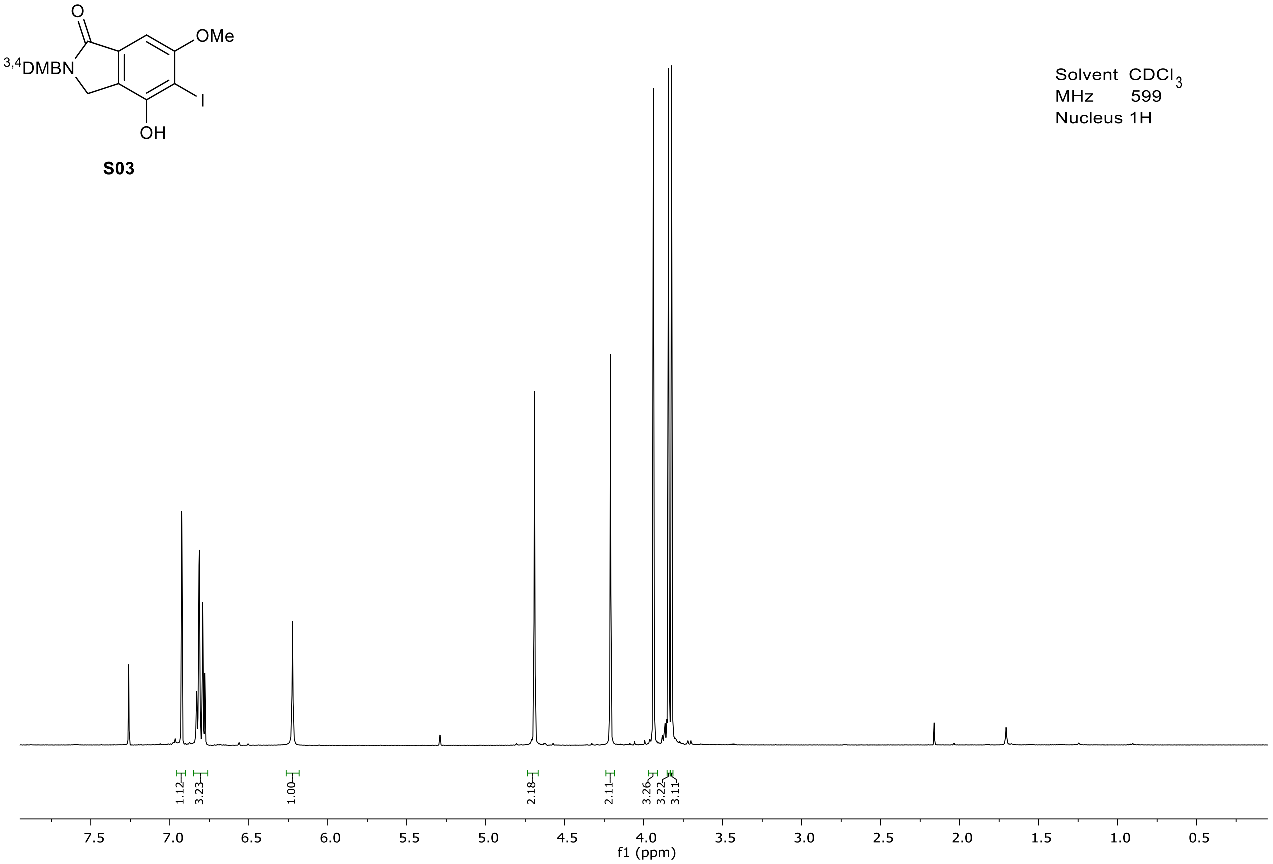

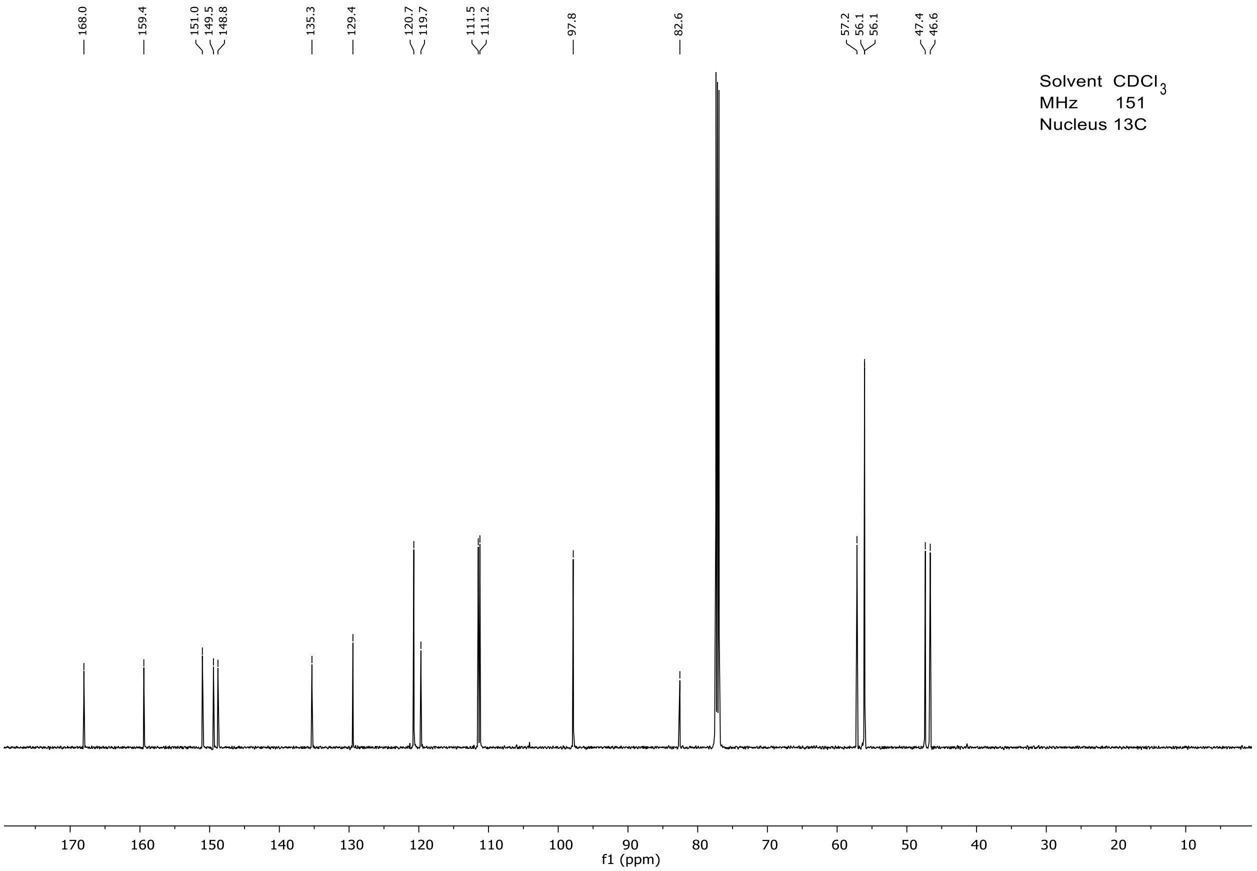

Supplementary Figure 6 ^{1}H and ^{13}C NMR Spectra for **S03** in $CDCl_3$.

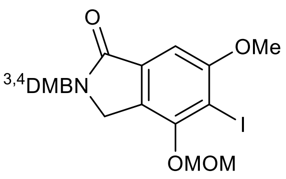

15

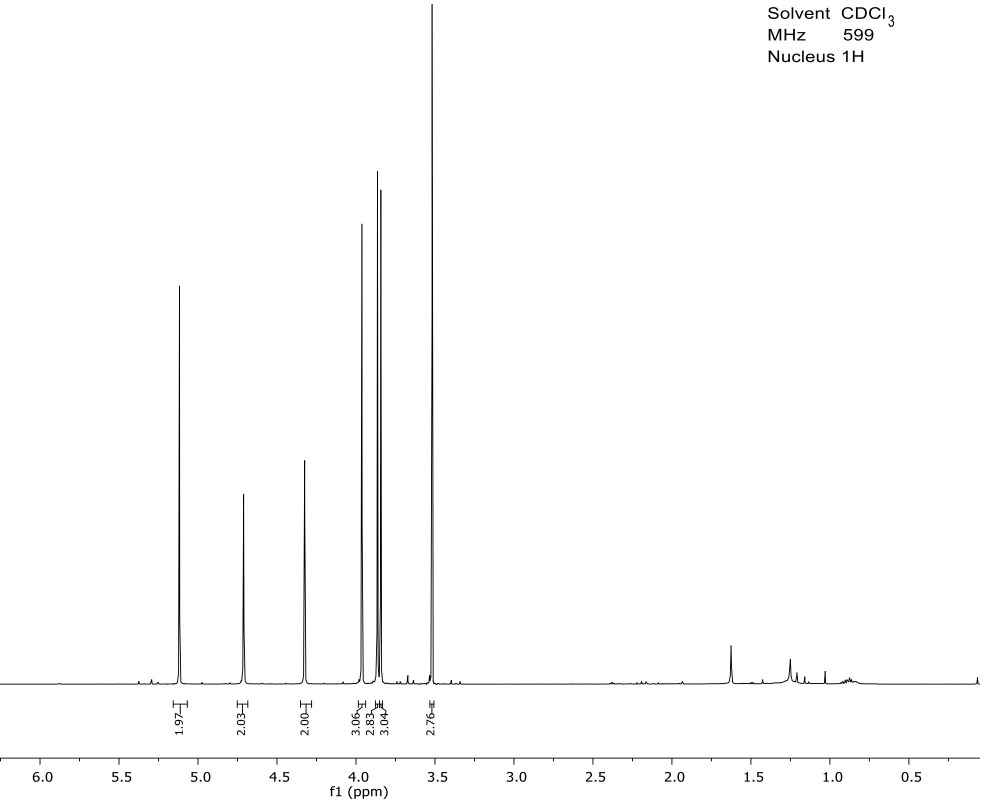

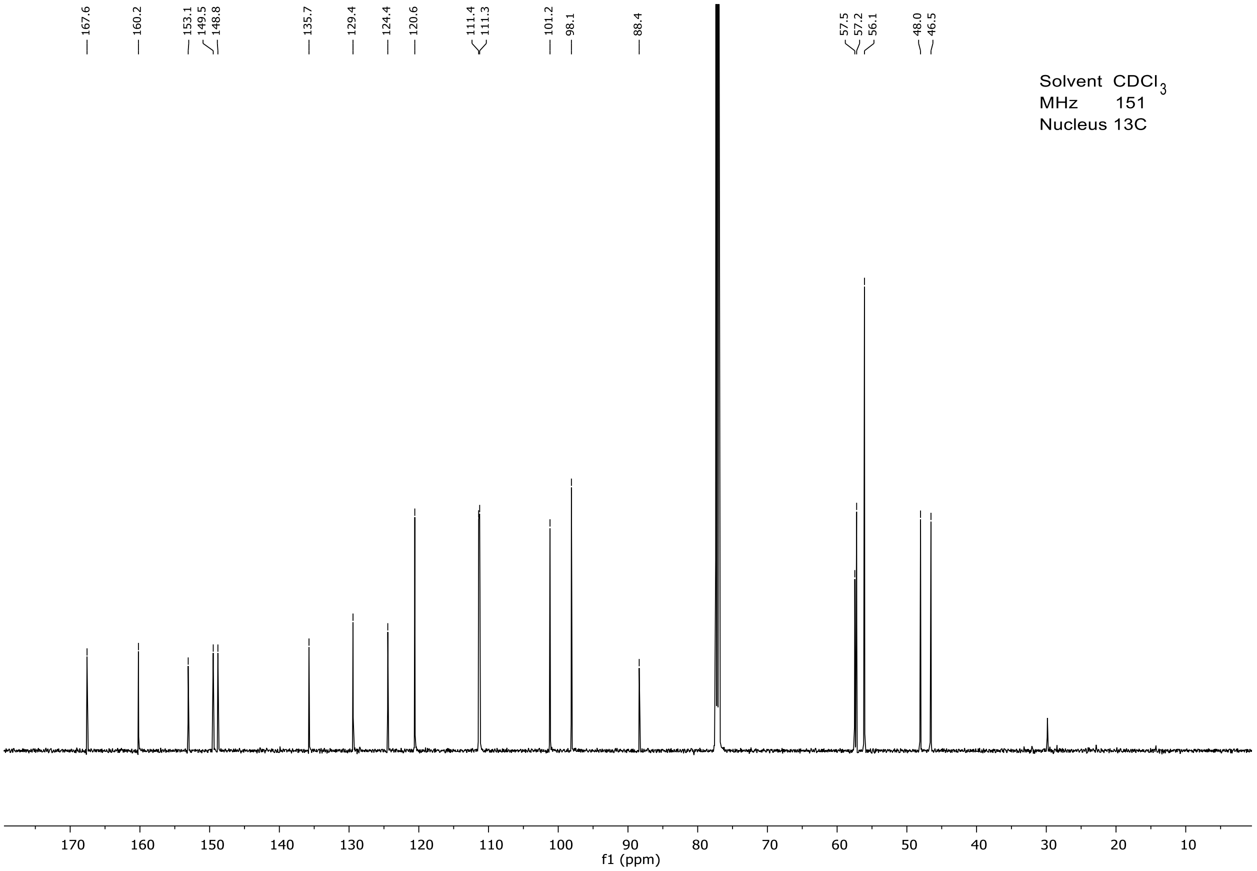

Supplementary Figure 7 ^{1}H and ^{13}C NMR Spectra for **15** in $CDCl_3$.

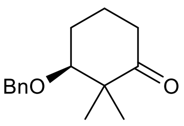

18

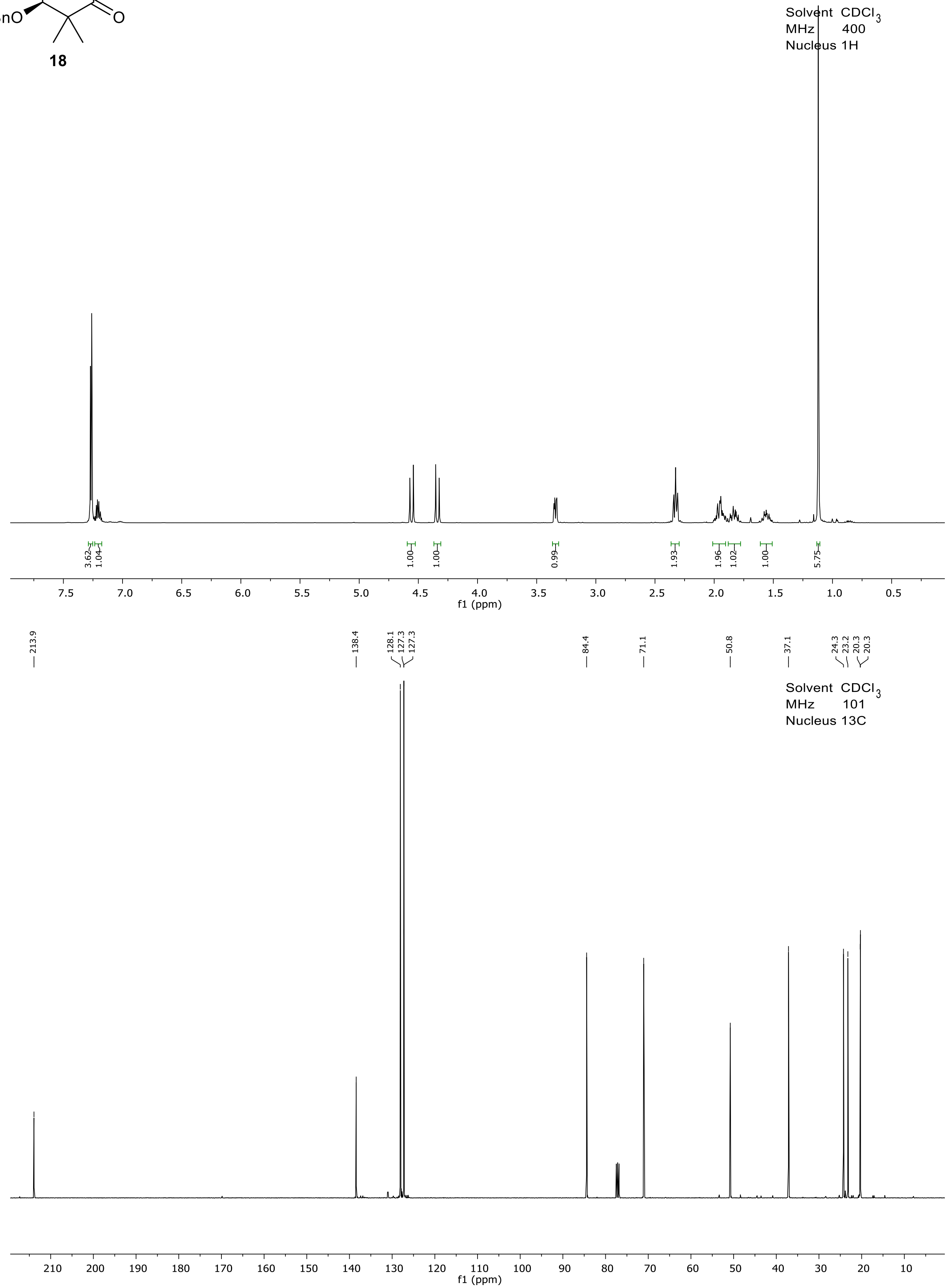

Supplementary Figure 8 ^{1}H and ^{13}C NMR Spectra for **18** in $CDCl_3$.

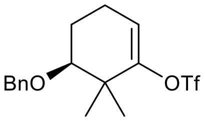

19

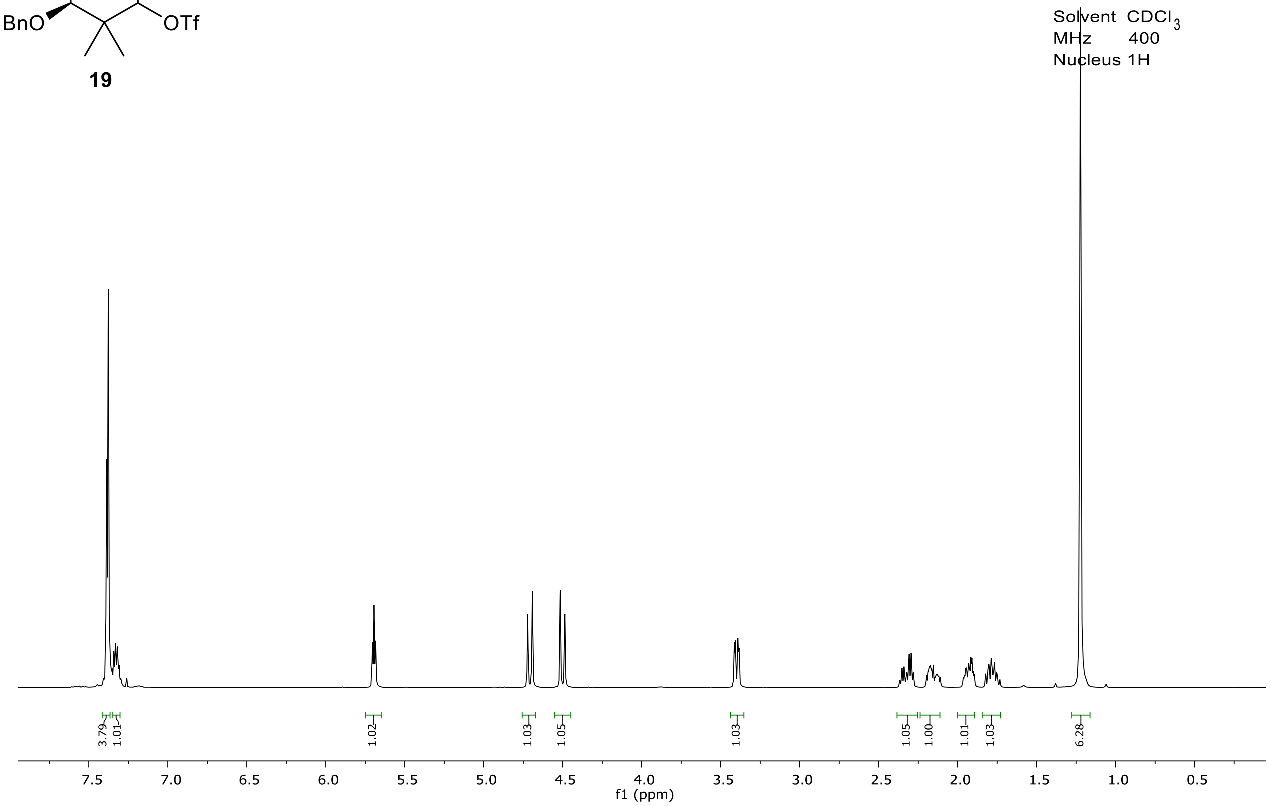

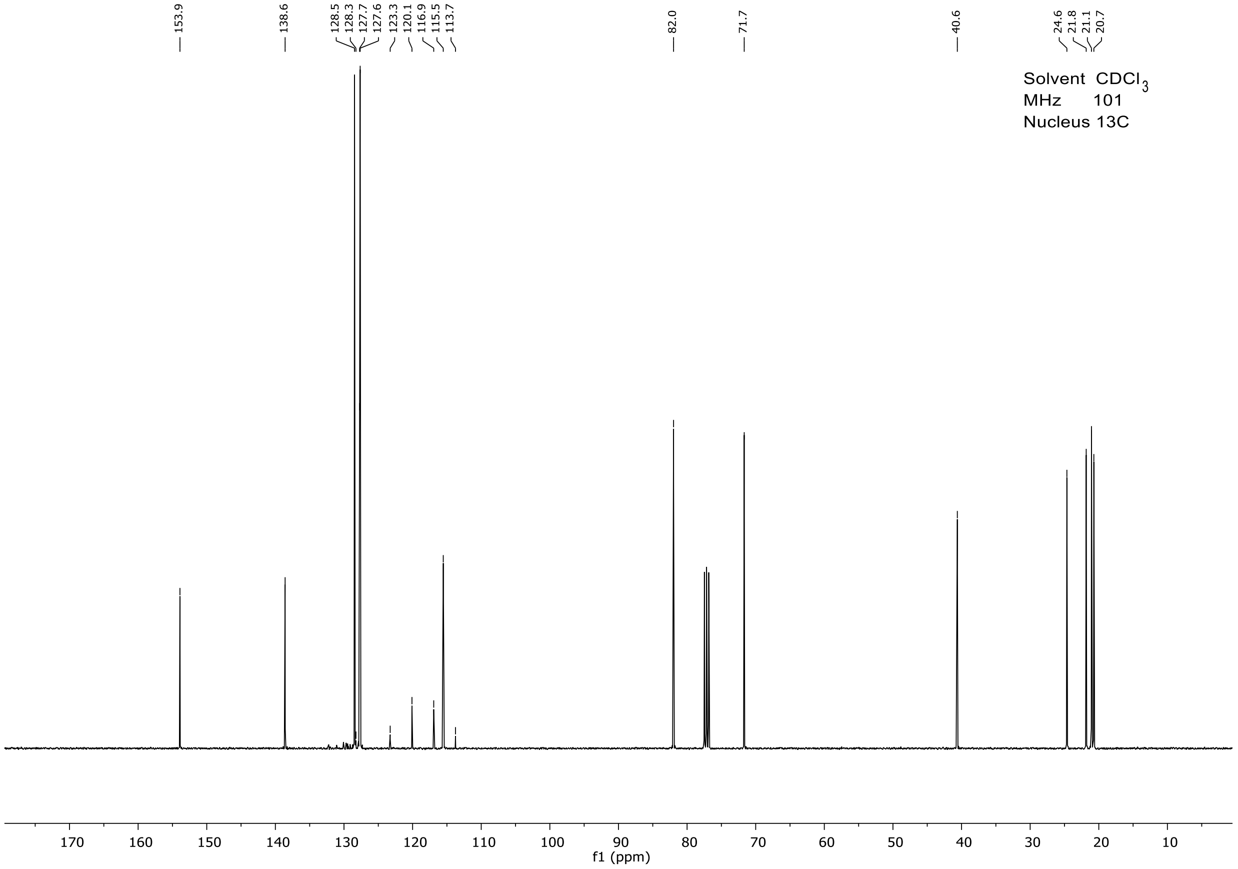

Supplementary Figure 9 ^{1}H and ^{13}C NMR Spectra for **19** in $CDCl_3$.

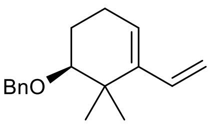

20

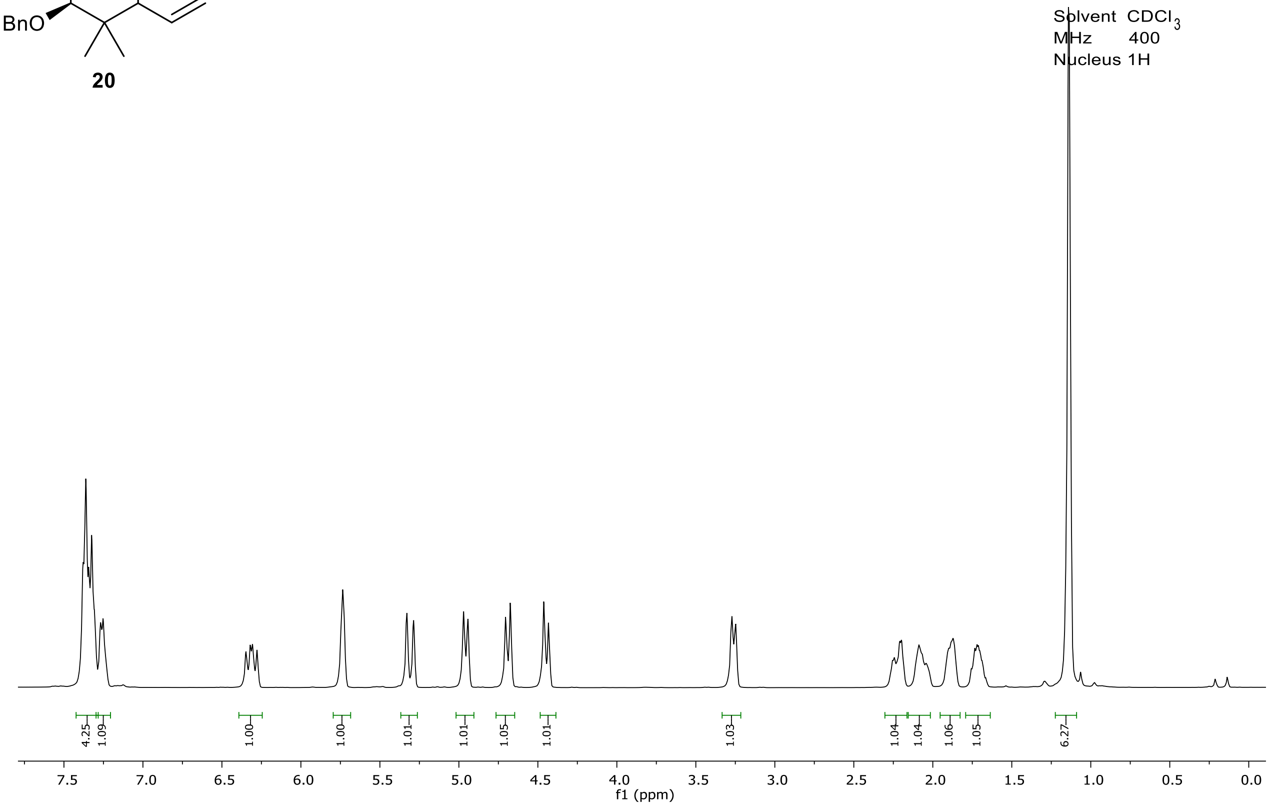

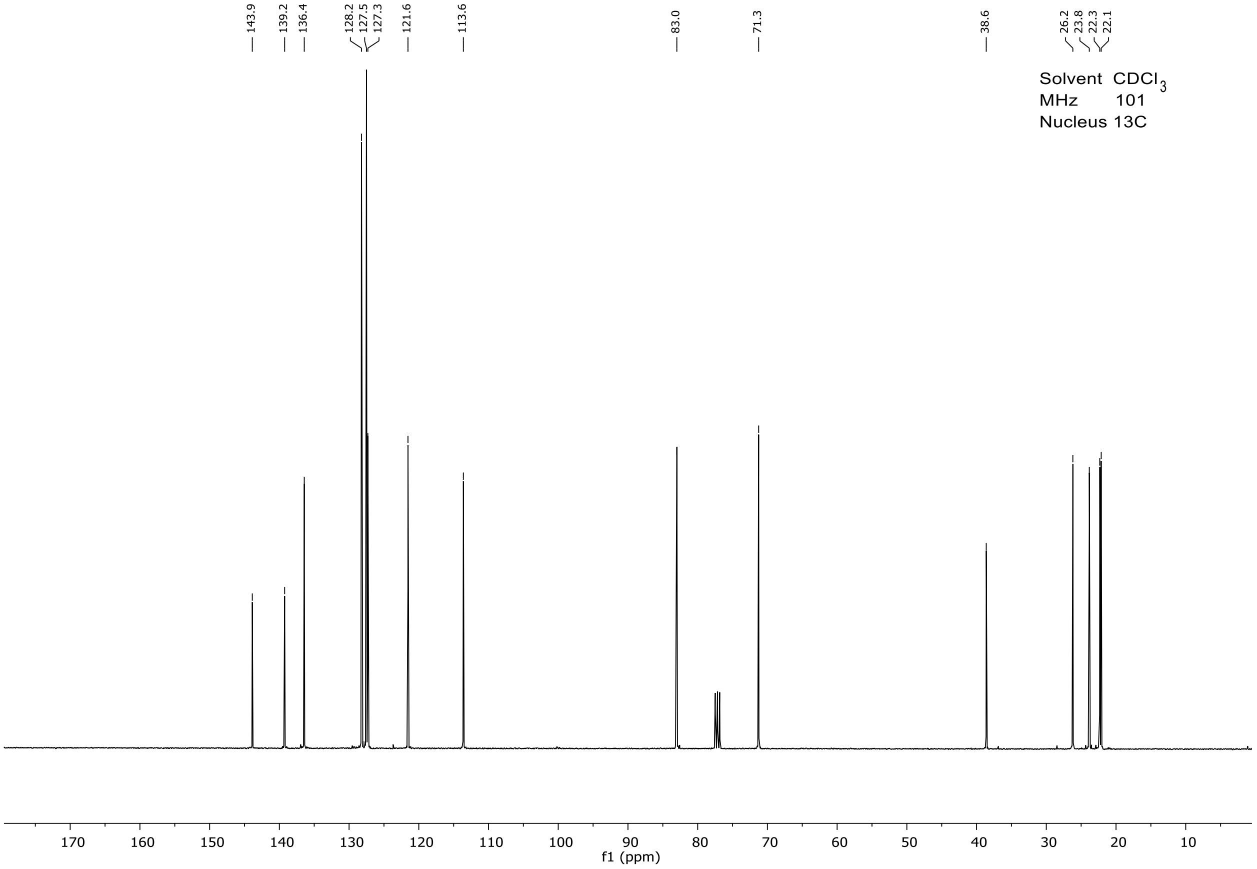

Supplementary Figure 10 ^{1}H and ^{13}C NMR Spectra for **20** in $CDCl_3$.

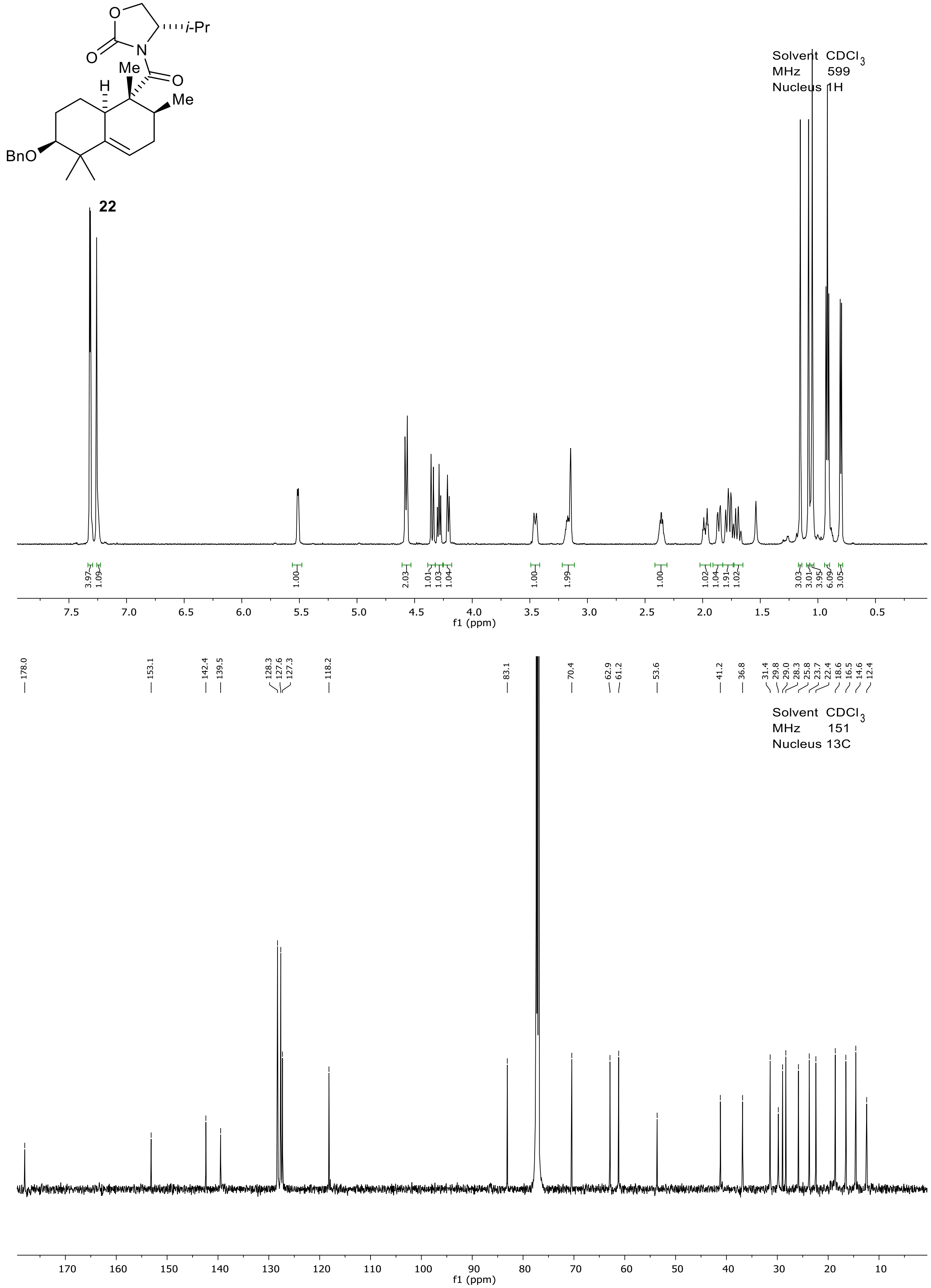

Supplementary Figure 11 ^{1}H and ^{13}C NMR Spectra for **22** in $CDCl_3$.

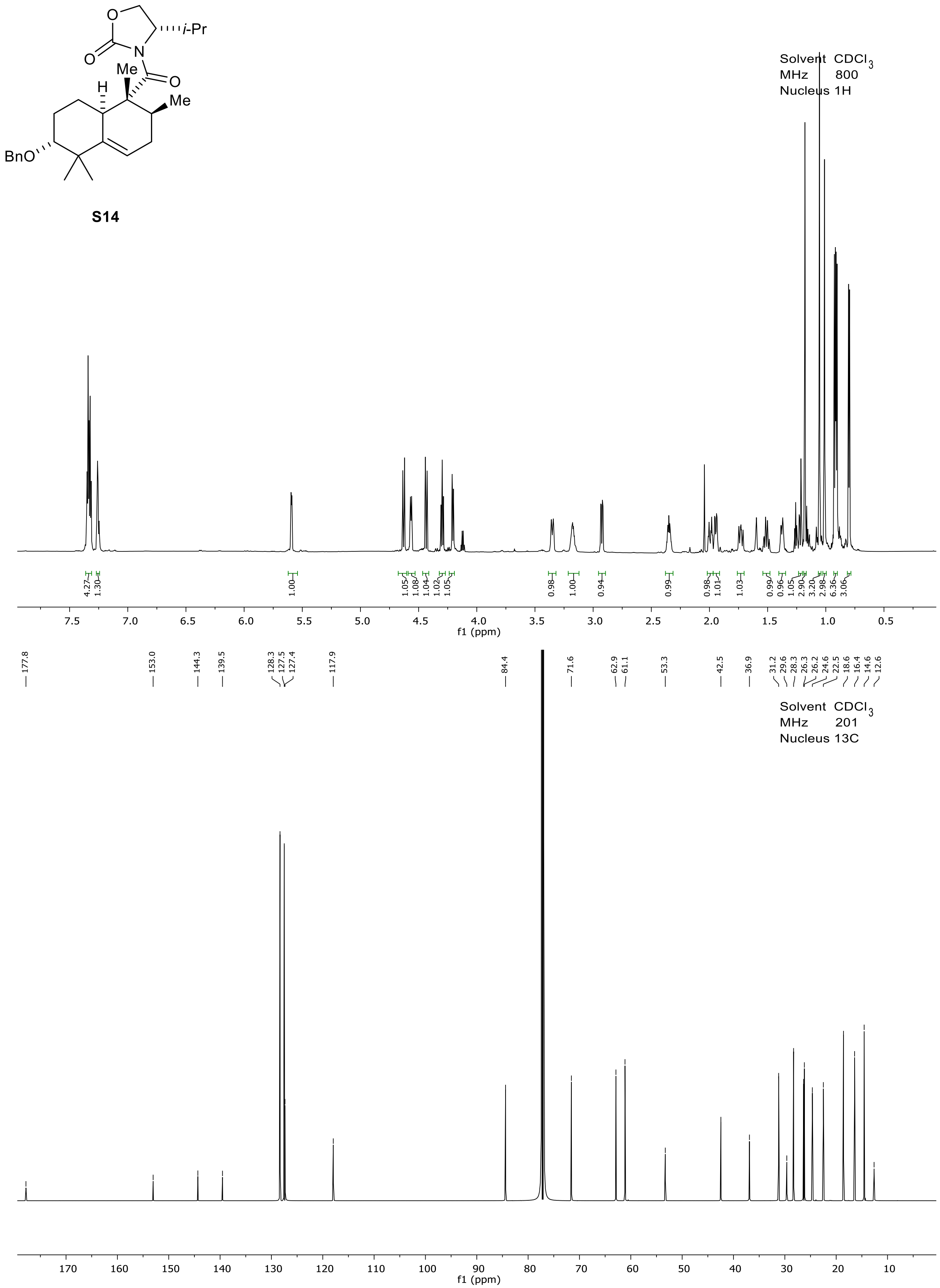

Supplementary Figure 12 ^{1}H and ^{13}C NMR Spectra for **S14** in $CDCl_3$.

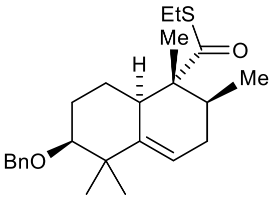

23

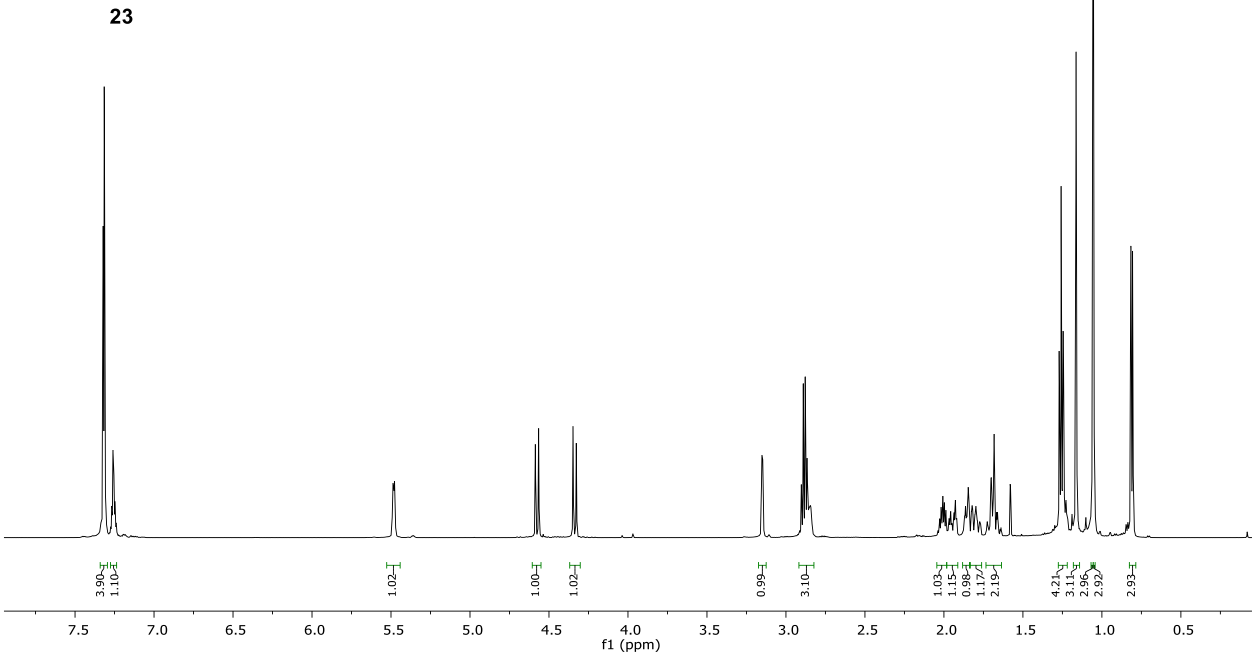

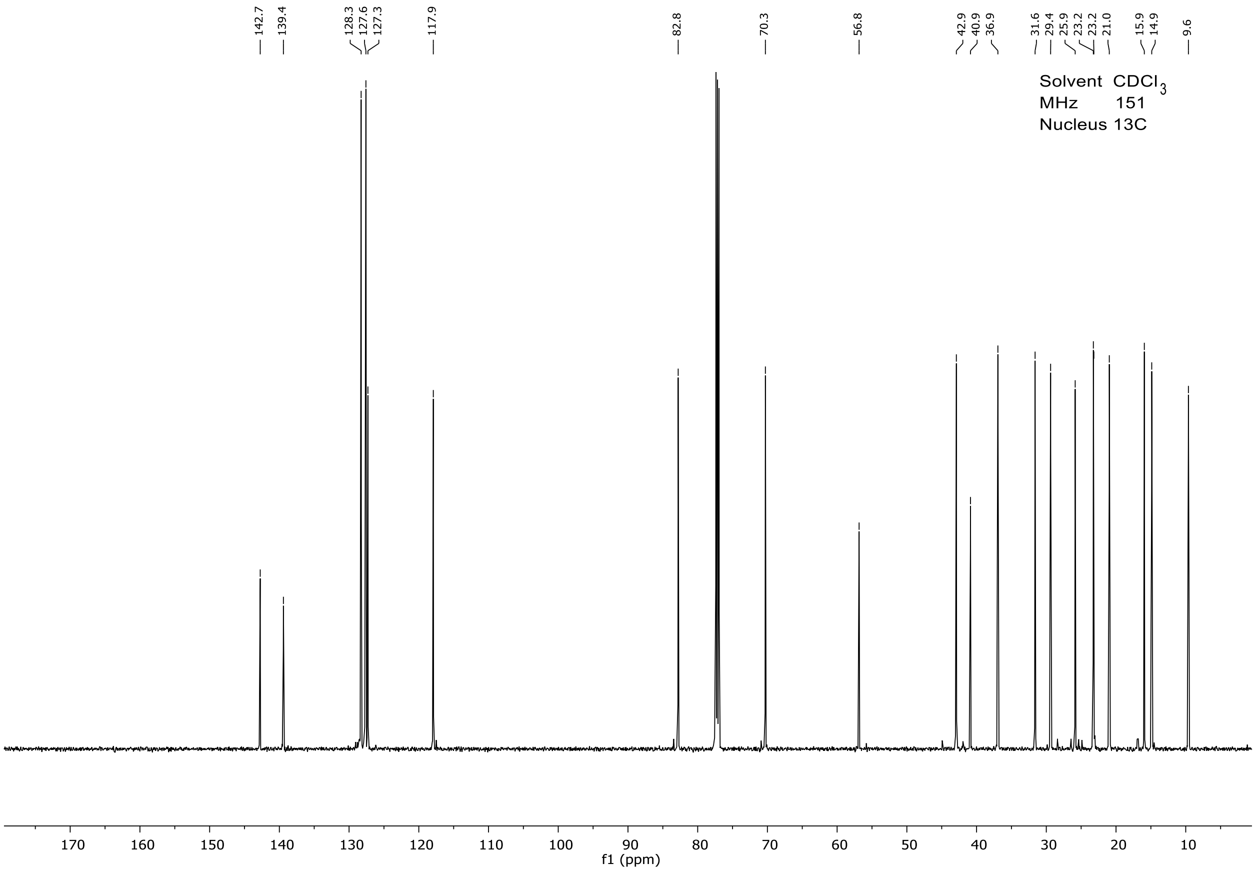

Supplementary Figure 13 ^{1}H and ^{13}C NMR Spectra for **23** in $CDCl_3$.

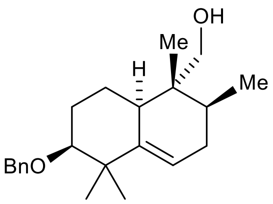

24

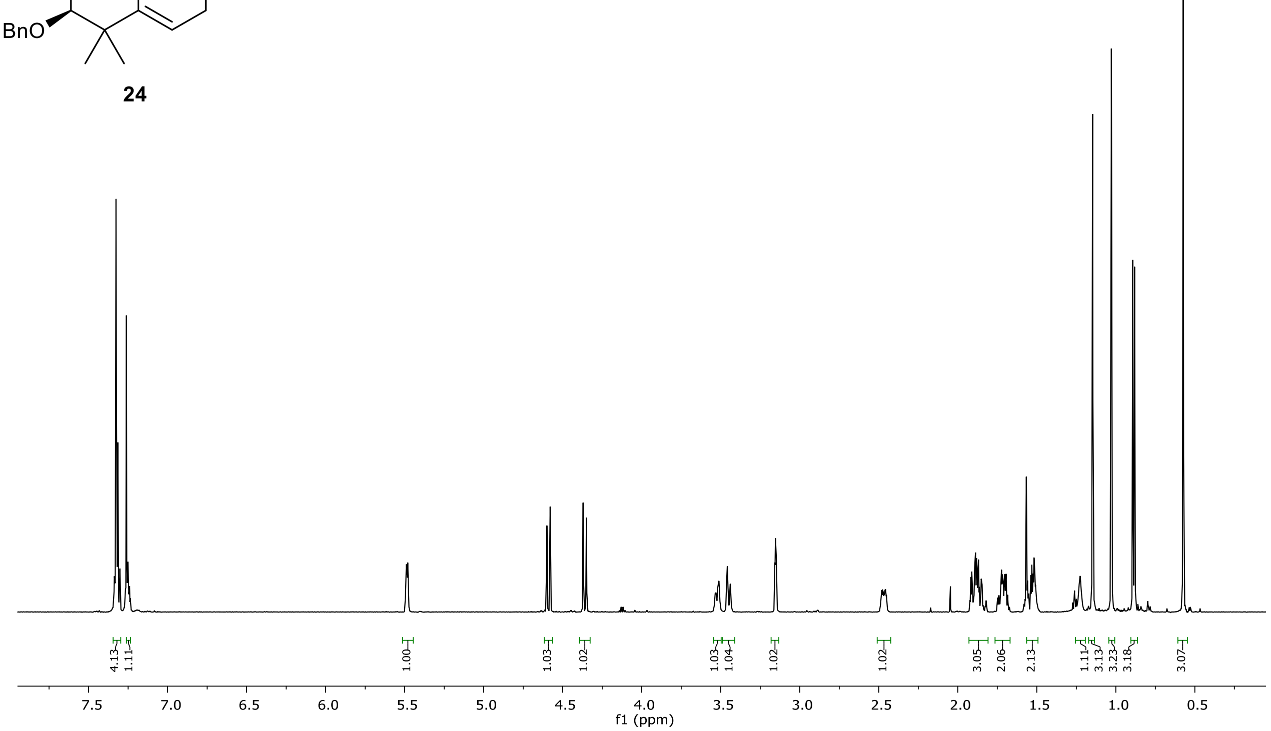

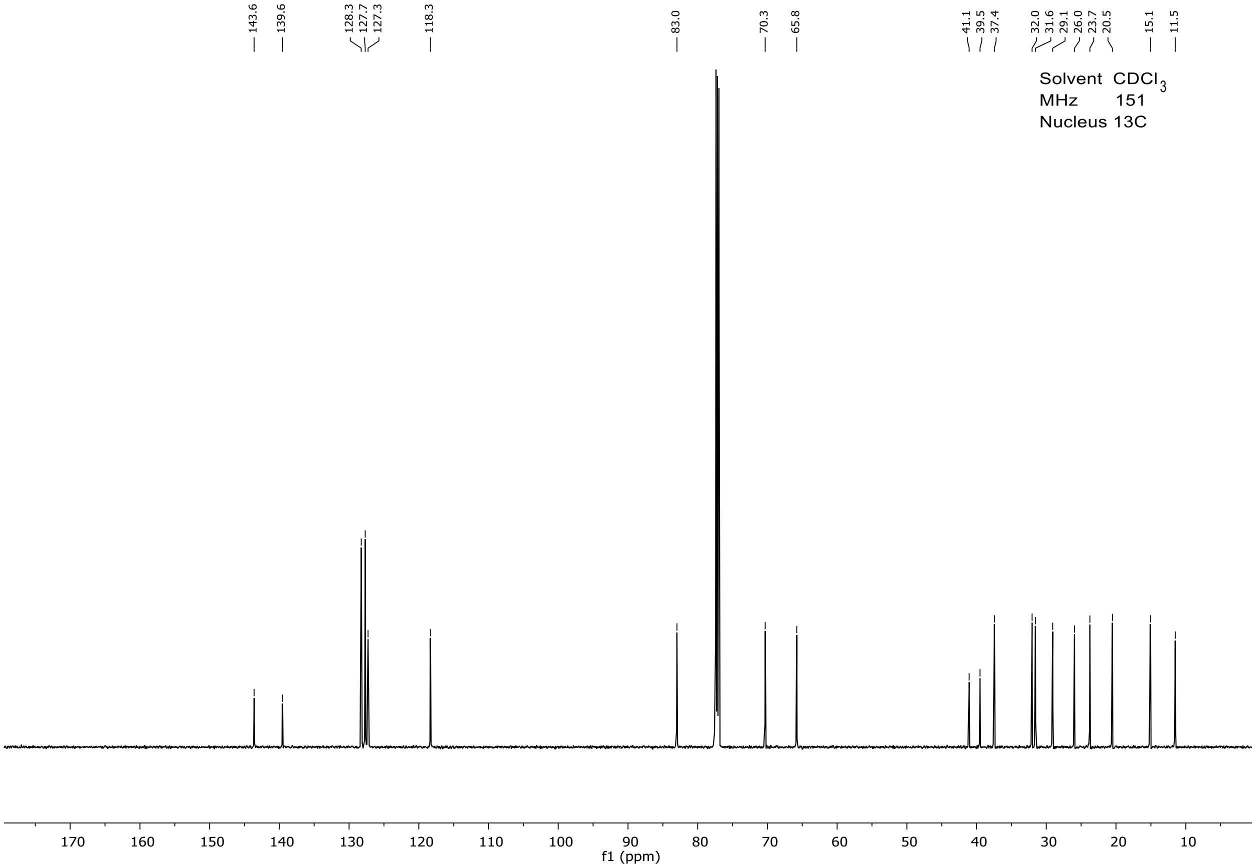

Supplementary Figure 14 ^{1}H and ^{13}C NMR Spectra for **24** in $CDCl_3$.

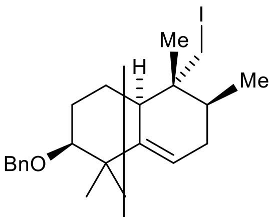

25

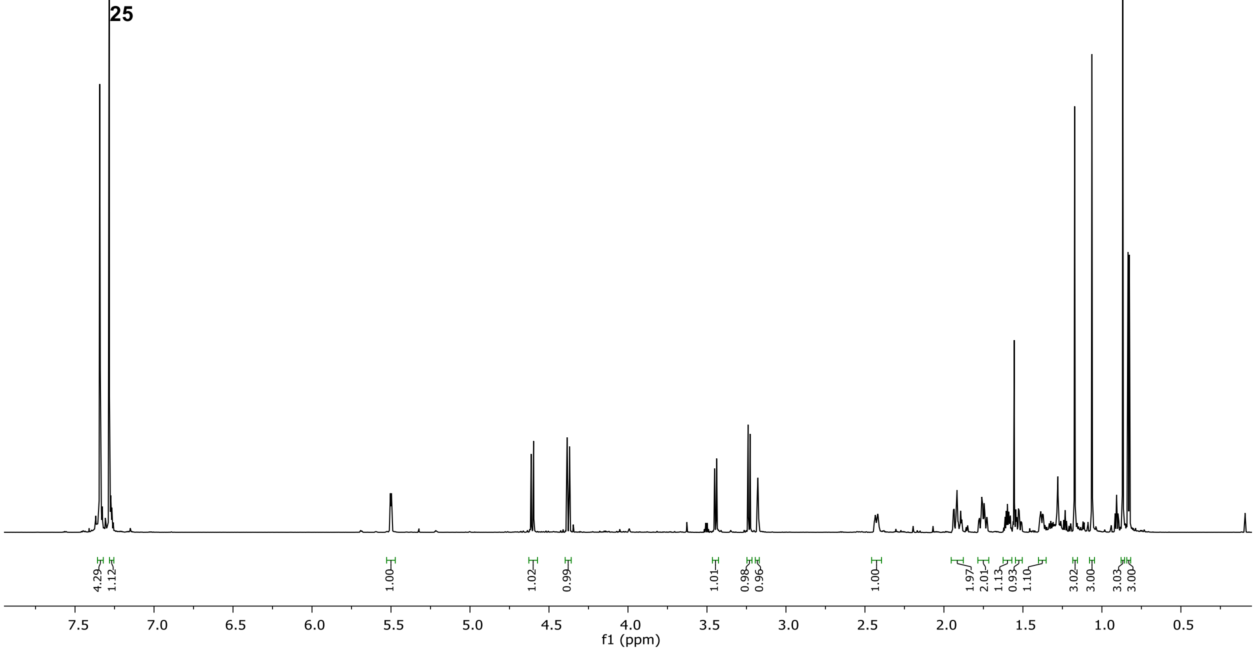

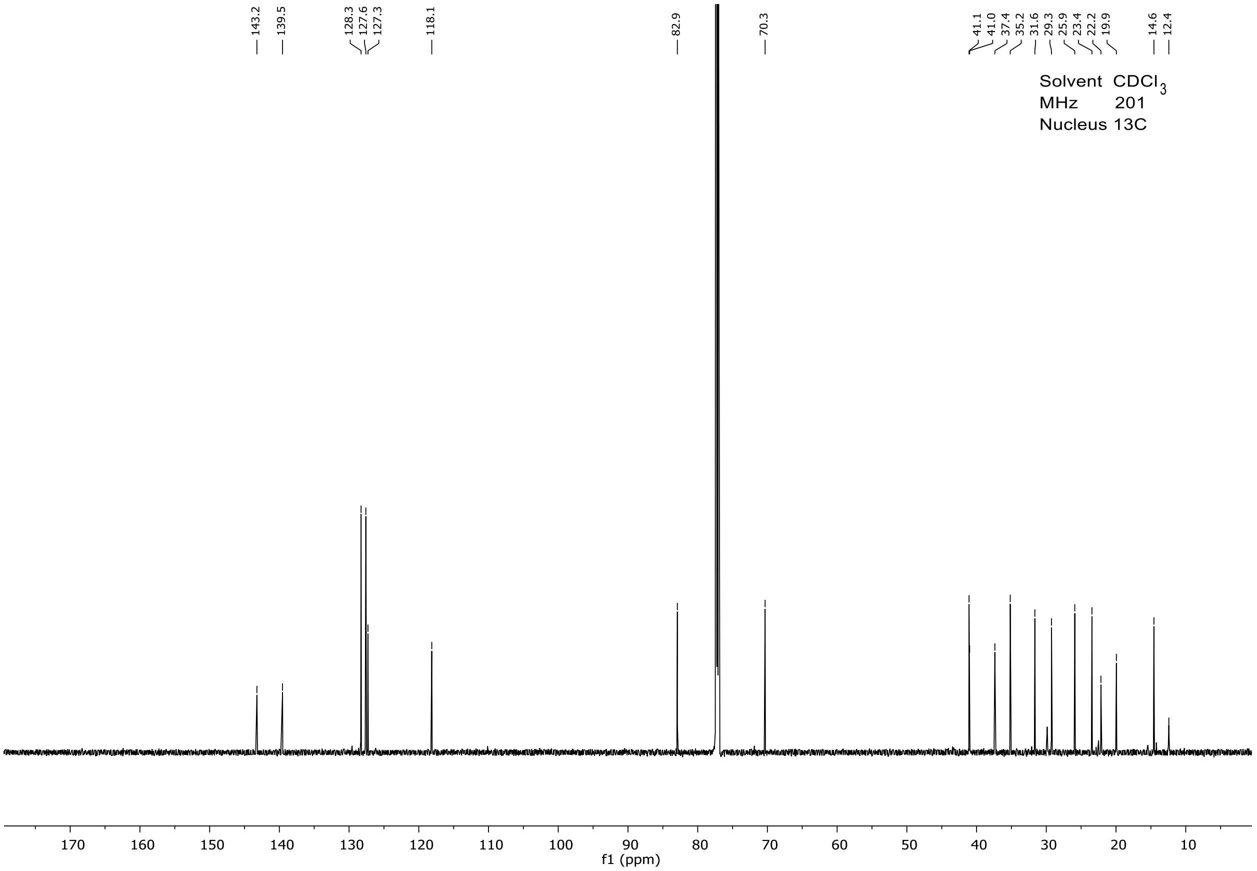

Supplementary Figure 15 ^{1}H and ^{13}C NMR Spectra for **25** in $CDCl_3$.

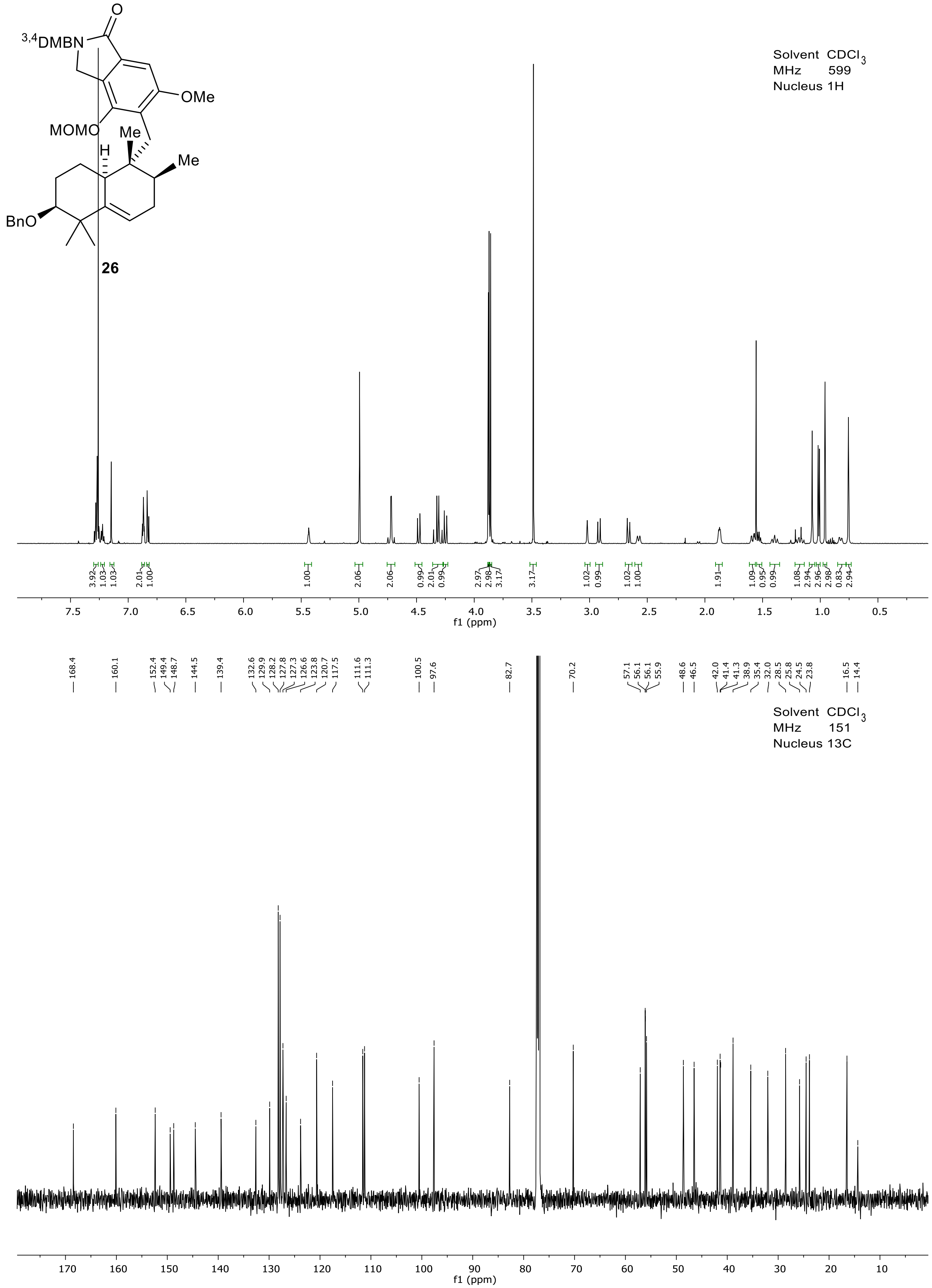

Supplementary Figure 16 ^{1}H and ^{13}C NMR Spectra for **26** in $CDCl_3$.

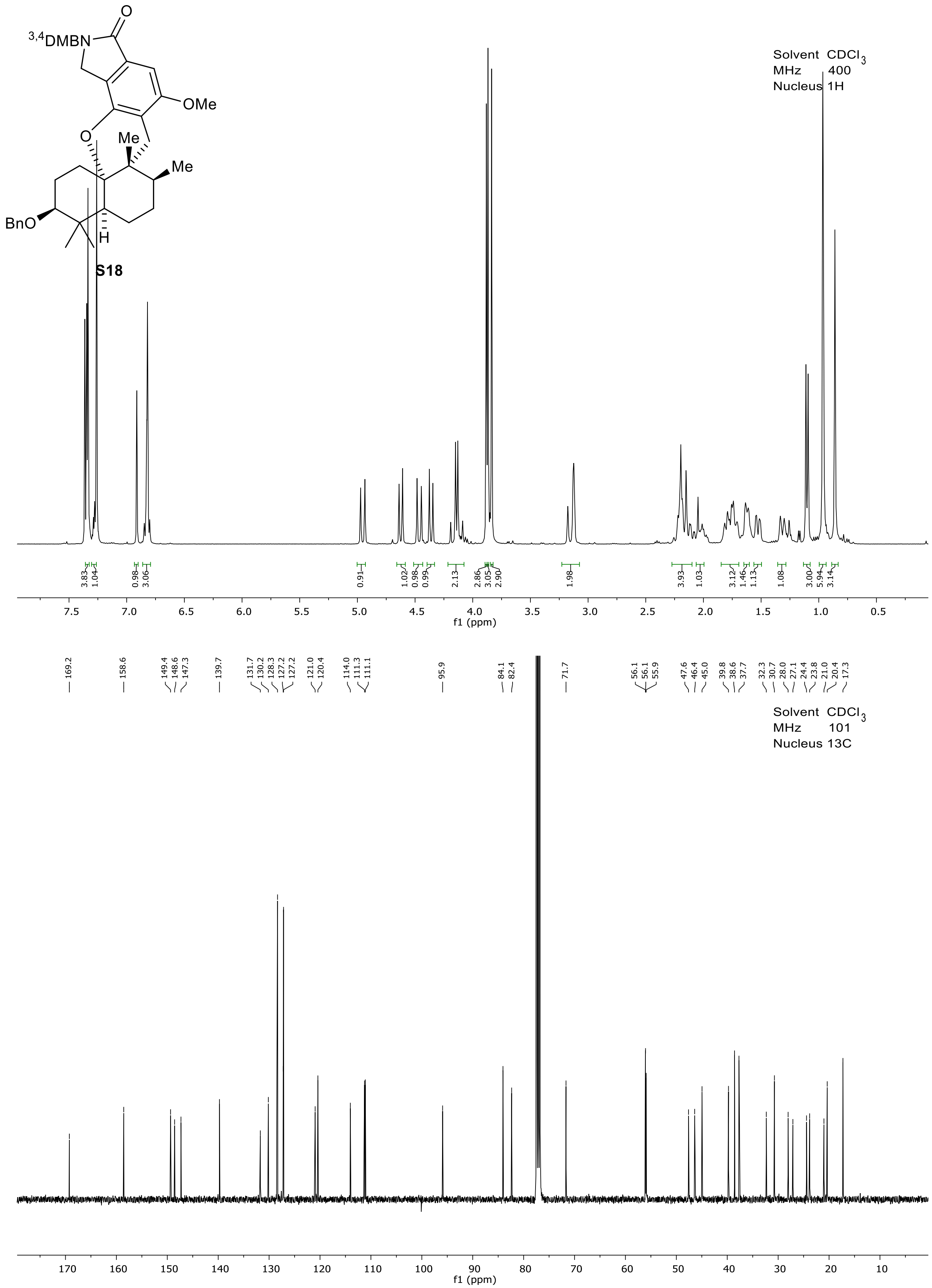

Supplementary Figure 17 ^{1}H and ^{13}C NMR Spectra for **S18** in $CDCl_3$.

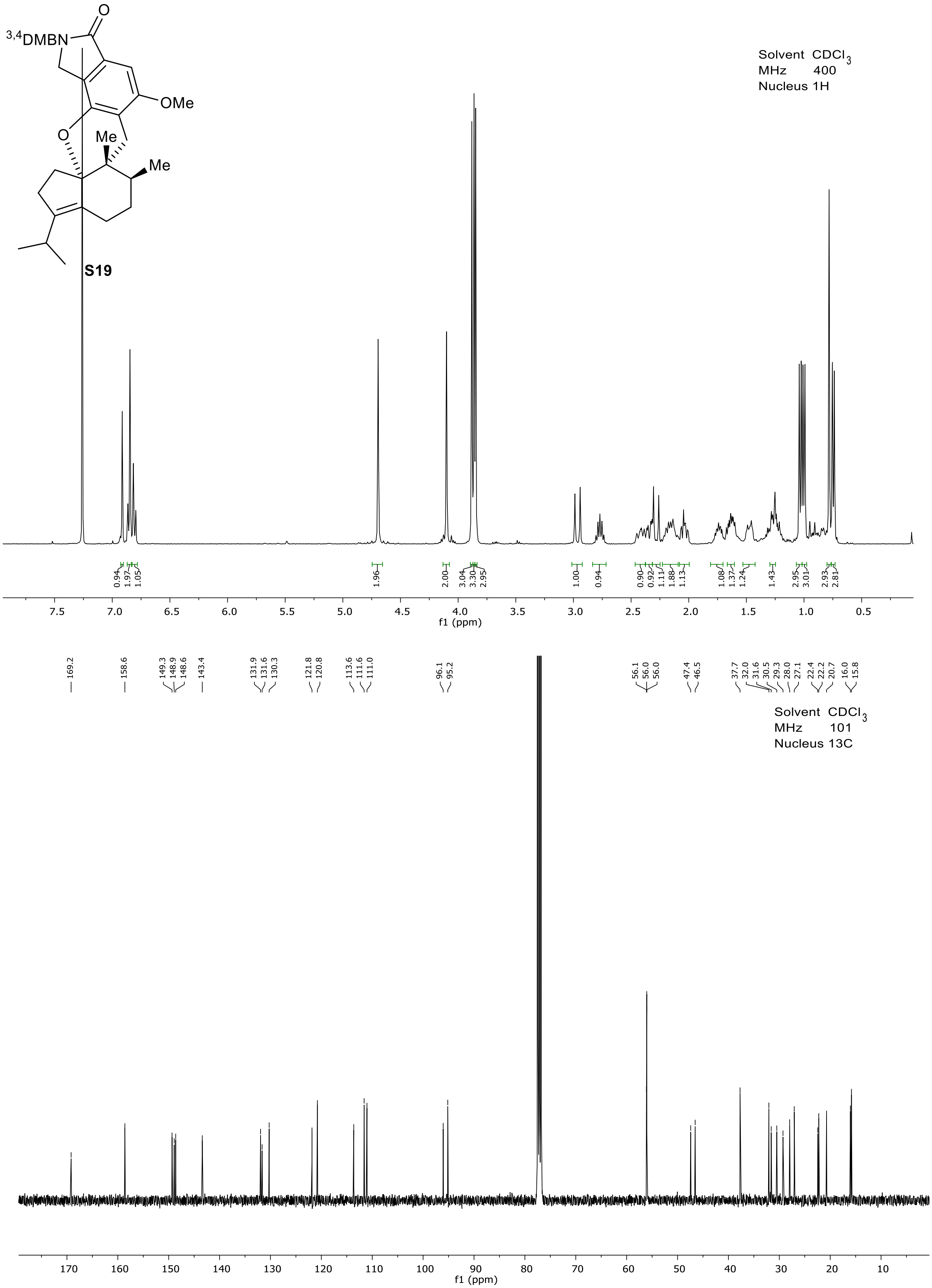

Supplementary Figure 18 ^{1}H and ^{13}C NMR Spectra for **S19** in $CDCl_3$.

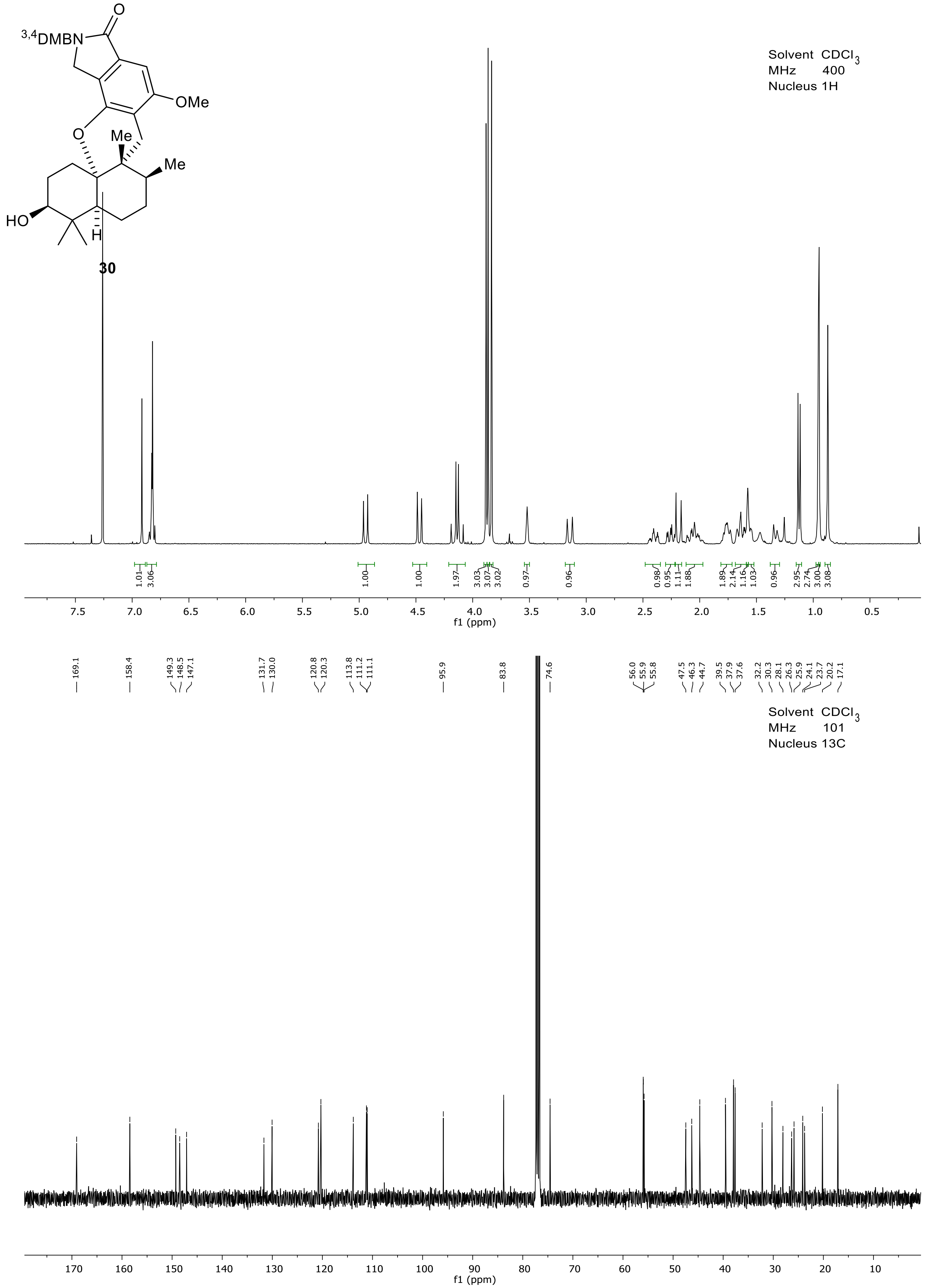

Supplementary Figure 19 ^{1}H and ^{13}C NMR Spectra for **30** in $CDCl_3$.

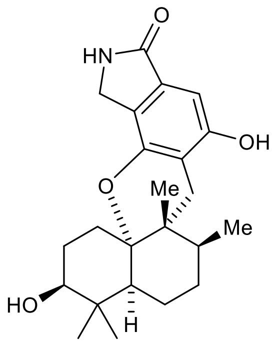

(+)-stachyflin (**1**)

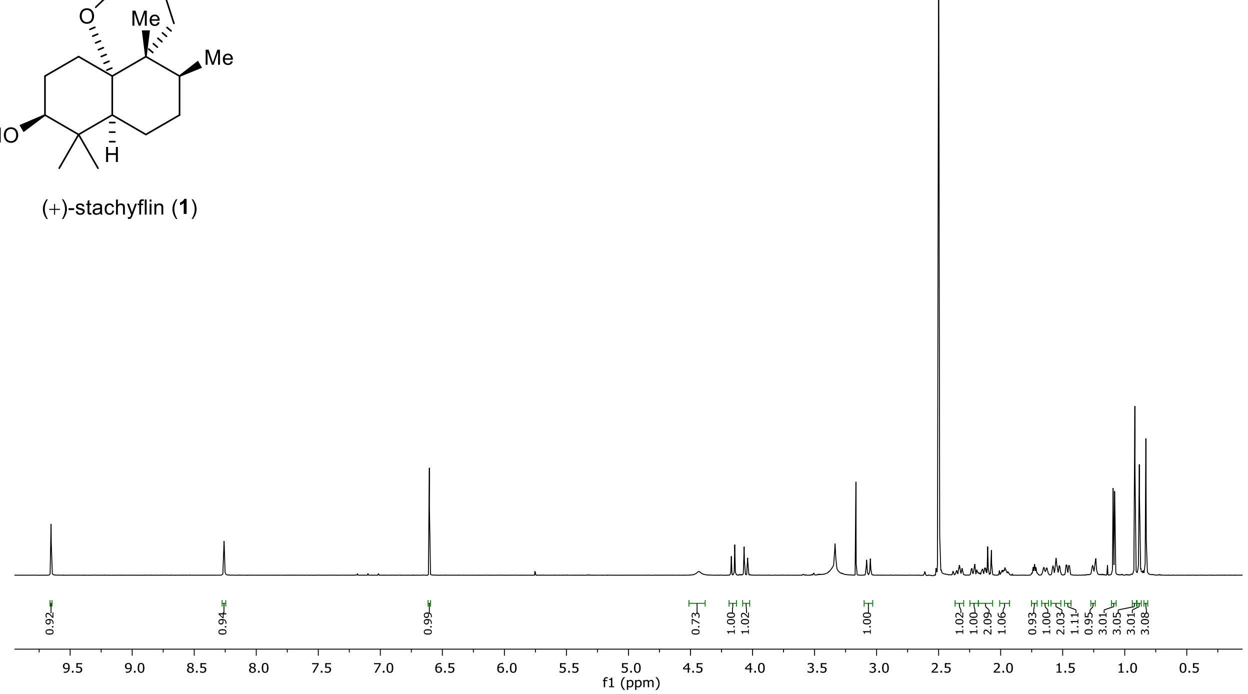

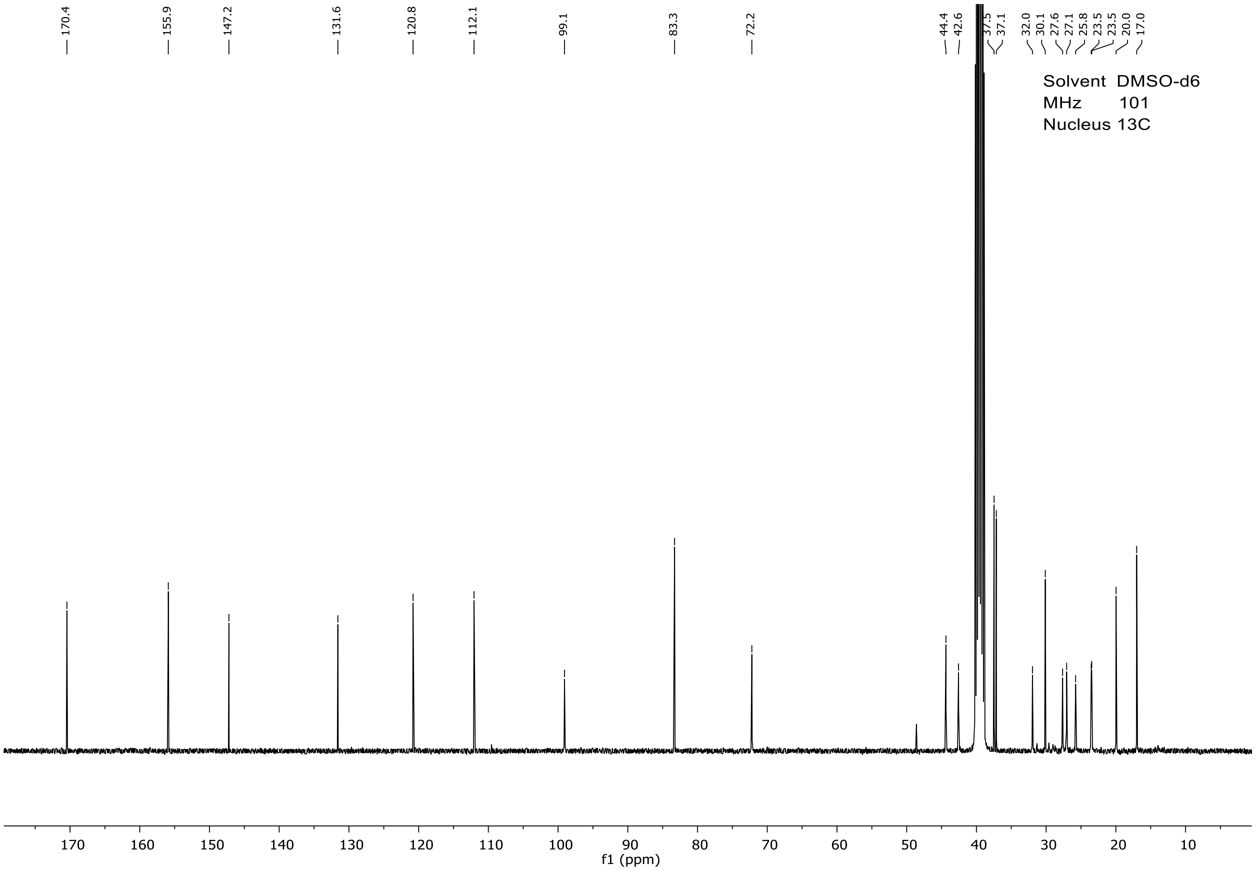

Supplementary Figure 20 ^{1}H and ^{13}C NMR Spectra for **1** in DMSO-d6.

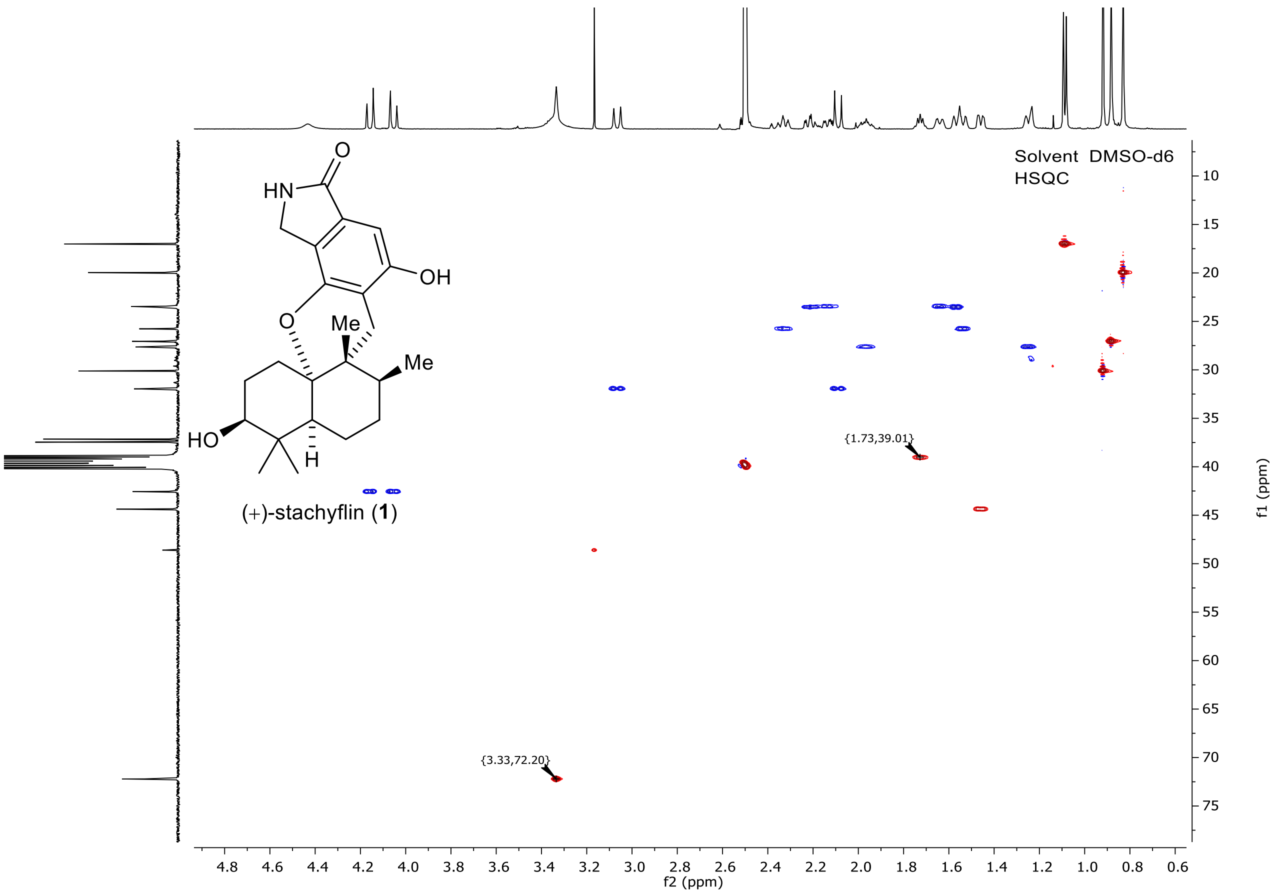

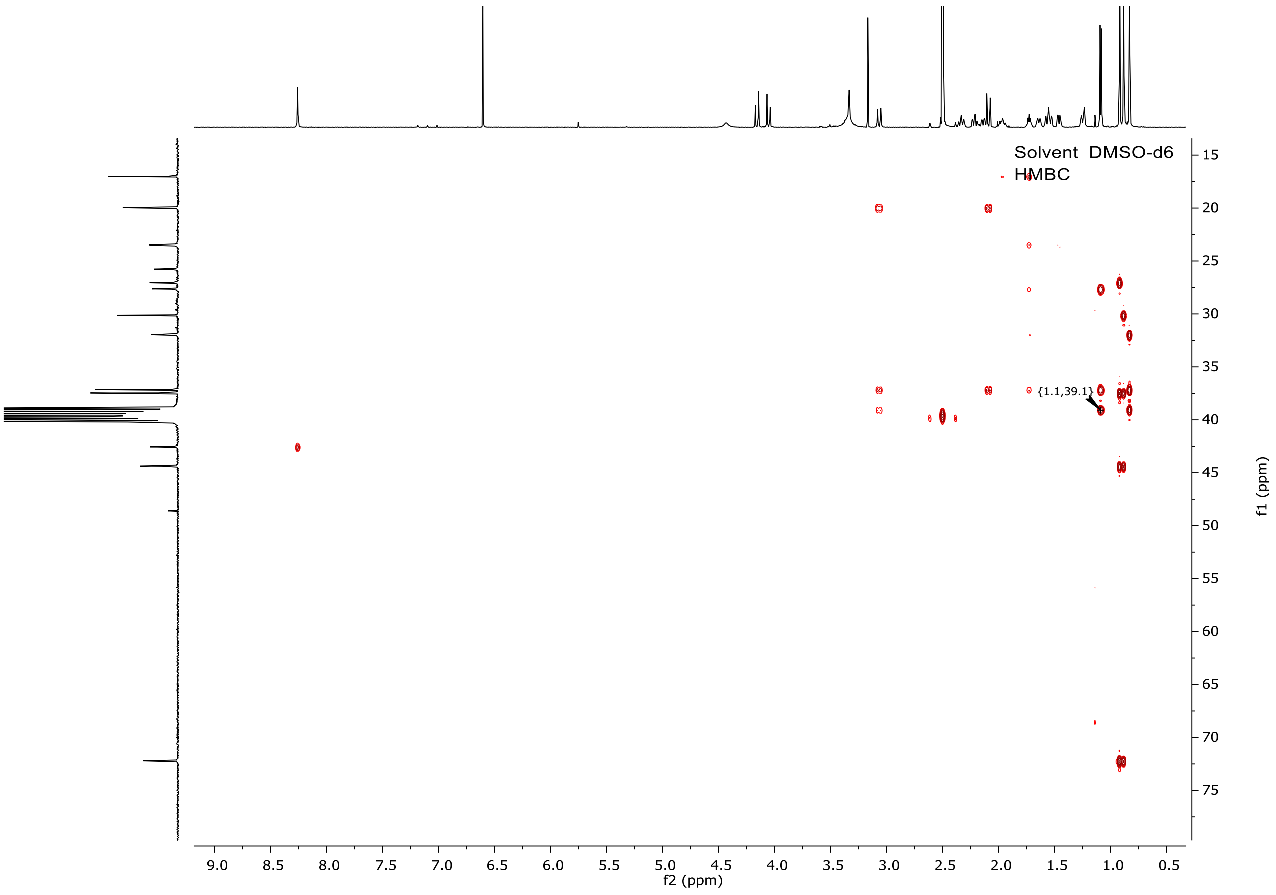

Supplementary Figure 21 HSQC and HMBC NMR Spectra for **1** in DMSO-d6.

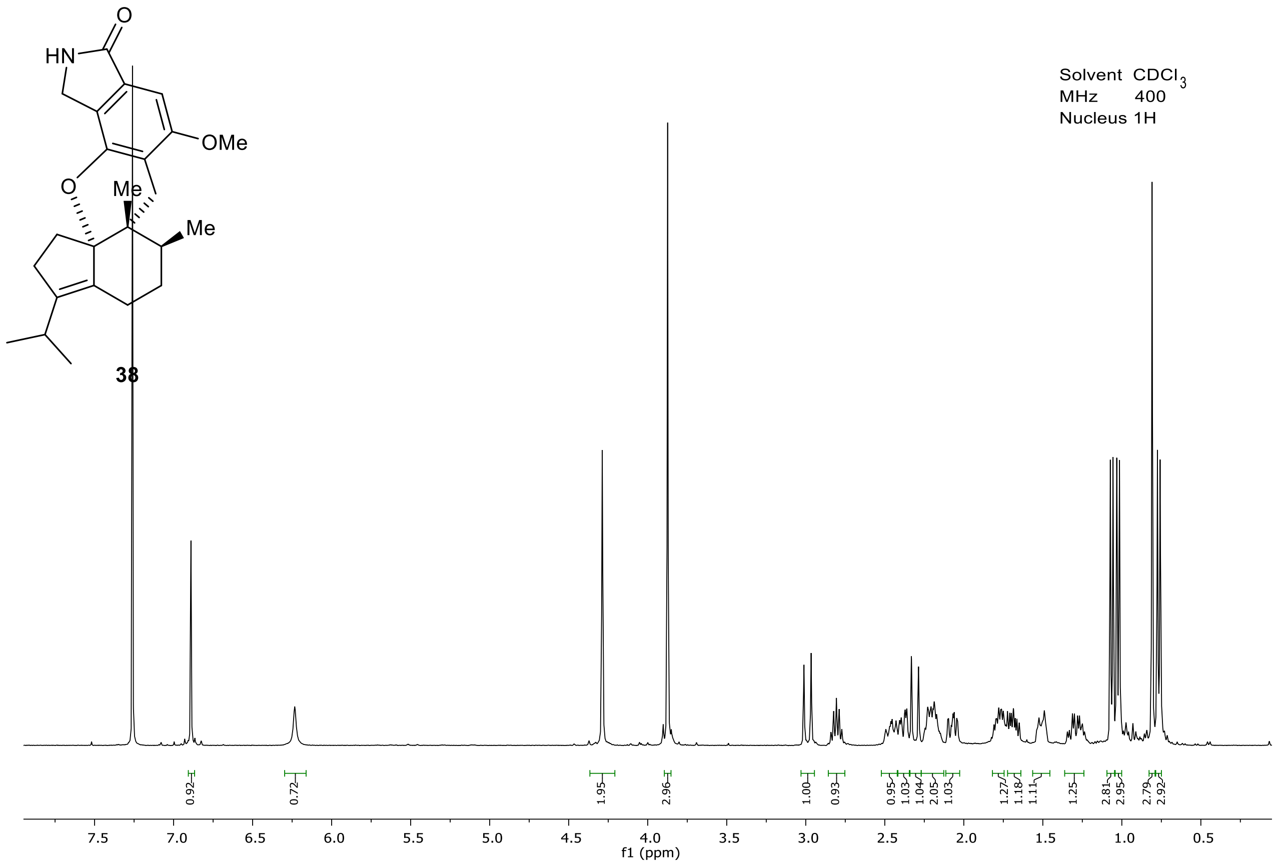

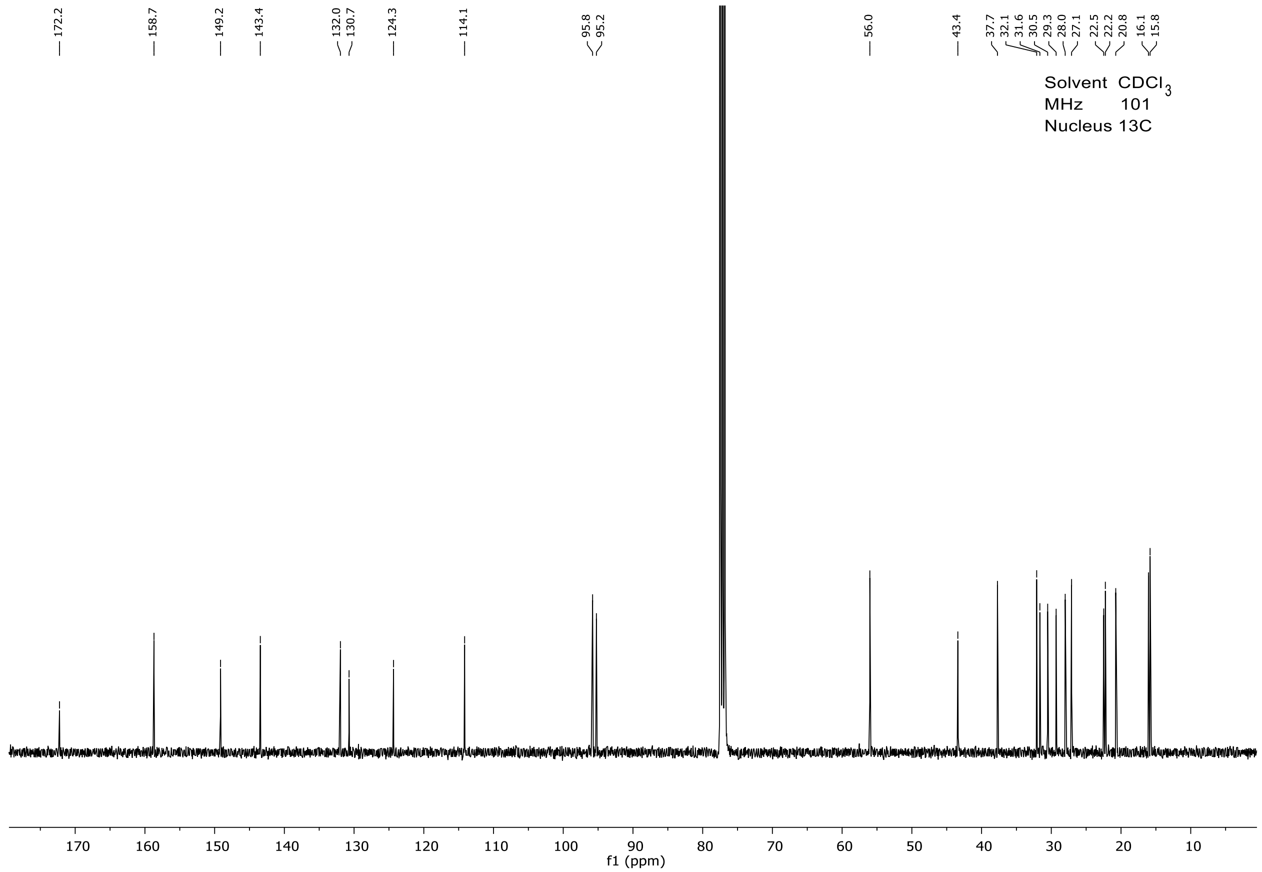

Supplementary Figure 22 ^{1}H and ^{13}C NMR Spectra for **38** in $CDCl_3$.

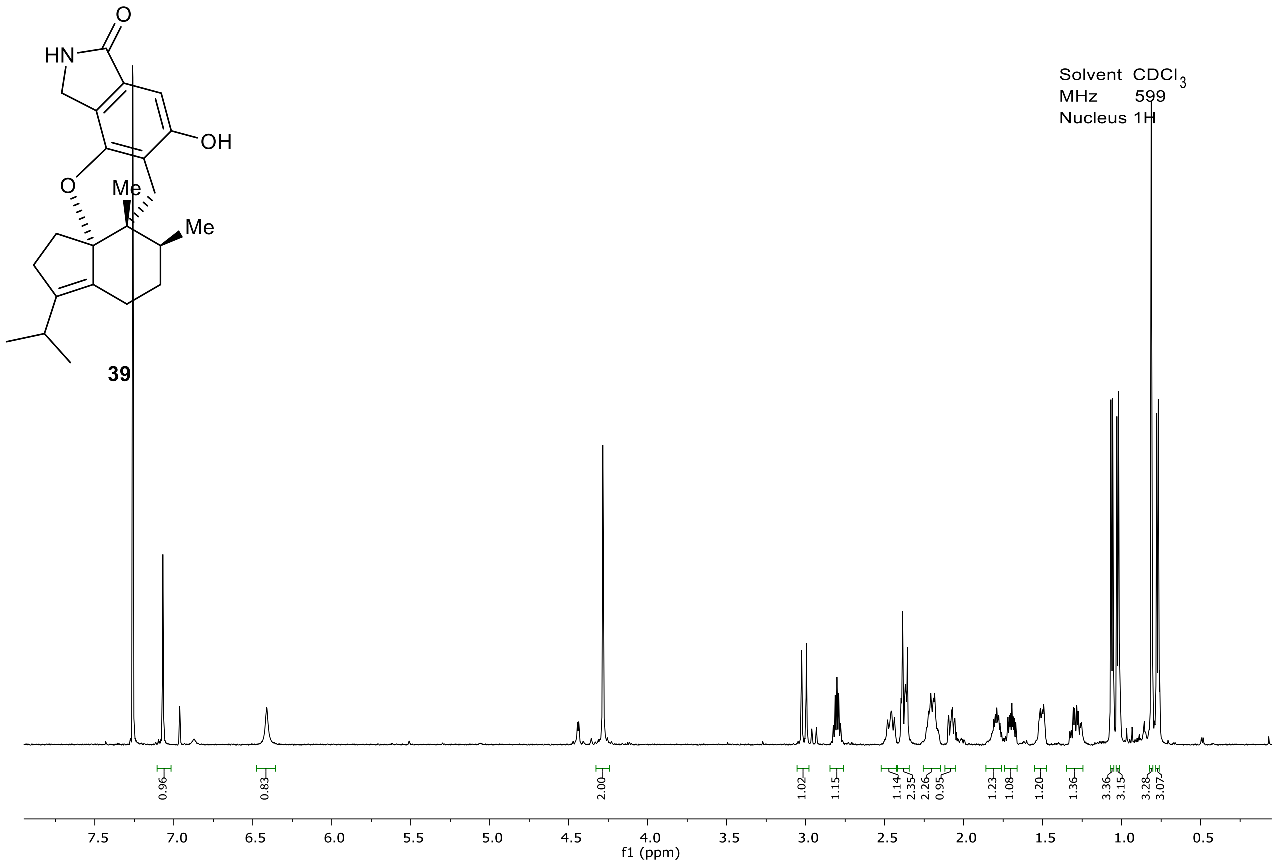

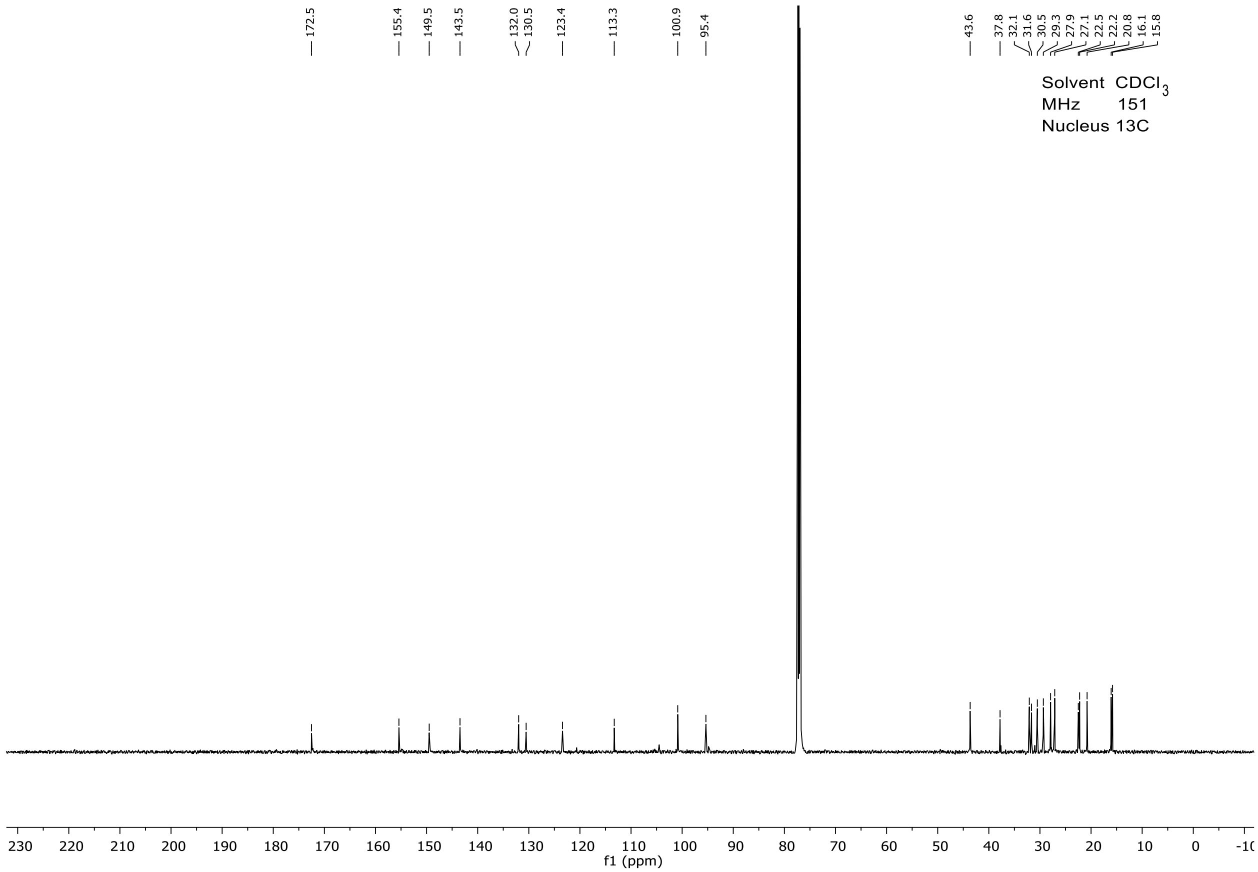

Supplementary Figure 23 ^{1}H and ^{13}C NMR Spectra for **39** in $CDCl_3$.

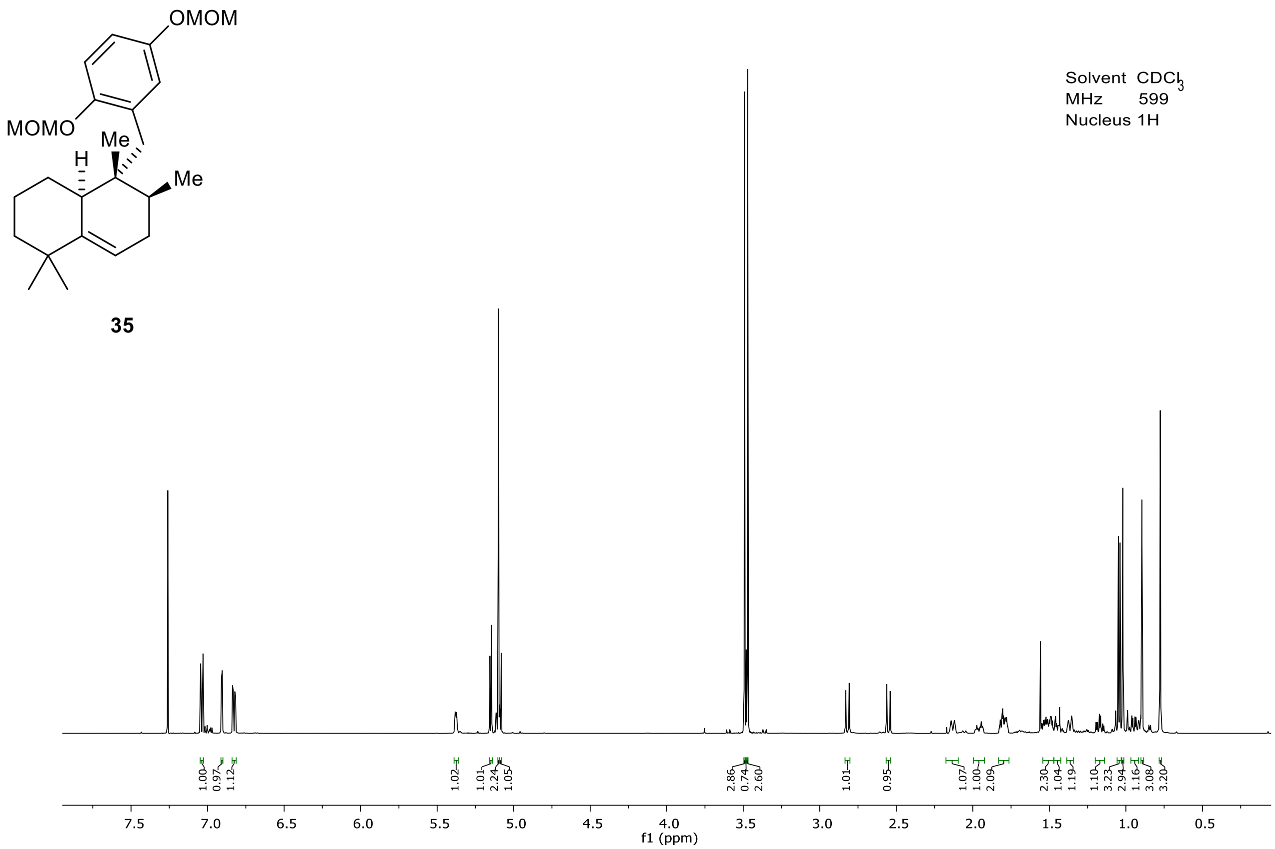

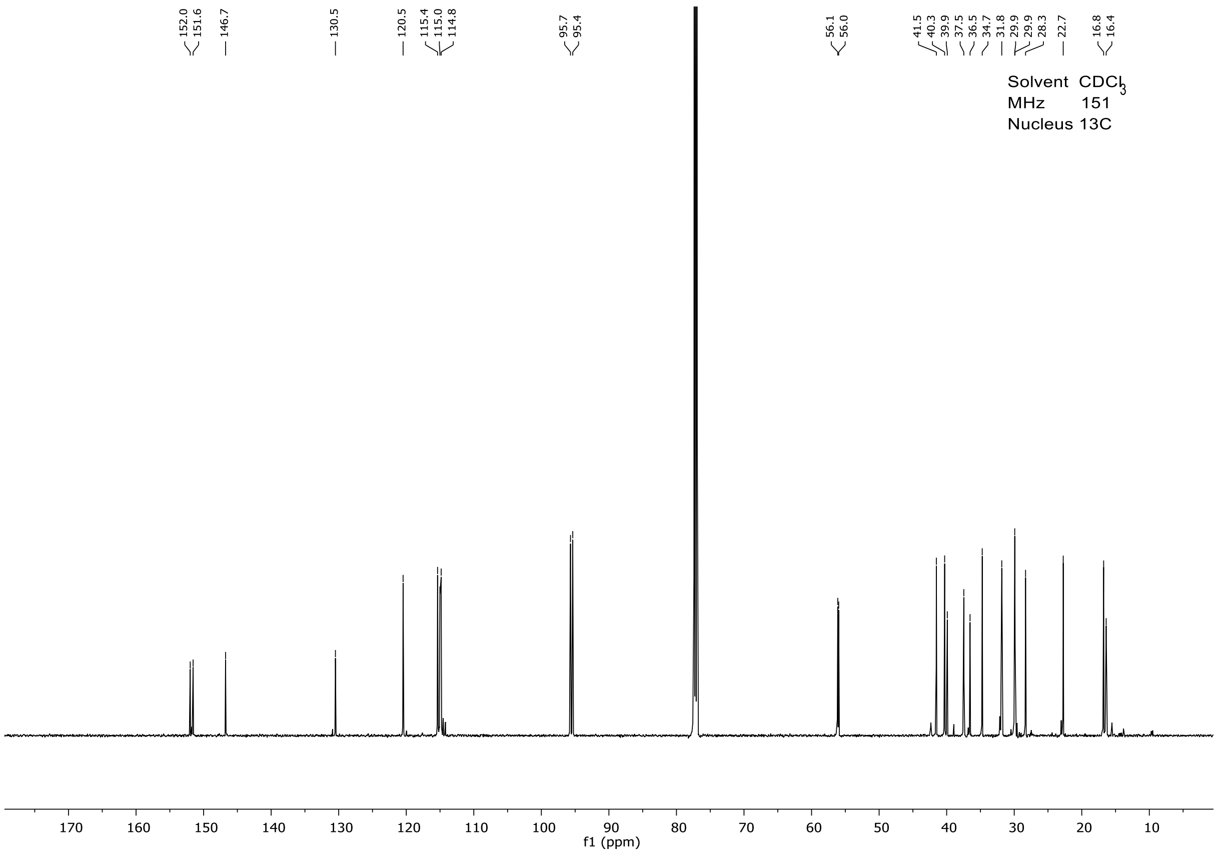

Supplementary Figure 24 ^{1}H and ^{13}C NMR Spectra for **35** in $CDCl_3$.

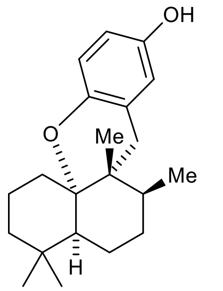

(+)-aureol (**2**)

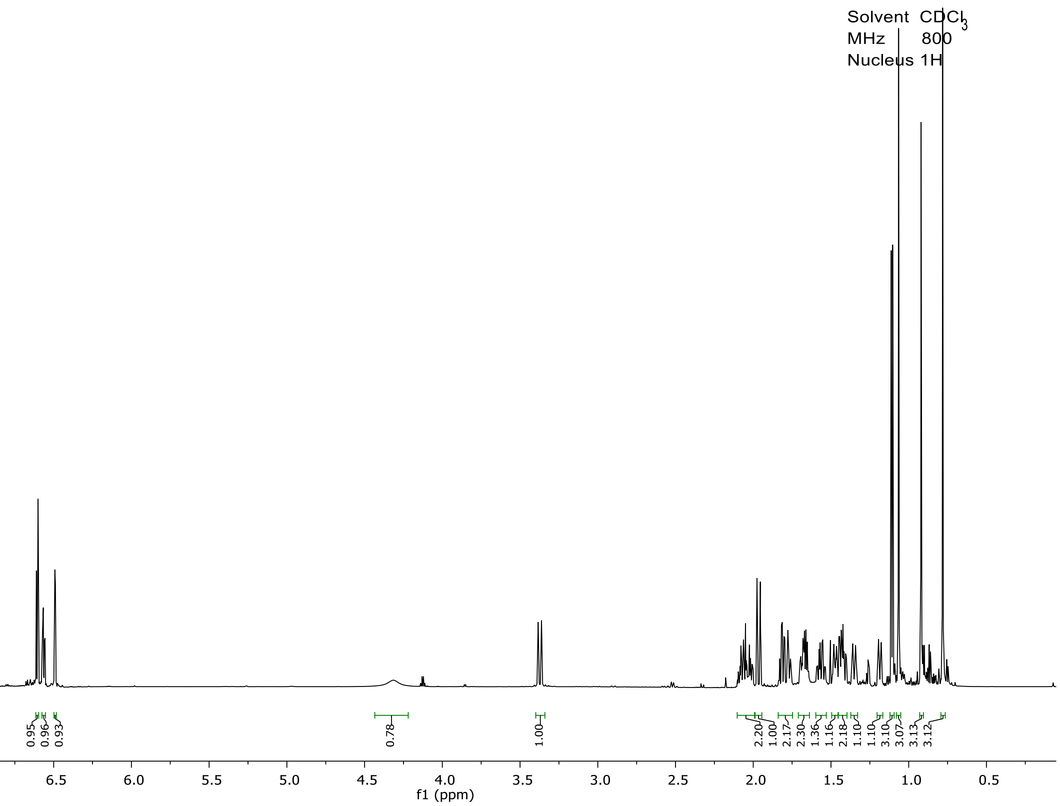

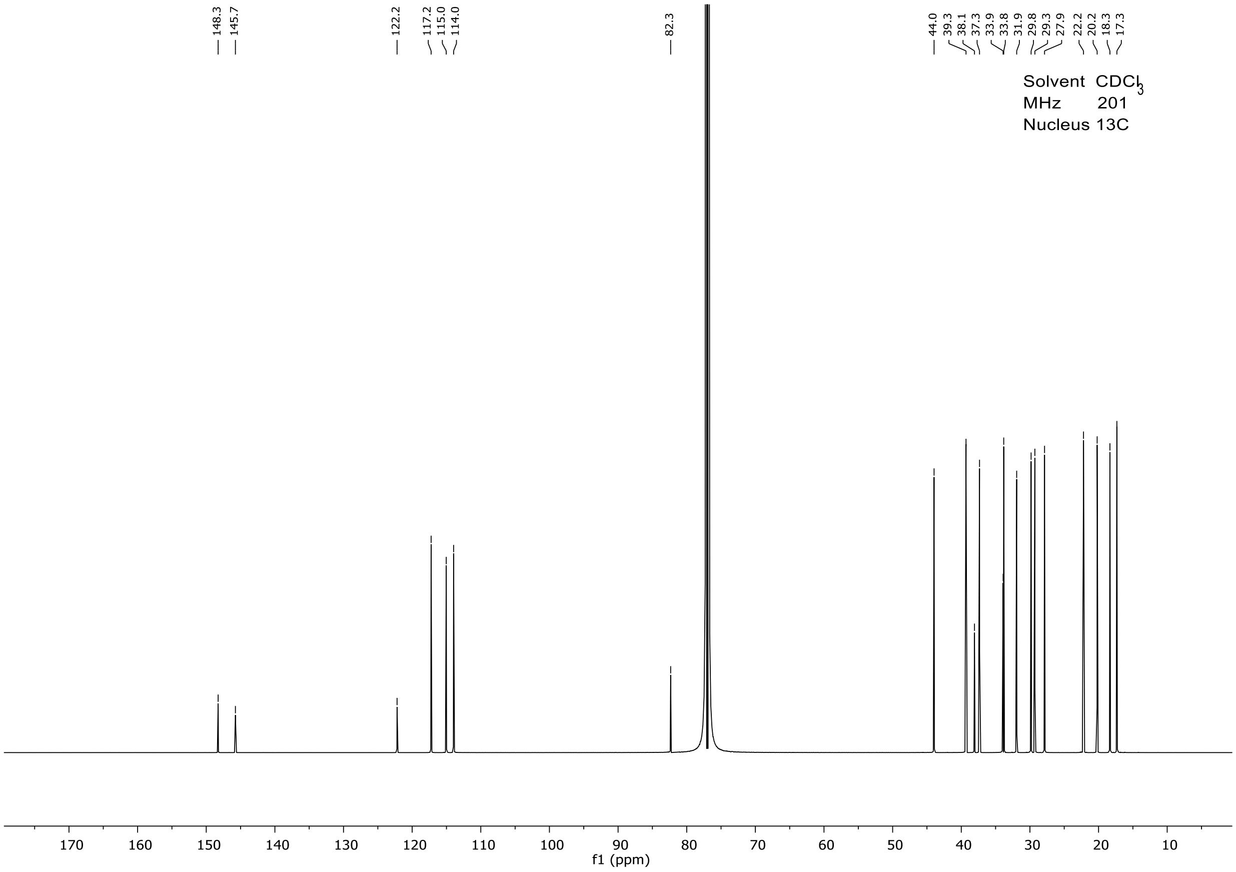

Supplementary Figure 25 ^{1}H and ^{13}C NMR Spectra for **2** in $CDCl_3$.

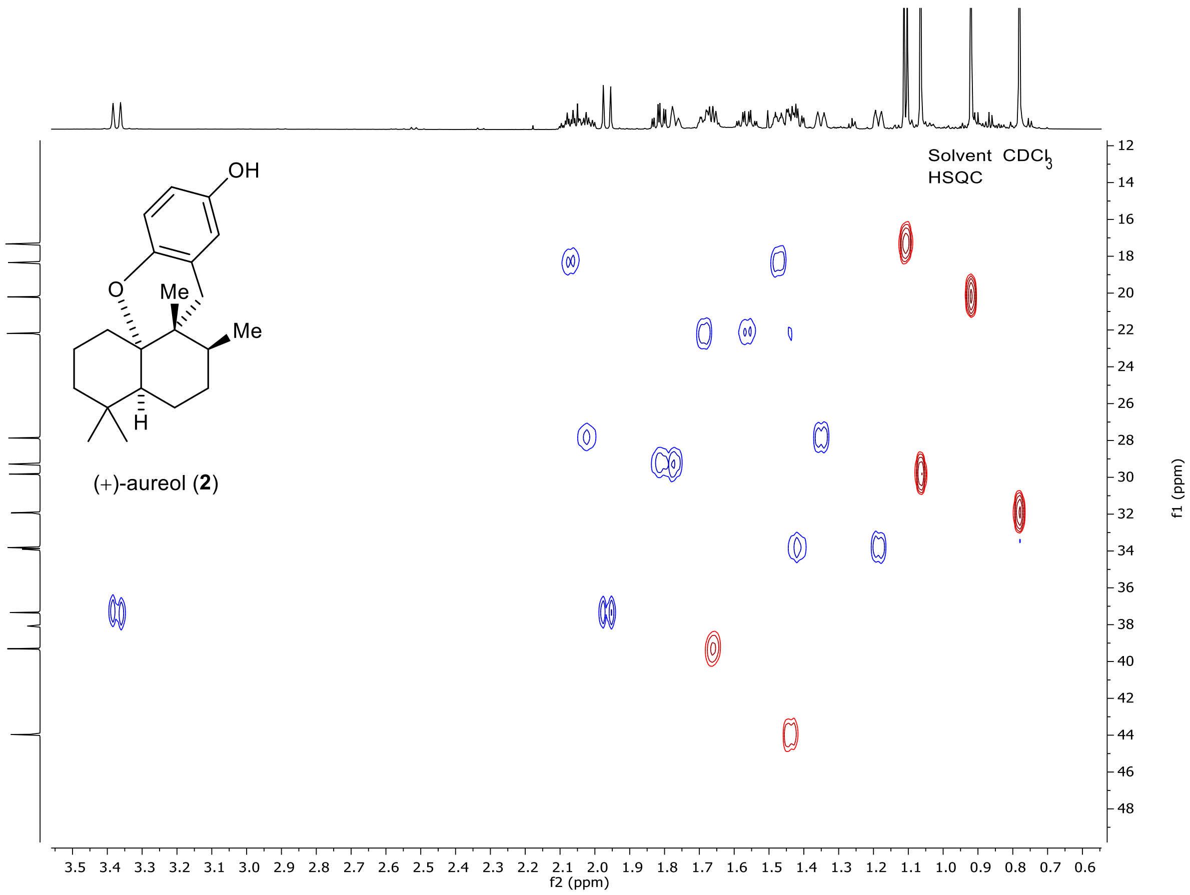

Supplementary Figure 26 HSQC NMR Spectra for **2** in $CDCl_3$.

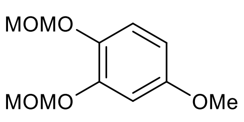

S28

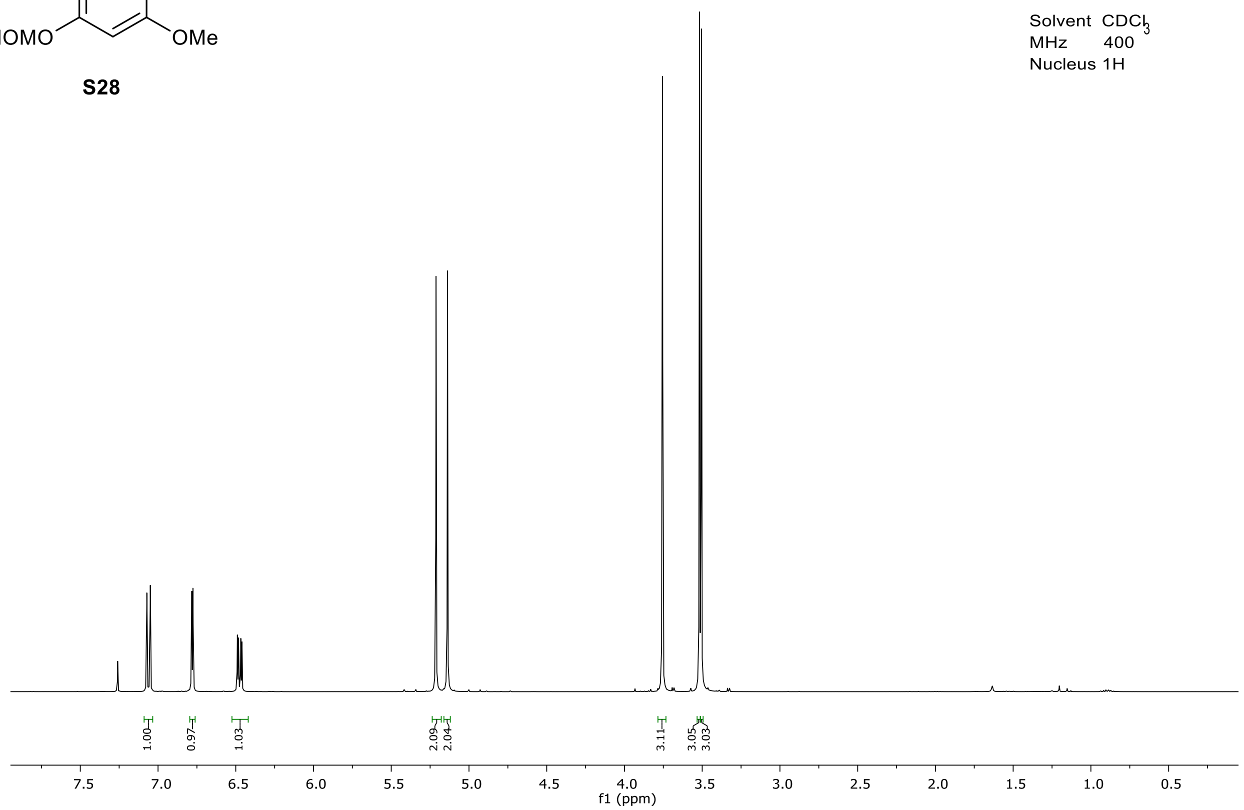

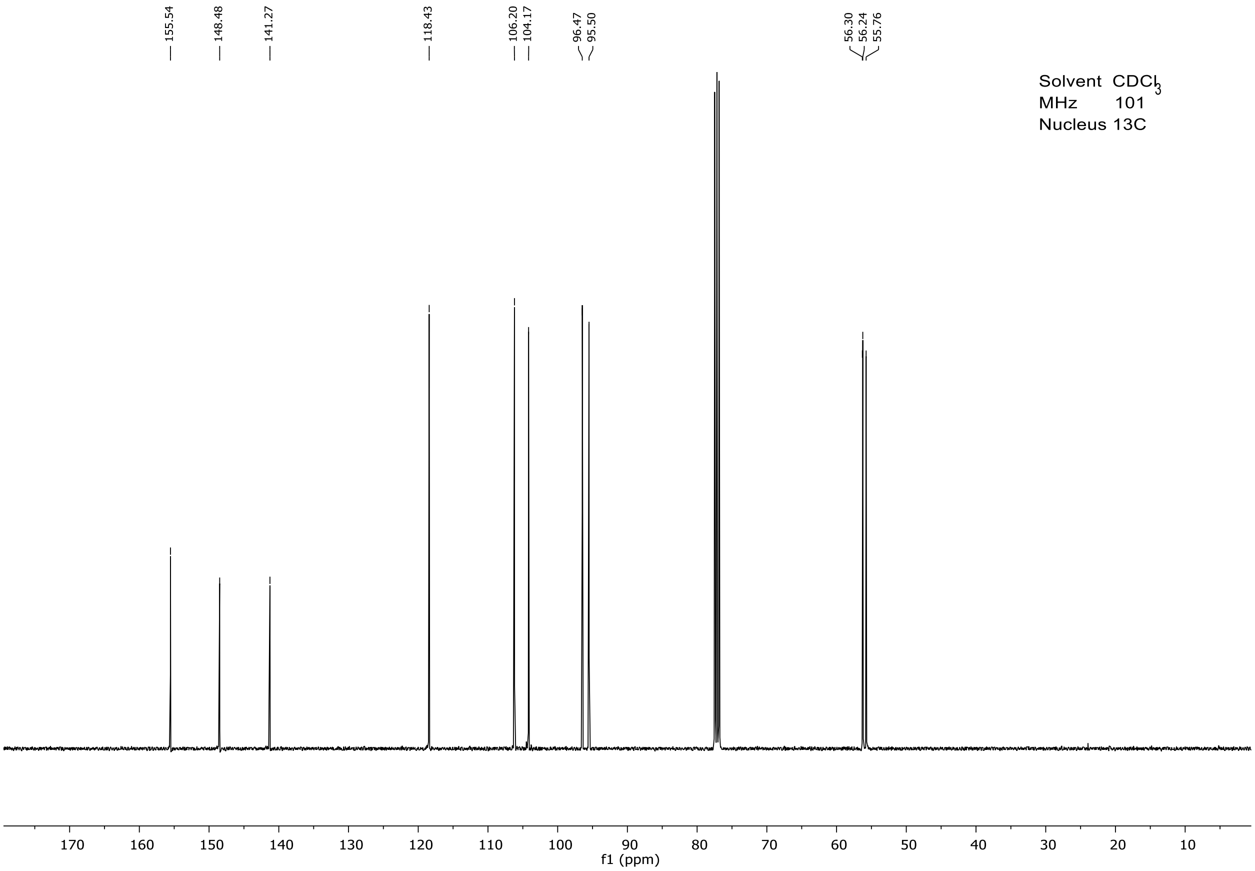

Supplementary Figure 27 ^{1}H and ^{13}C NMR Spectra for **S28** in $CDCl_3$.

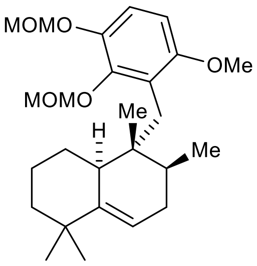

S30

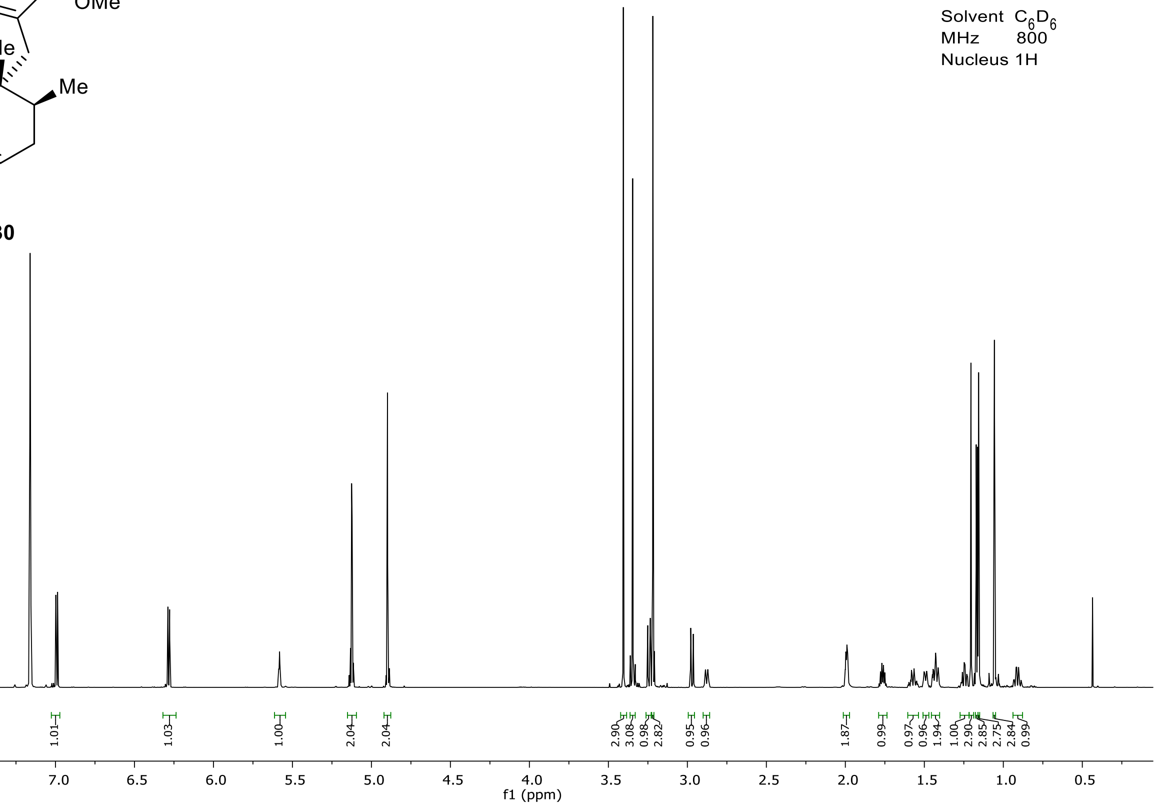

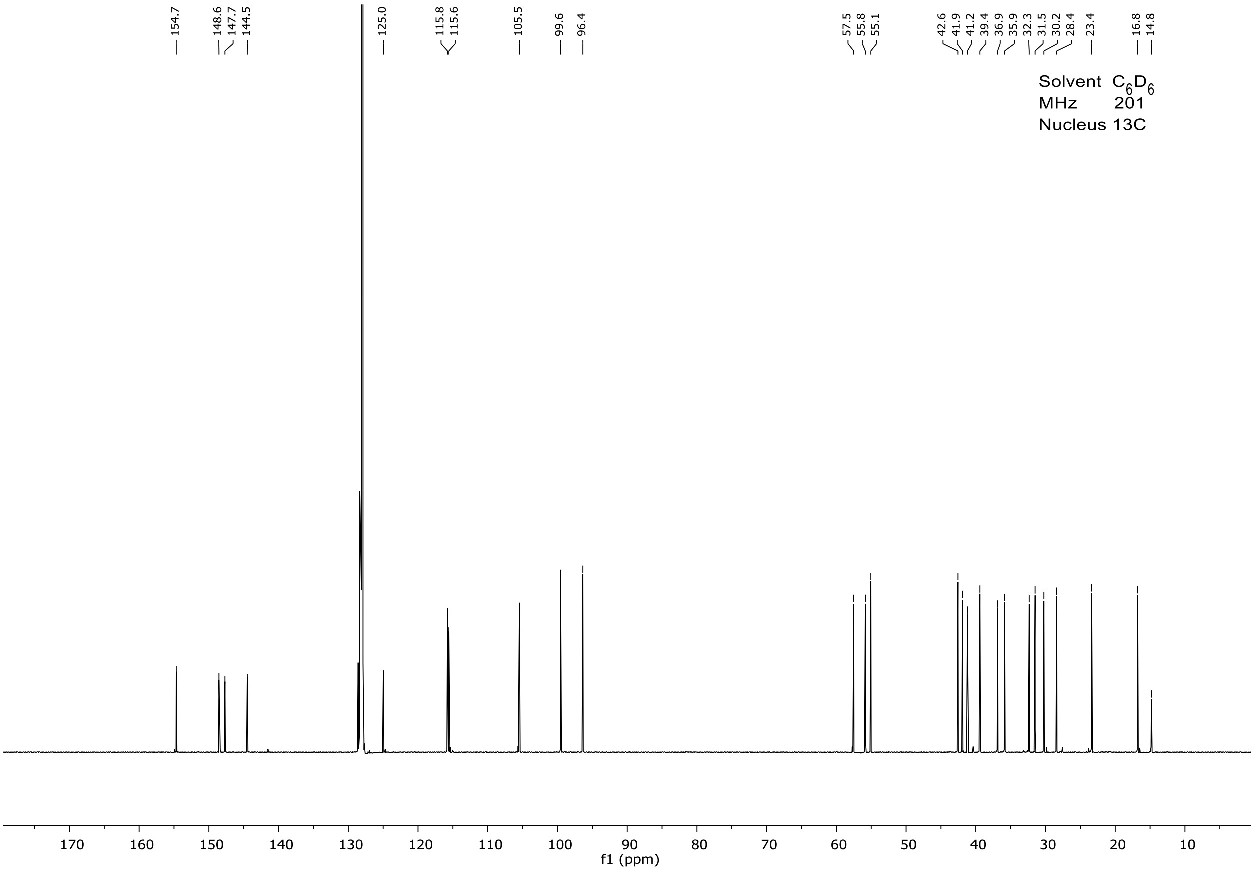

Supplementary Figure 28 ^{1}H and ^{13}C NMR Spectra for **S30** in C_6D_6.

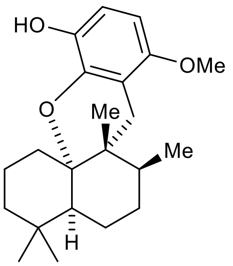

(+)-strongylin A (**4**)

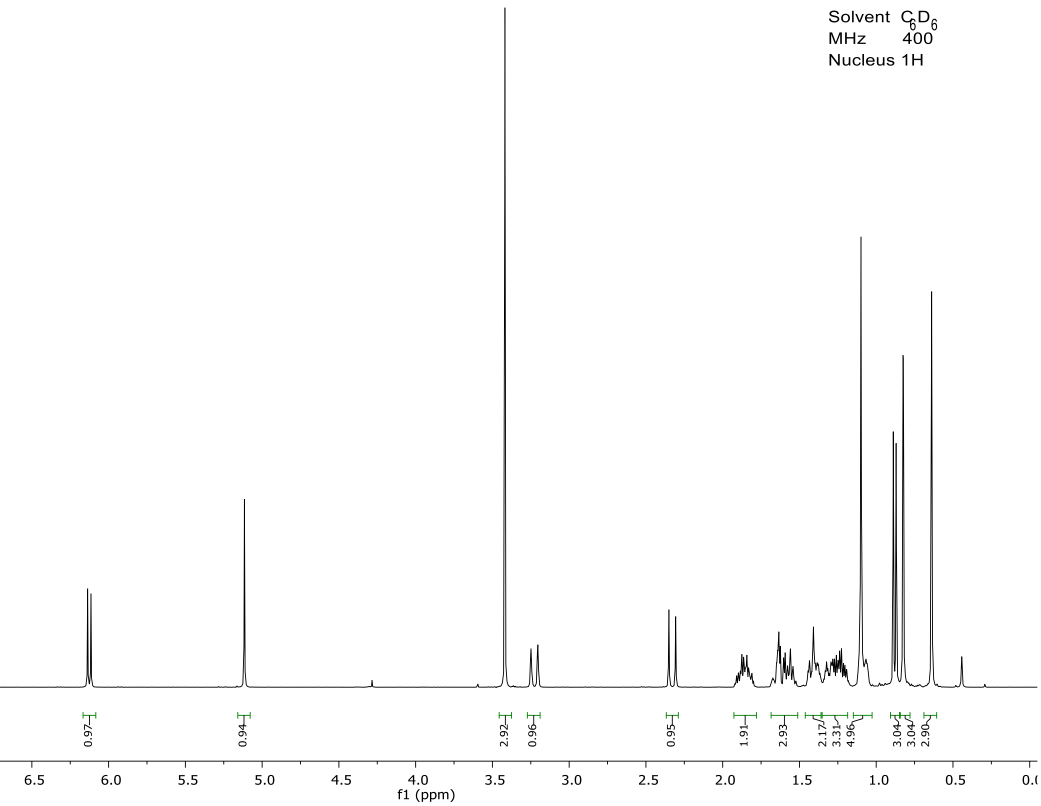

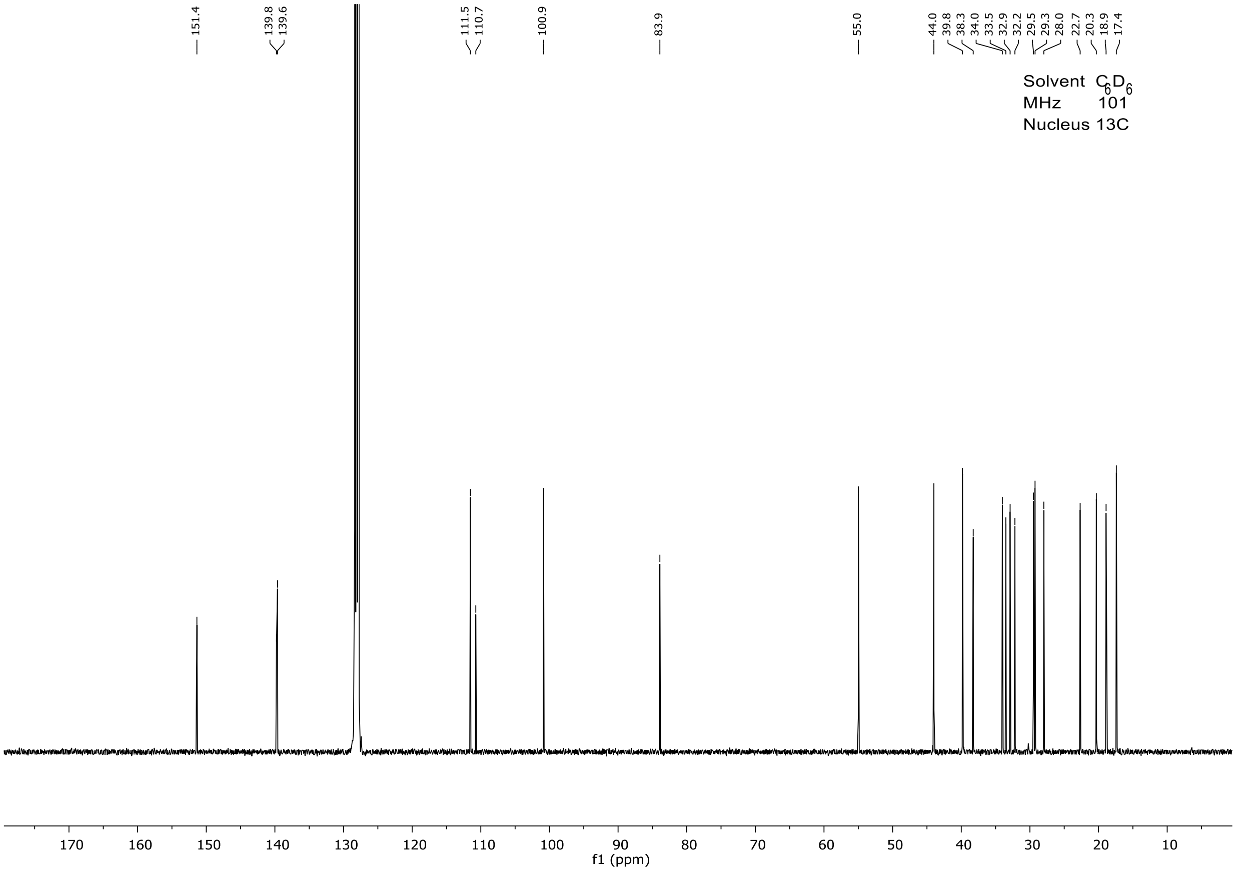

Supplementary Figure 29 ^{1}H and ^{13}C NMR Spectra for **4** in C_6D_6.

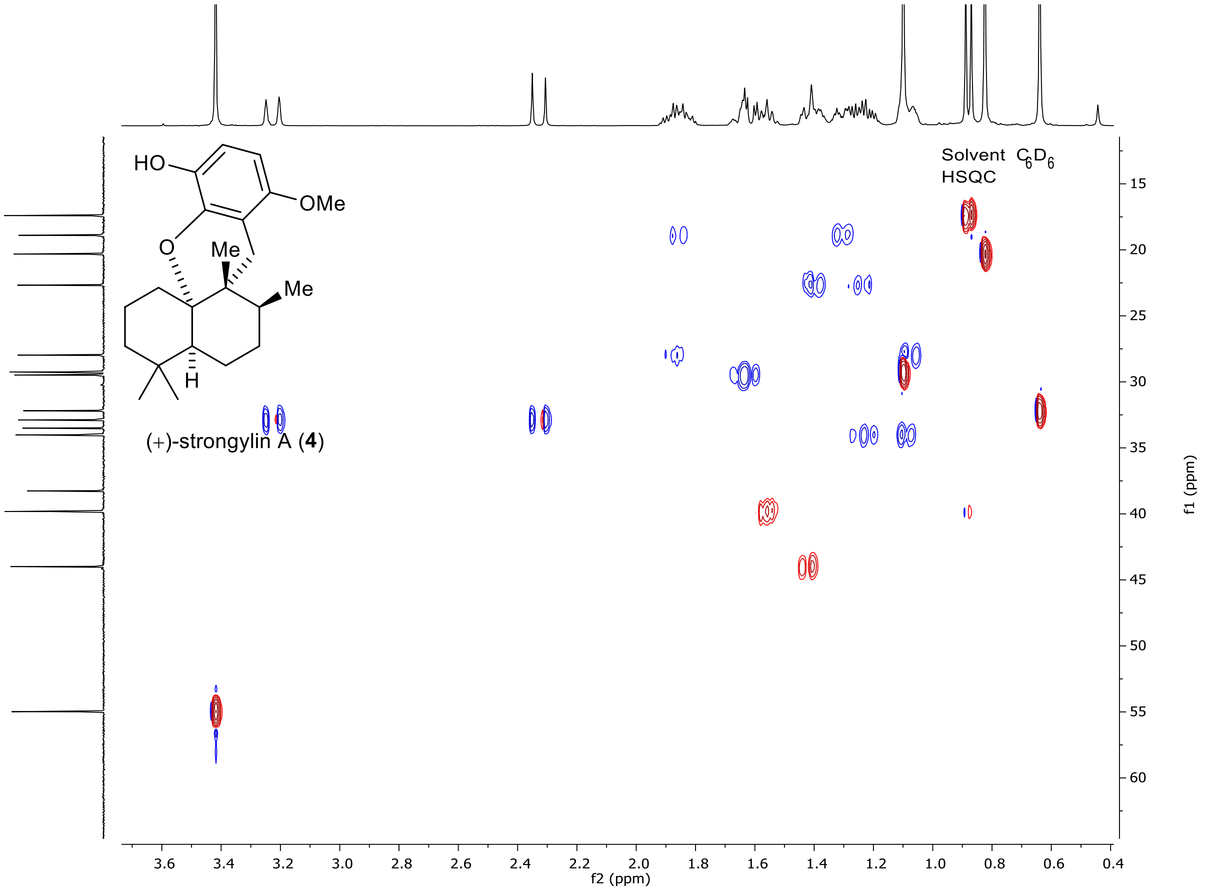

Supplementary Figure 30 HSQC NMR Spectra for **4** in C_6D_6.

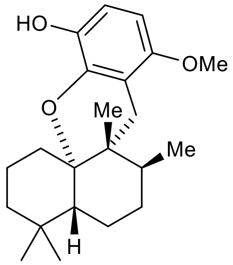

5-*epi*-strongylin A (**45**)

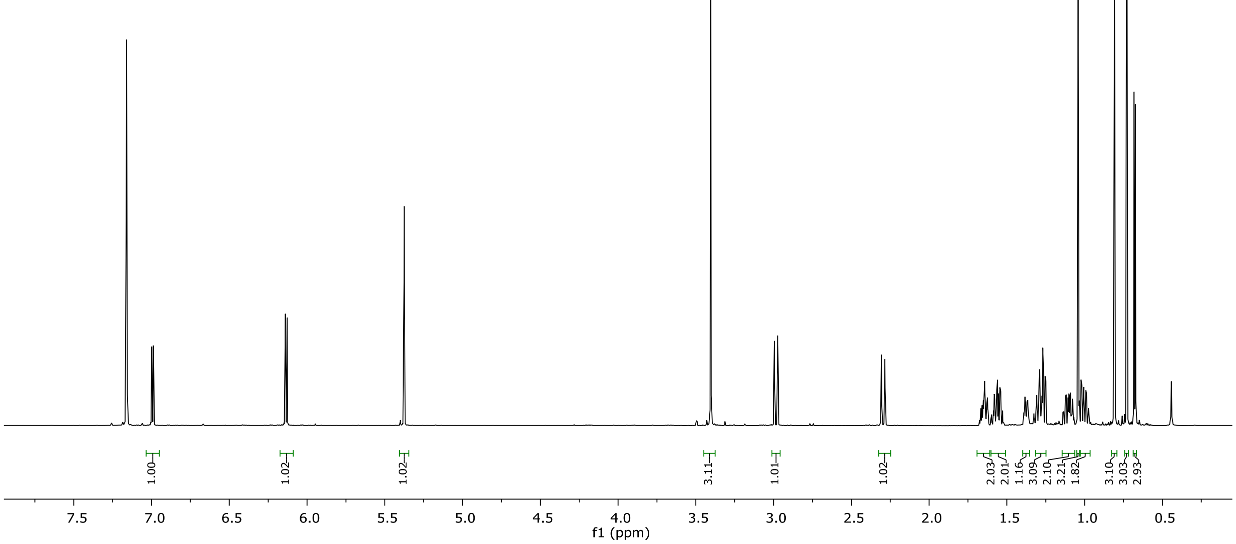

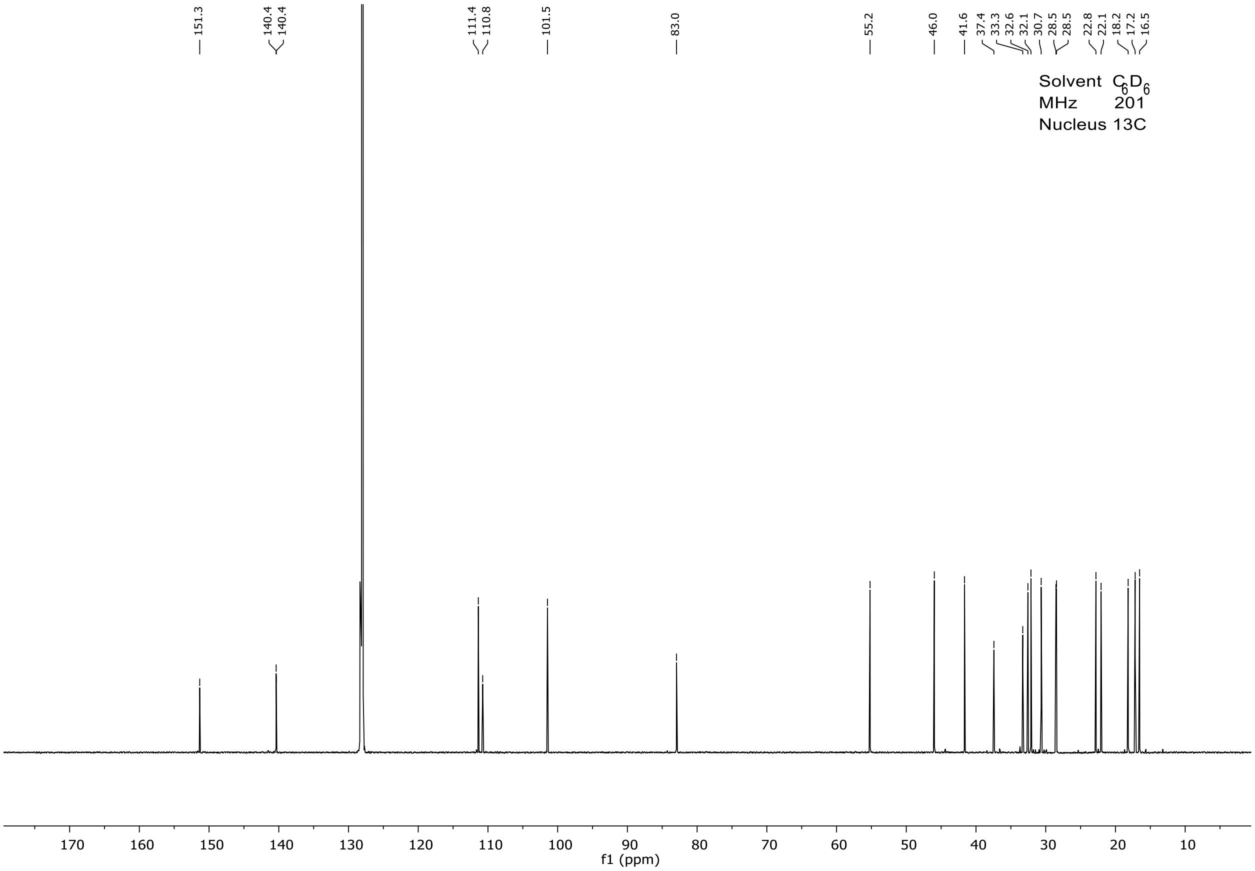

Supplementary Figure 31 ^{1}H and ^{13}C NMR Spectra for **45** in $CDCl_3$.

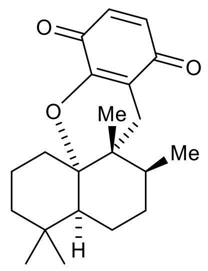

46

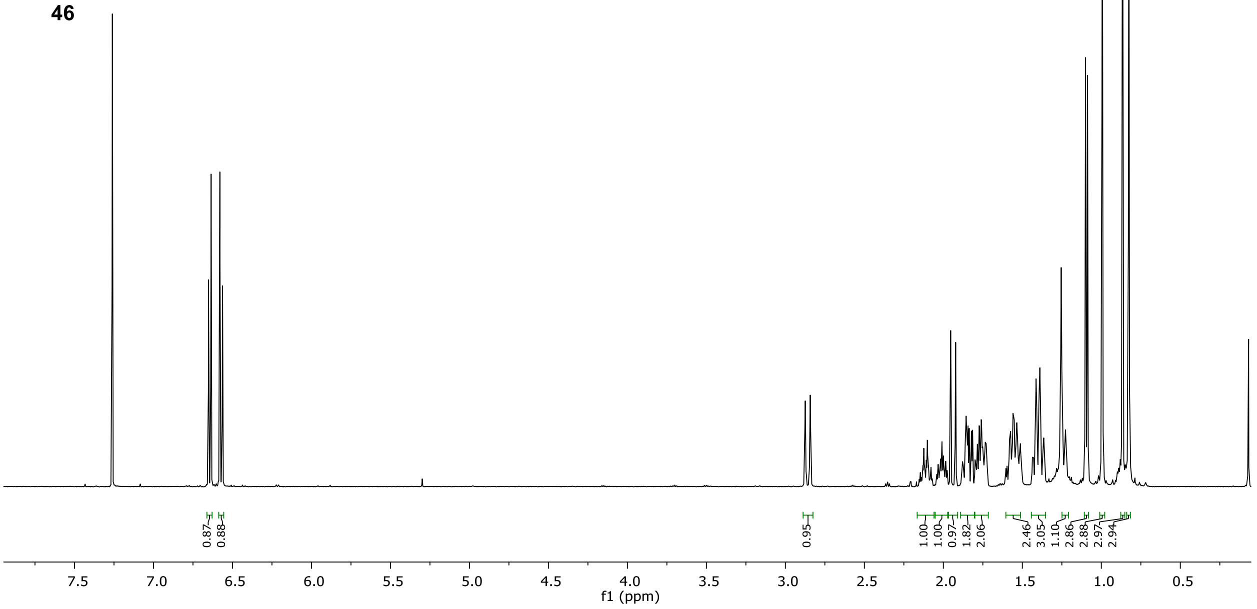

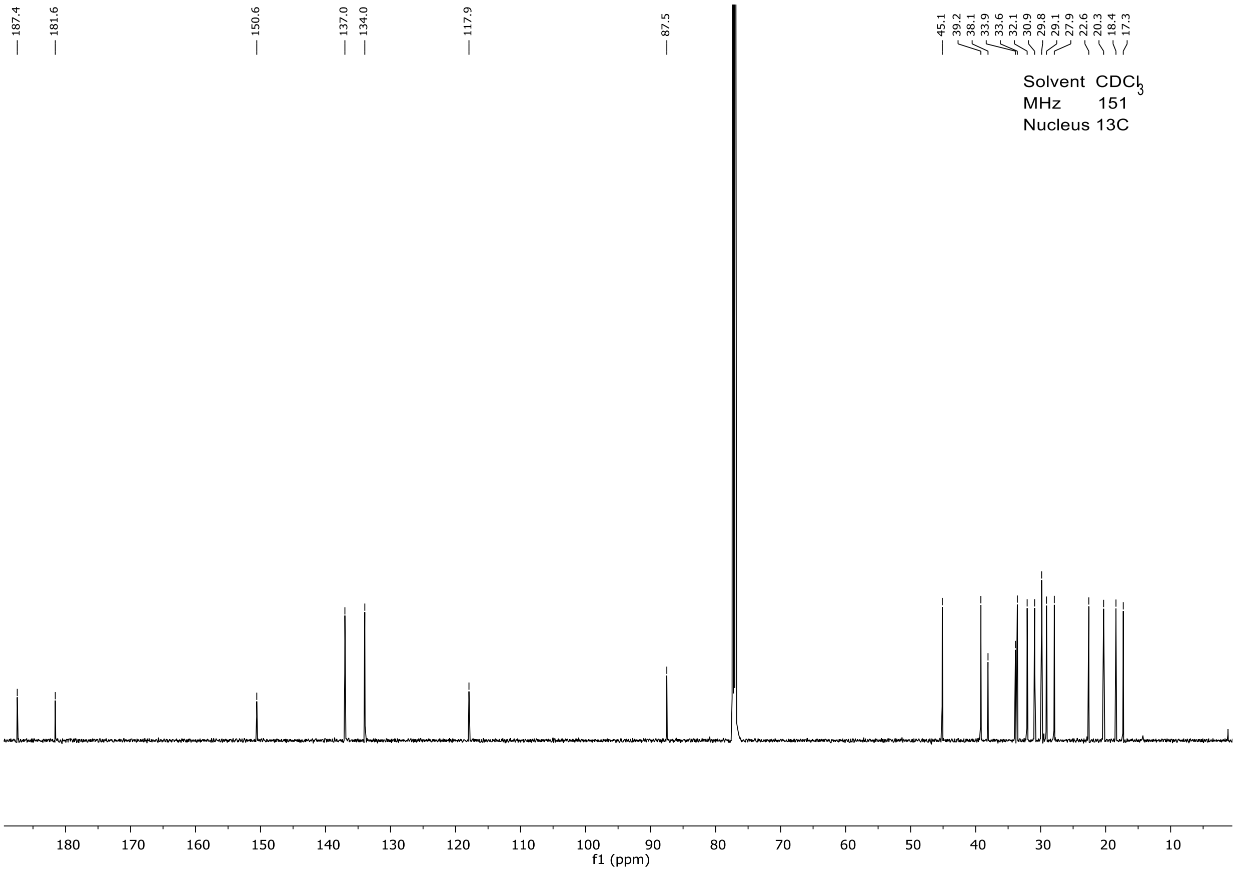

Supplementary Figure 32 ^{1}H and ^{13}C NMR Spectra for **46** in $CDCl_3$.

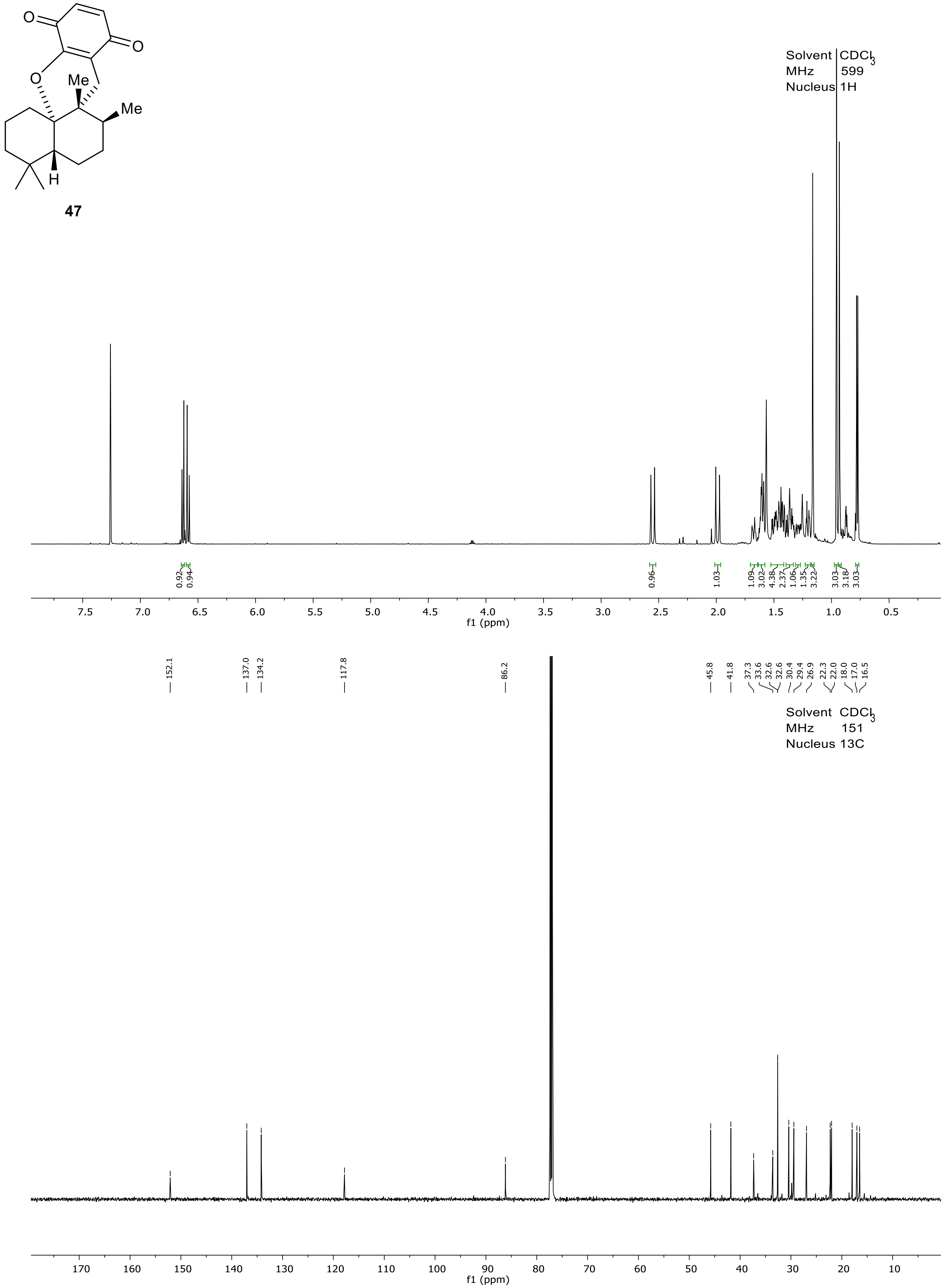

Supplementary Figure 33 ^{1}H and ^{13}C NMR Spectra for **47** in $CDCl_3$.

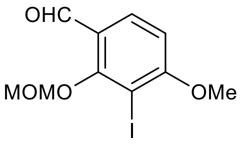

S34

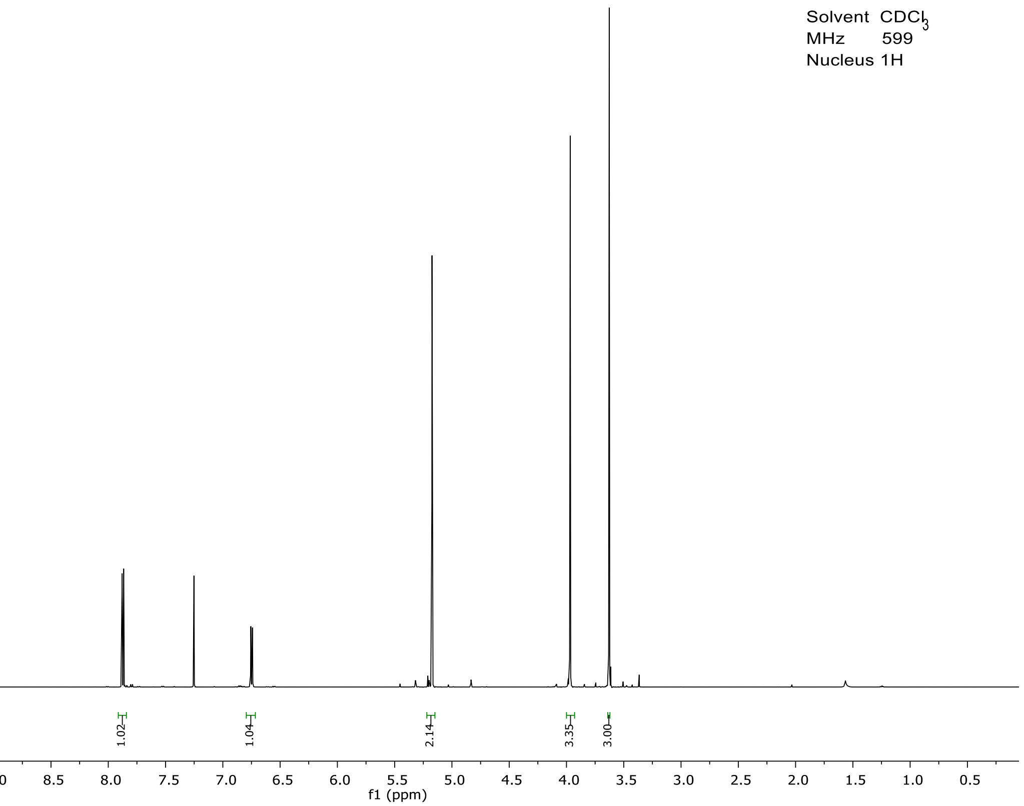

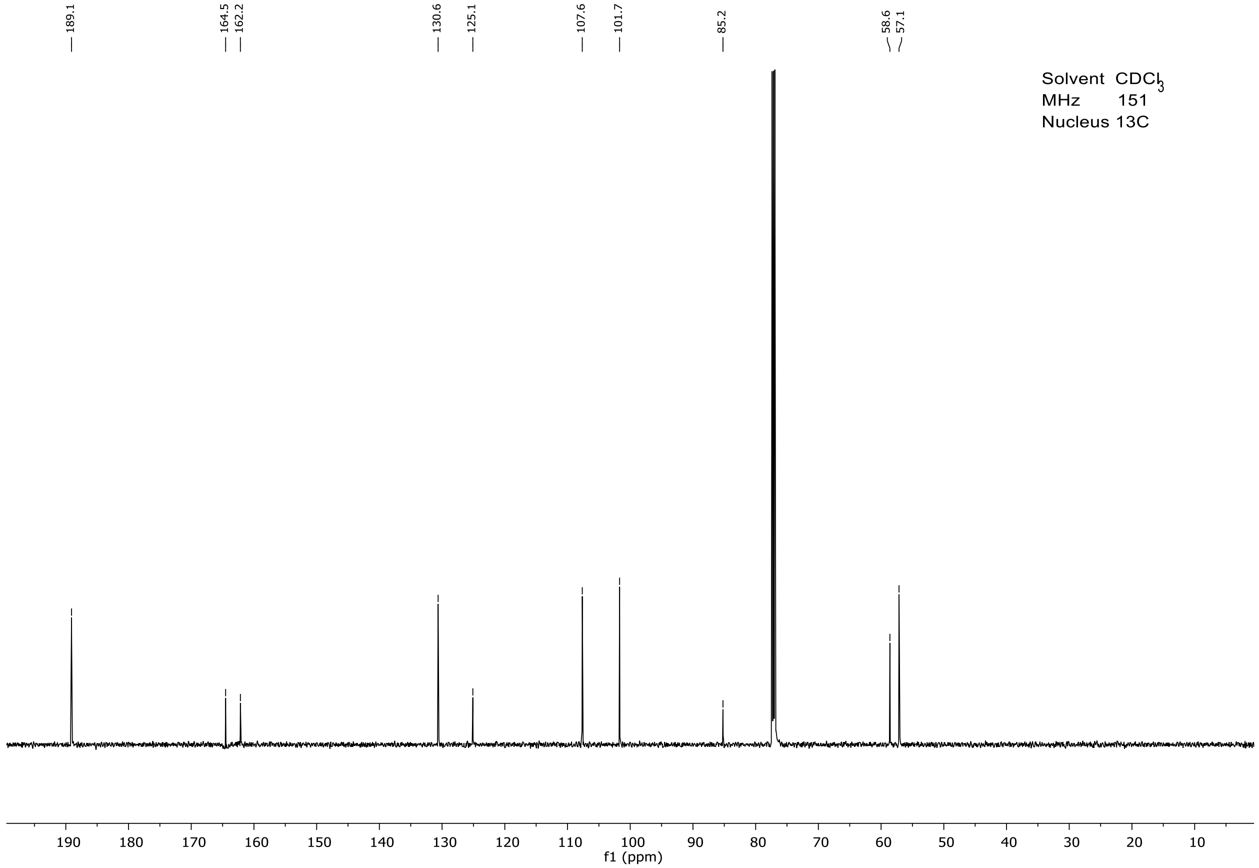

Supplementary Figure 34 ^{1}H and ^{13}C NMR Spectra for **S34** in $CDCl_3$.

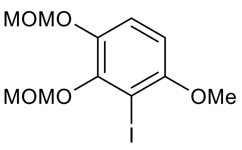

S36

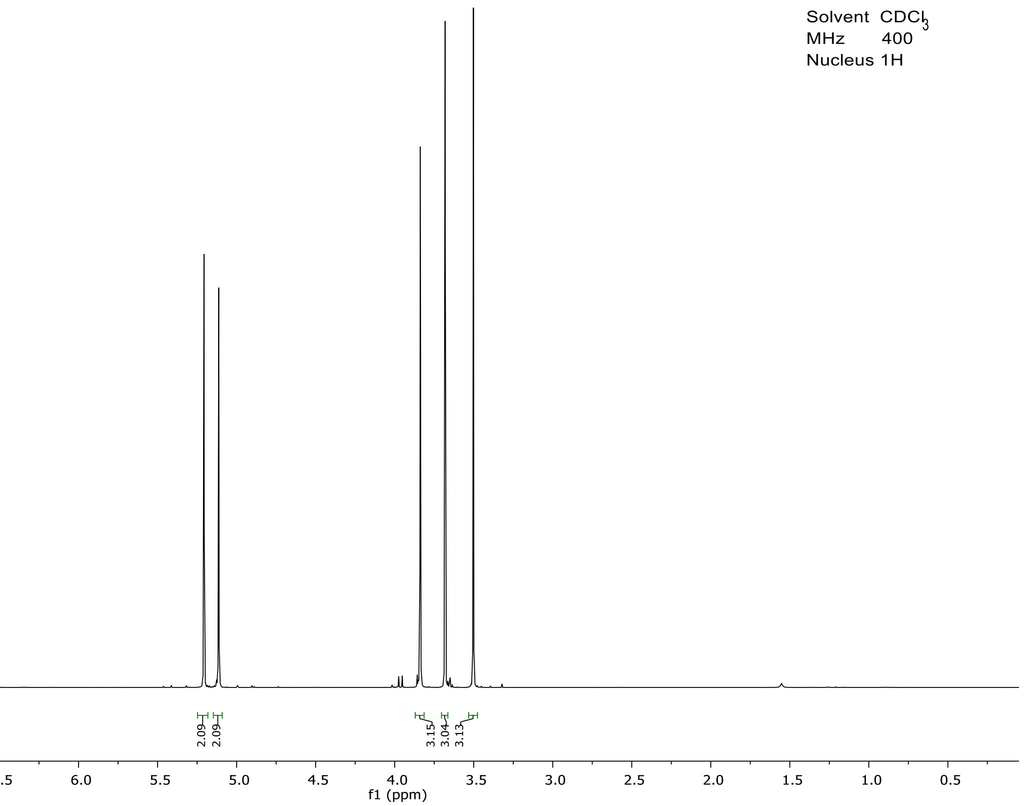

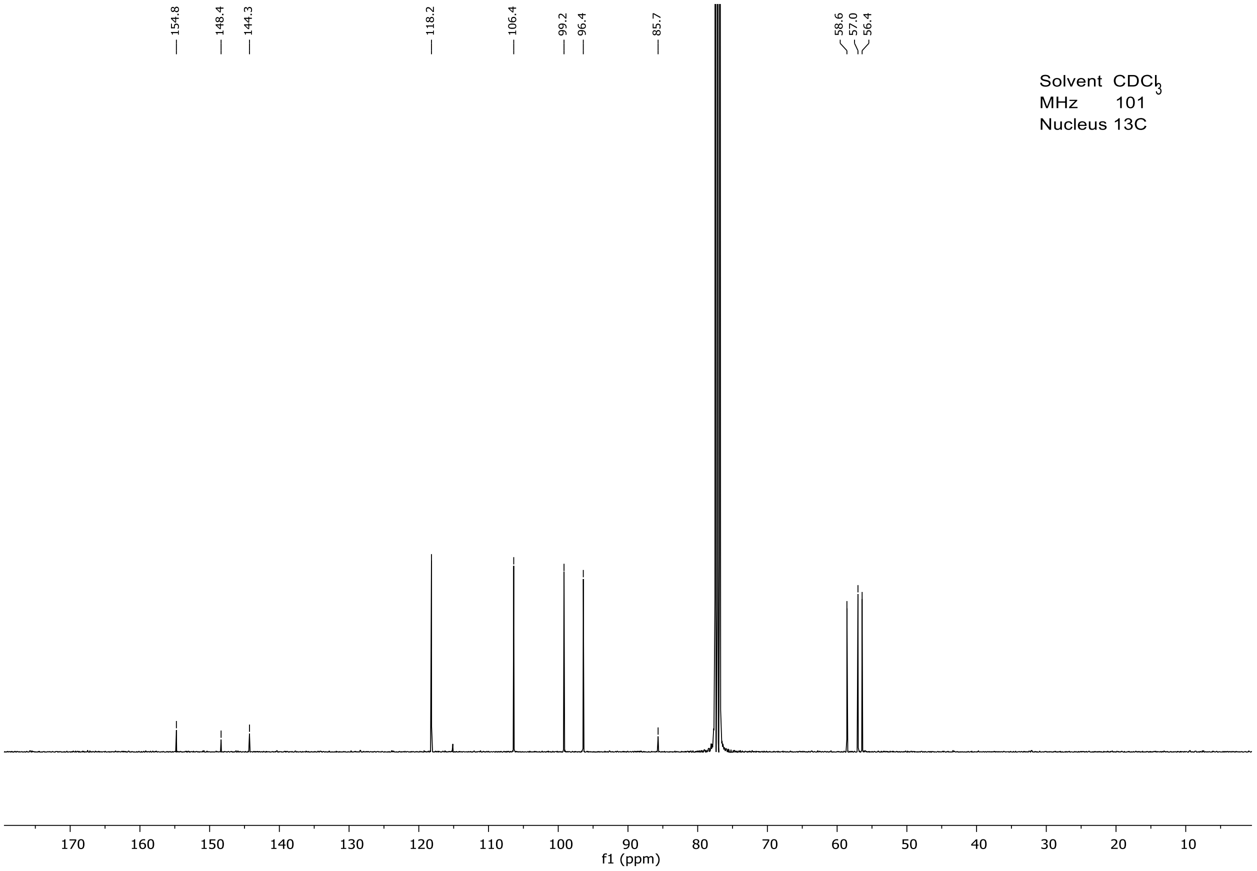

Supplementary Figure 35 ^{1}H and ^{13}C NMR Spectra for **S36** in $CDCl_3$.

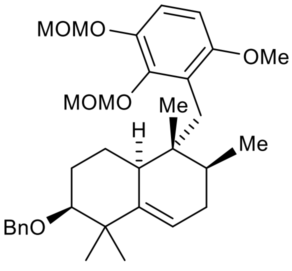

S37

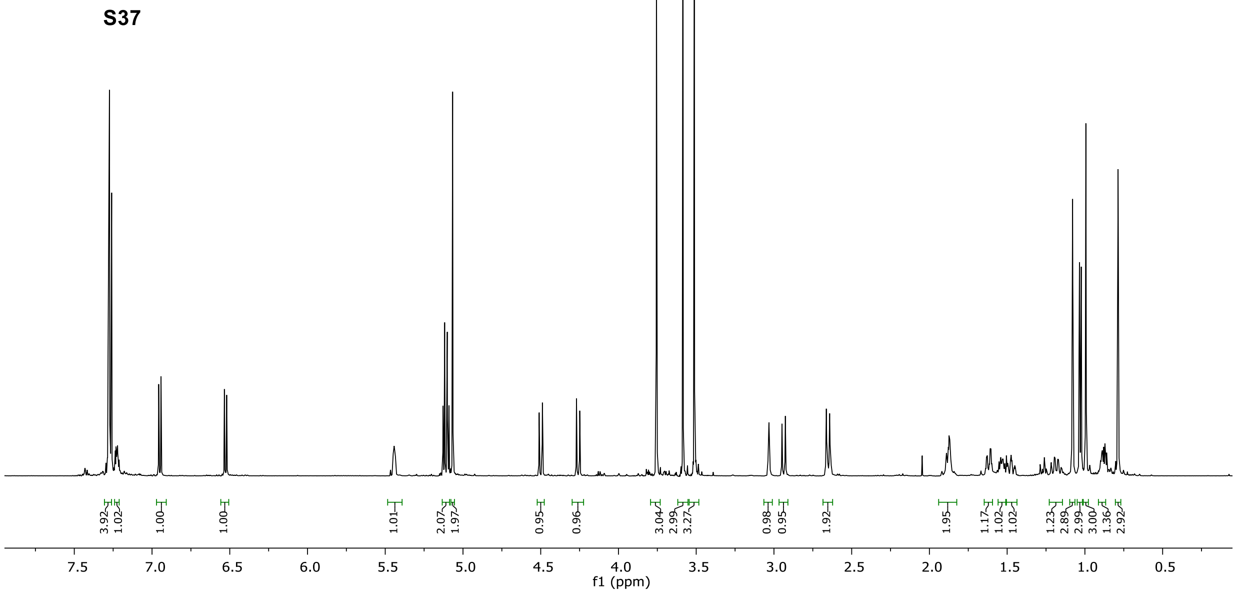

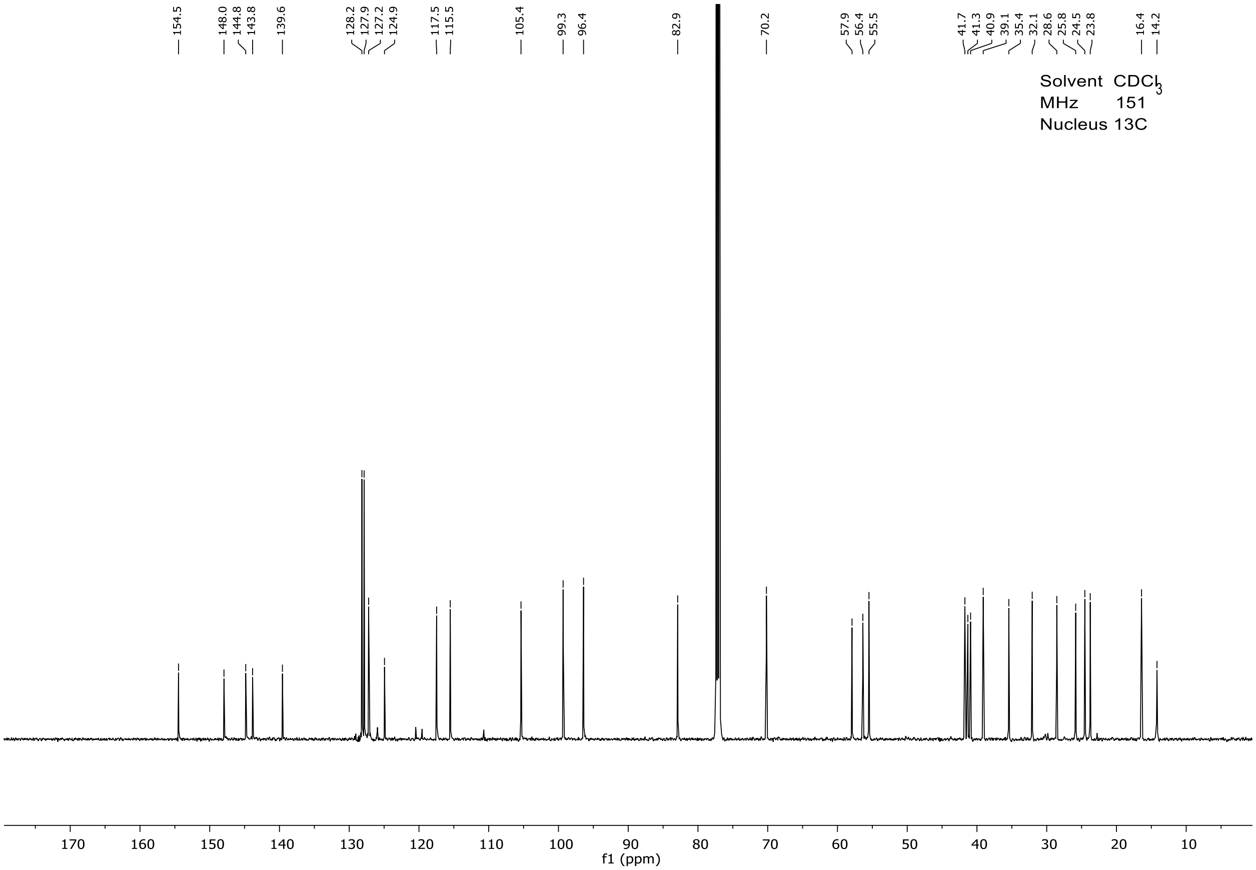

Supplementary Figure 36 ^{1}H and ^{13}C NMR Spectra for **S37** in $CDCl_3$.

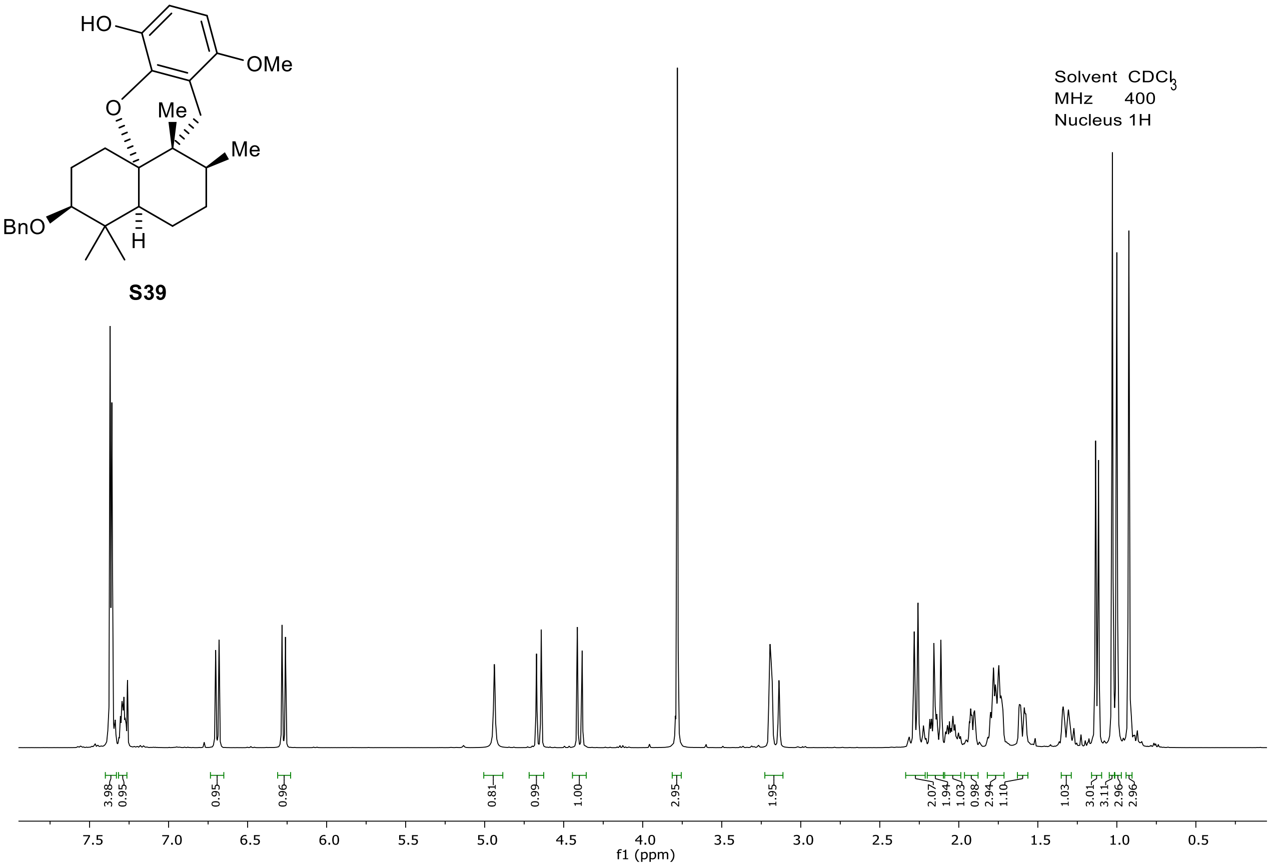

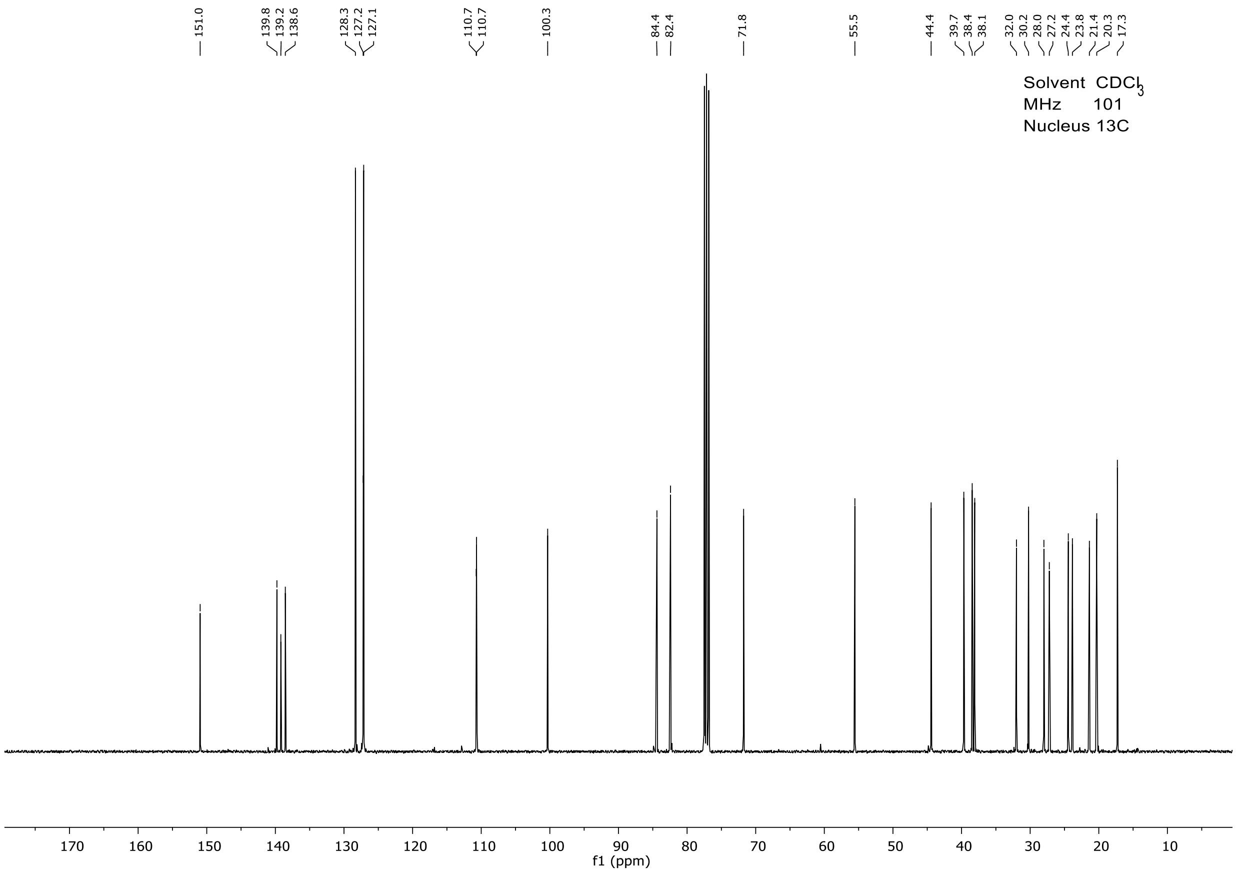

Supplementary Figure 37 ^{1}H and ^{13}C NMR Spectra for **S39** in $CDCl_3$.

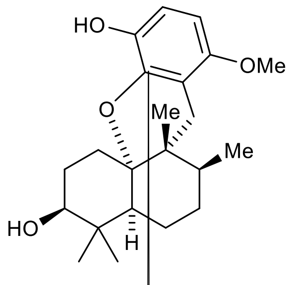

48

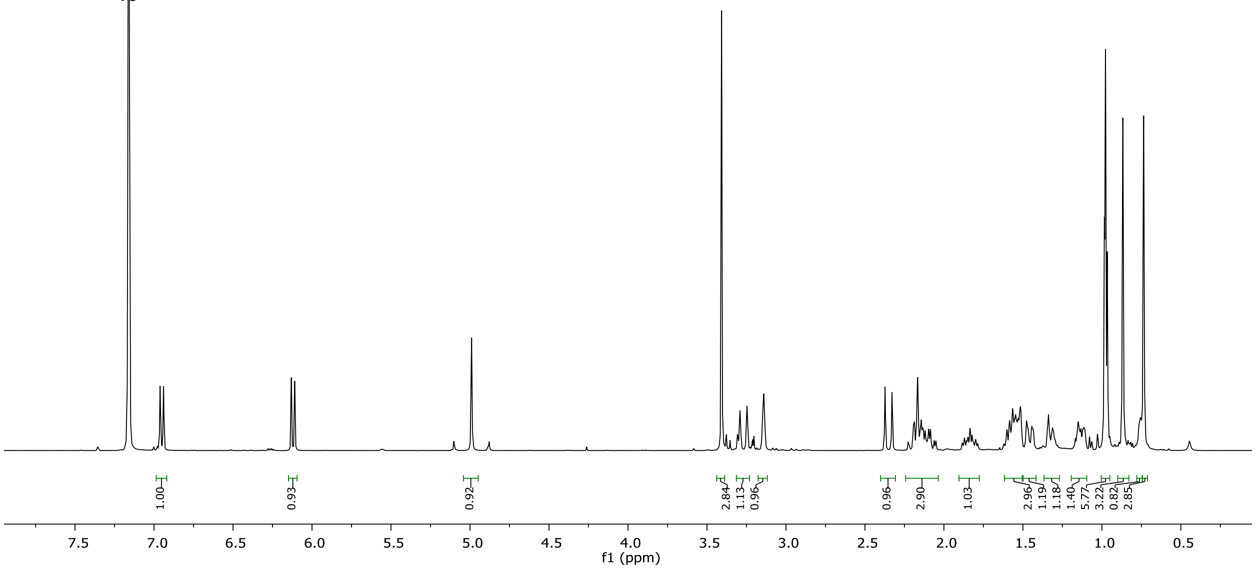

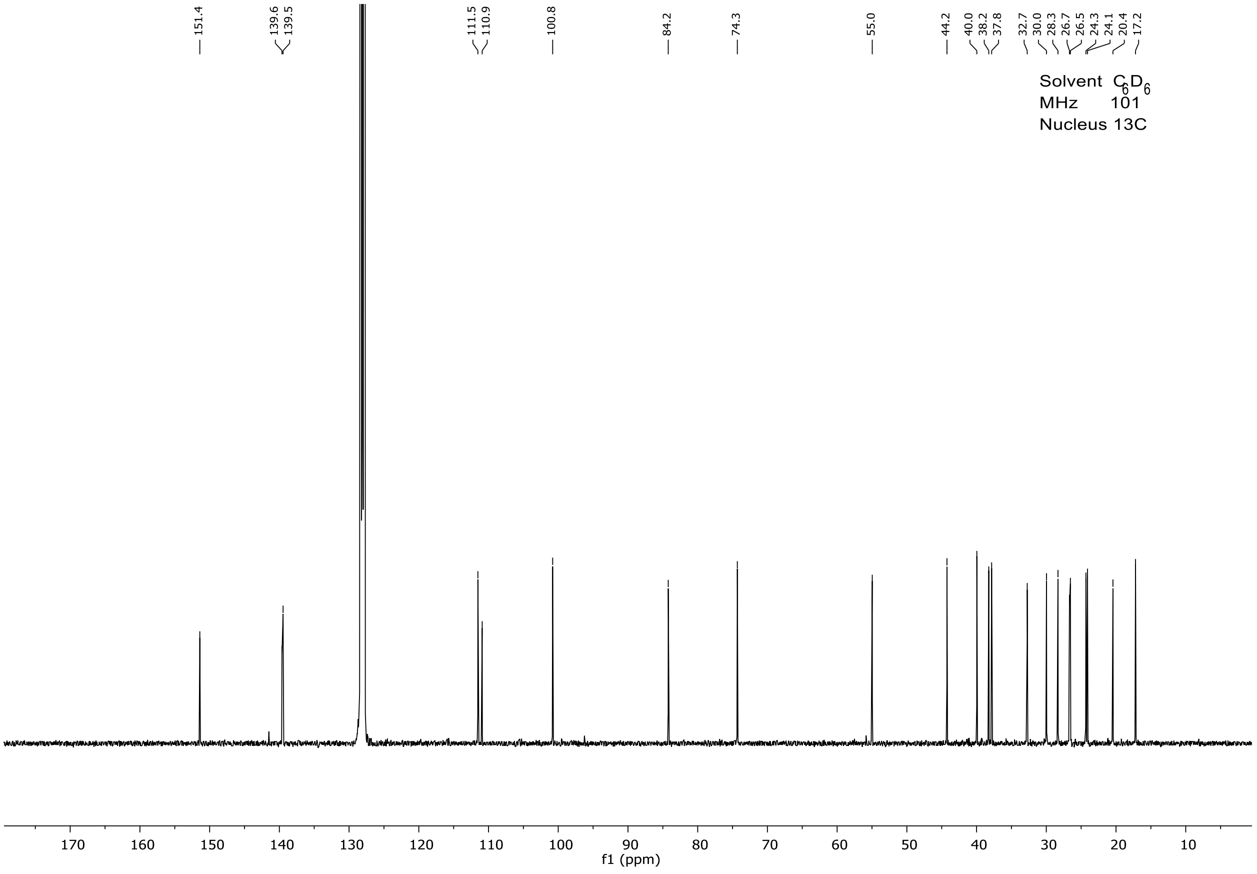

Supplementary Figure 38 ^{1}H and ^{13}C NMR Spectra for **48** in C_6D_6.

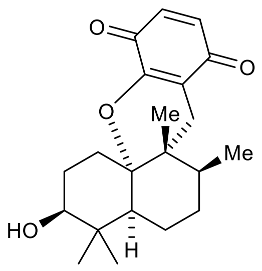

49

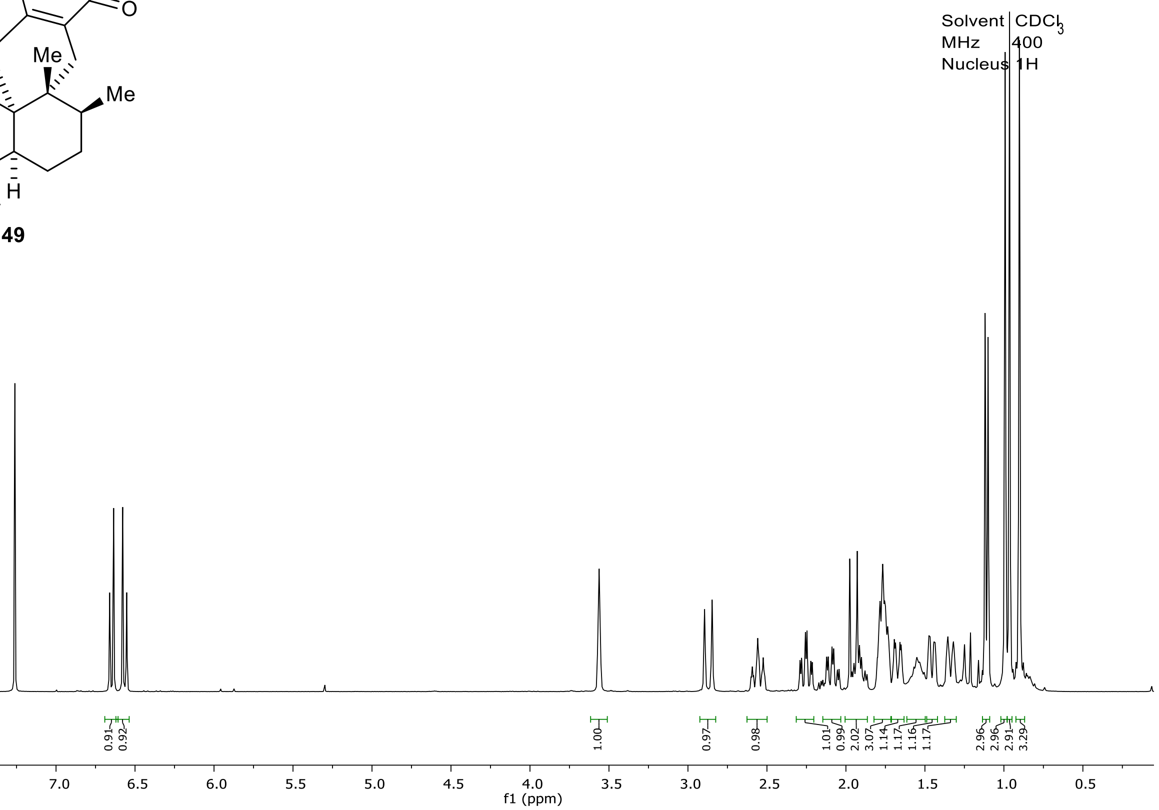

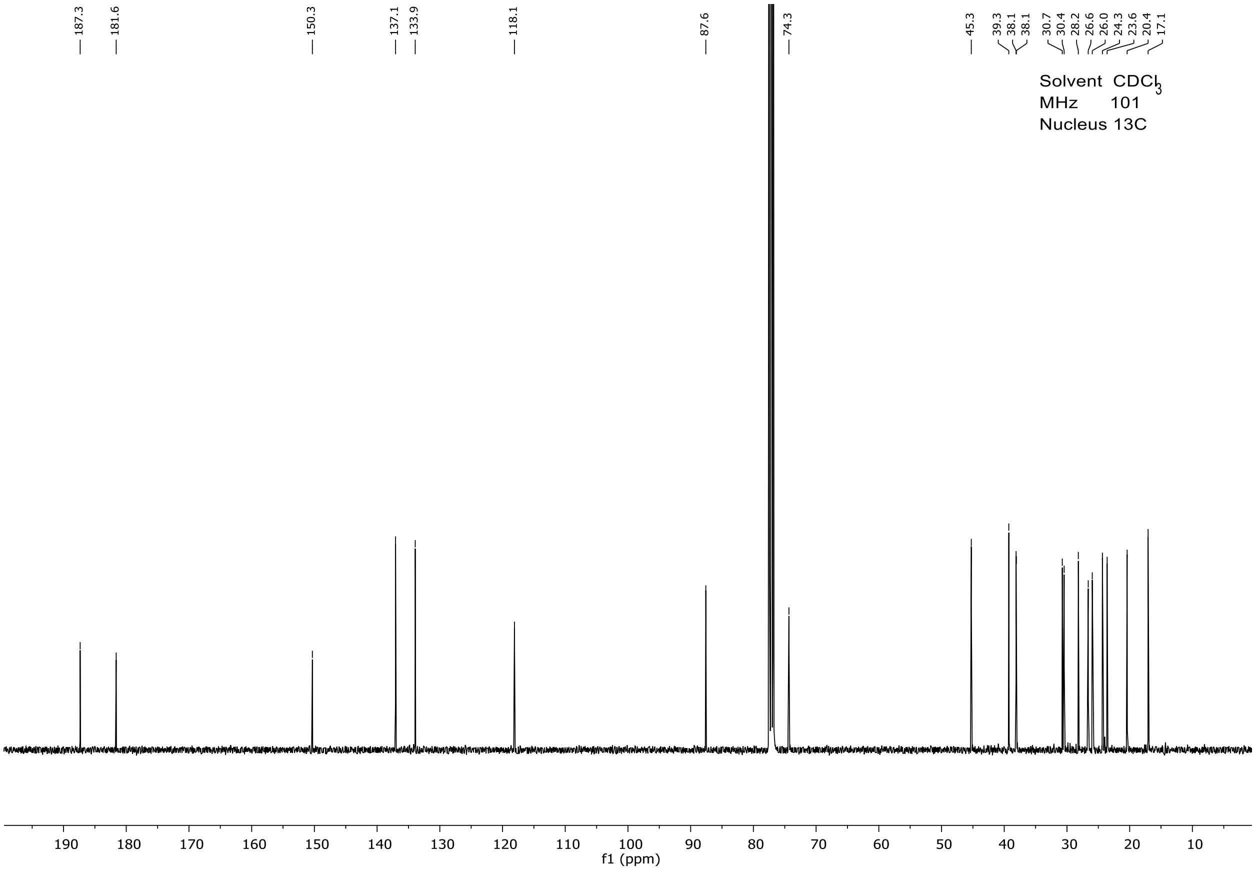

Supplementary Figure 39 ^{1}H and ^{13}C NMR Spectra for **49** in $CDCl_3$.

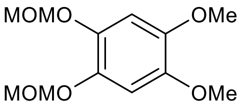

S43

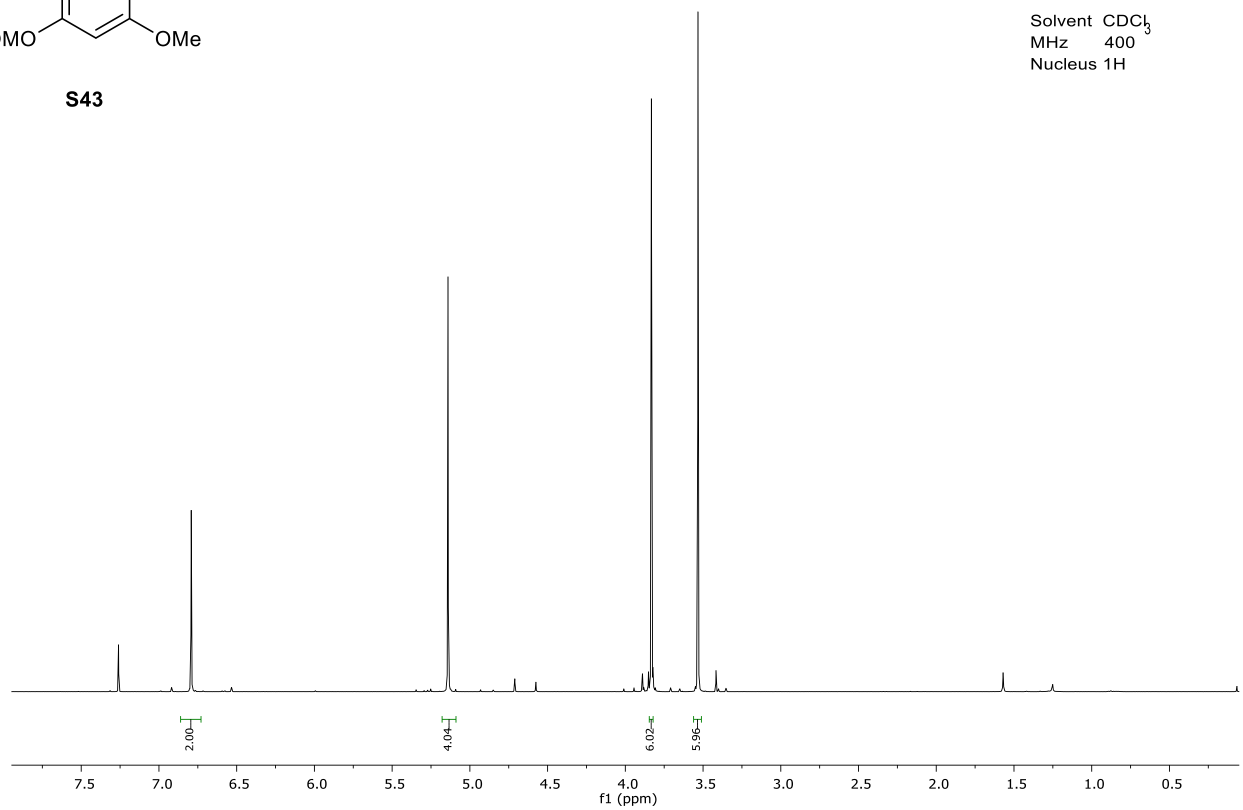

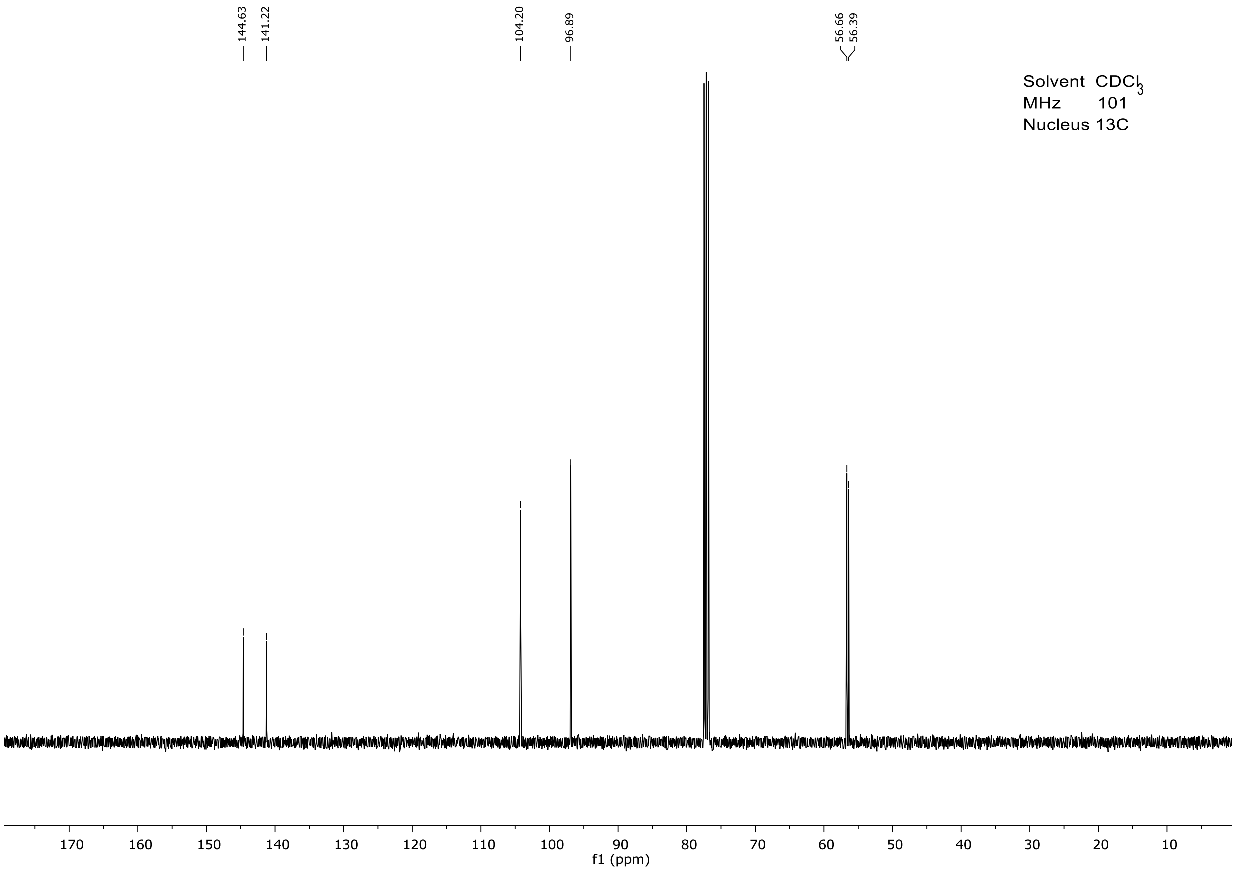

Supplementary Figure 40 ^{1}H and ^{13}C NMR Spectra for **S43** in $CDCl_3$.

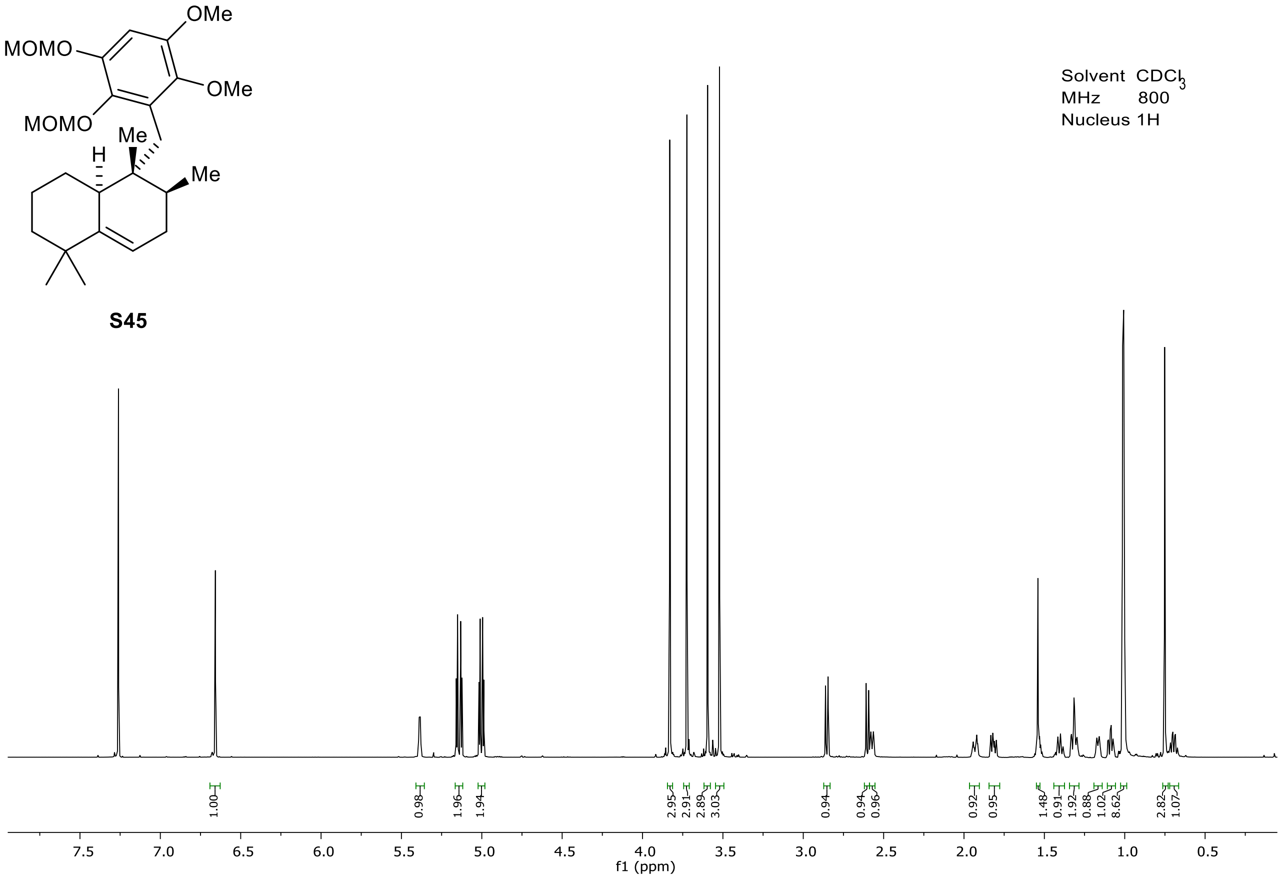

S45

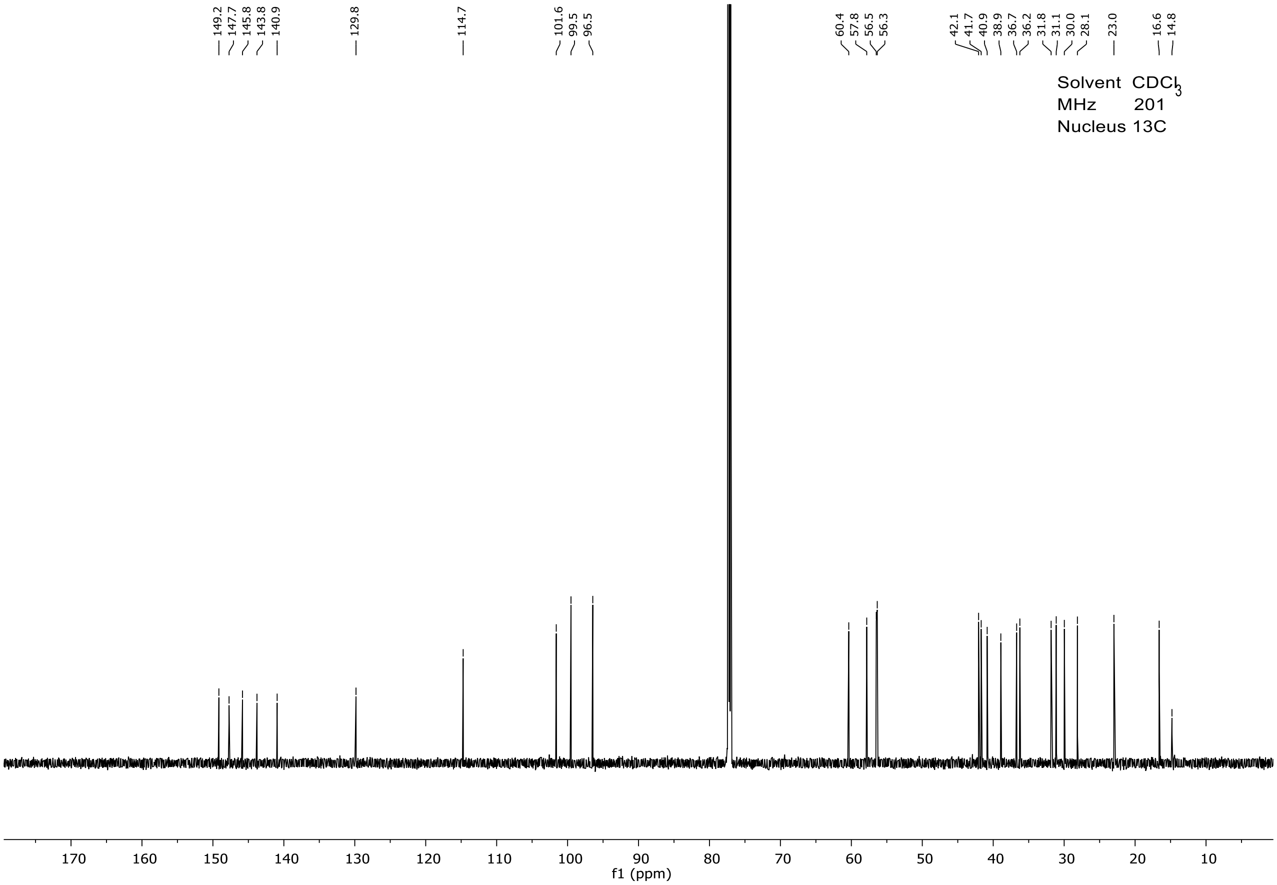

Supplementary Figure 41 ^{1}H and ^{13}C NMR Spectra for **S45** in $CDCl_3$.

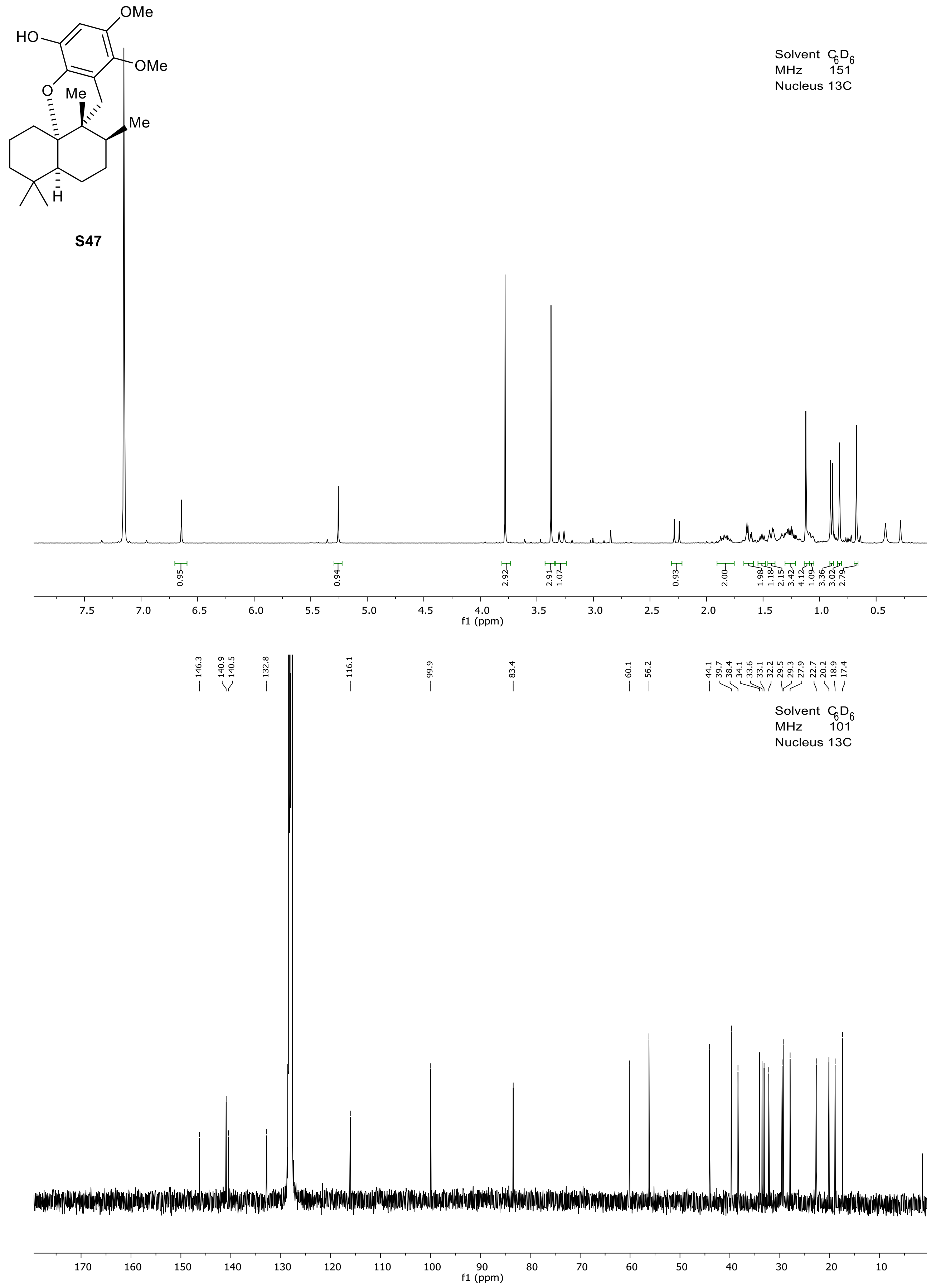

Supplementary Figure 42 ^{1}H and ^{13}C NMR Spectra for **S47** in C_6D_6.

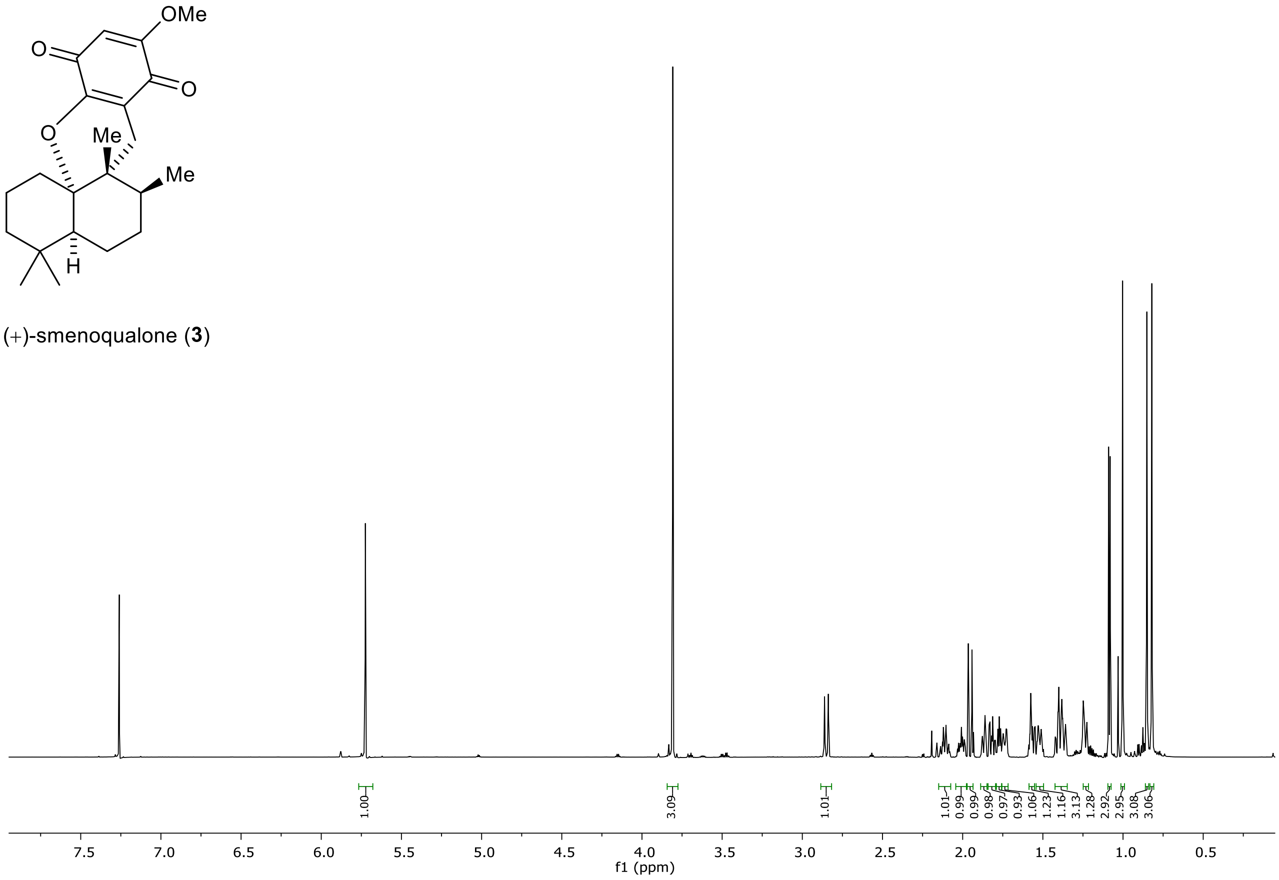

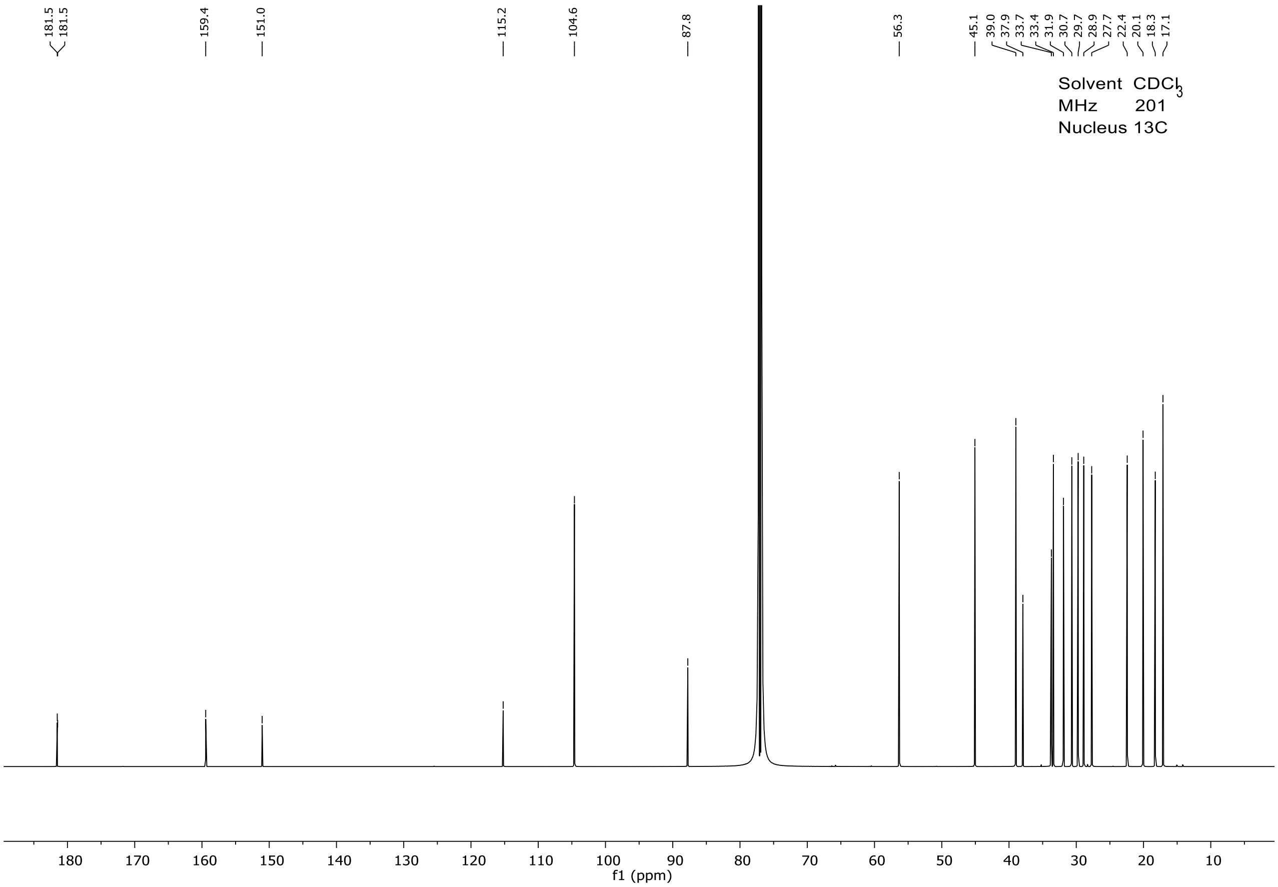

Supplementary Figure 43 ^{1}H and ^{13}C NMR Spectra for **3** in $CDCl_3$.

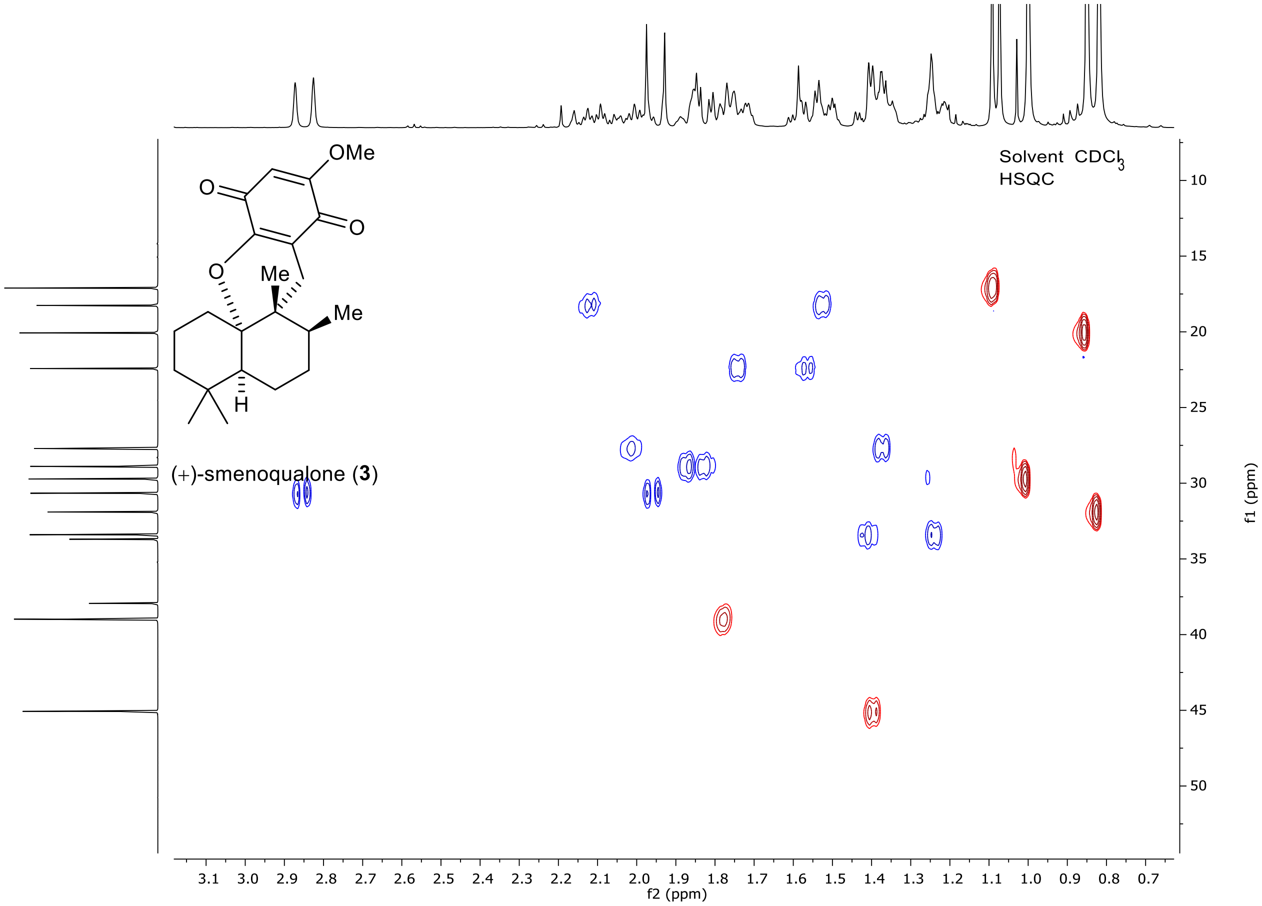

Supplementary Figure 44 HSQC C NMR Spectra for **3** in $CDCl_3$.

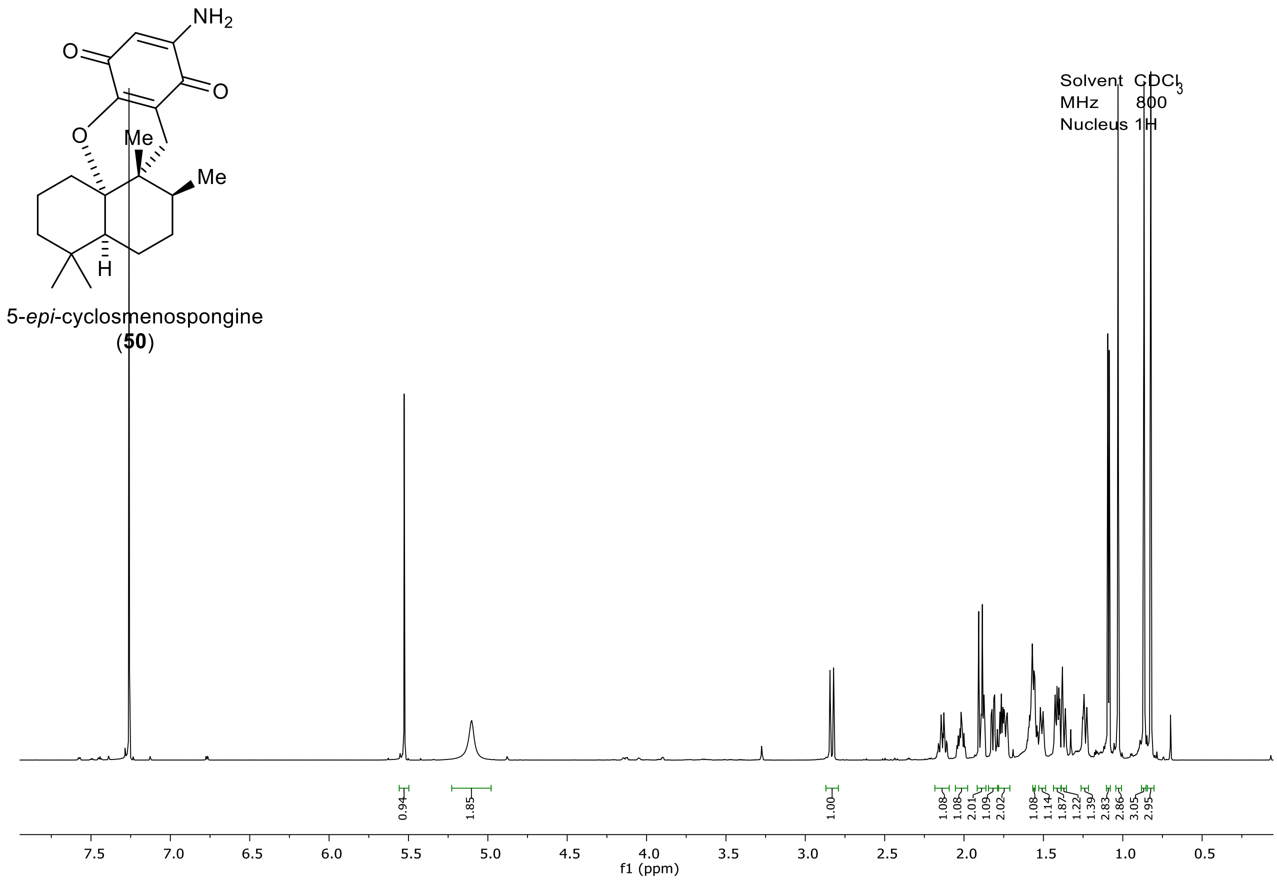

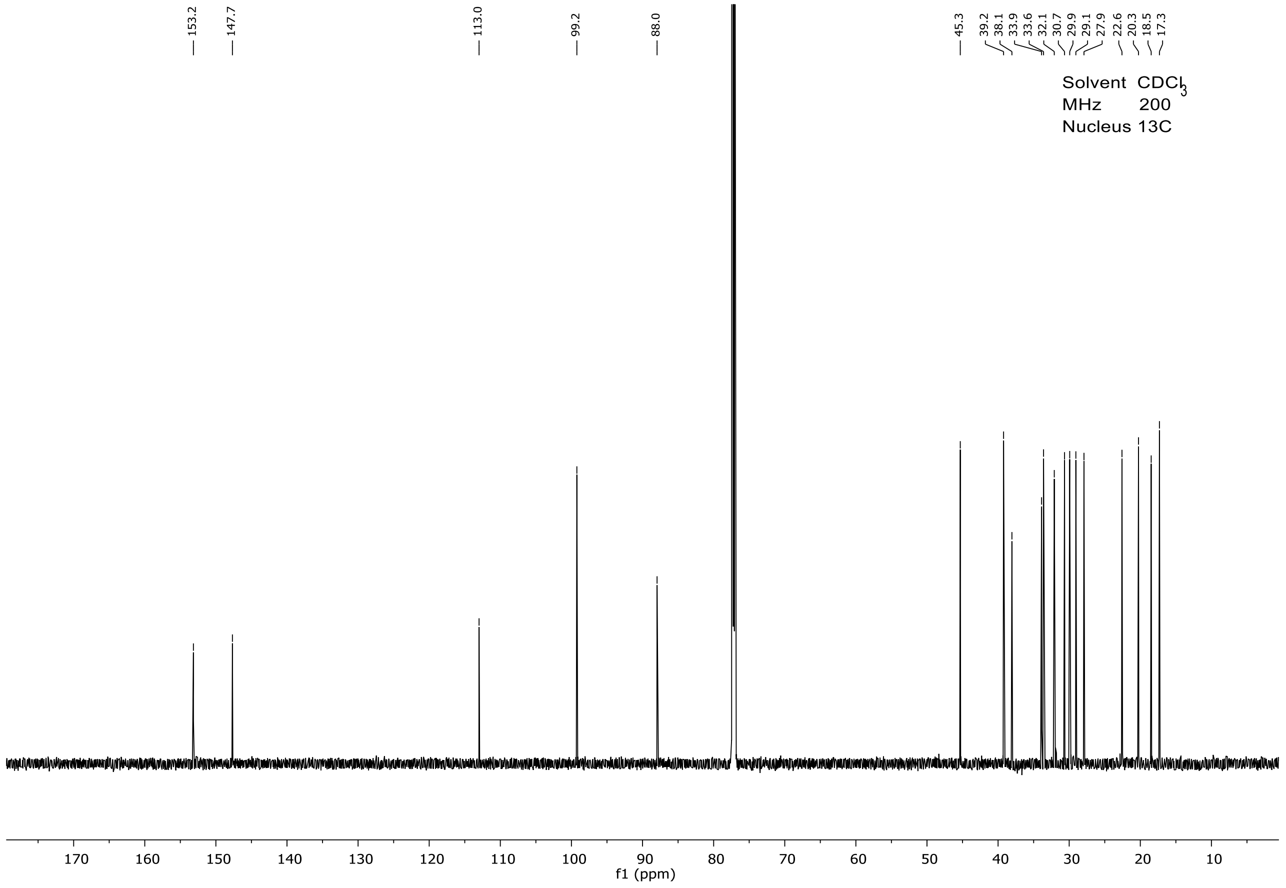

Supplementary Figure 45 ^{1}H and ^{13}C NMR Spectra for **50** in $CDCl_3$.

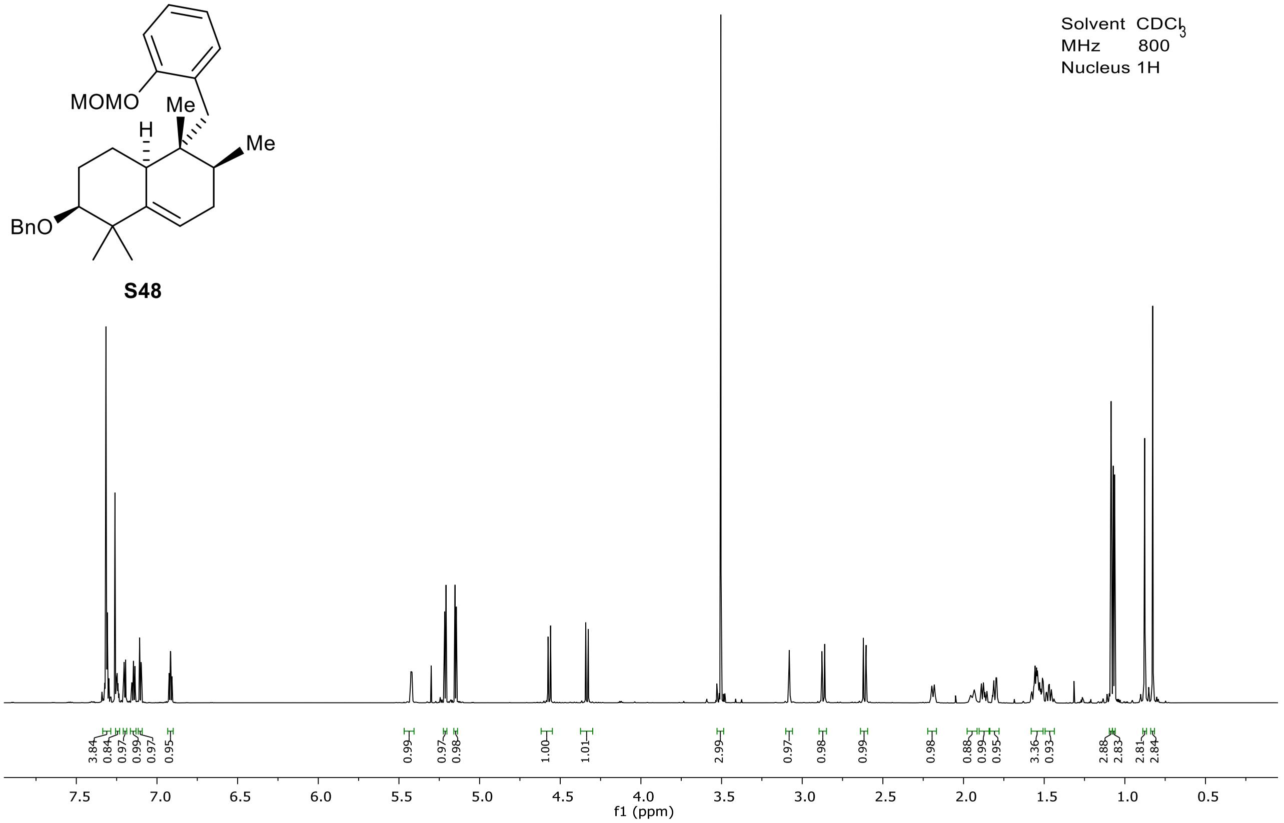

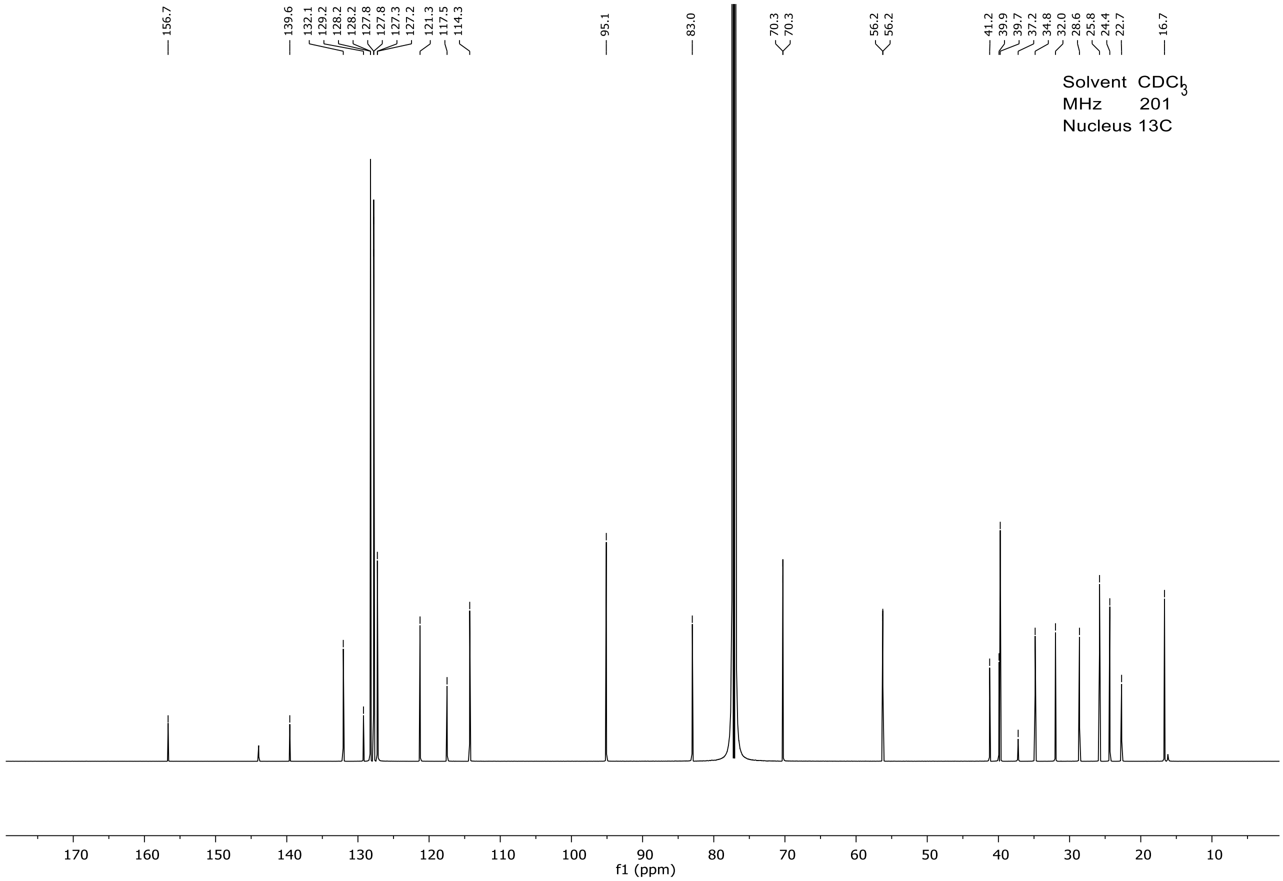

Supplementary Figure 46 ^{1}H and ^{13}C NMR Spectra for **S48** in $CDCl_3$.

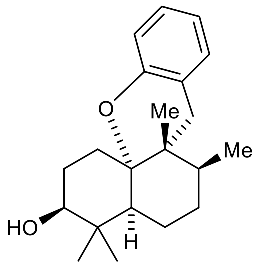

40

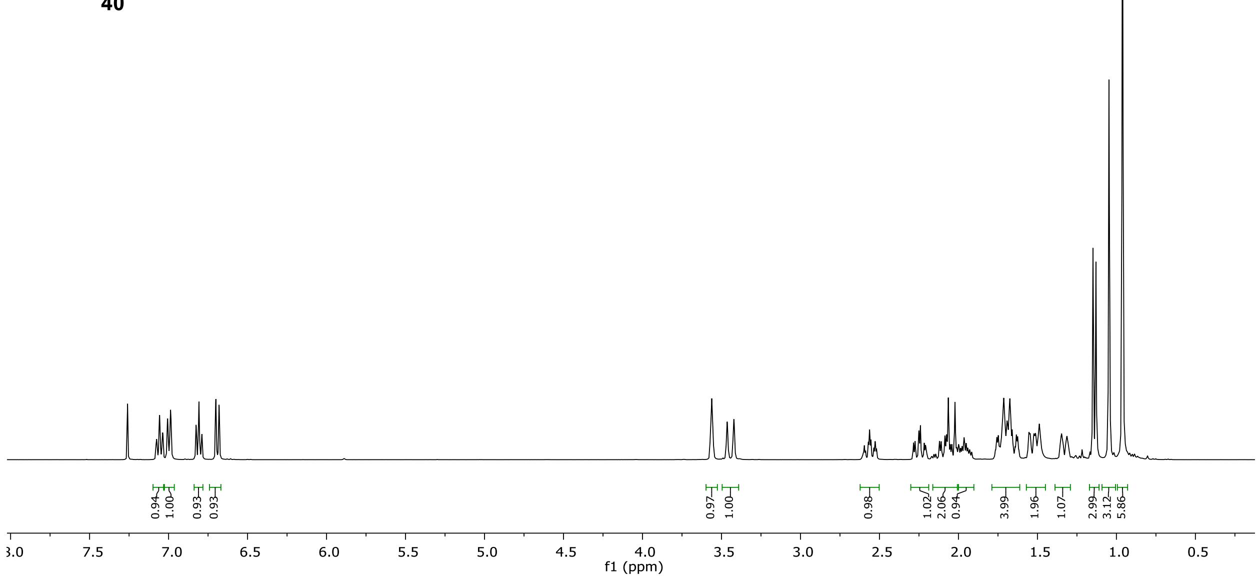

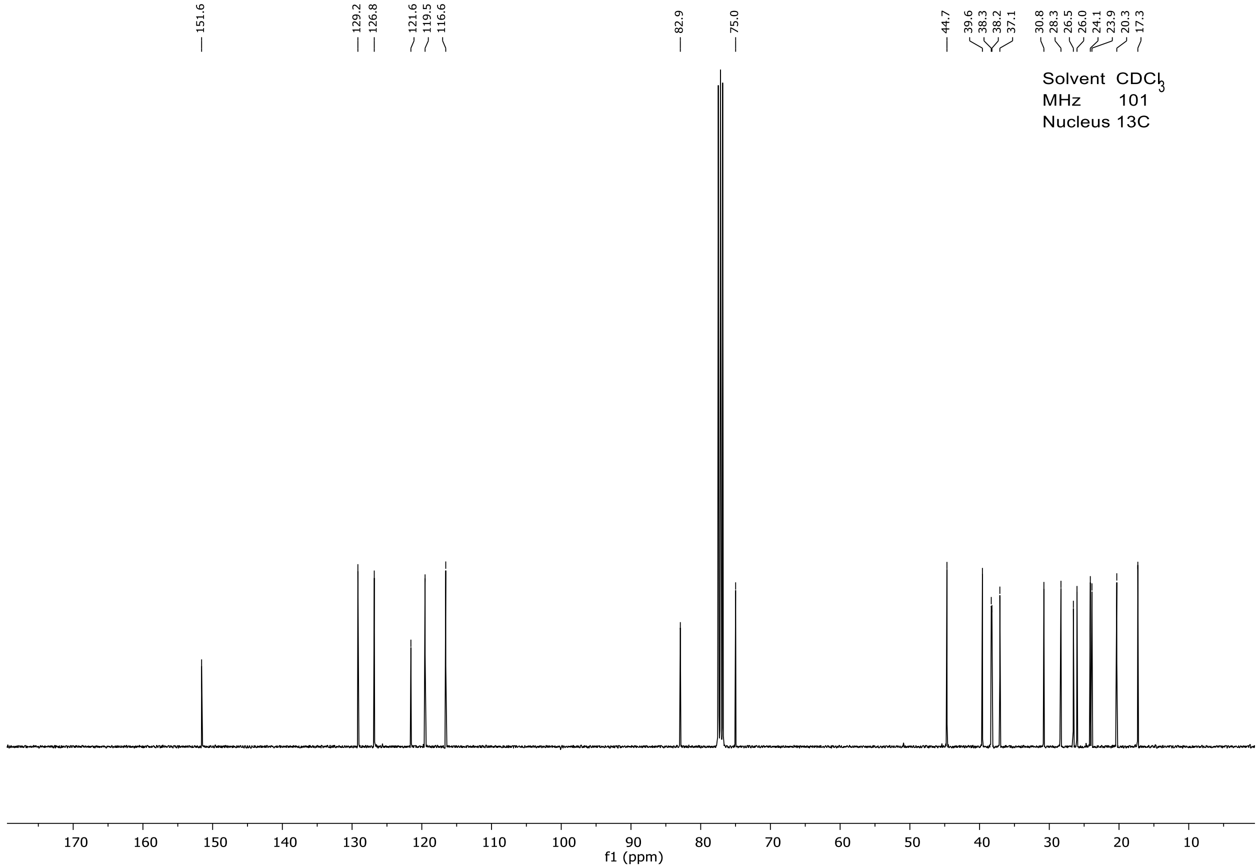

Supplementary Figure 47 ^{1}H and ^{13}C NMR Spectra for **40** in $CDCl_3$.

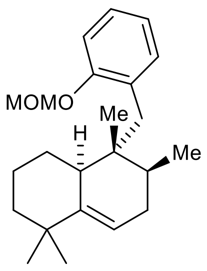

S51

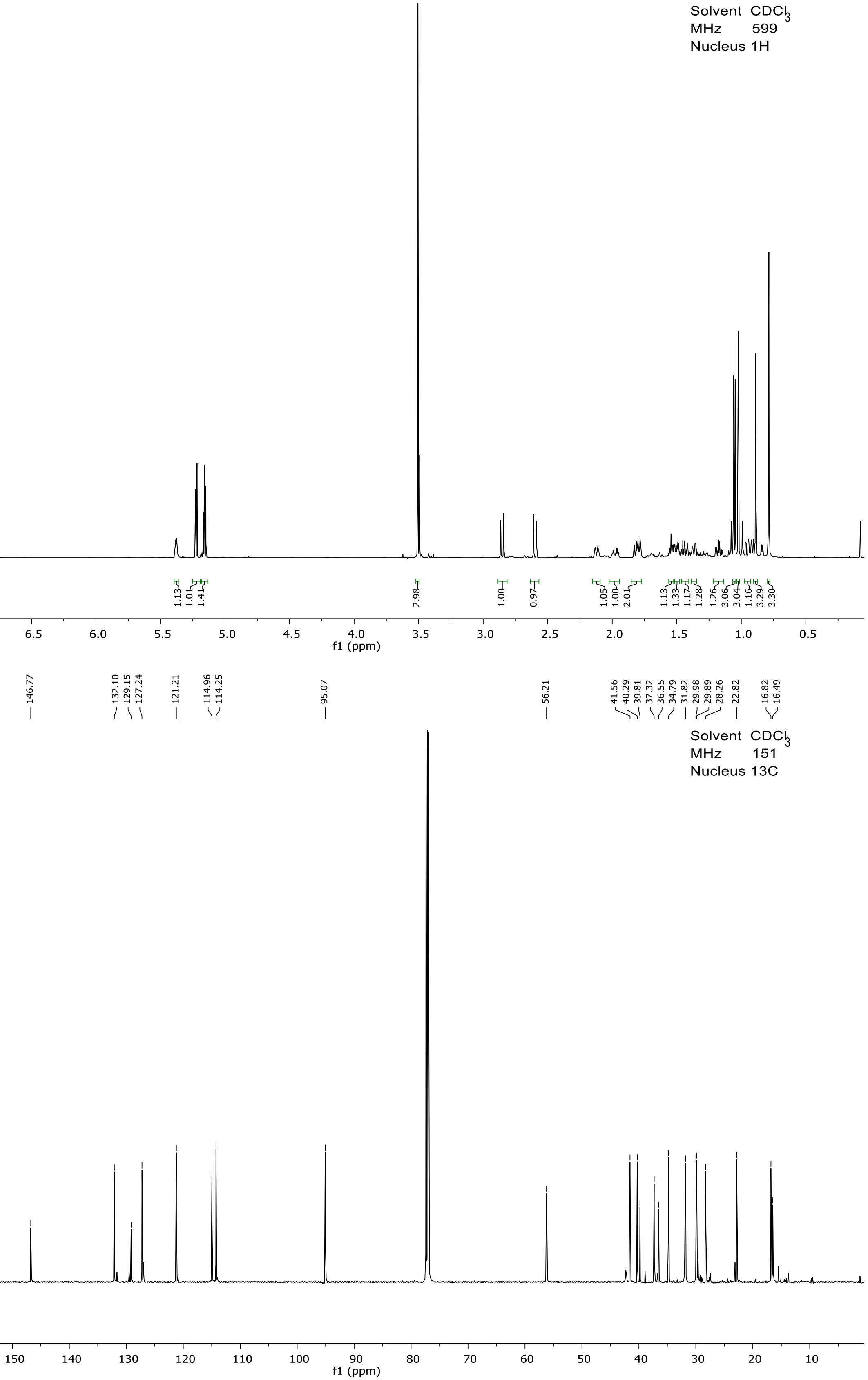

Supplementary Figure 48 ^{1}H and ^{13}C NMR Spectra for **S51** in $CDCl_3$.

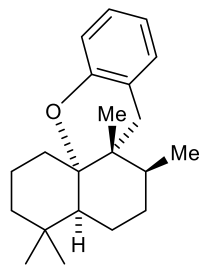

41

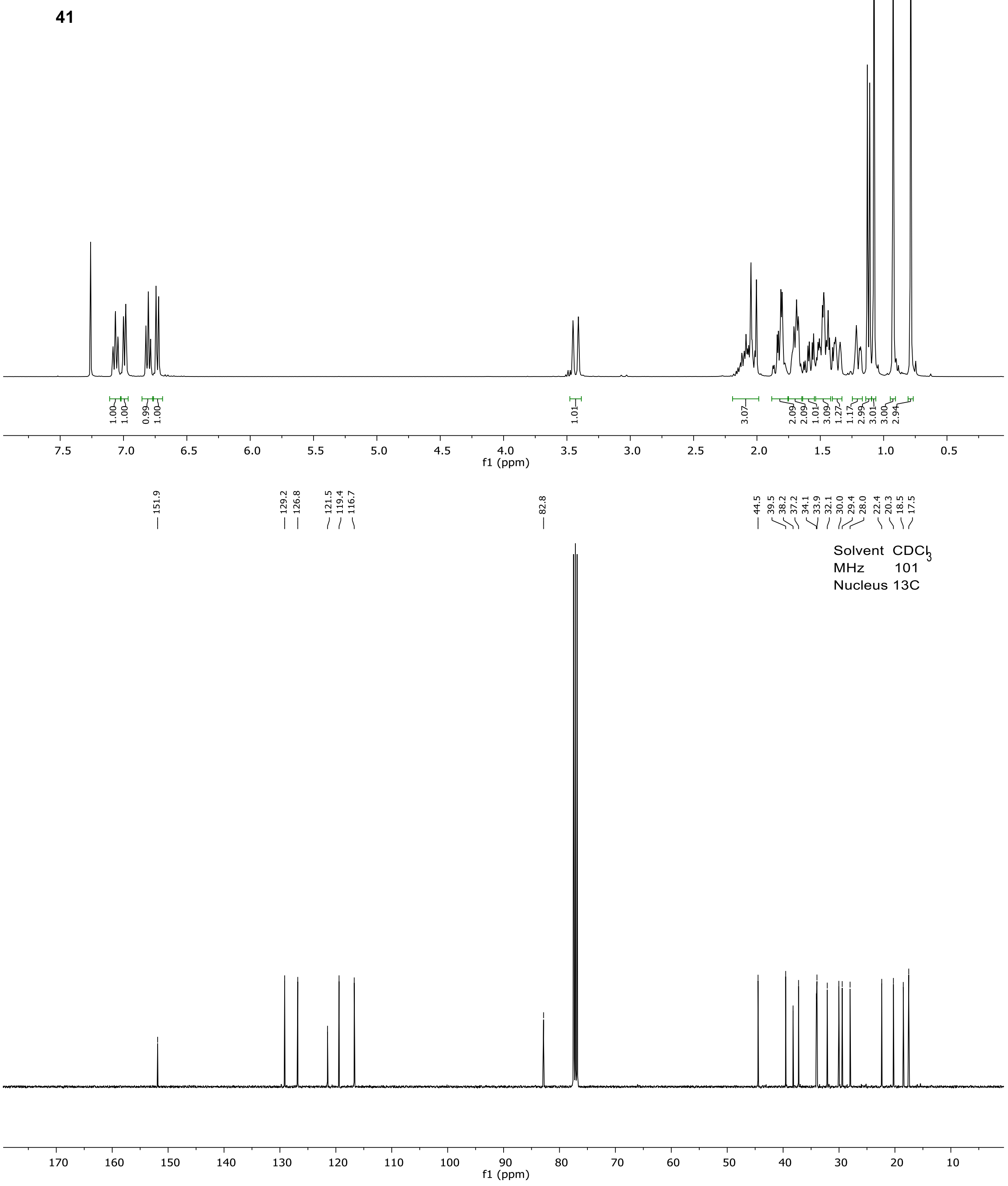

Supplementary Figure 49 ^{1}H and ^{13}C NMR Spectra for **41** in $CDCl_3$.

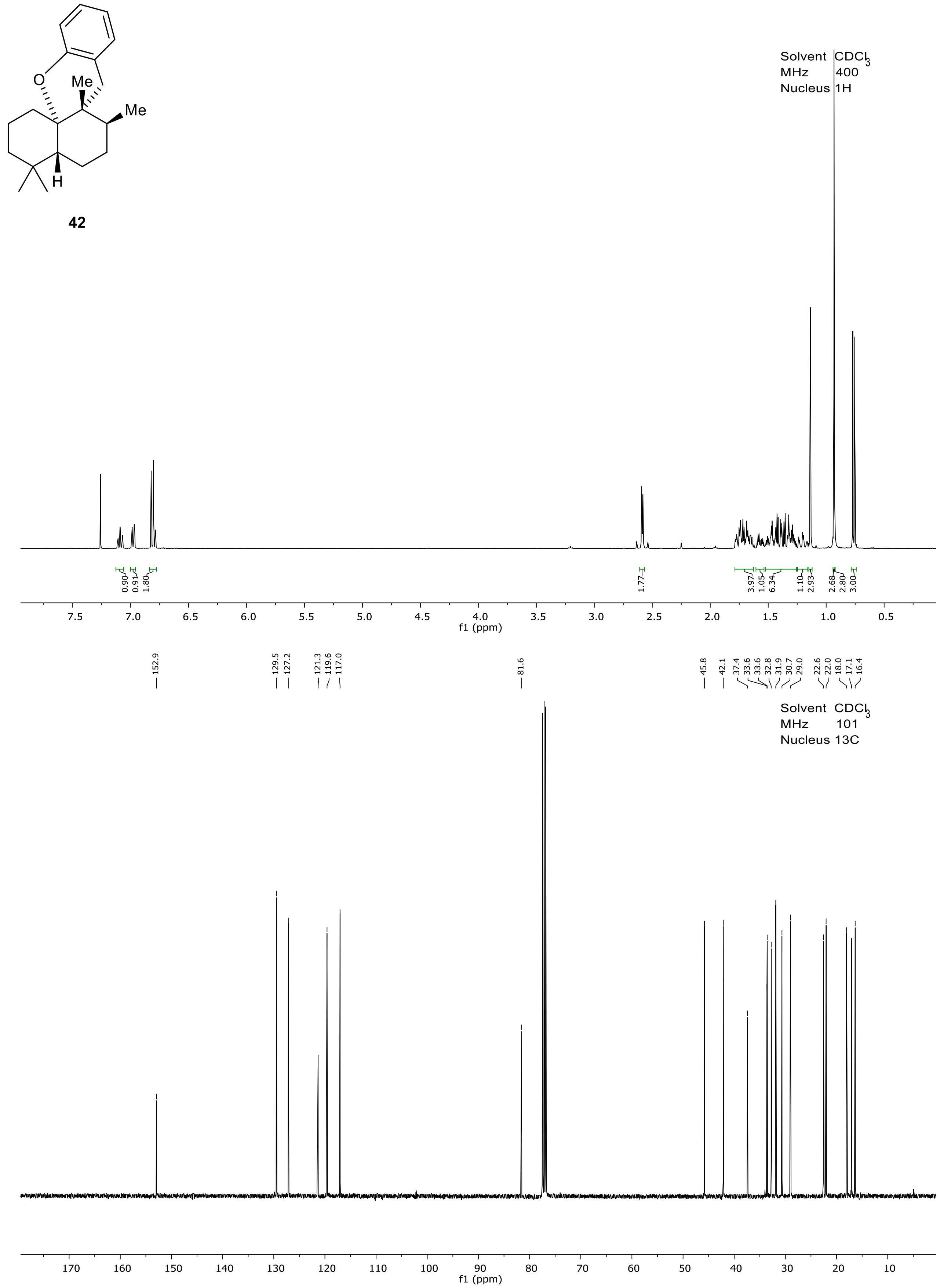

Supplementary Figure 50 ^{1}H and ^{13}C NMR Spectra for **42** in $CDCl_3$.

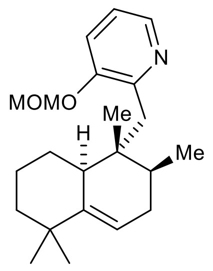

S55

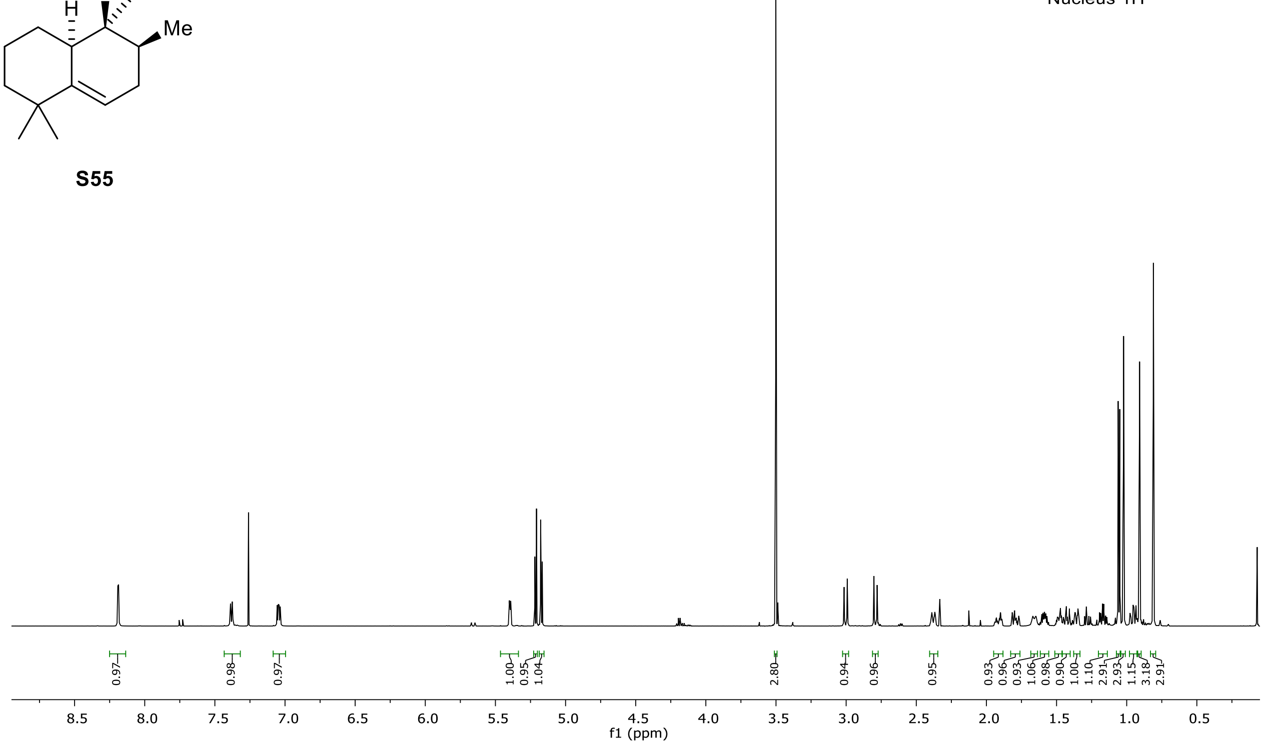

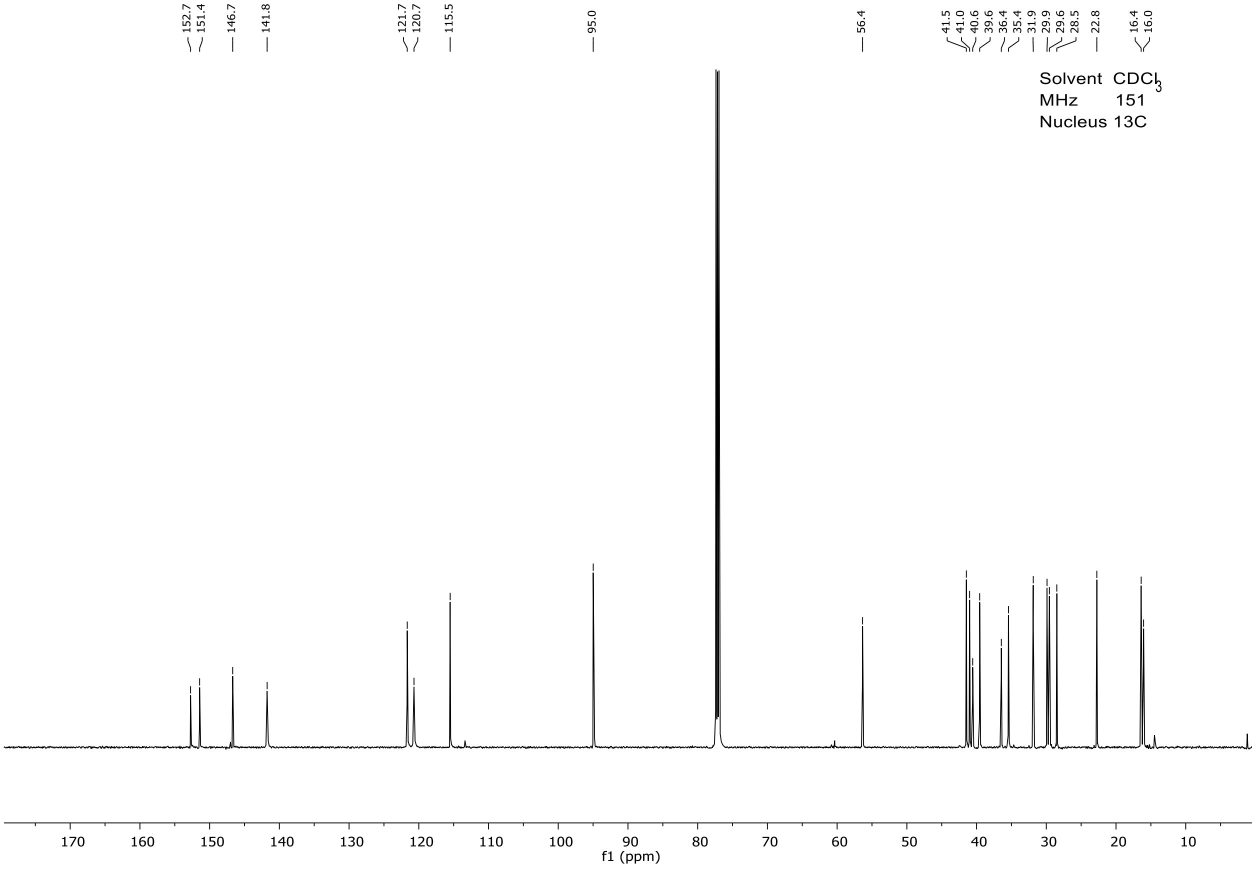

Supplementary Figure 51 ^{1}H and ^{13}C NMR Spectra for **S55** in $CDCl_3$.

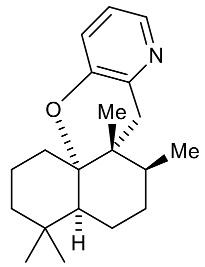

43

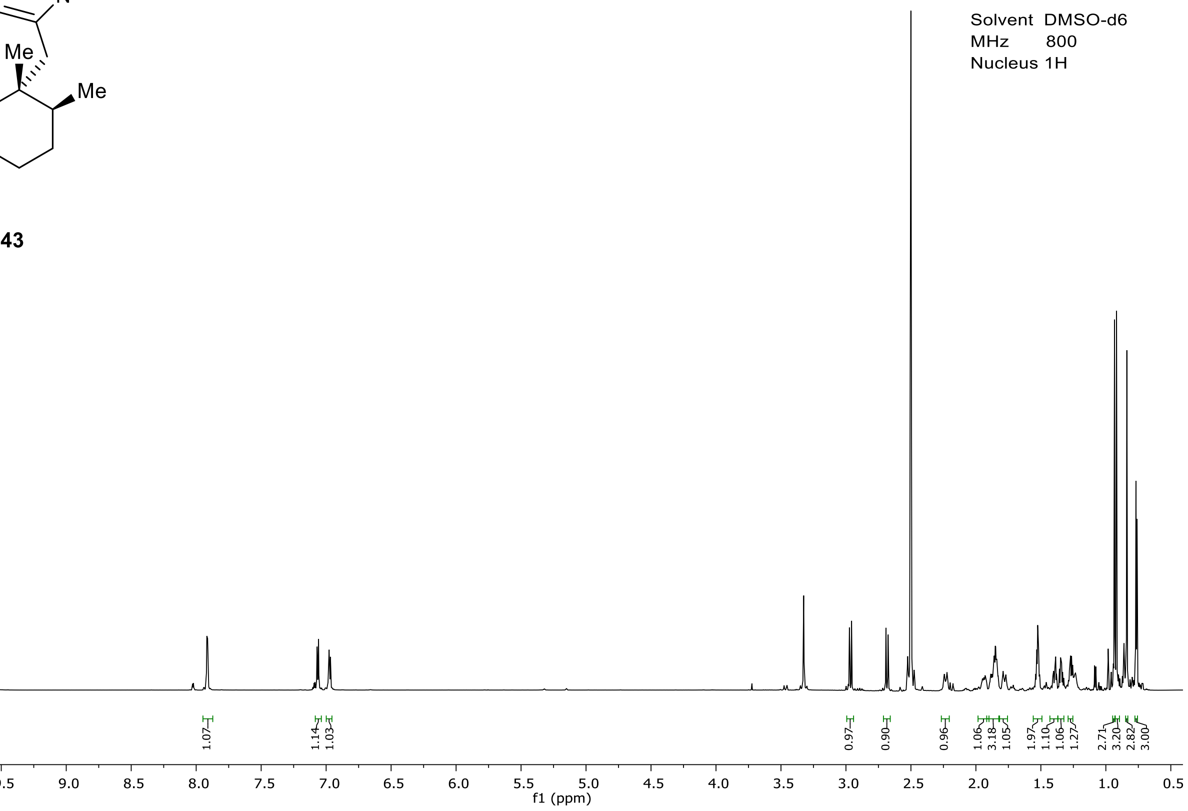

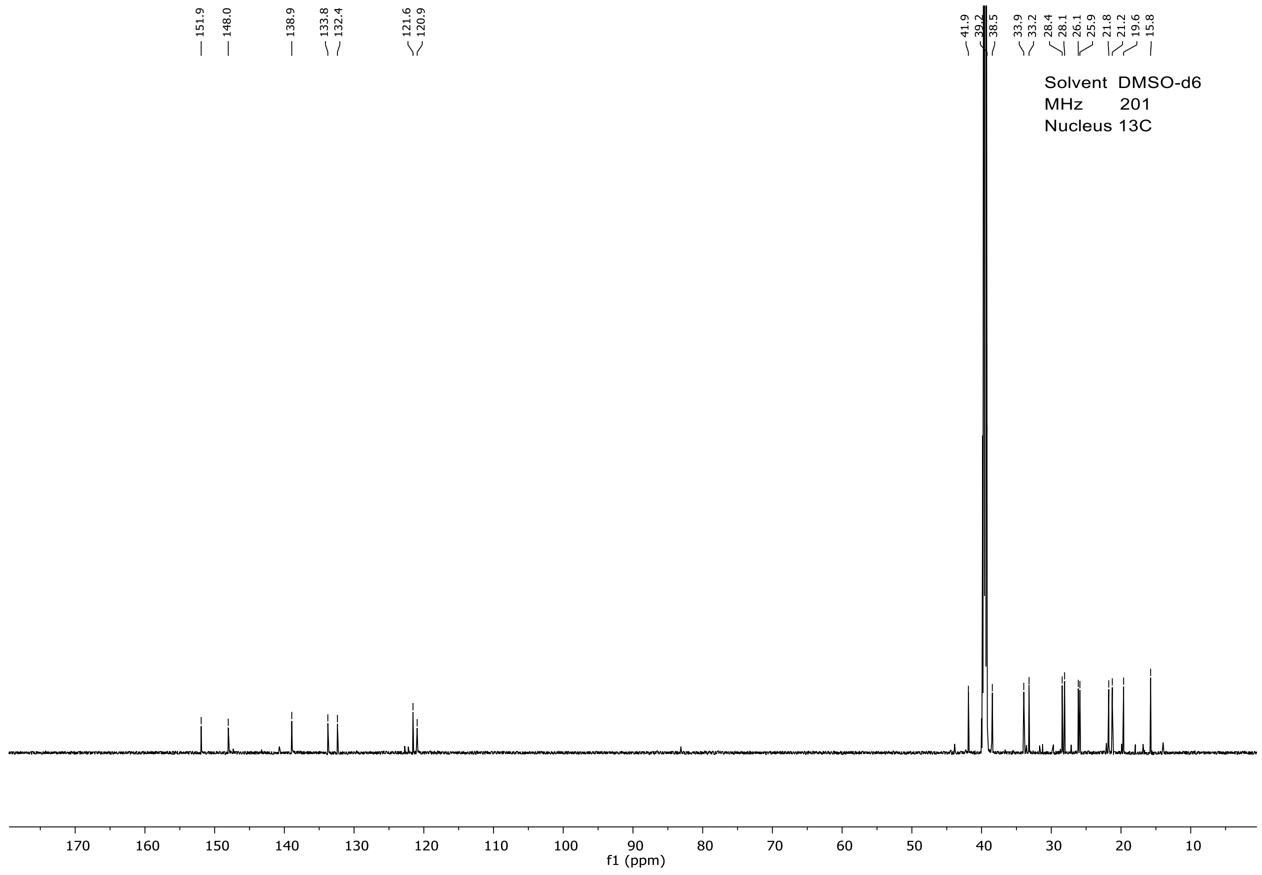

Supplementary Figure 52 ^{1}H and ^{13}C NMR Spectra for **43** in DMSO-d6.

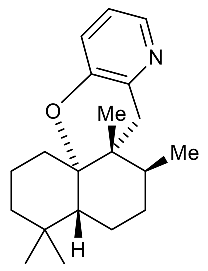

44

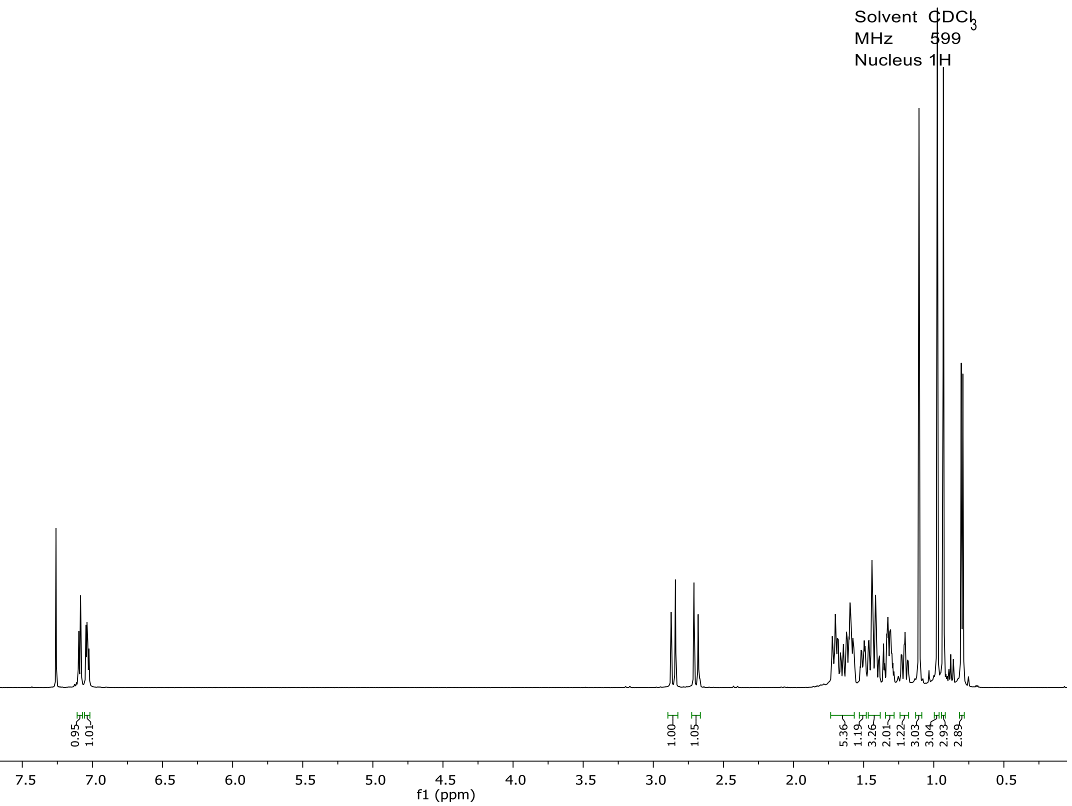

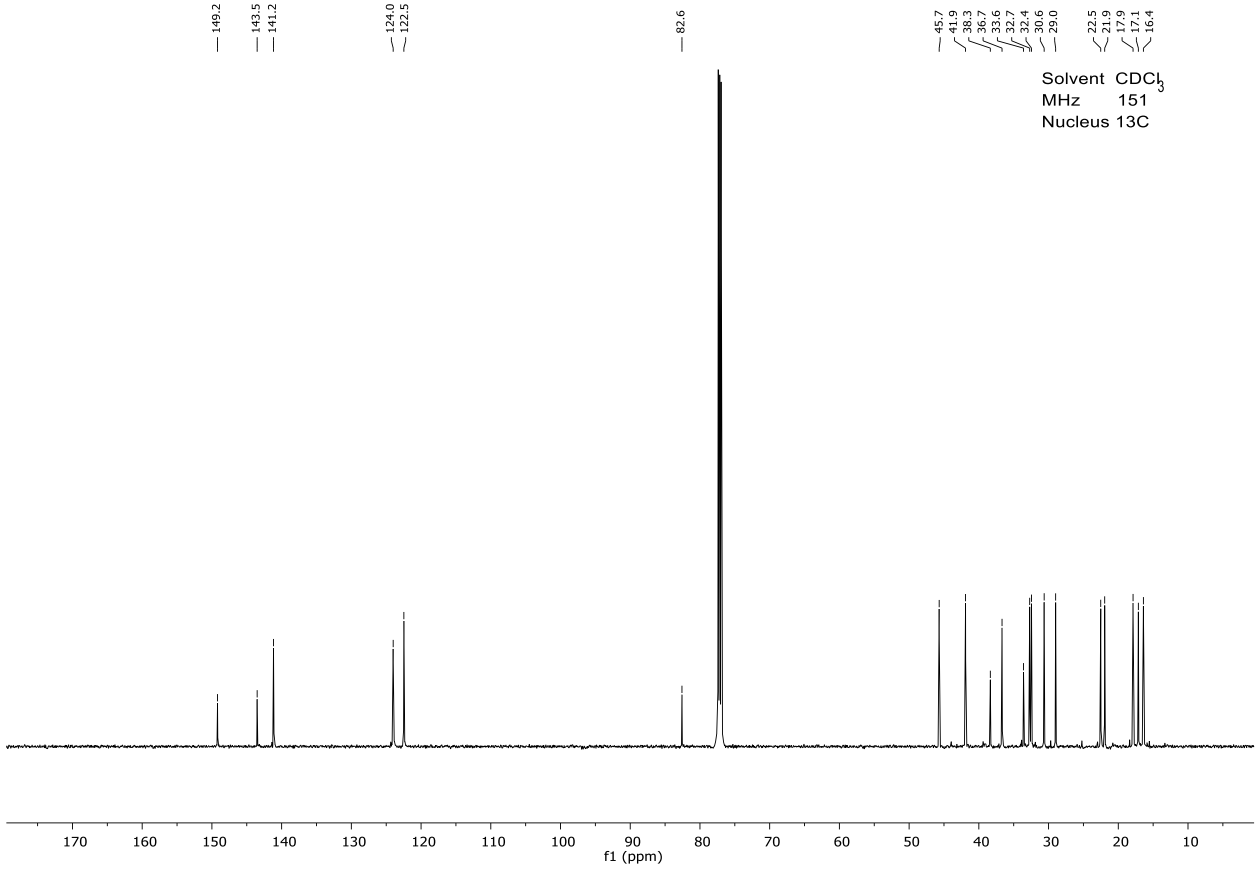

Supplementary Figure 53 ^{1}H and ^{13}C NMR Spectra for **44** in $CDCl_3$.

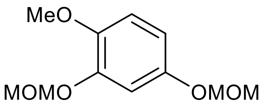

36

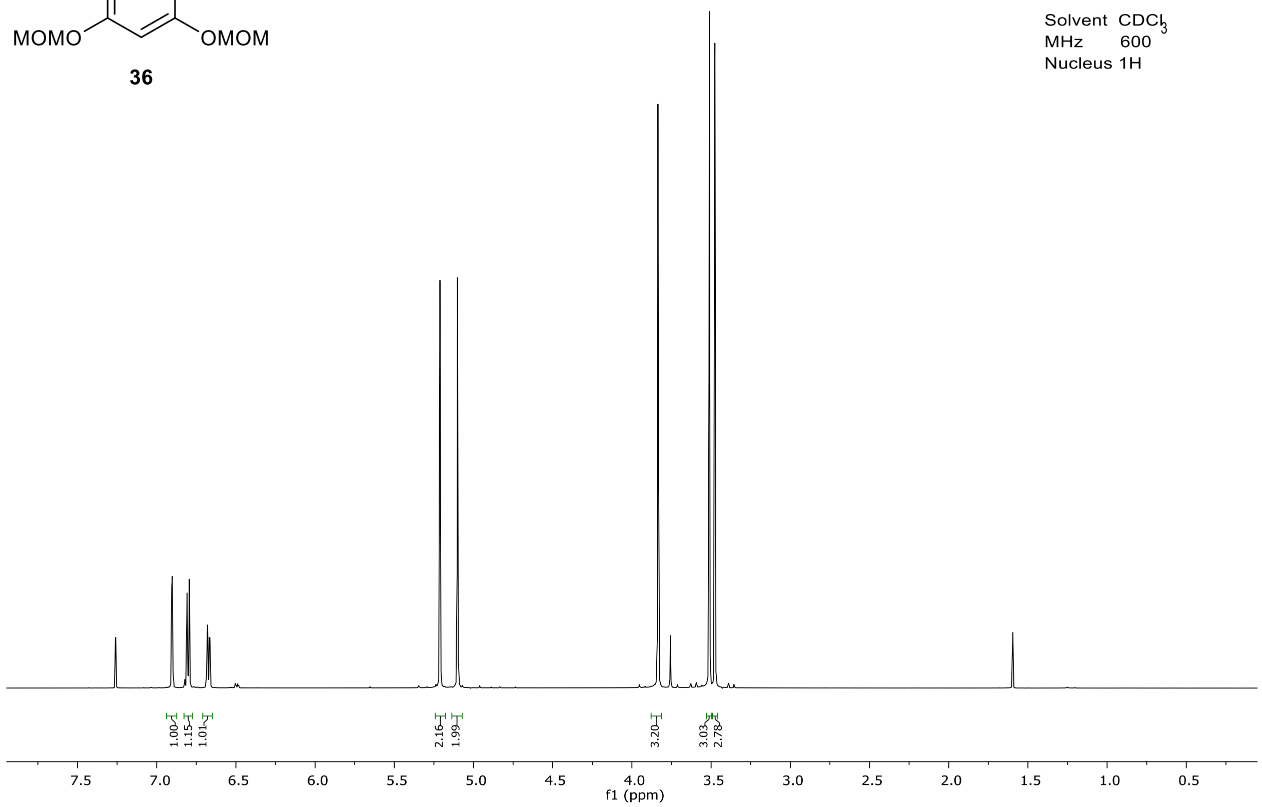

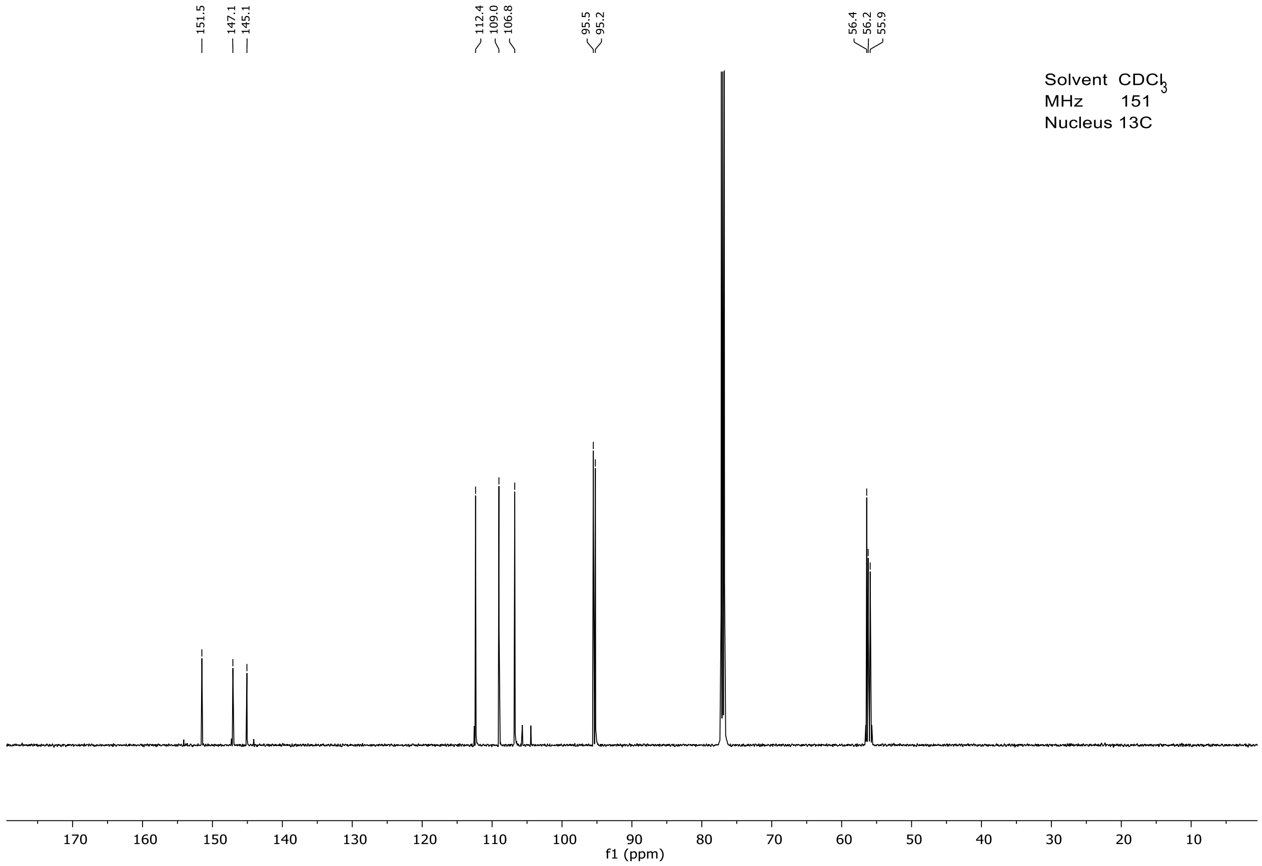

Supplementary Figure 54 ^{1}H and ^{13}C NMR Spectra for **36** in $CDCl_3$.

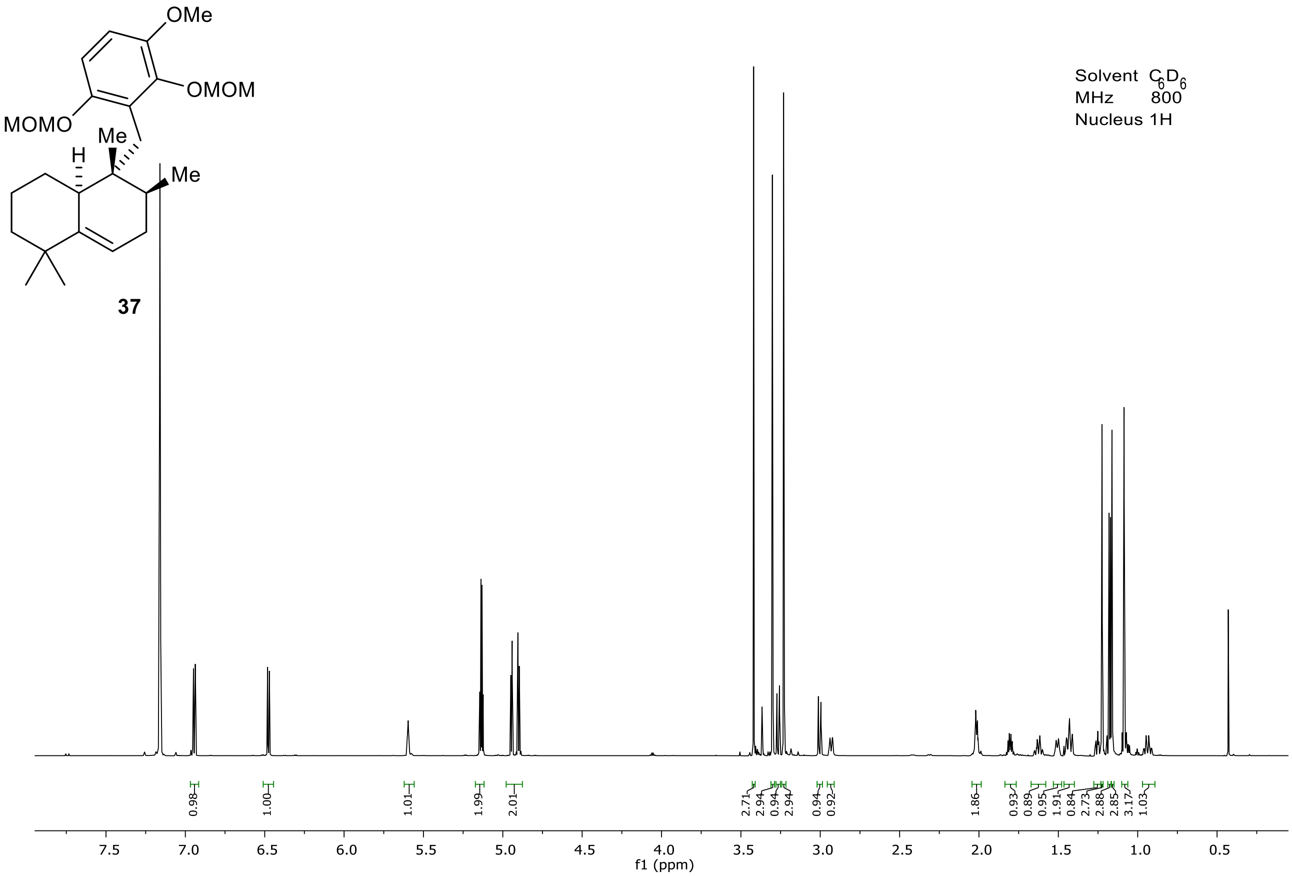

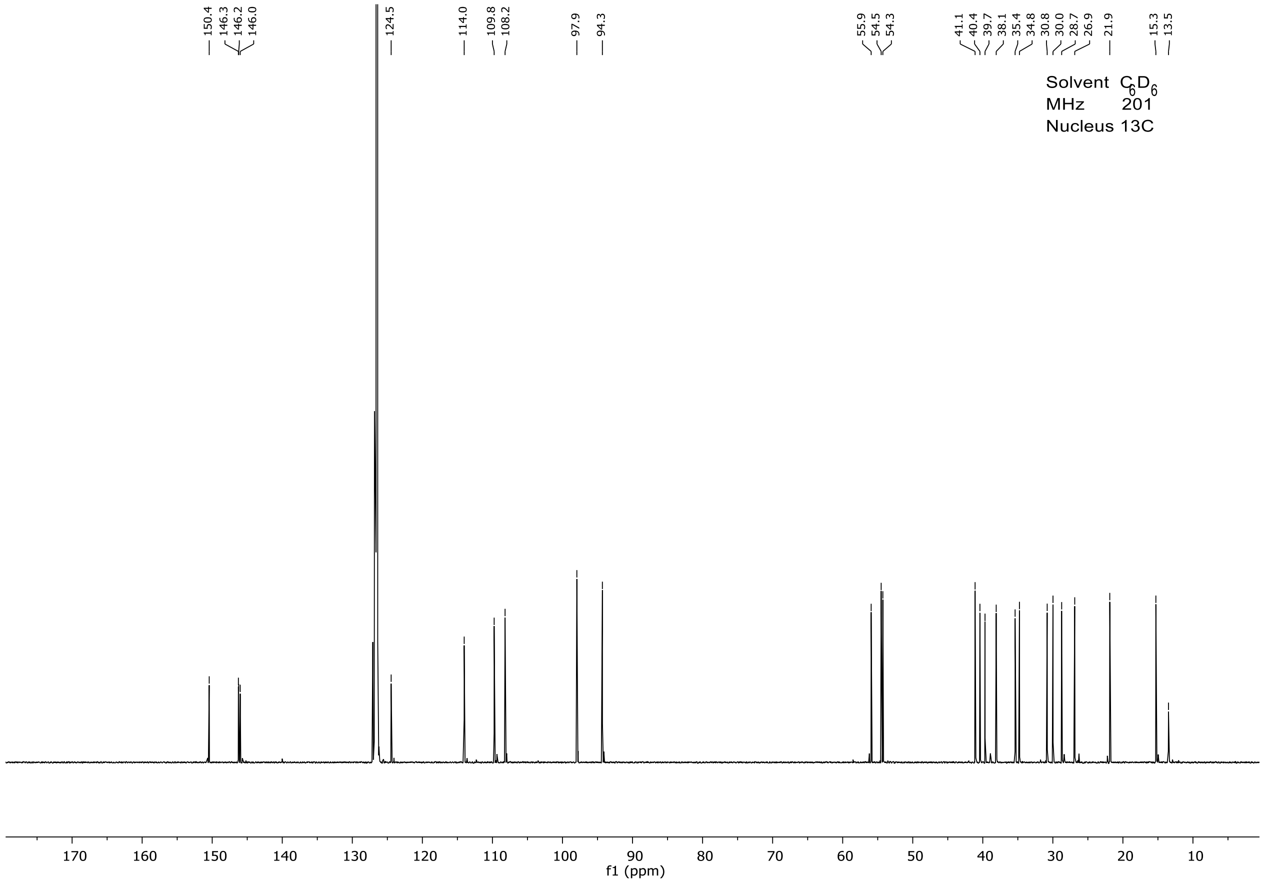

Supplementary Figure 55 ^{1}H and ^{13}C NMR Spectra for **37** in C_6DC_6.

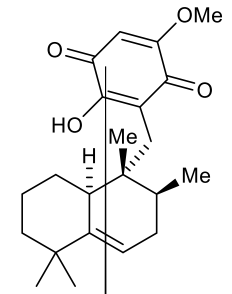

(−)-mamanuthaquinone
(**6**)

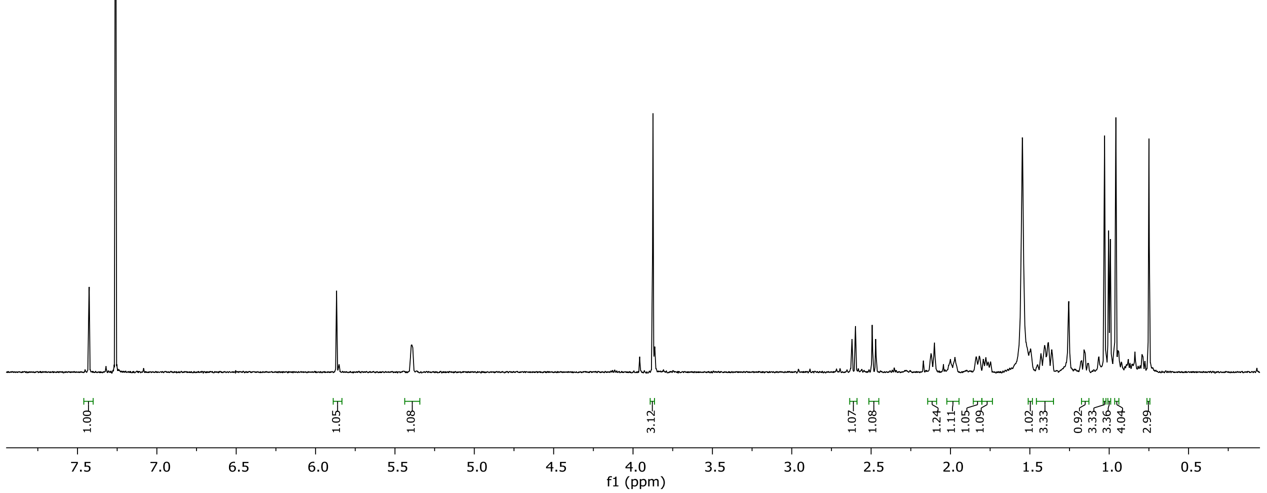

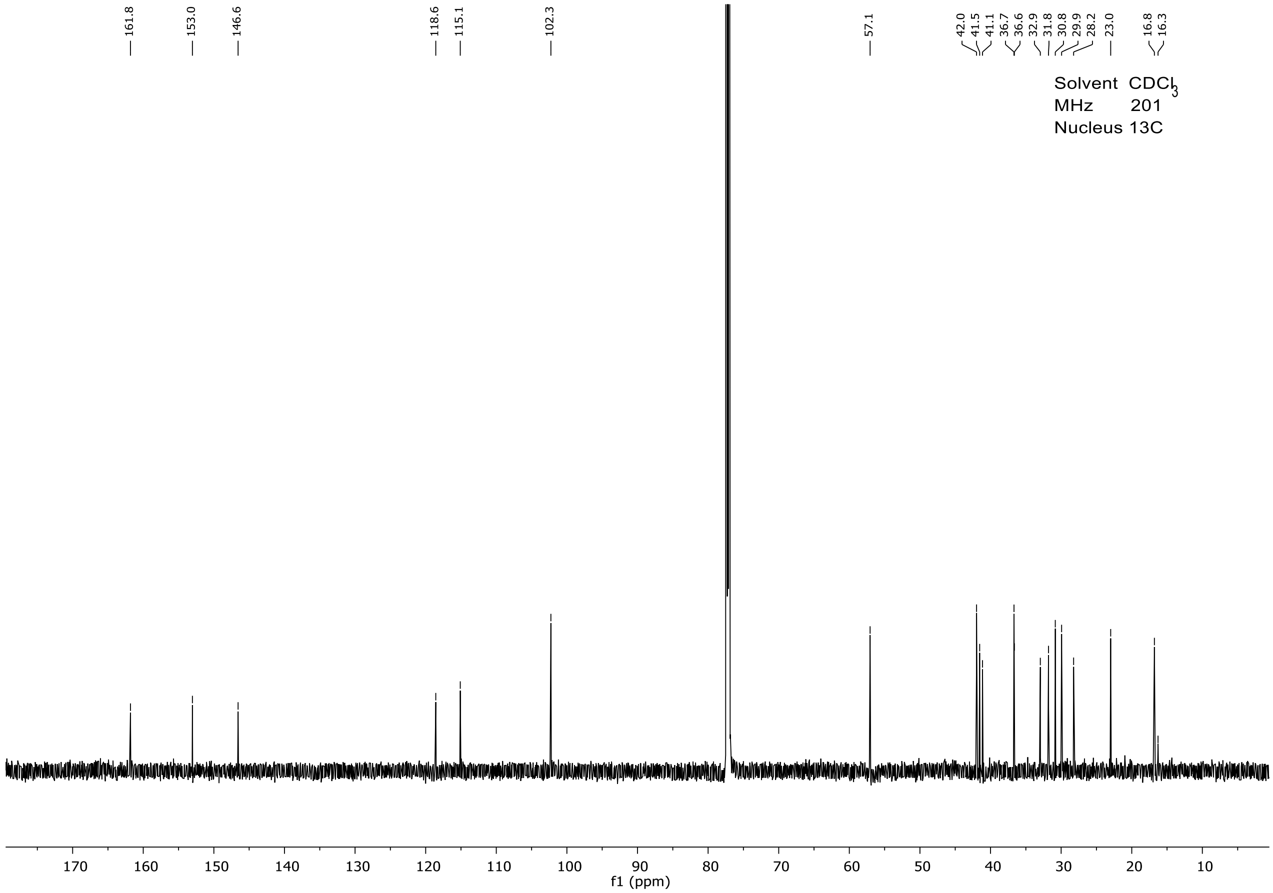

Supplementary Figure 56 ^{1}H and ^{13}C NMR Spectra for **6** in $CDCl_3$.

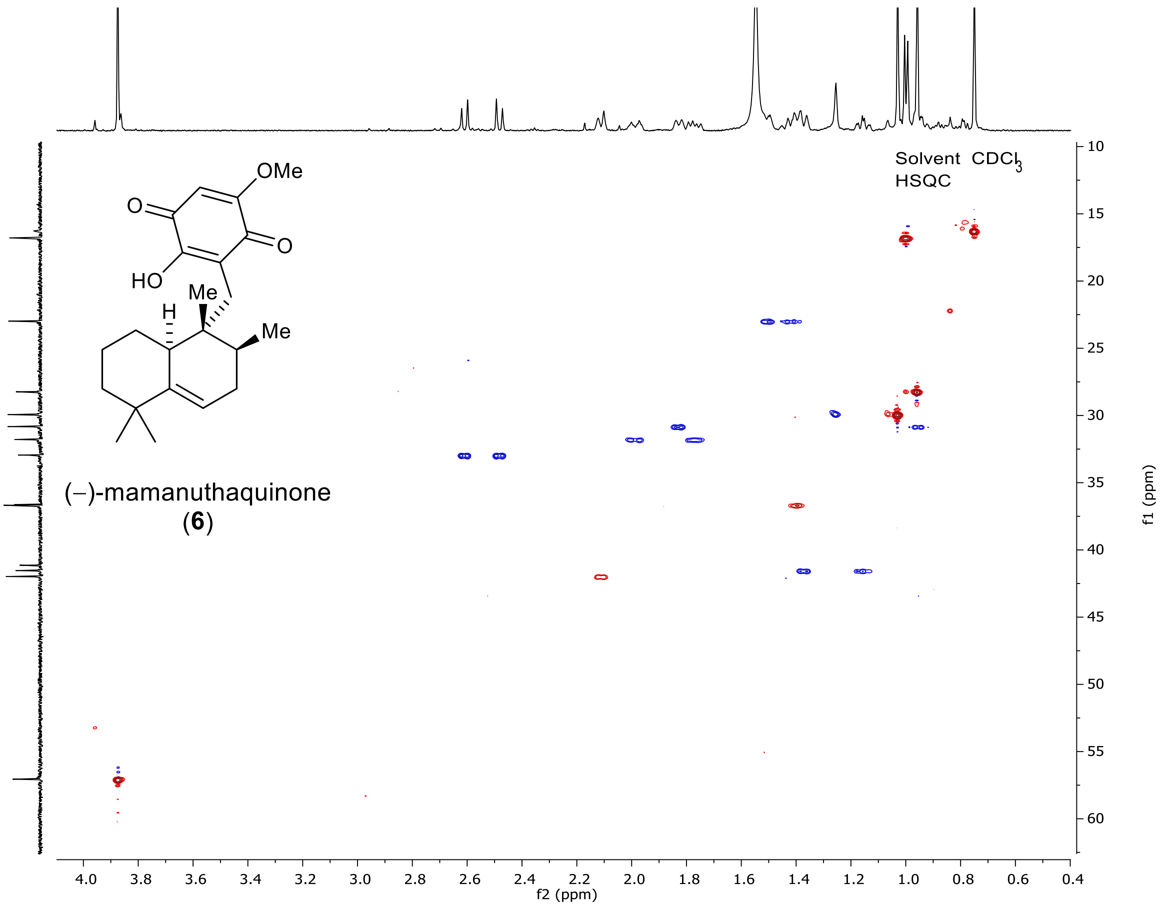

Supplementary Figure 57 HSQC NMR Spectra for **2** in $CDCl_3$.

X-Ray Crystallographic Data

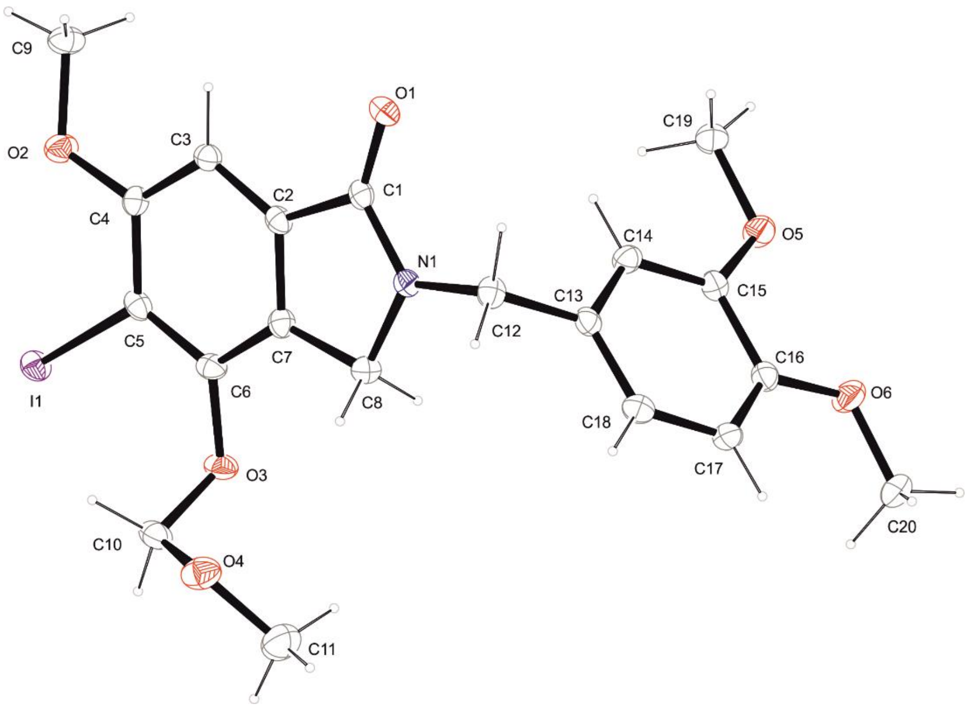

Supplementary Figure 58 CCDC 1534418 contains the supplementary crystallographic data for isoindole **15**. These data can be obtained free of charge from The Cambridge Crystallographic Data Centre *via* www.ccdc.cam.ac.uk/data_request/cif.

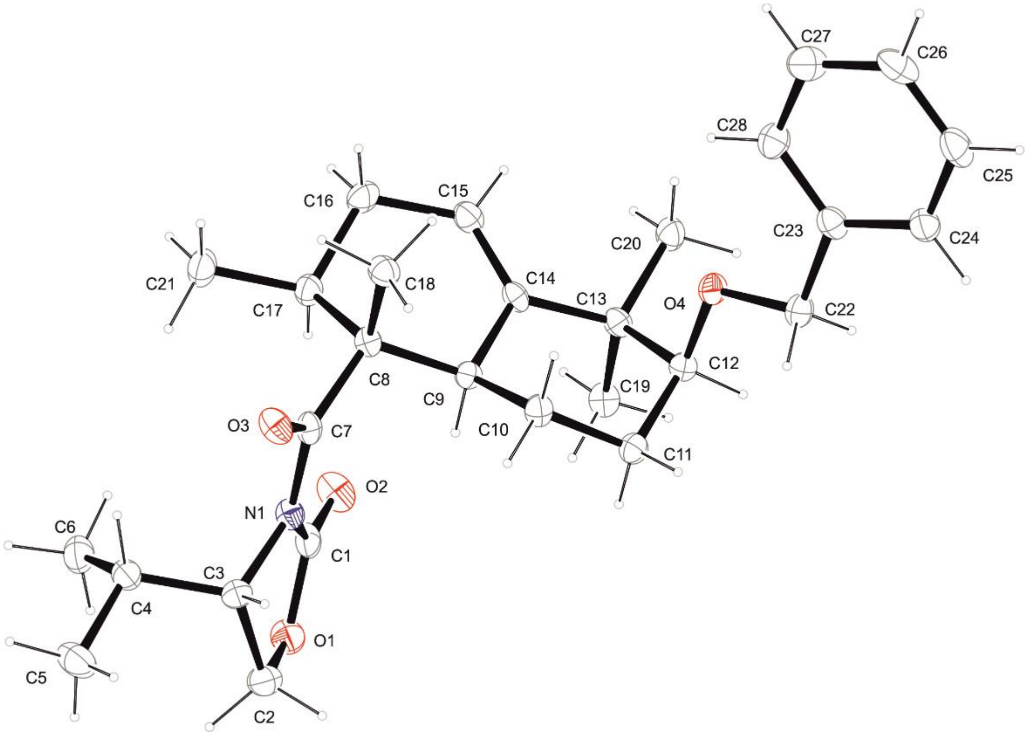

Supplementary Figure 59 **CCDC 1534416** contains the supplementary crystallographic data for oxazolidone **22**. These data can be obtained free of charge from The Cambridge Crystallographic Data Centre *via* www.ccdc.cam.ac.uk/data_request/cif.

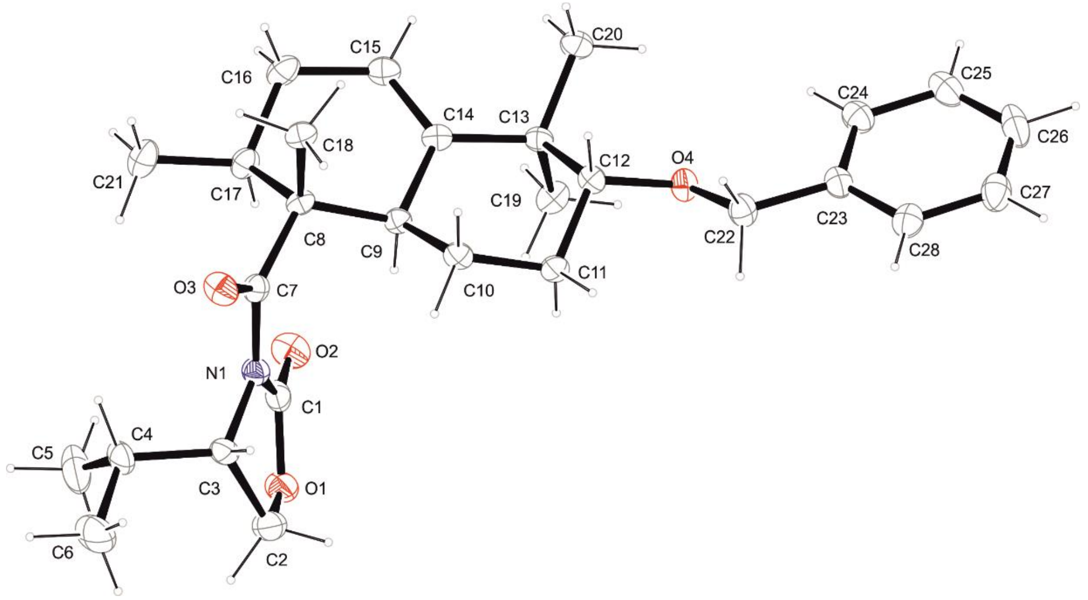

Supplementary Figure 60 CCDC 1534417 contains the supplementary crystallographic data for oxazolidone **S14**. These data can be obtained free of charge from The Cambridge Crystallographic Data Centre *via* www.ccdc.cam.ac.uk/data_request/cif.

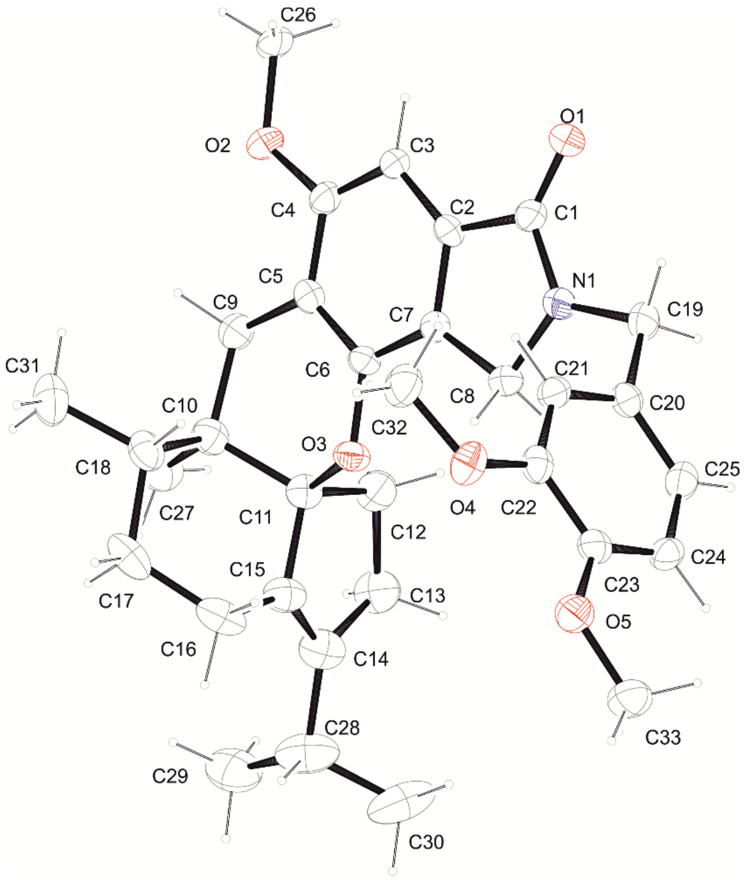

Supplementary Figure 61 CCDC 1534419 contains the supplementary crystallographic data for pentacycle **S19**. These data can be obtained free of charge from The Cambridge Crystallographic Data Centre *via* www.ccdc.cam.ac.uk/data_request/cif.

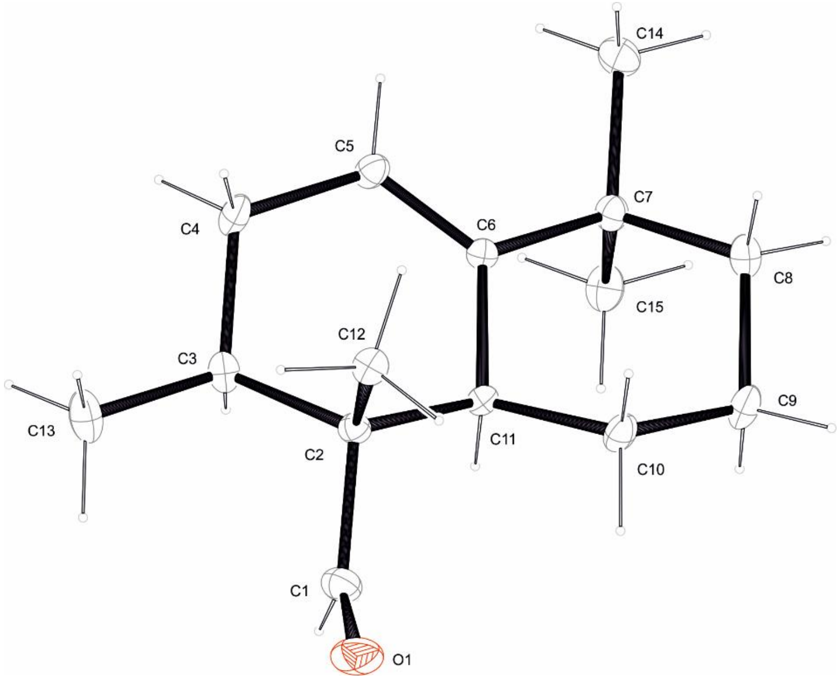

Supplementary Figure 62 CCDC 1534618 contains the supplementary crystallographic data for aldehyde **33**. These data can be obtained free of charge from The Cambridge Crystallographic Data Centre *via* www.ccdc.cam.ac.uk/data_request/cif.

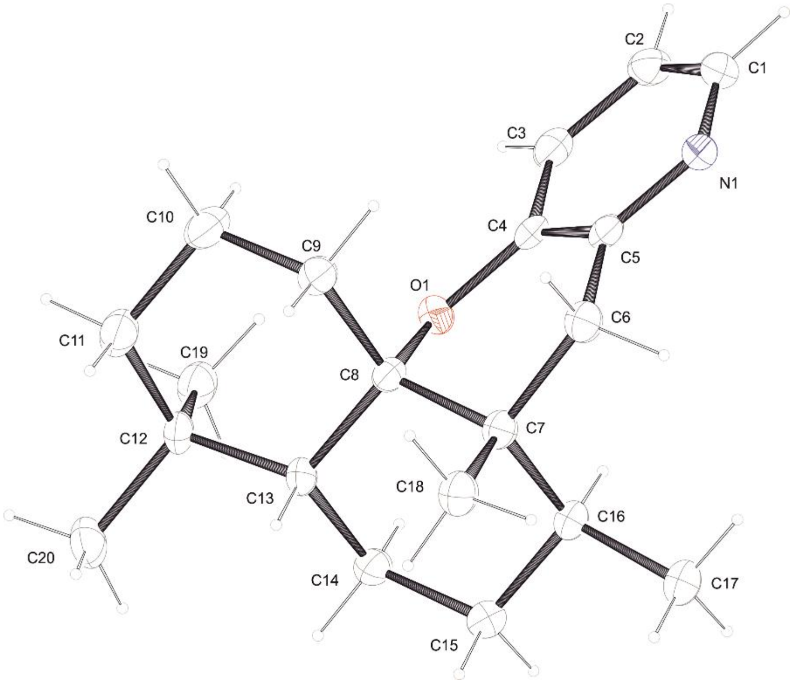

Supplementary Figure 63 CCDC 1534421 contains the supplementary crystallographic data for pyridine **44**. These data can be obtained free of charge from The Cambridge Crystallographic Data Centre *via* www.ccdc.cam.ac.uk/data_request/cif.

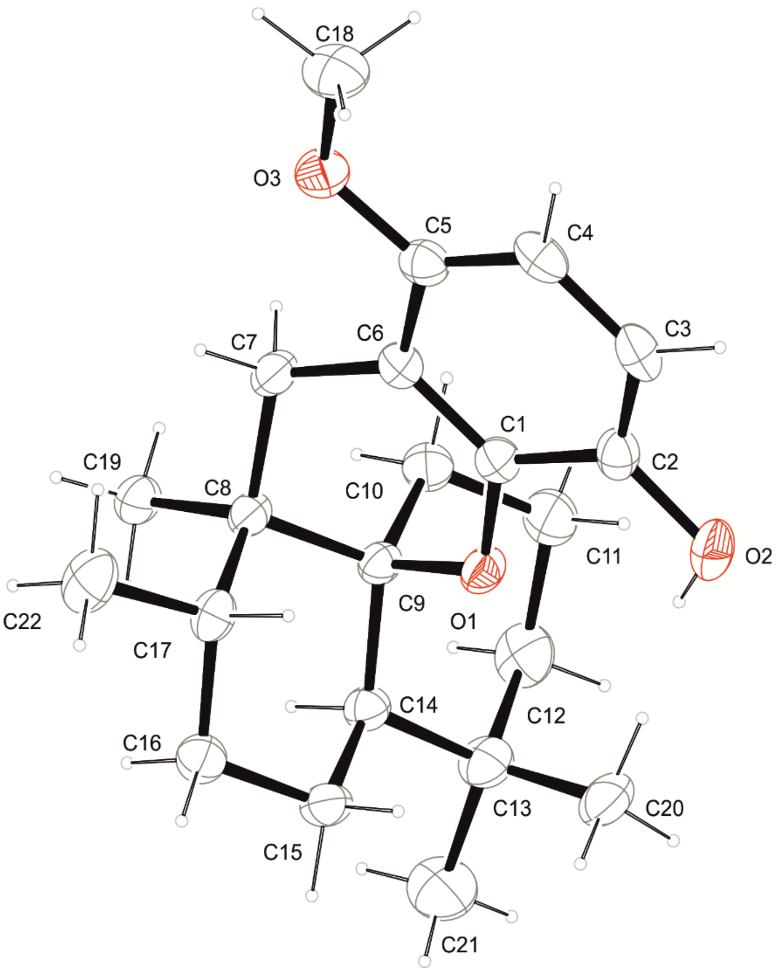

Supplementary Figure 64 CCDC 1509377 contains the supplementary crystallographic data for 5-*epi*-strongylin A (**45**). These data can be obtained free of charge from The Cambridge Crystallographic Data Centre *via* www.ccdc.cam.ac.uk/data_request/cif.

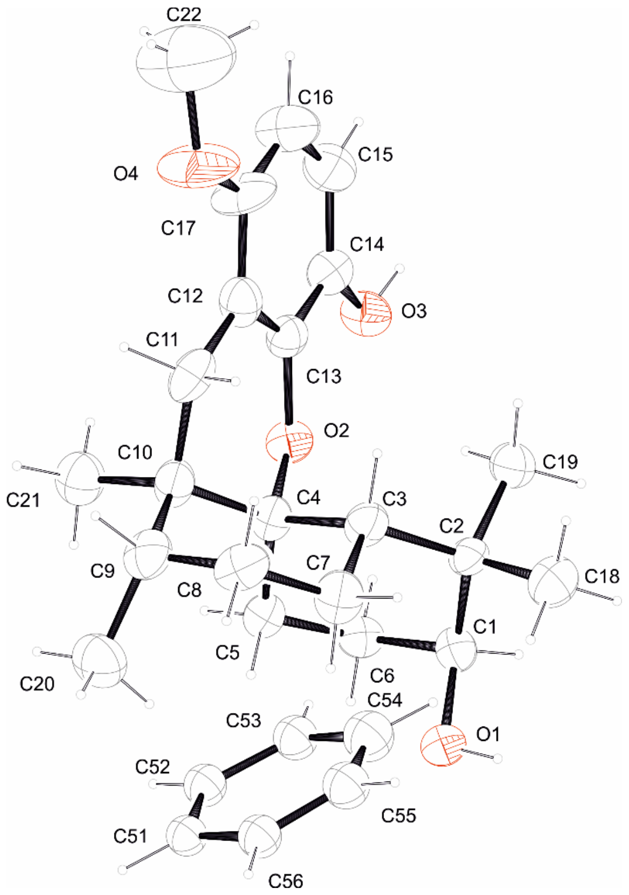

Supplementary Figure 65 CCDC 1534420 contains the supplementary crystallographic data for 3-hydroxy-strongylin A (**48**). These data can be obtained free of charge from The Cambridge Crystallographic Data Centre *via* www.ccdc.cam.ac.uk/data_request/cif.

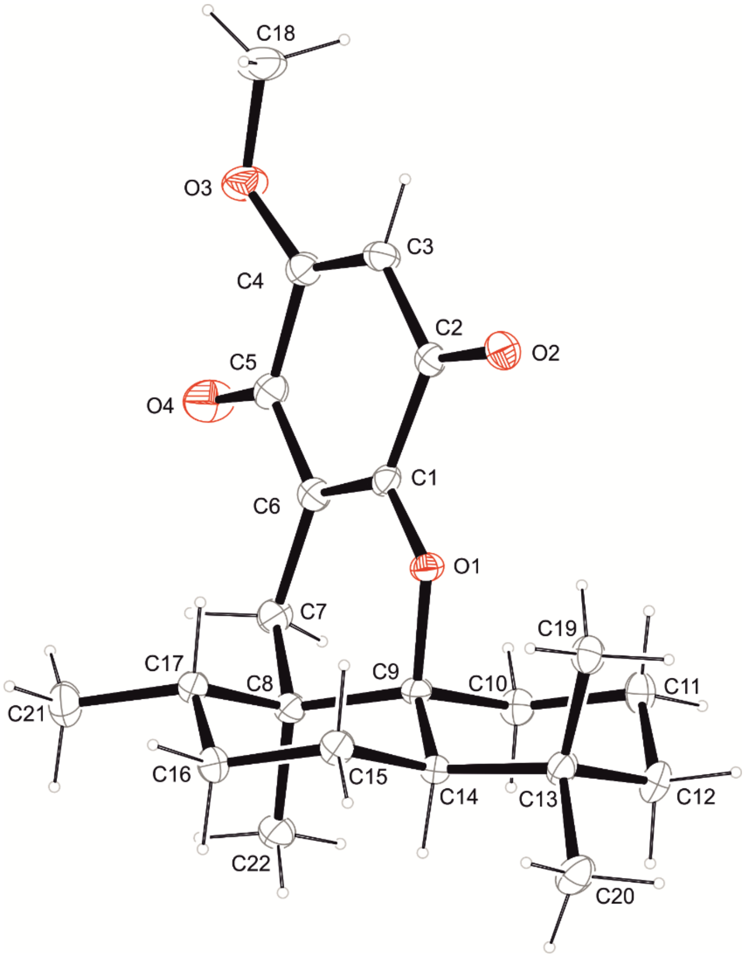

Supplementary Figure 66 CCDC 1534580 contains the supplementary crystallographic data for 5-*epi*-smenoqualone (**51**). These data can be obtained free of charge from The Cambridge Crystallographic Data Centre *via* www.ccdc.cam.ac.uk/data_request/cif.

Supplementary Table 12 Crystallographic data for isoindole 15.

net formula	$C_{20}H_{22}INO_6$
M_r/g mol^{-1}	499.28
crystal size/mm	0.100 × 0.070 × 0.050
T/K	100.(2)
radiation	MoKα
diffractometer	'Bruker D8 Venture TXS'
crystal system	monoclinic
space group	'P 1 21/c 1'
a/Å	11.5389(3)
b/Å	10.9523(3)
c/Å	15.9781(4)
α/°	90
β/°	100.7260(10)
γ/°	90
V/Å^3	1983.99(9)
Z	4
calc. density/g cm^{-3}	1.672
μ/mm^{-1}	1.651
absorption correction	Multi-Scan
transmission factor range	0.6975–0.7461
refls. measured	25091
R_{int}	0.0469
mean σ(I)/I	0.0427
θ range	3.193–30.504
observed refls.	4958
x, y (weighting scheme)	0.0187, 1.1451
hydrogen refinement	constr
refls in refinement	6066
parameters	257
restraints	0
R(F_{obs})	0.0284
R_w(F^2)	0.0624
S	1.046
shift/error$_{max}$	0.002
max electron density/e Å^{-3}	0.642
min electron density/e Å^{-3}	−0.549

Supplementary Table 13 Crystallographic data for oxazolidone 22.

net formula	$C_{28}H_{39}NO_4$
M_r/g mol^{-1}	453.60
crystal size/mm	0.100 × 0.030 × 0.020
T/K	100.(2)
radiation	MoKα
diffractometer	'Bruker D8 Venture TXS'
crystal system	orthorhombic
space group	'P 21 21 21'
a/Å	10.5044(2)
b/Å	13.8351(3)
c/Å	34.2367(8)
α/°	90
β/°	90
γ/°	90
V/Å^3	4975.60(18)
Z	8
calc. density/g cm^{-3}	1.211
μ/mm^{-1}	0.080
absorption correction	Multi-Scan
transmission factor range	0.8985–0.9705
refls. measured	21786
R_{int}	0.0375
mean σ(I)/I	0.0688
θ range	3.176–28.282
observed refls.	9666
x, y (weighting scheme)	0.0342, 1.3785
hydrogen refinement	constr
Flack parameter	0.5(5)
refls in refinement	11844
parameters	607
restraints	0
$R(F_{obs})$	0.0494
$R_w(F^2)$	0.1085
S	1.025
shift/error$_{max}$	0.001
max electron density/e Å^{-3}	0.288
min electron density/e Å^{-3}	−0.246

Supplementary Table 14 Crystallographic data for oxazolidone S13.

net formula	$C_{28}H_{39}NO_4$
M_r/g mol^{-1}	453.60
crystal size/mm	0.100 × 0.060 × 0.020
T/K	100.(2)
radiation	MoKα
diffractometer	'Bruker D8 Venture TXS'
crystal system	monoclinic
space group	'C 1 2 1'
a/Å	21.1557(11)
b/Å	6.9835(3)
c/Å	18.5788(10)
α/°	90
β/°	113.077(2)
γ/°	90
V/Å^3	2525.2(2)
Z	4
calc. density/g cm^{-3}	1.193
μ/mm^{-1}	0.079
absorption correction	Multi-Scan
transmission factor range	0.8764–0.9705
refls. measured	11053
R_{int}	0.0308
mean σ(I)/I	0.0629
θ range	3.362–28.280
observed refls.	5097
x, y (weighting scheme)	0.0364, 0.7835
hydrogen refinement	constr
Flack parameter	−0.8(6)
refls in refinement	6217
parameters	304
restraints	1
$R(F_{obs})$	0.0475
$R_w(F^2)$	0.0986
S	1.022
shift/error$_{max}$	0.001
max electron density/e Å^{-3}	0.247
min electron density/e Å^{-3}	−0.184

Supplementary Table 15 Crystallographic data for pentacycle S18.

net formula	$C_{33}H_{41}NO_5$
M_r/g mol^{-1}	531.67
crystal size/mm	0.080 × 0.070 × 0.030
T/K	100.(2)
radiation	MoKα
diffractometer	'Bruker D8 Venture TXS'
crystal system	orthorhombic
space group	'P 21 21 21'
a/Å	9.2680(3)
b/Å	13.5377(6)
c/Å	22.8966(10)
α/°	90
β/°	90
γ/°	90
V/Å^3	2872.8(2)
Z	4
calc. density/g cm^{-3}	1.229
μ/mm^{-1}	0.082
absorption correction	Multi-Scan
transmission factor range	0.9145–0.9705
refls. measured	22538
R_{int}	0.0659
mean σ(*I*)/*I*	0.0688
θ range	3.138–26.360
observed refls.	4535
x, y (weighting scheme)	0.0462, 0.6830
hydrogen refinement	constr
Flack parameter	0.6(8)
refls in refinement	5852
parameters	359
restraints	0
$R(F_{obs})$	0.0516
$R_w(F^2)$	0.1189
S	1.042
shift/error$_{max}$	0.001
max electron density/e Å^{-3}	0.231
min electron density/e Å^{-3}	−0.239

Supplementary Table 16 Crystallographic data for aldehyde 33.

net formula	$C_{15}H_{24}O$
M_r/g mol^{-1}	220.34
crystal size/mm	0.100 × 0.070 × 0.050
T/K	153.(2)
radiation	MoKα
diffractometer	'Bruker D8 Venture TXS'
crystal system	monoclinic
space group	'P 1 21 1'
a/Å	7.1180(3)
b/Å	8.2644(3)
c/Å	11.4134(5)
α/°	90
β/°	94.5133(16)
γ/°	90
V/Å^3	669.32(5)
Z	2
calc. density/g cm^{-3}	1.093
μ/mm^{-1}	0.066
absorption correction	Multi-Scan
transmission factor range	0.9195–0.9593
refls. measured	7512
R_{int}	0.0204
mean σ(I)/I	0.0292
θ range	3.262–28.255
observed refls.	2940
x, y (weighting scheme)	0.0457, 0.1045
hydrogen refinement	constr
Flack parameter	−0.2(4)
refls in refinement	3068
parameters	149
restraints	1
$R(F_{obs})$	0.0328
$R_w(F^2)$	0.0910
S	1.068
shift/error$_{max}$	0.001
max electron density/e Å^{-3}	0.223
min electron density/e Å^{-3}	−0.160

Supplementary Table 17 Crystallographic data for pyridine 43.

net formula	$C_{20}H_{29}NO$
M_r/g mol^{-1}	299.44
crystal size/mm	0.100 × 0.030 × 0.020
T/K	100.(2)
radiation	MoKα
diffractometer	'Bruker D8 Venture TXS'
crystal system	orthorhombic
space group	'P 21 21 21'
a/Å	6.5144(10)
b/Å	15.165(2)
c/Å	16.776(2)
α/°	90
β/°	90
γ/°	90
V/Å^3	1657.3(4)
Z	4
calc. density/g cm^{-3}	1.200
μ/mm^{-1}	0.072
absorption correction	Multi-Scan
transmission factor range	0.7177–0.9705
refls. measured	13220
R_{int}	0.0975
mean σ(*I*)/*I*	0.0852
θ range	3.355–25.328
observed refls.	2336
x, y (weighting scheme)	0.0453, 0.7034
hydrogen refinement	constr
Flack parameter	0.3(10)
refls in refinement	3019
parameters	203
restraints	0
$R(F_{obs})$	0.0576
$R_w(F^2)$	0.1354
S	1.062
shift/error$_{max}$	0.001
max electron density/e Å^{-3}	0.182
min electron density/e Å^{-3}	−0.274

Supplementary Table 18 Crystallographic data for 5-*epi*-strongylin A (44).

net formula	$C_{22}H_{32}O_3$
M_r/g mol^{-1}	344.47
crystal size/mm	0.100 × 0.040 × 0.030
T/K	153.(2)
radiation	MoKα
diffractometer	'Bruker D8 Venture TXS'
crystal system	orthorhombic
space group	'P 21 21 21'
a/Å	9.7896(4)
b/Å	10.6910(5)
c/Å	18.2352(10)
α/°	90
β/°	90
γ/°	90
V/Å^3	1908.51(16)
Z	4
calc. density/g cm^{-3}	1.199
μ/mm^{-1}	0.078
absorption correction	Multi-Scan
transmission factor range	0.9329–0.9705
refls. measured	22500
R_{int}	0.0375
mean σ(*I*)/*I*	0.0317
θ range	3.600–28.276
observed refls.	4252
x, y (weighting scheme)	0.0464, 0.3655
hydrogen refinement	C-H constr, O-H refall
Flack parameter	0.3(4)
refls in refinement	4731
parameters	235
restraints	0
$R(F_{obs})$	0.0395
$R_w(F^2)$	0.0985
S	1.042
shift/error$_{max}$	0.001
max electron density/e Å^{-3}	0.249
min electron density/e Å^{-3}	−0.216

Supplementary Table 19 Crystallographic data for 3-hydroxy-strongylin A (47).

net formula	$C_{25}H_{35}O_4$
M_r/g mol^{-1}	399.53
crystal size/mm	0.080 × 0.060 × 0.020
T/K	173.(2)
radiation	MoKα
diffractometer	'Bruker D8 Venture TXS'
crystal system	monoclinic
space group	'P 1 21 1'
a/Å	13.5556(9)
b/Å	13.8798(11)
c/Å	15.6721(14)
α/°	90
β/°	103.108(4)
γ/°	90
V/Å^3	2871.9(4)
Z	4
calc. density/g cm^{-3}	0.924
μ/mm^{-1}	0.061
absorption correction	Multi-Scan
transmission factor range	0.7873–0.9705
refls. measured	8128
R_{int}	0.1132
mean σ(I)/I	0.1198
θ range	3.225–23.256
observed refls.	5284
x, y (weighting scheme)	0.1620, 0.0
hydrogen refinement	constr
Flack parameter	0.0(10)
refls in refinement	8128
parameters	532
restraints	7
$R(F_{obs})$	0.0931
$R_w(F^2)$	0.2635
S	1.035
shift/error$_{max}$	0.001
max electron density/e Å^{-3}	0.568
min electron density/e Å^{-3}	−0.305

Supplementary Table 20 Crystallographic data for 5-*epi*-smenoqualone (50).

net formula	$C_{22}H_{30}O_4$
M_r/g mol^{-1}	358.46
crystal size/mm	0.080 × 0.050 × 0.030
T/K	100.(2)
radiation	MoKα
diffractometer	'Bruker D8 Venture TXS'
crystal system	orthorhombic
space group	'P 21 21 21'
a/Å	6.77460(10)
b/Å	11.7096(3)
c/Å	23.4581(6)
α/°	90
β/°	90
γ/°	90
V/Å^3	1860.88(7)
Z	4
calc. density/g cm^{-3}	1.279
μ/mm^{-1}	0.086
absorption correction	Multi-Scan
transmission factor range	0.8996–0.9705
refls. measured	11364
R_{int}	0.0360
mean σ(*I*)/*I*	0.0502
θ range	3.473–28.278
observed refls.	3970
x, y (weighting scheme)	0.0409, 0.3301
hydrogen refinement	constr
Flack parameter	−0.1(6)
refls in refinement	4554
parameters	240
restraints	0
$R(F_{obs})$	0.0424
$R_w(F^2)$	0.0956
S	1.038
shift/error$_{max}$	0.001
max electron density/e Å^{-3}	0.269
min electron density/e Å^{-3}	−0.245

Antibacterial assays

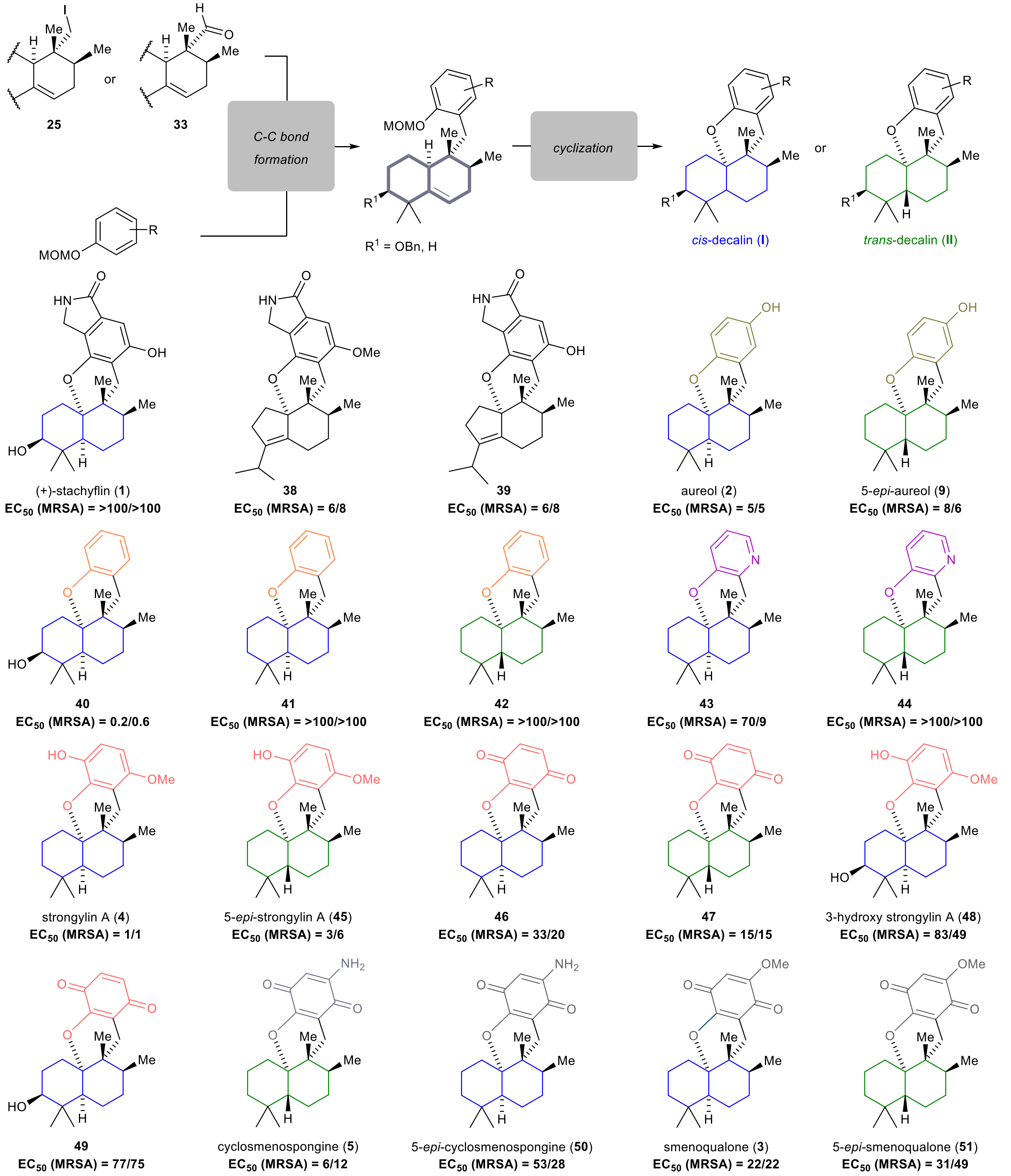

Supplementary Figure 67 Color-coding was used to indicate the decalin stereochemistry (blue = *cis*-decalin, green = *trans*-decalin) and to highlight the modified arene component. The effective concentrations (EC_{50} values) that inhibited the growth of two MRSA strains (DSM 11822/RKI 11-02670) are given in µM.

Supplementary Table 21 Antibacterial activities of meroterpenoids against Gram-positive pathogens[1)]. EC_{50} and MIC values are given in µM.

	MRSA DSM		***MRSA RKI***		***E. faecium***	
**Cmpd no**	**MIC**	**EC_{50}**	**MIC**	**EC_{50}**	**MIC**	**EC_{50}**
**1**	>100	>100	>100	>100	>100	>100
**2**	10	5	5	5	12	8
**3**	76	22	56	22	>100	14
**4**	1.4	1	2	1	>100	>100
**5**	13	6	15	12	>100	20
**9**	11	8	10	6	10	8
**38**	13	6	11	8	100	15
**39**	15	6	12	8	100	15
**40**	0.8	0.2	4	0.6	>100	>100
**41**	>100	>100	>100	>100	>100	>100
**42**	>100	>100	>100	>100	>100	>100
**43**	100	70	12	9	>100	>100
**44**	>100	>100	>100	>100	>100	>100
**45**	6	3	6	6	35	13
**46**	47	33	22	20	>100	80
**47**	29	15	22	15	52	32
**48**	>100	83	94	49	>100	>100
**49**	>100	77	>100	75	>100	100
**50**	77	53	37	28	>100	>100
**51**	46	31	68	49	>100	13
Linezolid	1.3	0.6	0.6	0.2		
Ciprofloxacin					20	7

1) All compounds were inactive (>100 µM) against *Acinetobacter baumannii* (DSM 30007), *Escherichia coli* (DSM 1116), *Klebsiella pneumoniae* (DSM 11678) and *Pseudomonas aeruginosa* PA7 (DSM 24068).

Antiproliferative assays

Supplementary Table 22 Antiproliferative activities of meroterpenoids against four mammalian cell lines. EC_{50} values are given in µM.

Cmpd no	L929	KB-3-1	MCF-4	FS4-LTM
**1**	51	51	32	>100
**2**	>100	16	14	25
**3**	30	25	15	61
**4**	49	12	12	25
**5**	14	12	27	26
**9**	14	11	23	33
**38**	19	25	15	25
**39**	34	29	23	26
**40**	7	7	9	14
**41**	>100	>100	>100	>100
**42**	>100	>100	>100	>100
**43**	40	20	13	21
**44**	>100	75	>100	>100
**45**	>100	40	27	>100
**46**	9	24	13	32
**47**	3	13	13	>100
**48**	45	87	43	69
**49**	10	21	8	16
**50**	54	24	28	46
**51**	27	56	25	45
Auranofin	1.2	1.2	1.1	1.7
Staurosporine	0.5	2.2	1.8	2.0

2

Supplementary References

1. Still, W. C., Kahn, M. & Mitra, A. Rapid Chromatographic Technique for Preparative Separations with Moderate Resolution. *J. Org. Chem.* **43,** 2923–2925 (1978).
2. Kofron, W. G. & Baclawski, L. M. A Convenient Method for Estimation of Alkyllithium Concentrations. *J. Org. Chem.* **41,** 1879–1880 (1976).
3. Altomare, A. *et al. SIR* 97: a new tool for crystal structure determination and refinement. *J. Appl. Crystallogr.* **32,** 115–119 (1999).
4. Sheldrick, G. M. A short history of SHELX. *Acta Cryst.* **A64,** 112–122 (2007).
5. Ishiyama, M. *et al.* A combined assay of cell viability and in vitro cytotoxicity with a highly water-soluble tetrazolium salt, neutral red and crystal violet. *Biol. Pharm. Bull.* **19,** 1518–1520 (1996).
6. Vollbrecht, L. *et al.* Argyrins, immunosuppressive cyclic peptides from myxobacteria I. Production, Isolation, Physico-chemical and Biological Properties. *J. Antibiot.* **55,** 543–551 (2002).
7. Quintiliano, S. A. P. & Silva, L. F. Practical synthesis of a functionalized 1-oxo-1,2,3,4-tetrahydroisoquinoline-3-carboxylic acid. *Tetrahedron Lett.* **53,** 3808–3810 (2012).
8. Miyata, O., Shinada, T., Ninomiya, I. & Naito, T. Asymmetric construction of two contiguous stereocenters by diastereoface differentiating addition reaction of thiols to chiral imides: Formal synthesis of (+)-diltiazem. *Tetrahedron* **53,** 2421–2438 (1997).
9. Fukaya, K. *et al.* Synthesis of paclitaxel. 1. Synthesis of the ABC ring of Paclitaxel by SmI_2-Mediated Cyclization. *Org. Lett.* **17,** 2570–2573 (2015).
10. Seyferth, D. & Stone, F. G. A. Vinyl Derivatives of the Metals. I. Synthesis of Vinyltin Compounds. *J. Am. Chem. Soc.* **79,** 515–517 (1957).
11. Mathre, D. J. *et al.* A Practical Enantioselective Synthesis of α,α-Diaryl-2-pyrrolidinemethanol. Preparation and Chemistry of the Corresponding Oxazaborolidines. *J. Org. Chem.* **56,** 751–762 (1991).
12. Yeung, Y. Y., Chein, R. J. & Corey, E. J. Conversion of Torgov's synthesis of estrone into a highly enantioselective and efficient process. *J. Am. Chem. Soc.* **129,** 10346–10347 (2007).
13. Williams, D. B. G. & Lawton, M. Drying of organic solvents: Quantitative evaluation of the efficiency of several desiccants. *J. Org. Chem.* **75,** 8351–8354 (2010).
14. Sakurai, J., Kikuchi, T., Takahashi, O., Watanabe, K. & Katoh, T. Enantioselective Total Synthesis of (+)-Stachyflin: A Potential Anti-Influenza A Virus Agent Isolated from a Microorganism. *Eur. J. Org. Chem.* 2948–2957 (2011).
15. Minagawa, K. *et al.* Stachyflin and Acetylstachyflin, Novel Anti-influenza A Virus Substances, Produced by Stachybotrys sp. RF-7260. *J. Antibiot.* **55,** 155–164 (2002).
16. Edwards, O. E., Dixon, J., Elder, J. W., Kolt, R. J. & Lesage, M. Protonated cyclopropanes in α-aminoketone deamination. *Can. J. Chem.* **59,** 2096–2115 (1981).
17. Tanis, S. P. & Abdallah, Y. M. A Study of the Diels-Alder Regioselectivity of Substituted Vinyl Cyclohexenes. *Synth. Commun.* **16,** 251–259 (1986).
18. Buter, J. *et al.* Stereoselective Synthesis of 1-Tuberculosinyl Adenosine; A Virulence Factor of Mycobacterium tuberculosis. *J. Org. Chem.* **81,** 6686–6696 (2016).
19. Shi, Z. F., Wang, L. J., Wang, H., Cao, X. P. & Zhang, H. L. Synthesis of oligo(phenylene ethynylene)s with dendrimer 'shells' for molecular electronics. *Org. Lett.* **9,** 595–598 (2007).
20. Djura, P. *et al.* Some Metabolites of the Marine Sponges Smenospongia aurea and Smenospongia (Polyfibrospongia) echina. *J. Org. Chem.* **45,** 1435–1441 (1980).
21. Speck, K., Wildermuth, R. & Magauer, T. Convergent Assembly of the Tetracyclic Meroterpenoid (–)-Cyclosmenospongine by a Non-Biomimetic Polyene Cyclization. *Angew. Chemie Int. Ed.* **55,** 14131–14135 (2016).

22. Kabalka, G. W., Reddy, N. K. & Narayana, C. Sodium percarbonate: A convenient reagent for the dakin reaction. *Tetrahedron Lett.* **33,** 865–866 (1992).

23. Wright, A. E., Rueth, S. A. & Cross, S. S. An Antiviral Sesquiterpene Hydroquinone from the Marine Sponge Strongylophora Hartmani. *J. Nat. Prod.* **54,** 1108–1111 (1991).

24. Schmidt, B., Krehl, S., Kelling, A. & Schilde, U. Synthesis of 8-Aryl-Substituted Coumarins Based on Ring-Closing Metathesis and Suzuki-Miyaura Coupling: Synthesis of a Furyl Coumarin Natural product from Galipea panamensis. *J. Org. Chem.* **77,** 2360–2367 (2012).

25. Batsomboon, P., Phakhodee, W., Ruchirawat, S. & Ploypradith, P. Generation of ortho-Quinone Methides by p-TsOH on Silica and Their Hetero-Diels–Alder Reactions with Styrenes. *J. Org. Chem.* **74,** 4009–4012 (2009).

26. Bernet, A. & Seifert, K. A New Approach to Sesquiterpene Arenes of the 9, 11-Drimenyl Type (=[(1E,2RS,4aRS,8aRS)-Octahydro-2,5,5,8a-tetramethylnaphthalen-1(2H)-ylidene] methyl Type). *Helv. Chim. Acta* **89,** 784–796 (2006).

27. Bourguet-Kondracki, M.-L., Martin, M.-T. & Guyot, M. Smenoqualone a Novel Sesquiterpenoid from the Marine Sponge Smenospongia sp. *Tetrahedron Lett.* **33,** 8079–8080 (1992).

28. Türkmen, Y. E. & Rawal, V. H. Exploring the potential of diarylacetylenediols as hydrogen bonding catalysts. *J. Org. Chem.* **78,** 8340–8353 (2013).

29. Mongin, F., Trécourt, F., Mongin, O. & Quéguiner, G. New syntheses of orelline and analogues via metalation and cross-coupling reactions. *Tetrahedron* **58,** 309–314 (2002).

30. Shen, L., Xie, N., Yang, B., Hu, Y. & Zhang, Y. Design and total synthesis of Mannich derivatives of marine natural product lamellarin D as cytotoxic agents. *Eur. J. Med. Chem.* **85,** 807–817 (2014).

31. Swersey, J. C., Barrows, L. R. & Ireland, C. M. Mamamuthaquinone: An Antimicrobial and Cytotoxic Metabolite of Fasciospongia sp. *Tetrahedron Lett.* **32,** 6687–6690 (1991).
